# Discovery and preliminary SAR of 14-aryloxy-andrographolide derivatives as antibacterial agents with immunosuppressant activity†

**DOI:** 10.1039/c8ra01063c

**Published:** 2018-03-06

**Authors:** Feng Li, Xiao-Min Li, Dekuan Sheng, Shao-Ru Chen, Xin Nie, Zhuyun Liu, Decai Wang, Qi Zhao, Yitao Wang, Ying Wang, Guo-Chun Zhou

**Affiliations:** School of Pharmaceutical Sciences, Nanjing Tech University Nanjing 211816 PR China gczhou@njtech.edu.cn dcwang@njtech.edu.cn +86-25-58139415; State Key Laboratory of Quality Research in Chinese Medicine, Institute of Chinese Medical Sciences, University of Macau Avenida da Universidade, Taipa Macao SAR PR China emilyywang@umac.mo; Faculty of Health Sciences, University of Macau Avenida da Universidade, Taipa Macao SAR PR China

## Abstract

Antibacterials (which restore gut flora balance) and immunosuppressants (which correct immune defects) are two important and effective therapeutic agents for the treatment of inflammatory bowel disease (IBD) in clinical use today. Since the structural skeleton of andrographolide, isolated from *Andrographis paniculata*, has become known as a natural antibiotic with anti-inflammation and heat-clearing and detoxifying properties, 14-aryloxy andrographolide derivatives have been designed, synthesized, and tested for their antibacterial effects on *E. coli*, *S. aureus*, and *E. faecalis*, which are related to IBD. It has been discovered in this study that the andrographolide skeleton is more selective against *E. faecalis*, the 14-aryloxy group with basicity is important for antibacterial functions, and the 14-(8′-quinolinyloxy) group is a good pharmacophore with antibacterial activity. In addition, we found that 7b1 and 8b1 are good and selective inhibitors of *E. faecalis*; two 14β-(8′-quinolinyloxy) andrographolide derivatives, 6b17 and 9b, exhibit good activity against *E. coli*, *S. aureus*, and *E. faecalis*. Likewise and importantly, further exploration of immunosuppressant activity for IBD shows that compound 7b1 is a selective inhibitor of the TNF-α/NF-κB signaling pathway, whereas 8b1 is selectively active against the TLR4/NF-κB signaling pathway; moreover, the compounds 6b17 and 9b are active in inhibiting the IL-6/STAT3, TLR4/NF-κB, and TNF-α/NF-κB signaling pathways. Based on these results, we have further focused on the development of dual function inhibitors of IBD as antibacterial and immunosuppressant agents by structural modification of andrographolide.

## Abstract 2

6b17 and 9b are active against 3 tested bacteria while they block IL-6/STAT3, TLR4/NF-κB and TNF-α/NF-κB pathways. 7b1 and 8b1 are selectively active against *E. faecalis* but 7b1 selectively blocks TNF-α/NF-κB pathway and 8b1 selectively blocks TLR4/NF-κB pathway.

## Graphical abstract

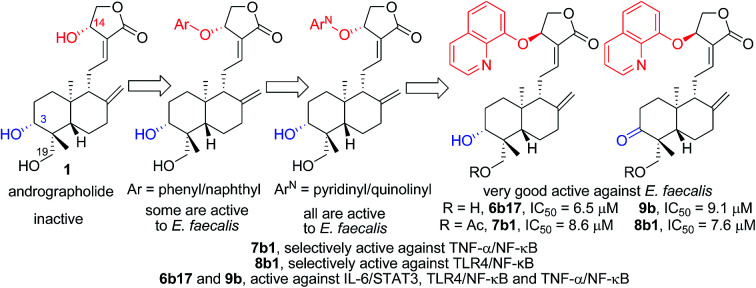

## 1. Introduction

Inflammatory bowel disease (IBD)^1^ is an autoimmune disease that is characterized by relapsing and remitting chronic inflammation of the gastrointestinal tract; it is believed to be caused by unbalanced host-commensal microbiota and a common immune defect in addition to genetic predisposition.^2^ Immune responses have been recognized to be related to IBD pathogenesis,^3^ and a recent study demonstrated that the innate immune system is a major determinant of the serum and tissue profiles of IBD,^4^ further explaining the existence of regulatory cytokines that are implicated in immune responses related to IBD.^5^ Especially, overexpression of proinflammatory cytokines, such as interleukin-6 (IL-6), toll-like receptor 4 (TLR4), and tumor necrosis factor-α (TNF-α), is crucial for IBD onset and progression; these proinflammatory cytokines, as inflammatory mediators, are connected with the activation of nuclear factor of κ light polypeptide gene enhancer in B-cells (NF-κB) and signal transducer and activator of transcription 3 (STAT3).^5^ As NF-κB and STAT3 are known to play central roles in regulating inflammatory responses in patients with IBD, they are recognized as important targets for therapeutic intervention of IBD.^6^ Gastrointestinal microbiota are believed to be commensal and mutualistic to humans and animals and to have health benefits for humans and animals in some aspects.^7^ Significant imbalances of gastrointestinal flora are observed in patients with IBD as compared to the case of healthy people; for example, proteobacteria and actinobacteria appear to dominate in people with ulcerative colitis (UC), whereas *Enterococcus* (*E.*) *faecium* and several proteobacteria over-inhabit the gastrointestinal tracts of people with Crohn's disease (CD).^8^ Although the etiology and pathogenesis of IBD are not fully understood, *E. coli* and *E. faecalis* are recognized as pathogens of IBD.^9^ It is controversial whether *S. aureus* infection is involved in early lesions or established lesions of IBD; however, *S. aureus* infection occasionally may occur and complicate IBD during the course of the disease.^10^ Metabolites from certain members of the gut flora may have causal contributions to disorders such as obesity and colon cancer by changing host signaling pathways.^11^ In addition, the intrusion of gut flora components into other host compartments can lead to sepsis.^11^ Therefore, immunosuppressants (which correct immune defects) and antibacterials (which restore flora balance) are two important and effective therapeutic agents to treat IBD in clinical use. Considering this, we envisage that it is a meaningful exploration to discover single compounds with dual functions of antibacterial and immunosuppressant activities.

**Scheme 1:**
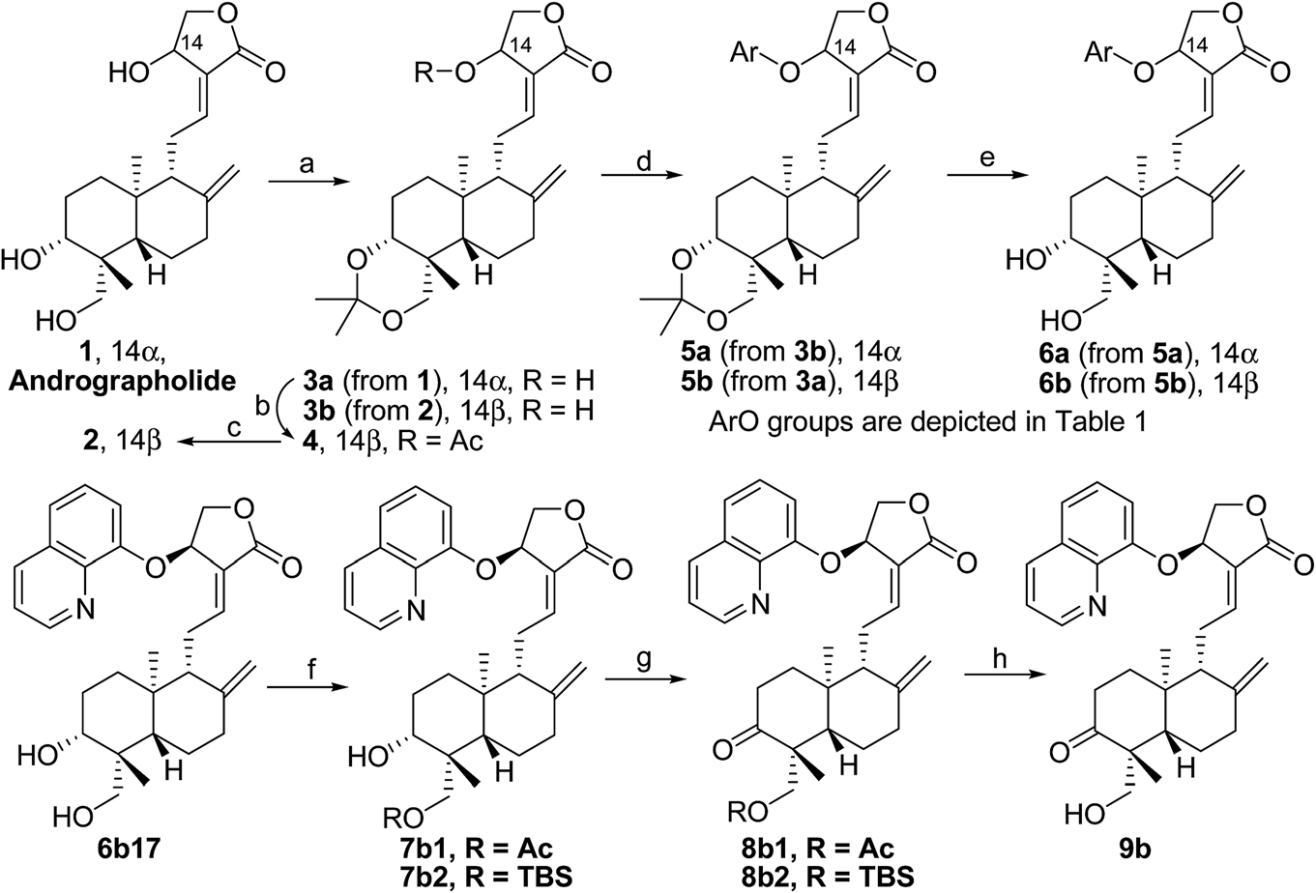
Reagents and conditions (andro = andrographolide): (a) anhydrous DCM, 2,2-dimethoxypropane, PPTS, 40 °C; (b) anhydrous THF; 3a (1.0 eq.), anhydrous HOAc (1.5 eq.), DIAD (1.5 eq.), PPh_3_ (1.5 eq.), 0 °C to room temperature; (c) MeOH/H_2_O (4/1), TsOH·H_2_O, 40 °C. (d) anhydrous THF; 3a or 3b (1.0 eq.), phenol (1.5 eq.), DIAD (1.5 eq.), PPh_3_ (1.5 eq.), 0 °C to room temperature; (e) MeOH/H_2_O (4/1), TsOH·H_2_O, 20 °C; (f) AcCl, TEA, 0 °C, 90% yield for 7b1 or TBSCl, TEA, rt, 89% yield for 7b2; (g) DCM, Dess–Martin periodinane, rt, 90% yield for 8b1 or 86% yield for 8b2; (h) *p*-TSA, MeOH, 40 °C, 8 h, 80% yield from 8b1 or DCM, TFA, −20 °C, 20 min, 81% yield from 8b2.

*Andrographis paniculata* [Burm. F.] Nees, an herb known as a “natural antibiotic”, is commonly used in China, India and Southeast Asia for the treatment of a large variety of illnesses, especially infectious diseases, by reducing inflammation and “heat-clearing and detoxifying”. Andrographolide (1, Scheme 1), a bicyclic diterpenoid lactone, is a major active component isolated from *Andrographis paniculata* [Burm. F.] Nees;^12^ its derivatives,^13^ such as “Chuanhuning”,^14,15^ have been used in China to treat bacterial and viral infections for many years. Numerous andrographolide derivatives have been designed and synthesized in recent years.^16^ The antibacterial activities of andrographolide and its derivatives are not potent and show only minimal or marginal direct inhibition of bacterial growth; however, it was discovered that andrographolide inhibits the quorum sensing (QS) system^17^ of *P. aeruginosa* and that andrographolide derivatives block biofilm formation^18^ of *P. aeruginosa*, indicating that andrographolide and its derivatives can directly inhibit bacterial growth or/and infection by specific mechanisms and modes of action. On the other hand, some evidence has been found that the anti-inflammatory effects of andrographolide are related to regulation of the immune system^19^ and that andrographolide suppresses TLR4 expression and NF-κB signaling in multiple myeloma cells.^20^ Multi-targeting andrographolide and its analogs have potential use in the prevention and treatment of metabolic syndrome^21^ and stroke^22^*via* the NF-κB signaling pathway. The andrographolide derivative isoandrographolide significantly inhibited the release of NO and prostaglandin E2 and the production of interleukin-1β (IL-1β) and IL-6 in lipopolysaccharide (LPS)-stimulated J774A.1 macrophage cells in a dose-dependent manner.^23^ Andrographolide analog AL-1 improved insulin resistance by down-regulating the NF-κB signaling pathway.^24^ Our previous report^25^ revealed that some analogs of andrographolide play a role in the attenuation of innate immunity; these were identified as potential immunomodulatory inhibitors of TLR signaling with distinct regulation of NF-κB family members. Furthermore, one compound effectively reduced LPS-induced pulmonary injury in a mouse *in vivo* study; biochemical results showed that the nucleus translocation of phosphorylated p65 and serum pro-inflammatory cytokines decreased. Based upon these facts, we are interested in developing dual-function analogs of andrographolide as immunosuppressants to inhibit excessive immunity to “self” gastrointestinal microbiota and also as antibacterial agents to control infection by specific “commensal/mutualistic” gut flora; such compounds should be valuable in the treatment of IBD.

In our search for dual-functional 14-aryloxy andrographolide analogs inhibiting both bacterial growth and innate immunity, we launched a panel of screening platforms of these analogs to study their antibacterial activities against *E. coli*, *S. aureus* and *E. faecalis* and preliminary structure–activity relationship (SAR) studies as the first step. Selected active anti-bacterial compounds were explored as immunosuppressants to protect the host from abnormal immune response initiated by intestinal commensal/mutualistic bacteria. In this paper, we describe the synthesis and evaluation of a series of 14-aryloxy andrographolide compounds against *E. coli*, *S. aureus* and *E. faecalis* as well as against the IL-6/STAT3, TLR4/NF-κB and TNF-a/NF-κB signaling pathways of innate immune response and their preliminary SAR. It was discovered that analogs of andrographolide are more sensitive to *E. faecalis* and that active antibacterial compounds are greatly superior to andrographolide to attenuate innate immune response. Our results revealed that the andrographolide skeleton has immunosuppressant and antibacterial properties and that these analogs of andrographolide can potentially be used to treat IBD.

## 2. Results and discussion

### 2.1. Synthesis

The title derivatives were synthesized according to our previously reported synthesis;^25,26^ the synthesis is outlined in Scheme 1 (ArO groups are depicted in Table 1). Accordingly, 14α-3,19-acetonylidene-protected andrographolide (3a) was prepared by the reaction of andrographolide (1) with 2,2-dimethoxypropane catalyzed by PPTS, followed by a Mitsunobu reaction with acetic acid, which inverted 14α-OH of 3a into 14β-OAc of 4. Full hydrolysis of 4 by TsOH·H_2_O in MeOH/H_2_O (4/1) at 40 °C afforded 2 as the 14β-epimer of andrographolide; then, 3,19-protection of 2 afforded 14β-3,19-acetonylidene andrographolide (3b).

**Table 1:** Antibacterial activities of the synthesized compounds against *E. coli*, *S. aureus* and *E. faecalis*^a^^,^^b^

Cmpd	ArO	IC_50_ (μM)^c^ (inhibition rate (%)^d^)			Cmpd	ArO	IC_50_ (μM) (inhibition rate (%))		
		EC	SA	EF			EC	SA	EF
5a1	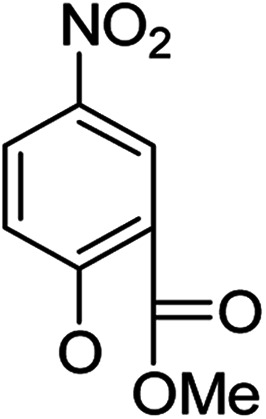	/	(14)	51.2 ± 11.6	5a8	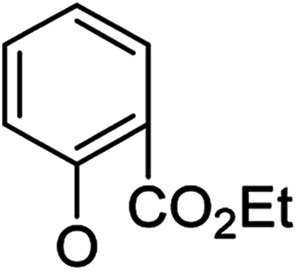	(32)	(20)	(33)
5b1		(22)	(20)	(56)	5b8		/	/	(22)
6a1		/	(10)	72.2 ± 9.2	6a8		(21)	(17)	83.3 ± 8.3
6b1		(17)	(40)	27.7 ± 3.5	6b8		(21)	/	89.7 ± 12.0
5a2	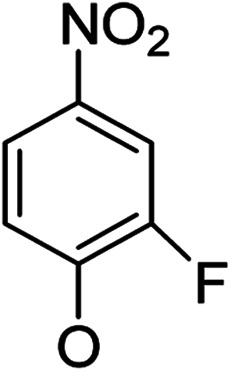	(20)	(20)	56.7 ± 6.5	5a9	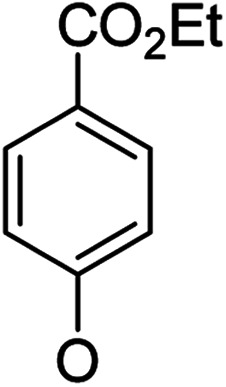	/	(10)	(45)
5b2		(11)	(26)	(56)	5b9		/	(15)	(42)
6a2		(67)	(65)	38.7 ± 4.5	6a9		/	/	38.3 ± 5.9
6b2		(42)	(25)	24.4 ± 3.2	6b9		(17)	(40)	53.3 ± 4.1
5a3	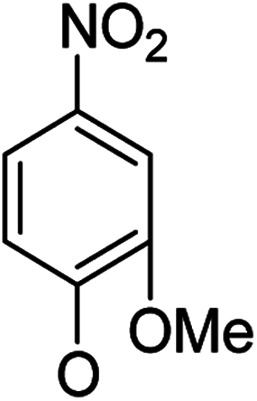	(21)	(18)	(60)	5b10	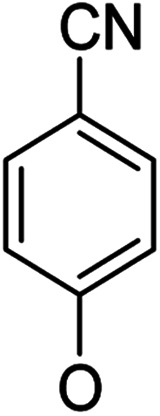	(20)	(22)	(46)
5b3		/	(16)	(19)	6b10		(39)	(29)	72.1 ± 9.4
6a3		(31)	(17)	112.9 ± 14.3	5b11	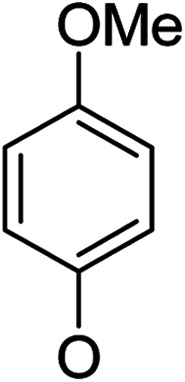	/	/	(39)
6b3		(9)	(44)	140.3 ± 11.9	6b11		/	/	(17)
5a4	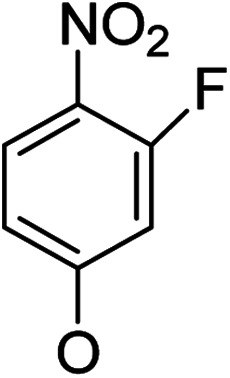	/	/	(46)	5a12	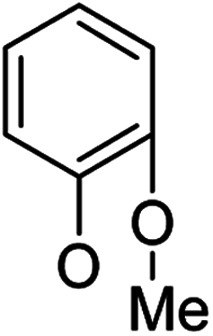	/	/	(49)
5b4		(19)	(15)	(73)	5b12		/	(24)	(52)
6a4		/	/	(44)	6a12		/	(14)	75.6 ± 17.8
6b4		(60)	(28)	19.6 ± 2.7	6b12		(12)	(13)	20.8 ± 4.5
5a5	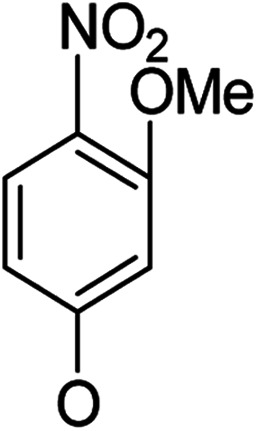	(12)	(10)	(73)	5b13	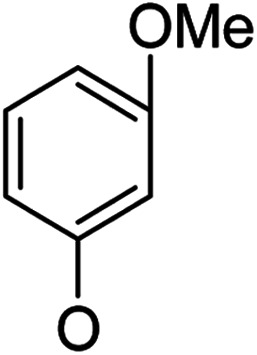	/	/	(17)
5b5		(18)	(32)	(34)	6b13		15	13	137.8 ± 5.4
6a5		(11)	(10)	50.4 ± 2.7	5b14	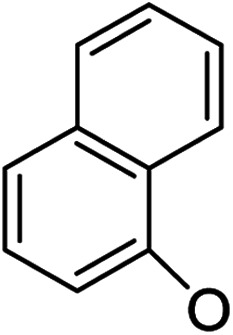	/	/	(47)
6b5		(23)	(37)	21.8 ± 2.5	6b14		(35)	(18)	(50)
5b6	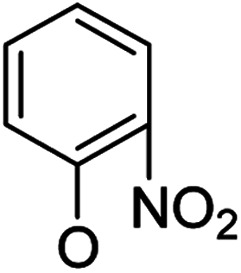	/	/	43	5a15	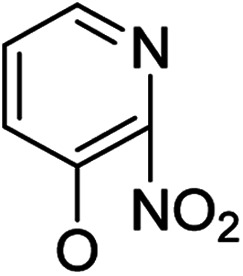	(13)	(11)	25.5 ± 1.5
6b6		(28)	(12)	15.9 ± 9.0	5b15		/	(33)	38.4 ± 3.4
5b7	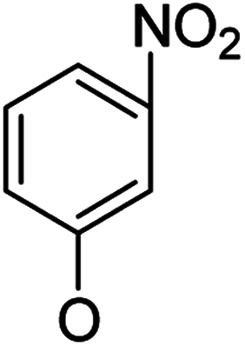	/	/	/	6a15		(14)	(13)	56.7 ± 5.5
6b7		/	26	17.8 ± 4.2	6b15		(14)	(41)	29.3 ± 2.1
5a16	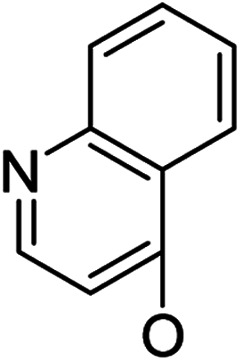	/	(14)	16.9 ± 5.9	6b17	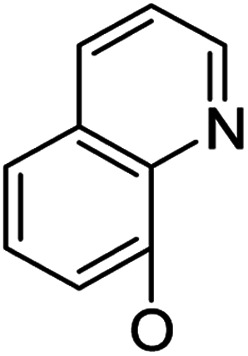	21.6 ± 1.7	37.5 ± 2.2	6.3 ± 1.5
5b16		/	/	15.5 ± 4.2	7b1		(60)	(58)	8.6 ± 0.7
6a16		/	(22)	65.8 ± 13.5	7b2		(24)	(23)	26.2 ± 2.8
6b16		/	/	58.5 ± 4.2	8b1		(34)	(42)	7.6 ± 0.8
5a17	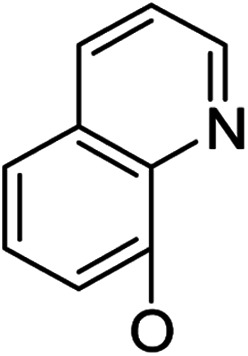	(44)	(35)	10.4 ± 2.5	8b2		(25)	(28)	(71)
5b17		(47)	(27)	19.3 ± 2.3	9b		27.9 ± 1.5	27.6 ± 1.8	9.1 ± 2.1
6a17		(70)	(66)	16.3 ± 2.1	Ciprofloxacin		0.31 ± 0.06	0.4 ± 0.06	2.3 ± 0.6

a EC: *E. coli*; SA: *S. aureus*; EF: *E. faecalis*.

b “/”represents no inhibition or lower than 10% inhibition rate at 250 μM of tested compound.

c IC_50_ value was generated when the inhibition rate at 250 μM of the tested compound was higher than 80%.

d The inhibition rate at 250 μM of the tested compound is indicated in parentheses.

Mitsunobu reactions of 3a or 3b with varied aromatic phenols were carried out in general from 0 °C to room temperature in anhydrous THF to afford 14β-aryloxy andrographolide 5b (5b1–5b17) or 14α-aryloxy andrographolide 5a (5a1–5a5, 5a8–5a9, 5a12, 5a15–5a17), respectively, in mild to moderate isolated yield depending on the distinct properties of the phenols. Deprotection of 3,19-acetonylidene from 5a or 5b afforded the corresponding alcohol 6a (6a1–6a5, 6a8–6a9, 6a12, 6a15–6a17) or 6b (6b1–6b17). As we noted before,^26^ 14α-aryloxy derivatives are generally much less stable than their corresponding 14β-isomers, and some 14α-aryloxy compounds were not obtained or were obtained in very low yields. Furthermore, mono-acetylation or mono-silylation of 6b17 produced 7b1 (R = Ac) or 7b2 (R = TBS), which was oxidized into ketone 8b1 or 8b2 by Dess–Martin periodinane (DMP); finally, deprotection of 8b1 or 8b2 afforded 3-keto-19-alcohol product 9b.

### 2.2. Antibacterial activity and SAR analysis

Numerous bacteriostatic and bactericidal agents have been discovered and developed against bacterial growth and infection; however, bacterial resistance to currently used antibacterial drugs is becoming a major problem in clinical practice, especially hospital-acquired infections by these drug-resistant bacteria.^27^ To meet the demand of clinical medication for the treatment of digestive diseases caused by gastrointestinal bacterial infection, antibacterial drugs with diverse and novel structural origins are required.^28^

On the basis of reported literature studies stating that andrographolide and some of its derivatives may function as antibiotics, we are pursuing the discovery of more potent and specific andrographolide derivatives for the development of chemotherapeutical agents against bacterial infection, especially for inhibiting gastrointestinal bacterial infection in the development of IBD. The strategy in this study was to submit these synthesized compounds to a preliminary screening test against *E. coli*, *S. aureus* and *E. faecalis* (ciprofloxacin was used as a reference compound; see Table 1). Because these andrographolide analogs did not obviously function as bactericidal agents (data not shown), bacteriostatic screening was employed with gradient concentrations and the percent growth was plotted *versus* test concentration to afford the IC_50_ values.^29^ The current antibacterial activity results are listed in Table 1.

In our study streamline, 5 series of 20 compounds bearing 14-(4′-nitro)phenoxy groups were first tested. It was found that all of these compounds are more selective against *E. faecalis* than against *E. coli* and *S. aureus*. 14β-3,19-Diol compounds (6b1–6b5) are more active against *E. faecalis* than the corresponding 14β-3,19-acetonylidene protected compounds (5b1–5b5). Furthermore, 14β-(3′-substituted 4′-nitro)phenoxy-3,19-diol compounds 6b4 and 6b5 are more sensitive to *E. faecalis* than 14β-(2′-substituted 4′-nitro)phenoxy-3,19-diol compounds 6b1, 6b2 and 6b3. It was observed that the activity patterns of the 14-(2′-fluro-4′-nitro)phenoxy and 14-(2′-carboxylate-4′-nitro)phenoxy derivatives are quite similar. The activity differences between 14α-(2′-substituted-4′-nitro)phenoxy groups and 14β-(2′-substituted-4′-nitro)phenoxy groups are variable, leading to an order of 5a1 > 5b1, 5a2 > 5b2, 6b1 > 6a1 and 6b2 > 6a2 when an electron-withdrawing group (EWG) is at the 2′-position but 6b3 < 6a3 when an electron-donating group (EDG) is at the 2′-position. Overall, an EWG at the 2′-position (5a1, 5a2, 6a1, 6a2, 6b1 and 6b2) was more favorable for antibacterial activity than an EDG at the 2′-position (5a3, 6a3 and 6b3). Compounds 6b4 and 6b5 with 14β-(3′-substituted 4′-nitro)phenoxy groups are more active to *E. faecalis* than the corresponding compounds 6a4 and 6a5 with 14α-(3′-substituted 4′-nitro)phenoxy groups, respectively.

Then, 2′-nitro compounds and 3′-nitro compounds were explored; it was discovered that the 14β-3,19-diol isomers of 14-(2′-nitro)phenoxy analog 6b6 and 14-(3′-nitro)phenoxy analog 6b7 exhibited mild inhibitory activity to *E. faecalis* and were more active than the corresponding 3,19-acetonylidene protected compounds 5b6 and 5b7. 14α- and 14β-(2′-Carboxyl ester)phenoxy-3,19-diol analogs of 6a8 and 6b8 possessed similar but weak inhibitory activities to *E. faecalis*, indicating that 14-stereochemistry is not the key to their activity; meanwhile, their 3,19-acetonylidene protected analogs 5a8 and 5b8 were almost inactive. We compared the 14-(2′-carboxyl ester 4′-nitro)phenoxy analogs 6a1 and 6b1 with the 14-(2′-carboxyl ester)phenoxy analogs 6a8 and 6b8; the results suggested that the 4′-nitro group enhanced antibacterial activity against *E. faecalis*. A similar inhibition tendency was observed for 4′-carboxyl ester phenoxy analogs, where the 4′-carboxyl ester phenoxy-3,19-diol analogs of 6a9 and 6b9 exhibited weak activity against *E. faecalis*, whereas 14α-isomer 6a9 was slightly more active than 14β-isomer 6b9 against *E. faecalis*. However, the 4′-carboxyl ester phenoxy analogs 6a9 and 6b9 were more active against *E. faecalis* than the 2′-carboxyl ester phenoxy counterparts 6a8 and 6b8. Introduction of a cyano (CN) EWG at the 4′-position did not improve the antibacterial activity, and only 14β-isomer 6b10 showed very weak activity against *E. faecalis*. As above, all of these analogs were very weakly active or inactive against *E. coli* and *S. aureus*.

On the other hand, OMe as an EDG occupying the 2′-, 3′- or 4′-position afforded different results. 4′-OMe derivatives 5b11 and 6b11 showed very weak activity against *E. faecalis* and no antibacterial activity against *E. coli* and *S. aureus*; it is possible that 4′-EDG substitution was not as beneficial to antibacterial activity as 4′-EWG substitution, according to the comparison of 4′-OMe derivatives 5b11 and 6b11 with the 4′-CO_2_Et and 4′-CN derivatives 6a9, 6b9 and 6b10. 14β-(3′-OMe) derivative 6b13 was very weakly active, while its corresponding 14β-(3′-OMe-4′-nitro) analog 6b5 possessed mild inhibitory activity toward *E. faecalis*, indicating that the 4′-nitro group enhanced the inhibitory activity toward *E. faecalis*. 14α-2′-OMe-3,19-diol derivative 6a12 was weakly active against *E. faecalis* and 6b12 (14β) showed mild inhibitory activity toward *E. faecalis*, suggesting that the 14β-isomer is more stereochemically active for antibacterial activity. Meanwhile, the 4′-nitro group negatively influenced inhibitory activity toward *E. faecalis*; 6a12 and 6b12 were more active against *E. faecalis* than corresponding the 4′-nitro analogs 6a3 and 6b3, respectively.

Based on the above data, further exploration to introduce more complex aryl groups at the 14-position were conducted. 14β-(1′-Naphthyloxy) compound 5b14 exhibited very weak inhibitory activity only to *E. faecalis*, and 14β-(1′-naphthyloxy)diol compound 6b14 expressed very weak activity against all three bacteria. Interestingly, the introduction of a 2′-nitro-pyridinyl-3-oxy group at the 14-position increased the activity of all four derivatives 5a15, 5b15, 6a15 and 6b15 against *E. faecalis*; 14α-3,19-acetonylidene analog 5a15 was the most active, while 14α-3,19-diol analog 6a15 was the least active. Moreover, 5b15 was more active than its corresponding 2′-nitro phenoxy derivative 5b6, but 6b15 was less active than 2′-nitro phenoxy analog 6b6. Corresponding to the replacement of phenoxy groups with pyridinyloxy groups, substitution of naphthyloxy by quinolinyloxy was subsequently studied. Unlike 14β-(1′-naphthyloxy) analogs 5b14 and 6b14, four analogs of 14-(4′-quinolinyloxy) derivatives 5a16 (clog *P* 6.8988), 5b16 (clog *P* 6.8988), 6a16 (clog *P* 4.8578) and 6b16 (clog *P* 4.8578) expressed antibacterial activity against *E. faecalis*; however, none of these was active against *E. coli*, and two 14α-isomers, 5a16 and 6a16, were very weak inhibitors of *S. aureus*. The clog *P* values of 3,19-protected compounds 5a16 and 5b16 were higher than 5.0 and higher than those of 6a16 and 6b16; 5a16 and 5b16 were more active against *E. faecalis* than the corresponding 3,19-diol compounds 6a16 and 6b16, respectively. Unlike the activity relationships between 5a15, 5b15, 6a15 and 6b15, the activity variation patterns against *E. faecalis* of 5a16 and 5b16 were parallel to those of 6a16 and 6b16 and 14β-isomers 5a16 and 6a16 were slightly more active than 14α-isomers 5b16 and 6b16, respectively. These data imply that a positive charge of nitrogen in the 14-aryloxy group under physiological conditions may play a role in antibacterial activity for the substitutions of phenoxy groups by pyridinyloxy groups and of naphthyloxy groups by quinolinyloxy groups.

Continued exploration in the quinolinyloxy line disclosed that 14-(8′-quinolinyloxy) derivatives exhibited novel antibacterial activity profiles. In contrast to the 14-(4′-quinolinyloxy) derivatives, the 14-(8′-quinolinyloxy) derivatives were active against all three bacteria; they also showed more selective inhibition of *E. faecalis* than of *E. coli* or *S. aureus*, similar to the other series of 14-aryloxy andrographolide derivatives in this work. Specifically, 14α-3,19-acetonylidene compound 5a17 (clog *P* 6.77355) was more active against *E. faecalis* than 14α-3,19-diol compound 6a17 (clog *P* 4.73255), while 14β-3,19-acetonylidene compound 5b17 (clog *P* 6.77355) was less active against *E. faecalis* than 14β-3,19-diol compound 6b17 (clog *P* 4.73255); this activity pattern against *E. faecalis* is the same as that of 14-(2′-nitro-pyridinyl-3-oxy) derivatives 5a15, 5b15, 6a15 and 6b15. Importantly, 6b17 expressed good antibacterial activity against all three bacteria (Table 1) and was the most potent inhibitor of *E. faecalis* in this paper (Table 1). These data illustrate that the 14-(8′-quinolinyloxy) compounds showed superior results against all three bacteria compared to their corresponding 14-(4′-quinolinyloxy) compounds. 19-Acetylated 7b1 (clog *P* 5.64055) and its 3-ketone 8b1 (clog *P* 5.37725) had almost no inhibitory activity toward *E. coli* and *S. aureus* but were still very active against *E. faecalis*. However, 19-silylated 7b2 (clog *P* 8.10955) had decreased inhibitory activity toward all three bacteria and possessed only mild activity against *E. faecalis*, while the antibacterial activity of 3-ketone 8b2 (clog *P* 7.85675) was very weak against all three bacteria. It is interesting that 3-keto-19-alcohol compound 9b (clog *P* 4.47495) regained the same antibacterial activity against all three bacteria (Table 1) as 6b17, although its inhibitory activity toward *E. faecalis* was slightly less potent than that of 6b17. By combining these data, it is suggested that in addition to the importance of the andrographolide skeleton, the 14-(8′-quinolinyloxy) group is essential to antibacterial activity; moreover, modifications at the 3- or/and 19-positions are not ignorable, and 19-acetylation greatly reduced inhibitory activity to *E. coli* and *S. aureus* while transformation of 3-alcohols into 3-ketones slightly influenced the antibacterial activity.

### 2.3. Inhibition of innate immune response and SAR analysis

Considering that initiation of a dysregulated immune response within the intestinal mucosa by “self” gut flora and/or food is due to immune defects in IBD, reduction of over-immunity is an effective treatment of IBD.^3,30^ TNF-α, the best studied NF-κB activator, is correlated with transformation of NF-κB by various stimuli from its inactive form to its active form. TLRs are involved in the regulation of innate and adaptive immunity,^31^ and LPS-activated TLR4 regulates the expression and nucleus translocation of NF-κB.^32^ IL-6 is a crucial pathogenic mediator in IBD that functions by triggering cellular effects and functions *via* STAT3 signaling. The proinflammatory IL-6/STAT3-dependent biological network is upregulated in active IBD patients and is considered to be an important pathogenic factor in IBD onset^33^ and progression.^34^ Based on these facts, immunosuppressants, corticosteroids and anti-TNF-α antibodies have become commonly used drugs in IBD treatment; they may affect IBD progression by interfering with cellular oxidative stress and cytokine production.^35^

We utilized luciferase reporters bearing the promoter regions of TNF-α/NF-κB, TLR4/NF-κB, and IL-6/STAT3 (ref. 25) to examine the activities of the antibacterial active andrographolide analogs in the regulation of signaling pathways that govern inflammatory response (Table 2). It was noted that these derivatives did not show obvious cytotoxicity to AD-293 cells; DCB-3503 was used as a positive compound^36^ (Table 2). Our previous study revealed that compound 6b3 is active against the TNF-α/NF-κB and TLR4/NF-κB signaling pathways;^25^ however, 6b3 does not have antibacterial activity (Table 1). 14α-Compounds 5a17 and 6a17 are active against the IL-6/STAT3 signaling pathway but do not inhibit the NF-κB signaling pathways. 14β-Compounds 5b17 and 6b17 are active to suppress the three signaling pathways (Table 2), suggesting that 3,19-acetonylidene does not obviously affect the inhibition of these signaling pathways; although the feature of 3,19-acetonylidene may contribute to the distinct activities of 5b17 and 6b17 against *E. faecalis*, 6b17 is also 3 times more active than 5b17 against *E. faecalis*. Compounds 7b1 and 8b1 possess similar inhibitory activities to *E. faecalis*; however, 3-alcohol-19-acetylated compound 7b1 shows selective anti-TNF-α/NF-κB activity, while 3-keto-19-acetylated compound 8b1 is selectively active against the TLR4/NF-κB signaling pathway. Like their similarity in inhibitory activity against *E. faecalis*, *E. coli* and *S. aureus* (Table 1), 3-keto-19-alcohol compound 9b1 suppresses three signaling pathways in the same fashion as 6b17 (Table 2). These interesting data suggest that dual-functional analogs of andrographolide can behave as antibacterial and immunosuppressant agents and possibly have synergistic effects in IBD treatment.

**Table 2:** EC_50_ (half effective concentration) values of the antibacterially active compounds against the IL-6/STAT3, TLR4/NF-κB, and TNF-α/NF-κB signaling pathways and cytotoxicities (CC_50_, half cytotoxic concentration) in AD-293 cells

Entry	Cmpd	EC_50_ (μM)			CC_50_^c^ (μM)
		IL-6/STAT3^a^	TLR4/NF-κB^a^	TNF-α/NF-κB^b^	
1	1	>10	>10	>10	>10
2	6b3 (ref. 25)	>10	3.49 ± 0.34	8.97 ± 1.03	>10
3	5a17	4.71 ± 0.17	>10	>10	>10
4	5b17	6.35 ± 0.77	2.85 ± 0.07	2.45 ± 0.49	>10
5	6a17	5.62 ± 0.13	>10	>10	>10
6	6b17	2.41 ± 0.31	2.65 ± 0.07	2.00 ± 0.28	>10
7	7b1	>10	>10	4.91 ± 0.02	>10
8	8b1	>10	4.84 ± 0.31	>10	>10
9	9b	6.13 ± 0.39	5.96 ± 0.03	2.03 ± 0.01	>10
10	DCB-3503 (ref. 36)	—d	—	—	1.83 ± 0.39

a Treated for 16 h.

b Treated for 4 h.

c Treated for 24 h.

d Not applicable.

## 3. Conclusion

Bacterial infections have long been regarded as a formidable enemy of mankind. Bacterial pathogens dramatically affect the normal function of host cells, including immune response, for their own benefit during infection; eventually, they destroy or damage host cells. As reconstitution of gut flora balance and correction of immune defects are two important strategies for IBD treatment, we attempted to discover dual-functional andrographolide analogs for use as antibacterial and immunosuppressant agents that could synergize ultimate efficacy.

In this study, 14-aryloxy andrographolide derivatives were designed, synthesized and tested against the growth of *E. coli*, *S. aureus* and *E. faecalis*. Preliminary data suggest that the andrographolide skeleton is primarily more selective against *E. faecalis*; modifications at the 3- or/and 19-positions are not ignorable; the 14-aryloxy moiety strongly determines essential efficacy against bacteria growth; 14α- and 14β-isomers exhibit different antibacterial activities; and the substitution positions at the phenoxy groups and the structural features of the substitution groups may alter the inhibitory activities of the compounds. The current data imply that nitrogen-containing 14-aryloxy arenes may play an enhanced role in antibacterial activity upon the substitution of phenoxy groups by pyridinyloxy groups and of naphthyloxy groups by quinolinyloxy groups, suggesting the possibility that the nitrogen of the arene group is charged under physiological conditions. Moreover, it was revealed that the 14-(8′-quinolinyloxy) group is an important pharmacophore for antibacterial activity and that 14β-(8′-quinolinyloxy) andrographolide derivatives exhibit superb inhibitory activity toward *E. faecalis*. Importantly, andrographolide analogs with antibacterial activities also possess immunosuppressant activities against the IL-6/STAT3, TLR4/NF-κB and/or TNF-α/NF-κB signaling pathways, enabling us to conclude that dual functional andrographolide analogs can play synergistic roles in IBD treatment.

The results of this study suggest that the development of andrographloide analogs as antibacterial and immunosuppressant agents is possible. Further discovery of more potent structural cores, structural tuning of the 14-aryloxy substitutions and modifications at other positions are underway on the basis of these findings.

## 4. Materials and methods

### 4.1. General information for chemistry

^1^H and ^13^C NMR spectra were recorded on a Bruker AV-400 spectrometer at 400 and 100 MHz, respectively, in CD_3_Cl, CD_3_OD, (CD_3_)_2_SO and C_6_D_6_ as indicated. Coupling constants (*J*) are expressed in hertz (Hz). Chemical shifts (*δ*) of NMR are reported in parts per million (ppm) units relative to the solvent. High resolution ESI-MS spectra were recorded on an Applied Biosystems Q-STAR Elite ESI-LC-MS/MS mass spectrometer. Unless otherwise noted, materials were obtained from commercial suppliers and were used without further purification. Melting points were measured using a YRT-3 melting point apparatus (Shanghai, China) and were uncorrected.

### 4.2. The preparation and characterization of 2, 3a, 3b, 4 and 5a1, 5b1, 6a1 and 6b1 were modified from our previous papers^25,26^

#### 4.2.1. Preparation of compounds 5a and 5b

##### General method for synthesis of 5a and 5b

Under N_2_ atmosphere and at 0 °C, 0.5 g (1.28 mmol) 3a or 3b, assorted phenols (1.92 mmol) and 0.5 g PPh_3_ (1.92 mmol) were dissolved in 8.0 mL anhydrous THF. To the above mixture, a solution of 0.39 g (1.92 mmol) diisopropyl azodiformate (DIAD) in 2.0 mL anhydrous THF was added dropwise in 5 min, and the reaction progress was monitored by TLC. After the reaction was complete, the reaction mixture was treated with ethyl acetate and sol. sat. NaHCO_3_. The organic phase was washed with brine and dried over anhydrous Na_2_SO_4_. The residue was filtered, dried and subjected to silica gel chromatography using petroleum ether and ethyl acetate to yield product 5b or 5a.

##### (14α)-(2′-Carboxy methyl ester-4′-nitro)phenoxy-3,19-acetonylidene andrographolide (5a1)

White solid; mp 77.3 °C to 78.4 °C; 51% yield; ^1^H NMR (400 MHz, C_6_D_6_) *δ* 8.52 (d, *J* = 2.9 Hz, 1H), 7.75 (dd, *J* = 9.1, 2.9 Hz, 1H), 7.27–7.22 (m, 1H), 5.79 (d, *J* = 9.1 Hz, 1H), 4.87 (s, 1H), 4.81 (d, *J* = 5.7 Hz, 1H), 4.61 (s, 1H), 3.79 (d, *J* = 11.6 Hz, 1H), 3.73 (dd, *J* = 10.7, 6.1 Hz, 1H), 3.66 (dd, *J* = 10.7, 2.6 Hz, 1H), 3.45 (dd, *J* = 7.4, 3.3 Hz, 1H), 3.39 (s, 3H), 3.06 (d, *J* = 11.6 Hz, 1H), 2.51 (m, 1H), 2.27–2.16 (m, 2H), 1.89–1.79 (m, 1H), 1.79–1.69 (m, 1H), 1.62–1.47 (m, 3H), 1.39 (s, 3H), 1.31 (s, 3H), 1.09 (s, 3H), 1.05–0.96 (m, 2H), 0.94 (dd, *J* = 6.4, 5.0 Hz, 1H), 0.89 (s, 3H), 0.86–0.82 (m, 1H); ^13^C NMR (101 MHz, (CD_3_)_2_SO) *δ* 169.2, 164.5, 160.7, 151.6, 148.1, 141.3, 129.3, 127.2, 124.8, 122.1, 116.0, 109.0, 98.6, 76.2, 73.4, 70.9, 63.2, 55.3, 53.0, 51.8, 38.3, 37.6, 37.4, 34.4, 27.9, 26.2, 25.6, 25.2, 25.1, 23.0, 16.0; ESI-HRMS: *m*/*z* 592.2525 [M + Na]^+^, calcd for C_31_H_39_NNaO_9_, 592.2523.

##### (14β)-(2′-Carboxy methyl ester-4′-nitro)phenoxy-3,19-acetonylidene andrographolide (5b1)

White solid; mp 104 °C to 106 °C; 82% yield; ^1^H NMR (400 MHz, C_6_D_6_) *δ* 8.57 (d, *J* = 2.9 Hz, 1H), 7.74 (dd, *J* = 9.1, 2.9 Hz, 1H), 7.25 (t, *J* = 6.2 Hz, 1H), 5.71 (d, *J* = 9.1 Hz, 1H), 4.86 (s, 1H), 4.80 (s, 1H), 4.42 (s, 1H), 3.78 (d, *J* = 11.5 Hz, 1H), 3.62 (m, 2H), 3.43 (dd, *J* = 7.1, 3.2 Hz, 1H), 3.35 (s, 3H), 3.06 (d, *J* = 11.5 Hz, 1H), 2.41 (dd, *J* = 16.5, 5.5 Hz, 1H), 2.26–2.14 (m, 2H), 1.78–1.67 (m, 2H), 1.59 (d, *J* = 10.8 Hz, 1H), 1.53–1.43 (m, 2H), 1.38 (s, 3H), 1.33 (s, 3H), 1.08 (s, 3H), 1.00 (m, 2H), 0.94 (d, *J* = 2.1 Hz, 1H), 0.91 (s, 3H), 0.87–0.83 (m, 1H); ^13^C NMR (101 MHz, C_6_D_6_) *δ* 167.90, 163.40, 160.05, 151.64, 148.46, 141.78, 128.31, 127.80, 124.20, 122.34, 114.01, 107.81, 99.48, 74.86, 73.23, 69.52, 64.21, 55.71, 51.99, 50.77, 38.37, 38.25, 37.61, 33.40, 26.17, 26.10, 25.89, 25.06, 24.41, 23.20, 16.70; ESI-HRMS: *m*/*z* 592.2523 [M + Na]^+^, calcd for C_31_H_39_NNaO_9_, 592.2515.

##### (14α)-(2′-Fluoro-4′-nitro)phenoxy-3,19-acetonylidene andrographolide (5a2)

White solid; mp 147 °C to 149 °C; 47% yield; ^1^H NMR (400 MHz, (CD_3_)_2_SO) *δ* 8.26 (dd, *J* = 10.9, 2.7 Hz, 1H), 8.16 (m, 1H), 7.48 (t, *J* = 8.8 Hz, 1H), 6.97 (t, *J* = 6.3 Hz, 1H), 6.07 (d, *J* = 5.2 Hz, 1H), 4.83 (s, 1H), 4.74 (dd, *J* = 11.1, 5.5 Hz, 1H), 4.59 (s, 1H), 4.45 (dd, *J* = 11.1, 1.0 Hz, 1H), 3.82 (d, *J* = 11.6 Hz, 1H), 3.36 (dd, *J* = 9.2, 4.2 Hz, 1H), 3.07 (d, *J* = 11.6 Hz, 1H), 2.47–2.36 (m, 2H), 2.33 (dd, *J* = 10.3, 2.8 Hz, 1H), 2.03–1.91 (m, 2H), 1.89–1.77 (m, 1H), 1.69–1.55 (m, 2H), 1.54–1.45 (m, 1H), 1.28 (s, 3H), 1.25–1.20 (m, 2H), 1.23 (s, 3H), 1.10 (s, 3H), 0.86–0.80 (m, 1H), 0.76 (s, 3H); ^13^C NMR (101 MHz, (CD_3_)_2_SO) *δ* 168.64, 152.23, 151.34, 150.40, 150.30, 149.76, 147.60, 141.15, 141.07, 124.19, 121.28, 121.25, 115.75, 112.68, 112.45, 108.54, 98.15, 75.65, 73.01, 70.35, 62.74, 54.93, 51.35, 37.85, 37.14, 36.96, 33.83, 27.35, 25.73, 25.13, 24.96, 24.69, 22.59, 15.59; ESI-HRMS: *m*/*z* 552.2374 [M + Na]^+^, calcd for C_29_H_36_FNNaO_7_, 552.2368.

##### (14β)-(2′-Fluoro-4′-nitro)phenoxy-3,19-acetonylidene andrographolide (5b2)

White solid; mp 114 °C to 117 °C; 72% yield; ^1^H NMR (400 MHz, C_6_D_6_) *δ* 7.57 (dd, *J* = 10.6, 2.6 Hz, 1H), 7.47 (m, 1H), 7.24–7.19 (m, 1H), 5.76 (m, 1H), 4.84 (d, *J* = 1.8 Hz, 1H), 4.71 (d, *J* = 5.1 Hz, 1H), 4.40 (d, *J* = 1.7 Hz, 1H), 3.78 (d, *J* = 11.6 Hz, 1H), 3.64–3.51 (m, 2H), 3.44 (dd, *J* = 7.0, 3.5 Hz, 1H), 3.06 (d, *J* = 11.5 Hz, 1H), 2.27–2.09 (m, 3H), 1.80–1.61 (m, 2H), 1.56–1.44 (m, 2H), 1.40 (s, 3H), 1.34 (s, 3H), 1.27–1.35 (m, 2H), 1.06 (s, 3H), 0.97 (td, *J* = 14.7, 13.8, 5.4 Hz, 2H), 0.89 (s, 3H), 0.87–0.90 (m, 1H); ^13^C NMR (101 MHz, C_6_D_6_) *δ* 167.99, 167.81, 153.15, 151.64, 151.49, 150.64, 149.80, 149.70, 148.26, 142.41, 142.34, 124.26, 120.68, 115.14, 113.08, 112.85, 107.85, 99.62, 74.78, 73.52, 69.61, 64.32, 55.55, 50.74, 38.40, 38.35, 37.62, 33.33, 26.12, 25.93, 25.12, 24.35, 23.24, 16.78; ESI-HRMS: *m*/*z* 552.2374 [M + Na]^+^, calcd for C_29_H_36_FNNaO_7_, 552.2418.

##### (14α)-(2′-Methoxy-4′-nitro)phenoxy-3,19-acetonylidene andrographolide (5a3)^25,26^

Pale yellow solid; mp 121 °C to 123 °C; 46% yield; ^1^HNMR (400 MHz, C_6_D_6_) *δ* 7.56 (dd, *J* = 8.8, 2.4 Hz, 1H), 7.45 (d, *J* = 2.5 Hz, 1H), 7.14–7.10 (m, 1H), 6.07–5.99 (m, 1H), 4.90 (s, 1H), 4.82 (s, 1H), 4.61 (s, 1H), 3.85–3.76 (m, 2H), 3.71 (dd, *J* = 10.8, 5.9 Hz, 1H), 3.46 (dd, *J* = 7.5, 3.7 Hz, 1H), 3.11–3.03 (m, 4H), 2.41–2.24 (m, 1H), 2.22–2.05 (m, 2H), 1.85 (m, 1H), 1.79–1.67 (m, 1H), 1.62–1.52 (m, 1H), 1.62–1.52 (m, 1H), 1.52–1.46 (m, 1H), 1.41 (s, 3H), 1.36 (s, 3H), 1.32 (m, 1H), 1.08 (s, 3H), 1.04–0.89 (m, 3H), 0.81 (s, 3H); ^13^C NMR (101 MHz, C_6_D_6_) *δ* 168.17, 151.11, 150.60, 147.17, 143.51, 124.80, 116.98, 115.61, 109.33, 107.40, 99.44, 75.17, 73.37, 69.90, 64.12, 55.82, 55.21, 51.25, 38.16, 38.04, 37.64, 34.06, 26.32, 26.01, 25.32, 25.20, 24.69, 23.16, 16.39; ESI-HRMS: *m*/*z* 564.2569 [M + Na]^+^, calcd for C_30_H_39_NNaO_8_, 564.2573.

##### (14β)-(2′-Methoxy-4′-nitro)phenoxy-3,19-acetonylidene andrographolide (5b3)^25,26^

Pale yellow solid; mp 129 °C to 131 °C; 51% yield; ^1^H NMR (400 MHz, C_6_D_6_) *δ* 7.56 (dd, *J* = 8.8, 2.6 Hz, 1H), 7.46 (d, *J* = 2.6 Hz, 1H), 7.18 (d, *J* = 1.5 Hz, 1H), 5.95 (d, *J* = 8.8 Hz, 1H), 4.90–4.86 (m, 1H), 4.85–4.80 (m, 1H), 4.39 (dd, *J* = 1.9, 1.0 Hz, 1H), 3.82–3.74 (m, 2H), 3.63–3.57 (m, 2H), 3.41 (dd, *J* = 7.3, 3.6 Hz, 1H), 3.10–3.02 (m, 1H), 3.06 (s, 3H), 2.19–2.10 (m, 3H), 1.77–1.60 (m, 2H), 1.52–1.44 (m, 1H), 1.44–1.37 (m, 1H), 1.40 (s, 3H), 1.36 (s, 3H), 1.34–1.25 (m, 2H), 1.06 (s, 3H), 0.99 (m, 1H), 0.94–0.80 (m, 3H), 0.85 (s, 3H); ^13^C NMR (101 MHz, CDCl_3_) *δ* 168.12, 150.83, 150.47, 150.31, 148.06, 143.21, 128.02, 127.78, 127.54, 124.91, 116.92, 114.96, 107.68, 107.11, 99.35, 74.78, 72.86, 69.71, 64.04, 55.41, 55.12, 50.72, 38.16, 38.06, 37.43, 33.20, 26.08, 25.82, 25.55, 24.99, 24.32, 23.04, 16.44; ESI-HRMS: *m*/*z* 564.2584 [M + Na]^+^, calcd for C_30_H_39_NNaO_8_, 564.2573.

##### (14α)-(3′-Fluoro-4′-nitro)phenoxy-3,19-acetonylidene andrographolide (5a4)

White solid; mp 137 °C to 139 °C; 51% yield; ^1^H NMR (400 MHz, C_6_D_6_) *δ* 7.46 (m, 1H), 6.34–6.26 (m, 1H), 6.05 (t, *J* = 7.9 Hz, 2H), 5.42 (s, 1H), 5.12 (m, 2H), 3.86 (d, *J* = 11.5 Hz, 1H), 3.69 (m, 2H), 3.50 (dd, *J* = 7.0, 3.2 Hz, 1H), 3.09 (d, *J* = 11.5 Hz, 1H), 2.32–2.22 (m, 2H), 2.03–1.91 (m, 2H), 1.86–1.64 (m, 4H), 1.43 (s, 3H), 1.41 (s, 3H), 1.50–1.32 (m, 2H), 1.11–1.06 (m, 2H), 1.04 (s, 3H), 1.01 (s, 3H); ^13^C NMR (101 MHz, C_6_D_6_) *δ* 171.37, 163.02, 162.91, 158.53, 155.89, 147.16, 145.92, 133.57, 131.56, 131.49, 128.03, 127.79, 110.42, 110.39, 109.14, 104.87, 104.63, 99.58, 74.86, 73.48, 69.98, 64.29, 52.30, 51.40, 38.39, 38.28, 38.16, 33.88, 30.73, 26.12, 25.86, 25.14, 24.56, 23.45, 16.99; ESI-HRMS: *m*/*z* 552.2365 [M + Na]^+^, calcd for C_29_H_36_FNNaO_7_, 552.2374.

##### (14β)-(3′-Fluoro-4′-nitro)phenoxy-3,19-acetonylidene andrographolide (5b4)

White solid; mp 184 °C to 187 °C; 52% yield; ^1^H NMR (400 MHz, C_6_D_6_) *δ* 7.51 (t, *J* = 8.9 Hz, 1H), 7.20 (d, *J* = 1.6 Hz, 1H), 5.89–5.77 (m, 2H), 4.86 (d, *J* = 1.1 Hz, 1H), 4.54–4.48 (m, 1H), 4.41 (d, *J* = 0.7 Hz, 1H), 3.76 (d, *J* = 11.5 Hz, 1H), 3.50 (d, *J* = 3.8 Hz, 2H), 3.43 (dd, *J* = 6.8, 3.5 Hz, 1H), 3.06 (d, *J* = 11.5 Hz, 1H), 2.23–2.06 (m, 3H), 1.76–1.63 (m, 2H), 1.48–1.35 (m, 2H), 1.38 (s, 3H), 1.33 (s, 3H), 1.34–1.27 (m, 2H), 1.05 (d, *J* = 4.6 Hz, 3H), 1.04–0.95 (m, 1H), 0.95–0.87 (m, 2H), 0.89 (s, 3H); ^13^C NMR (101 MHz, C_6_D_6_) *δ* 167.85, 167.84, 161.50, 161.50, 161.40, 161.40, 158.59, 158.59, 155.95, 155.95, 151.01, 151.01, 148.11, 148.11, 132.01, 131.94, 131.94, 124.20, 124.20, 111.06, 111.06, 111.03, 111.03, 107.94, 107.94, 104.20, 104.20, 103.96, 103.96, 99.72, 99.72, 74.57, 74.57, 72.19, 72.19, 69.41, 69.41, 64.33, 64.33, 55.60, 55.60, 50.79, 50.79, 38.40, 38.40, 37.67, 37.67, 33.47, 33.47, 25.97, 25.97, 25.93, 25.93, 25.07, 25.07, 24.25, 24.25, 23.21, 23.21, 16.82, 16.82; ESI-HRMS: *m*/*z* 552.2415 [M + Na]^+^, calcd for C_29_H_36_FNNaO_7_, 552.2374.

##### (14α)-(3′-Methoxy-4′-nitro)phenoxy-3,19-acetonylidene andrographolide (5a5)

White solid; mp 127 °C to 129 °C; 41% yield; ^1^H NMR (400 MHz, (CD_3_)_2_SO) *δ* 8.00 (d, *J* = 9.1 Hz, 1H), 6.98 (dd, *J* = 10.0, 3.9 Hz, 1H), 6.86 (d, *J* = 2.4 Hz, 1H), 6.74 (dd, *J* = 9.1, 2.5 Hz, 1H), 6.03 (d, *J* = 5.2 Hz, 1H), 4.87 (s, 1H), 4.71 (dd, *J* = 11.0, 5.5 Hz, 1H), 4.61 (s, 1H), 4.36 (dd, *J* = 11.0, 0.9 Hz, 1H), 3.93 (s, 3H), 3.83 (d, *J* = 11.7 Hz, 1H), 3.39–3.35 (m, 1H), 3.08 (d, *J* = 11.6 Hz, 1H), 2.47–2.30 (m, 3H), 2.02–1.92 (m, 2H), 1.88–1.78 (m, 1H), 1.69–1.57 (m, 2H), 1.53–1.44 (m, 1H), 1.29 (s, 3H), 1.23 (s, 3H), 1.21–1.25 (m, 2H), 1.21–1.16 (m, 1H), 1.10 (s, 3H), 0.77 (s, 3H); ^13^C NMR (101 MHz, (CD_3_)_2_SO) *δ* 168.84, 161.72, 154.94, 150.46, 147.60, 133.08, 127.88, 124.70, 108.46, 106.86, 101.33, 98.16, 75.67, 71.68, 70.69, 62.73, 56.88, 54.95, 51.35, 37.92, 37.14, 36.96, 33.87, 27.38, 25.73, 25.14, 24.97, 24.72, 22.58, 15.60; ESI-HRMS: *m*/*z* 564.2569 [M + Na]^+^, calcd for C_30_H_39_NNaO_8_, 564.2573.

##### (14β)-(3′-Methoxy-4′-nitro)phenoxy-3,19-acetonylidene andrographolide (5b5)

White solid; mp 169 °C to 171 °C; 75% yield; ^1^H NMR (400 MHz, C_6_D_6_) *δ* 7.60 (d, *J* = 9.0 Hz, 1H), 7.22 (m, 1H), 5.98 (d, *J* = 2.4 Hz, 1H), 5.54 (dd, *J* = 9.0, 2.5 Hz, 1H), 4.87 (d, *J* = 0.9 Hz, 1H), 4.76 (d, *J* = 5.4 Hz, 1H), 4.45 (s, 1H), 3.80–3.69 (m, 2H), 3.63 (m, 1H), 3.41 (m, 1H), 3.09 (s, 3H), 3.06 (m, 1H), 2.30–2.13 (m, 3H), 1.70 (m, 2H), 1.52 (t, *J* = 8.6 Hz, 1H), 1.45–1.36 (m, 1H), 1.38 (s, 3H), 1.35–1.28 (m, 2H), 1.32 (s, 3H), 1.06 (s, 3H), 1.04–0.96 (m, 1H), 0.95–0.90 (m, 1H), 0.98–0.82 (m, 1H), 0.87 (s, 3H); ^13^C NMR (101 MHz, C_6_D_6_) *δ* 168.23, 160.90, 155.43, 150.53, 148.07, 134.84, 127.80, 124.84, 107.95, 104.43, 101.58, 99.50, 74.86, 71.75, 69.87, 64.15, 55.72, 55.64, 51.01, 38.39, 38.21, 37.65, 33.57, 26.20, 25.92, 25.80, 25.08, 24.45, 23.16, 16.60; ESI-HRMS: *m*/*z* 564.2569 [M + Na]^+^, calcd for C_30_H_39_NNaO_8_, 564.2573.

##### (14β)-(2′-Nitro)phenoxy-3,19-acetonylidene andrographolide (5b6)

White solid; mp 146 °C to 148 °C; 72% yield; ^1^H NMR (400 MHz, C_6_D_6_) *δ* 7.32–7.25 (m, 1H), 7.24–7.18 (m, 2H), 6.77–6.65 (m, 1H), 6.42–6.30 (m, 1H), 6.06–5.92 (m, 1H), 4.93–4.80 (m, 2H), 4.42–4.35 (m, 1H), 3.82 (d, *J* = 11.5 Hz, 1H), 3.71–3.55 (m, 2H), 3.45 (dd, *J* = 7.7, 3.3 Hz, 1H), 3.08 (d, *J* = 11.5 Hz, 1H), 2.40–2.29 (m, 1H), 2.26–2.11 (m, 2H), 1.86–1.67 (m, 2H), 1.62–1.47 (m, 3H), 1.42 (s, 3H), 1.36 (s, 3H), 1.39–1.31 (m, 1H), 1.12 (s, 3H), 1.05–0.91 (m, 3H), 0.87 (s, 3H); ^13^C NMR (101 MHz, C_6_D_6_) *δ* 168.02, 168.02, 161.21, 151.64, 149.29, 149.29, 148.41, 141.55, 141.55, 133.24, 133.24, 128.28, 128.22, 128.16, 128.04, 127.92, 127.80, 127.68, 125.77, 124.31, 121.71, 121.71, 115.78, 107.77, 99.27, 99.26, 99.26, 75.38, 73.27, 69.64, 64.11, 59.13, 55.72, 51.04, 38.39, 38.11, 37.63, 37.57, 33.61, 26.66, 25.93, 25.22, 24.72, 23.19, 16.49; ESI-HRMS: *m*/*z* 534.2467 [M + Na]^+^, calcd for C_29_H_37_NNaO_7_, 534.2468.

##### (14β)-(3′-Nitro)phenoxy-3,19-acetonylidene andrographolide (5b7)

White solid; mp 163 °C to 165 °C; 32% yield; ^1^H NMR (400 MHz, C_6_D_6_) *δ* 7.51 (d, *J* = 7.1 Hz, 1H), 7.31 (d, *J* = 2.1 Hz, 1H), 7.20 (d, *J* = 6.8 Hz, 2H), 6.62 (t, *J* = 8.2 Hz, 1H), 6.52 (dd, *J* = 8.3, 1.8 Hz, 1H), 4.85 (s, 1H), 4.62 (d, *J* = 5.1 Hz, 1H), 4.43 (s, 1H), 3.77 (d, *J* = 11.6 Hz, 1H), 3.64 (dd, *J* = 10.8, 1.8 Hz, 1H), 3.58 (dd, *J* = 10.8, 5.5 Hz, 1H), 3.41 (dd, *J* = 7.1, 3.5 Hz, 1H), 3.06 (d, *J* = 11.5 Hz, 1H), 2.24–2.05 (m, 3H), 1.68 (m, 2H), 1.52–1.41 (m, 2H), 1.39 (s, 3H), 1.34 (s, 3H), 1.30 (m, 2H), 1.07 (s, 3H), 1.04–0.94 (m, 1H), 0.94–0.89 (m, 1H), 0.87 (d, *J* = 5.9 Hz, 1H), 0.84 (s, 3H); ^13^C NMR (101 MHz, C_6_D_6_) *δ* 168.13, 157.06, 150.18, 149.49, 147.85, 130.36, 128.16, 127.92, 127.80, 127.68, 124.91, 122.22, 116.85, 109.17, 107.97, 99.46, 74.86, 71.83, 69.76, 64.15, 55.58, 50.96, 38.36, 38.22, 37.61, 33.51, 26.20, 25.90, 25.79, 25.07, 24.38, 23.14, 16.58; ESI-HRMS: *m*/*z* 534.2463 [M + Na]^+^, calcd for C_29_H_37_NNaO_7_, 534.2468.

##### (14α)-(2′-Carboxy ethyl ester)phenoxy-3,19-acetonylidene andrographolide (5a8)

White solid; mp 122 °C to 124 °C; 60% yield; ^1^H NMR (400 MHz, C_6_D_6_) *δ* 7.78 (dd, *J* = 7.7, 1.8 Hz, 1H), 7.21 (t, *J* = 6.0 Hz, 1H), 6.98–6.92 (m, 1H), 6.71 (t, *J* = 7.6 Hz, 1H), 6.38 (d, *J* = 8.2 Hz, 1H), 5.11 (s, 1H), 4.84 (s, 1H), 4.57 (s, 1H), 4.19–4.11 (m, 2H), 4.10–4.06 (m, 1H), 3.83–3.75 (m, 2H), 3.44 (dd, *J* = 7.9, 3.7 Hz, 1H), 3.07 (d, *J* = 11.5 Hz, 1H), 2.22–2.13 (m, 3H), 1.84 (m, 1H), 1.78–1.67 (m, 1H), 1.60–1.51 (m, 1H), 1.51–1.43 (m, 2H), 1.42 (s, 3H), 1.35 (s, 3H), 1.31 (d, *J* = 2.5 Hz, 1H), 1.09 (s, 3H), 1.06 (t, *J* = 7.1 Hz, 3H), 0.99–0.88 (m, 3H), 0.80 (s, 3H); ^13^C NMR (101 MHz, C_6_D_6_) *δ* 168.70, 165.60, 156.04, 149.51, 147.00, 132.85, 132.04, 125.51, 124.38, 122.50, 117.83, 109.13, 99.26, 75.48, 73.78, 70.37, 64.02, 60.96, 55.62, 51.54, 38.23, 38.07, 37.66, 34.21, 26.70, 26.05, 25.29, 25.24, 24.89, 23.15, 16.22, 14.10; ESI-HRMS: *m*/*z* 561.2824 [M + Na]^+^ calcd for C_32_H_42_NaO_7_, 561.2828.

##### (14β)-(2′-Carboxy ethyl ester)phenoxy-3,19-acetonylidene andrographolide (5b8)

White solid; mp 168 °C to 170 °C; 78% yield; ^1^H NMR (400 MHz, C_6_D_6_) *δ* 7.80 (dd, *J* = 7.7, 1.8 Hz, 1H), 7.24–7.18 (m, 1H), 6.98–6.92 (m, 1H), 6.69 (m, 1H), 6.28 (d, *J* = 8.2 Hz, 1H), 5.04 (d, *J* = 5.4 Hz, 1H), 4.84 (d, *J* = 1.1 Hz, 1H), 4.44 (s, 1H), 4.19–4.04 (m, 2H), 4.01 (dd, *J* = 10.6, 1.4 Hz, 1H), 3.82 (d, *J* = 11.5 Hz, 1H), 3.68 (dd, *J* = 10.6, 5.5 Hz, 1H), 3.42 (dd, *J* = 7.6, 3.7 Hz, 1H), 3.08 (d, *J* = 11.5 Hz, 1H), 2.24–2.05 (m, 3H), 1.80–1.65 (m, 2H), 1.59 (d, *J* = 8.3 Hz, 1H), 1.53–1.43 (m, 1H), 1.41 (s, 3H), 1.40–1.31 (m, 2H), 1.37 (s, 3H), 1.11 (s, 3H), 1.04 (t, 3H), 1.00–0.88 (m, 3H), 0.85 (s, 3H); ^13^C NMR (101 MHz, C_6_D_6_) *δ* 168.70, 165.65, 155.99, 149.74, 148.21, 132.91, 132.15, 128.16, 127.92, 127.80, 127.68, 125.81, 123.92, 122.16, 116.61, 107.89, 99.29, 75.26, 73.23, 70.29, 64.14, 60.97, 55.83, 50.97, 38.39, 38.15, 37.64, 33.49, 26.55, 25.91, 25.87, 25.20, 24.64, 23.22, 16.51, 14.12; ESI-HRMS: *m*/*z* 561.2829 [M + Na]^+^, calcd for C_32_H_42_NaO_7_, 561.2828.

##### (14α)-(4′-Carboxy ethyl ester)phenoxy-3,19-acetonylidene andrographolide (5a9)

White solid; mp 67.8 °C to 73.7 °C; 30% yield; ^1^H NMR (400 MHz, C_6_D_6_) *δ* 8.15–8.08 (m, 2H), 6.49 (d, *J* = 8.7 Hz, 2H), 4.85 (s, 2H), 4.65 (s, 1H), 4.16 (q, *J* = 7.1 Hz, 2H), 3.82–3.72 (m, 3H), 3.44 (dd, *J* = 7.8, 3.8 Hz, 1H), 3.06 (d, *J* = 11.6 Hz, 1H), 2.39–2.28 (m, 1H), 2.21–2.12 (m, 2H), 1.86–1.69 (m, 2H), 1.54 (t, *J* = 10.1 Hz, 2H), 1.43–1.38 (m, 1H), 1.42 (s, 3H), 1.34 (s, 3H), 1.33–1.29 (m, 1H), 1.09 (s, 3H), 1.05 (t, *J* = 7.1 Hz, 3H), 1.00–0.93 (m, 2H), 0.85 (t, *J* = 6.1 Hz, 2H), 0.75 (s, 3H); ^13^C NMR (101 MHz, (CD_3_)_2_SO) *δ* 169.4, 165.7, 161.0, 150.8, 148.1, 131.9, 125.3, 123.7, 115.8, 109.0, 98.6, 76.1, 71.8, 71.3, 63.2, 60.9, 55.4, 51.8, 38.3, 37.6, 37.4, 34.3, 27.8, 26.2, 25.6, 25.4, 25.2, 23.1, 16.1, 14.7; ESI-HRMS: *m*/*z* 561.2822 [M + Na]^+^, calcd for C_32_H_42_NaO_7_, 561.2828.

##### (14β)-(4′-Carboxy ethyl ester)phenoxy-3,19-acetonylidene andrographolide (5b9)

White solid; mp 122 °C to 125 °C; 45% yield; ^1^H NMR (400 MHz, C_6_D_6_) *δ* 8.14–8.09 (m, 2H), 7.23–7.18 (m, 1H), 6.45 (d, *J* = 8.8 Hz, 2H), 4.86–4.78 (m, 2H), 4.44 (s, 1H), 4.16 (q, *J* = 7.1 Hz, 2H), 3.80–3.71 (m, 2H), 3.68 (t, *J* = 7.2 Hz, 1H), 3.38 (dd, *J* = 7.4, 3.6 Hz, 1H), 3.05 (d, *J* = 11.5 Hz, 1H), 2.16 (m, 3H), 1.75–1.63 (m, 2H), 1.52 (d, *J* = 10.2 Hz, 1H), 1.46–1.36 (m, 1H), 1.37 (s, 3H), 1.35–1.26 (m, 2H), 1.33 (s, 3H), 1.06 (s, 3H), 1.07–1.01 (m, 3H), 1.01–0.83 (m, 3H), 0.81 (s, 3H); ^13^C NMR (101 MHz, C_6_D_6_) *δ* 168.29, 165.52, 160.29, 149.91, 147.98, 132.07 (2C), 125.26, 124.92, 115.12 (2C), 107.85, 75.00, 71.48, 70.01, 64.14, 60.70, 55.66, 51.07, 38.37, 38.18, 37.64, 33.49, 26.32, 25.91, 25.68, 25.11, 24.47, 23.17, 16.49, 14.21; ESI-HRMS: *m*/*z* 561.2825 [M + Na]^+^, calcd for C_32_H_42_NaO_7_, 561.2828.

##### (14β)-(4′-Cyano)phenoxy-3,19-acetonylidene andrographolide (5b10)

White solid; mp 177 °C to 179 °C; 42% yield; ^1^H NMR (400 MHz, C_6_D_6_) *δ* 7.22–7.18 (m, 1H), 6.97–6.91 (m, 2H), 6.12–6.07 (m, 2H), 4.85 (d, *J* = 1.1 Hz, 1H), 4.66 (s, 1H), 4.42 (s, 1H), 3.77 (d, *J* = 11.5 Hz, 1H), 3.63–3.56 (m, 2H), 3.43 (dd, *J* = 7.1, 3.6 Hz, 1H), 3.06 (d, *J* = 11.5 Hz, 1H), 2.23–2.04 (m, 3H), 1.74–1.64 (m, 2H), 1.51–1.38 (m, 2H), 1.41 (s, 3H), 1.35 (s, 3H), 1.34–1.24 (m, 2H), 1.07 (s, 1H), 0.98 (m, 1H), 0.97 (m, 1H), 0.92–0.84 (m, 2H), 0.83 (s, 3H); ^13^C NMR (101 MHz, C_6_D_6_) *δ* 168.06, 159.36, 150.35, 148.00, 134.09, 124.74, 118.44, 115.68, 107.84, 106.04, 99.53, 74.78, 71.45, 69.64, 64.18, 55.54, 50.88, 38.31, 38.25, 37.60, 33.44, 26.12, 25.91, 25.72, 25.08, 24.33, 23.13, 16.59; ESI-HRMS: *m*/*z* 514.2566 [M + Na]^+^ calcd for C_30_H_37_NNaO_5_, 514.2569.

##### (14β)-(4′-Methoxy)phenoxy-3,19-acetonylidene andrographolide (5b11)

White solid; mp 149 °C to 151 °C; 48% yield; ^1^H NMR (400 MHz, C_6_D_6_) *δ* 7.20–7.17 (m, 1H), 6.69–6.62 (m, 2H), 6.57–6.51 (m, 2H), 4.86–4.78 (m, 2H), 4.43 (q, *J* = 1.4 Hz, 1H), 4.01 (dd, *J* = 10.5, 1.8 Hz, 1H), 3.82 (d, *J* = 11.5 Hz, 1H), 3.75–3.68 (m, 1H), 3.41 (m, 1H), 3.30 (s, 3H), 3.08 (d, *J* = 11.5 Hz, 1H), 2.21–2.05 (m, 3H), 1.82–1.64 (m, 2H), 1.55–1.43 (m, 2H), 1.41 (s, 3H), 1.38–1.29 (m, 2H), 1.37 (s, 3H), 1.08 (s, 3H), 1.02–0.90 (m, 2H), 0.89–0.79 (m, 1H), 0.83 (s, 3H); ^13^C NMR (101 MHz, C_6_D_6_) *δ* 168.82, 155.47, 150.74, 149.23, 148.08, 126.05, 117.95, 117.95, 115.12, 115.12, 107.93, 99.28, 75.34, 72.90, 70.43, 64.10, 55.63, 55.06, 51.21, 38.34, 38.11, 37.64, 33.66, 26.57, 25.98, 25.63, 25.24, 24.72, 23.18, 16.43; ESI-HRMS: *m*/*z* 519.2725 [M + Na]^+^, calcd for C_30_H_40_NaO_6_, 519.2723.

##### (14α)-(2′-Methoxy)phenoxy-3,19-acetonylidene andrographolide (5a12)

White solid; mp 48.7 °C to 52.6 °C; 57% yield; ^1^H NMR (400 MHz, C_6_D_6_) *δ* 6.81 (ddd, *J* = 8.1, 6.9, 2.3 Hz, 1H), 6.69–6.61 (m, 2H), 6.48 (d, *J* = 8.2 Hz, 1H), 5.15 (d, *J* = 5.2 Hz, 1H), 4.82 (s, 1H), 4.62 (s, 1H), 4.21 (dd, *J* = 10.6, 1.6 Hz, 1H), 3.82 (d, *J* = 11.5 Hz, 1H), 3.75 (dd, *J* = 10.6, 5.6 Hz, 1H), 3.46 (dd, *J* = 7.7, 3.7 Hz, 1H), 3.28 (s, 3H), 3.08 (d, *J* = 11.5 Hz, 1H), 2.25–2.12 (m, 3H), 1.87 (m, 1H), 1.77–1.66 (m, 1H), 1.61–1.52 (m, 1H), 1.50–1.41 (m, 2H), 1.43 (s, 3H), 1.39 (s, 3H), 1.36–1.27 (m, 2H), 1.09 (s, 3H), 1.02–0.87 (m, 4H), 0.82 (s, 3H); ^13^C NMR (101 MHz, (CD_3_)_2_SO) *δ* 169.8, 151.3, 149.8, 147.9, 145.9, 126.1, 124.1, 121.2, 119.2, 113.1, 109.1, 98.6, 76.2, 73.3, 71.7, 63.2, 56.0, 55.3, 51.8, 38.4, 37.6, 37.5, 34.4, 27.9, 26.2, 25.7, 25.2, 25.0, 23.1, 16.0; ESI-HRMS: *m*/*z* 519.2719 [M + Na]^+^, calcd for C_30_H_40_NaO_6_, 519.2723.

##### (14β)-(2′-Methoxy)phenoxy-3,19-acetonylidene andrographolide (5b12)

White solid; mp 130 °C to 131 °C; 60% yield; ^1^H NMR (400 MHz, C_6_D_6_) *δ* 7.13–7.10 (m, 1H), 6.80 (m, 1H), 6.65 (m, 1H), 6.58 (dd, *J* = 7.9, 1.6 Hz, 1H), 6.47 (dd, *J* = 8.1, 1.3 Hz, 1H), 5.18 (d, *J* = 5.4 Hz, 1H), 4.82 (d, *J* = 1.2 Hz, 1H), 4.42 (s, 1H), 4.15 (dd, *J* = 10.6, 1.5 Hz, 1H), 3.83 (d, *J* = 11.5 Hz, 1H), 3.65 (dd, *J* = 10.6, 5.5 Hz, 1H), 3.41 (dd, *J* = 7.7, 3.7 Hz, 1H), 3.29 (s, 3H), 3.09 (d, *J* = 11.5 Hz, 1H), 2.26–2.04 (m, 3H), 1.83–1.62 (m, 2H), 1.55–1.44 (m, 2H), 1.42 (s, 3H), 1.39 (s, 3H), 1.38–1.28 (m, 2H), 1.08 (s, 3H), 1.05–0.94 (m, 1H), 0.94–0.88 (m, 1H), 0.88–0.83 (m, 1H), 0.85 (s, 3H); ^13^C NMR (101 MHz, C_6_D_6_) *δ* 169.08, 151.78, 149.42, 148.27, 145.92, 128.15, 128.03, 127.91, 127.79, 127.67, 126.39, 124.00, 121.11, 120.16, 112.54, 107.85, 99.30, 75.39, 73.44, 70.56, 64.14, 55.72, 55.11, 51.10, 38.34, 38.12, 37.66, 33.56, 26.57, 26.00, 25.64, 25.29, 24.74, 23.24, 16.46; ESI-HRMS: *m*/*z* 519.2715 [M + Na]^+^, calcd for C_30_H_40_NaO_6_, 519.2723.

##### (14β)-(3′-Methoxy)phenoxy-3,19-acetonylidene andrographolide (2b13)

White solid; mp 168 °C to 170 °C; 58% yield; ^1^H NMR (400 MHz, C_6_D_6_) *δ* 7.25–7.19 (m, 1H), 6.97 (t, *J* = 8.2 Hz, 1H), 6.43 (dd, *J* = 8.2, 2.0 Hz, 1H), 6.38 (t, *J* = 2.2 Hz, 1H), 6.19 (d, *J* = 8.2 Hz, 1H), 4.90 (s, 1H), 4.84 (d, *J* = 0.9 Hz, 1H), 4.46 (s, 1H), 3.93 (d, *J* = 10.6 Hz, 1H), 3.81 (d, *J* = 11.5 Hz, 1H), 3.76–3.68 (m, 1H), 3.40 (dd, *J* = 7.7, 3.7 Hz, 1H), 3.30 (s, 3H), 3.07 (d, *J* = 11.5 Hz, 1H), 2.34–2.25 (m, 1H), 2.15 (m, 2H), 1.79–1.65 (m, 2H), 1.58 (d, *J* = 10.4 Hz, 1H), 1.51–1.42 (m, 1H), 1.41 (s, 3H), 1.40–1.37 (m, 1H), 1.37–1.30 (m, 2H), 1.36 (s, 3H), 1.09 (s, 3H), 1.02–0.92 (m, 2H), 0.88 (dd, *J* = 12.9, 2.1 Hz, 1H), 0.81 (s, 3H); ^13^C NMR (101 MHz, C_6_D_6_) *δ* 168.61, 161.60, 158.18, 149.42, 147.97, 130.45, 125.84, 107.90, 107.58, 107.42, 102.64, 99.25, 75.36, 71.37, 70.39, 64.06, 55.70, 54.79, 51.24, 38.40, 38.10, 37.66, 33.61, 26.66, 25.95, 25.59, 25.20, 24.72, 23.16, 16.40; ESI-HRMS: *m*/*z* 519.2717 [M + Na]^+^ calcd for C_30_H_40_NaO_6_, 519.2723.

##### (14β)-(Naphthyl-1′-oxy)-3,19-acetonylidene andrographolide (5b14)

White solid; mp 158 °C to 161 °C; 46% yield; ^1^H NMR (400 MHz, C_6_D_6_) *δ* 8.16 (dd, *J* = 6.2, 3.5 Hz, 1H), 7.59–7.54 (m, 1H), 7.39 (dd, *J* = 11.0, 4.2 Hz, 1H), 7.30 (d, *J* = 8.3 Hz, 1H), 7.26–7.21 (m, 2H), 7.11–7.06 (m, 1H), 6.02 (d, *J* = 7.6 Hz, 1H), 4.96 (d, *J* = 5.6 Hz, 1H), 4.85 (s, 1H), 4.51 (s, 1H), 3.88 (d, *J* = 7.2 Hz, 1H), 3.78 (dd, *J* = 10.7, 5.6 Hz, 1H), 3.70 (d, *J* = 11.5 Hz, 1H), 3.06 (dd, *J* = 7.6, 3.7 Hz, 1H), 2.98 (d, *J* = 11.5 Hz, 1H), 2.24–2.03 (m, 3H), 1.74 (dd, *J* = 20.3, 8.7 Hz, 2H), 1.40–1.33 (m, 1H), 1.32 (s, 3H), 1.31–1.24 (m, 1H), 1.28 (s, 3H), 1.12 (m, 1H), 0.98–0.85 (m, 1H), 0.92 (s, 3H), 0.84–0.73 (m, 1H), 0.71 (s, 3H), 0.70–0.60 (m, 2H); ^13^C NMR (101 MHz, C_6_D_6_) *δ* 168.55, 152.54, 149.60, 147.88, 135.21, 127.02, 126.27, 126.02, 125.83, 125.54, 122.47, 121.65, 107.86, 105.47, 99.17, 74.98, 71.22, 70.42, 64.02, 56.55, 50.80, 38.39, 37.95, 37.74, 33.27, 26.47, 25.68, 25.07, 24.08, 23.17, 16.35, 14.06; ESI-HRMS: *m*/*z* 539.2768 [M + Na]^+^, calcd for C_33_H_40_NaO_5_, 539.2773.

##### (14α)-(2′-Nitro-pyridinyl-3′-oxy)-3,19-acetonylidene andrographolide (5a15)

White solid; mp 174 °C to 176 °C; 53% yield; ^1^H NMR (400 MHz, (CD_3_)_2_SO) *δ* 8.21 (dd, *J* = 4.5, 1.0 Hz, 1H), 8.05–8.00 (m, 1H), 7.84 (m, 4.6 Hz, 1H), 6.94 (t, *J* = 6.1 Hz, 1H), 6.07 (d, *J* = 5.2 Hz, 1H), 4.81 (s, 1H), 4.73 (m, 1H), 4.50–4.43 (m, 2H), 3.83 (d, *J* = 11.7 Hz, 1H), 3.38–3.34 (m, 1H), 3.08 (d, *J* = 11.6 Hz, 1H), 2.48–2.41 (m, 1H), 2.40–2.29 (m, 2H), 1.96 (m, 2H), 1.81 (m, 1H), 1.68–1.55 (m, 2H), 1.52–1.43 (m, 1H), 1.28 (m, 3H), 1.23 (s, 3H), 1.21–1.25 (m, 1H), 1.21–1.13 (m, 2H), 1.11 (s, 3H), 0.77 (s, 3H); ^13^C NMR (101 MHz, (CD_3_)_2_SO) *δ* 168.57, 151.44, 149.03, 147.50, 144.23, 140.41, 129.46, 126.43, 124.08, 108.52, 98.14, 75.70, 73.14, 70.22, 62.73, 54.90, 51.35, 37.86, 37.13, 36.90, 33.88, 27.41, 25.75, 25.15, 24.74, 24.72, 22.57, 15.57; ESI-HRMS: *m*/*z* 535.2415 [M + Na]^+^, calcd for C_28_H_36_N_2_NaO_7_, 535.2420.

##### (14β)-(2′-Nitro-pyridinyl-3′-oxy)-3,19-acetonylidene andrographolide (5b15)

White solid; mp 104 °C to 107 °C; 42% yield; ^1^H NMR (400 MHz, C_6_D_6_) *δ* 7.53 (d, *J* = 4.6 Hz, 1H), 6.32 (m, 1H), 6.10 (m, 1H), 4.82 (s, 1H), 4.76 (s, 1H), 4.33 (s, 1H), 3.82 (d, *J* = 11.5 Hz, 1H), 3.51 (m, 2H), 3.44 (m, 1H), 3.07 (d, *J* = 11.5 Hz, 1H), 2.30–2.12 (m, 3H), 1.87–1.66 (m, 2H), 1.63–1.47 (m, 3H), 1.42 (s, 3H), 1.39–1.31 (m, 1H), 1.36 (s, 3H), 1.10 (s, 3H), 1.04–0.88 (m, 3H), 0.90 (s, 3H); ^13^C NMR (101 MHz, C_6_D_6_) *δ* 167.65, 152.42, 151.04, 148.50, 143.65, 140.77, 127.47, 124.75, 123.67, 107.70, 99.31, 75.29, 73.91, 69.17, 64.12, 55.77, 51.06, 38.40, 38.13, 37.58, 33.67, 26.56, 26.05, 25.93, 25.21, 24.63, 23.19, 16.51; ESI-HRMS: *m*/*z* 535.2415 [M + Na]^+^, calcd for C_28_H_36_N_2_NaO_7_, 535.2420.

##### (14α)-(Quinolyl-4′-oxy)-3,19-acetonylidene andrographolide (5a16)

White solid; mp 97.8 °C to 102.9 °C; 40% yield; ^1^H NMR (400 MHz, (CD_3_)_2_SO) *δ* 8.79 (d, *J* = 5.2 Hz, 1H), 8.07 (d, *J* = 8.3 Hz, 1H), 7.99 (d, *J* = 8.4 Hz, 1H), 7.79–7.74 (m, 1H), 7.57 (t, *J* = 7.6 Hz, 1H), 7.11–7.04 (m, 2H), 6.12 (d, *J* = 6.9 Hz, 1H), 4.88–4.81 (m, 2H), 4.63 (s, 1H), 4.47–4.42 (m, 1H), 3.76 (d, *J* = 11.7 Hz, 1H), 3.27 (dd, *J* = 9.2, 3.9 Hz, 1H), 3.02 (d, *J* = 11.5 Hz, 1H), 2.31 (d, *J* = 11.7 Hz, 1H), 2.02 (s, 1H), 1.92 (s, 1H), 1.76–1.57 (m, 3H), 1.53–1.43 (m, 1H), 1.42–1.30 (m, 2H), 1.23 (s, 3H), 1.20 (s, 3H), 1.15–1.12 (m, 2H), 1.10 (s, 1H), 1.06–1.04 (m, 3H), 0.66 (d, *J* = 9.5 Hz, 3H), 0.65 (s, 3H); ^13^C NMR (101 MHz, (CD_3_)_2_SO) *δ* 169.4, 159.3, 151.9, 151.5, 149.5, 148.2, 132.1, 130.5, 129.2, 126.5, 124.9, 121.8, 109.0, 103.0, 98.6, 76.1, 72.1, 71.1, 63.1, 55.2, 51.8, 38.3, 37.5, 37.3, 34.3, 27.9, 26.2, 25.6, 25.5, 25.1, 23.0, 16.0; ESI-HRMS: *m*/*z* 518.2902 [M + H]^+^, calcd for C_32_H_40_NO_5_, 518.2906.

##### (14β)-(Quinolyl-4′-oxy)-3,19-acetonylidene andrographolide (5b16)

White solid; mp 122 °C to 124 °C; 53% yield; ^1^H NMR (400 MHz, C_6_D_6_) *δ* 8.63 (d, *J* = 5.1 Hz, 1H), 8.31–8.25 (m, 1H), 7.98 (dd, *J* = 8.4, 1.4 Hz, 1H), 7.42 (m, 1H), 7.37–7.30 (m, 1H), 5.66–5.62 (m, 1H), 4.89–4.82 (m, 2H), 4.51 (d, *J* = 1.7 Hz, 1H), 3.76–3.65 (m, 3H), 3.10 (dd, *J* = 7.1, 3.5 Hz, 1H), 2.98 (d, *J* = 11.5 Hz, 1H), 2.23–2.05 (m, 3H), 1.80–1.63 (m, 2H), 1.34–1.24 (m, 2H), 1.30 (s, 3H), 1.28 (s, 3H), 1.14 (m, 1H), 1.00–0.91 (m, 1H), 0.90 (s, 3H), 0.76 (s, 3H), 0.74–0.59 (m, 3H); ^13^C NMR (101 MHz, C_6_D_6_) *δ* 168.22, 158.91, 151.10, 150.54, 150.43, 147.82, 130.19, 129.97, 128.15, 127.91, 127.79, 127.67, 126.07, 125.40, 121.97, 121.27, 107.91, 100.92, 99.36, 74.50, 71.28, 69.95, 64.12, 56.67, 50.62, 38.42, 38.08, 37.75, 33.15, 26.10, 25.73, 25.63, 24.94, 23.67, 23.18, 16.53; ESI-HRMS: *m*/*z* 518.2900 [M + H]^+^, calcd for C_32_H_40_NaO_5_, 518.2906.

##### (14α)-(Quinolyl-8′-oxy)-3,19-acetonylidene andrographolide (5a17)

White solid; mp 143 °C to 146 °C; 42% yield; ^1^H NMR (400 MHz, C_6_D_6_) *δ* 8.64 (dd, *J* = 4.1, 1.7 Hz, 1H), 7.46 (dd, *J* = 8.4, 1.7 Hz, 1H), 7.27–7.22 (m, 1H), 7.08 (dd, *J* = 8.2, 1.3 Hz, 1H), 6.99 (t, *J* = 7.8 Hz, 1H), 6.88 (m, 1H), 6.74 (dd, *J* = 8.3, 4.1 Hz, 1H), 6.08 (d, *J* = 5.6 Hz, 1H), 4.82 (s, 1H), 4.69 (s, 1H), 4.39 (dd, *J* = 10.8, 1.6 Hz, 1H), 3.88 (dd, *J* = 10.8, 5.6 Hz, 1H), 3.79 (d, *J* = 11.5 Hz, 1H), 3.41 (dd, *J* = 8.1, 3.9 Hz, 1H), 3.06 (d, *J* = 11.5 Hz, 1H), 2.35 (m, 1H), 2.25–2.11 (m, 2H), 1.81 (m, 1H), 1.74–1.64 (m, 1H), 1.55–1.48 (m, 1H), 1.48–1.40 (m, 1H), 1.42 (s, 3H), 1.36 (s, 3H), 1.32–1.23 (m, 2H), 1.08 (s, 3H), 0.99–0.78 (m, 3H), 0.71 (s, 3H); ^13^C NMR (101 MHz, C_6_D_6_) *δ* 169.33, 153.10, 149.55, 149.07, 147.00, 142.46, 135.98, 130.11, 126.78, 126.52, 123.22, 121.41, 119.68, 109.42, 99.18, 75.65, 75.33, 70.92, 64.00, 55.65, 51.54, 38.19, 38.00, 37.68, 34.09, 26.83, 26.09, 25.63, 25.39, 25.04, 23.18, 16.12; ESI-HRMS: *m*/*z* 518.2901 [M + H]^+^, calcd for C_32_H_40_NO_5_, 518.2906.

##### (14β)-(Quinolyl-8′-oxy)-3,19-acetonylidene andrographolide (5b17)

White solid; mp 146 °C to 148 °C; 87% yield; ^1^H NMR (400 MHz, C_6_D_6_) *δ* 8.67 (dd, *J* = 4.1, 1.7 Hz, 1H), 7.49 (dd, *J* = 8.3, 1.7 Hz, 1H), 7.30–7.24 (m, 1H), 7.08 (m, 1H), 7.01 (m, 1H), 6.75 (m, 2H), 5.97 (d, *J* = 5.5 Hz, 1H), 4.84 (d, *J* = 1.2 Hz, 1H), 4.54 (s, 1H), 4.25 (dd, *J* = 10.7, 1.6 Hz, 1H), 3.84–3.75 (m, 2H), 3.28 (m, 1H), 3.06 (d, *J* = 11.5 Hz, 1H), 2.37 (m, 1H), 2.30–2.21 (m, 1H), 2.21–2.13 (m, 1H), 1.74 (m, 1H), 1.69–1.58 (m, 2H), 1.39 (s, 3H), 1.36–1.30 (m, 1H), 1.34 (s, 3H), 1.29–1.19 (m, 2H), 1.04 (m, 3H), 1.01–0.92 (m, 1H), 0.84 (m, 2H), 0.75 (s, 3H); ^13^C NMR (101 MHz, C_6_D_6_) *δ* 169.13, 153.03, 149.84, 149.10, 148.26, 142.17, 135.83, 130.10, 126.66, 126.65, 122.68, 121.51, 117.63, 107.87, 99.13, 75.55, 74.63, 70.62, 64.03, 55.77, 51.17, 38.37, 37.99, 37.69, 33.57, 26.74, 25.99, 25.99, 25.31, 24.74, 23.30, 16.25; ESI-HRMS: *m*/*z* 518.2880 [M + H]^+^, calcd for C_32_H_40_NO_5_, 518.2906.

#### 4.2.2. Preparation of compounds 6a and 6b

##### General method for synthesis of 6a and 6b

At room temperature, 1.0 mmol 5a or 5b were dissolved in 15 mL methanol, and 0.1 mmol TsOH·H_2_O was added. The reaction was stirred at rt and was generally complete in 5 min. After the reaction was complete, the reaction mixture was treated with sol. sat. NaHCO_3_ and extracted with ethyl acetate. The organic phase was washed with brine and dried over anhydrous Na_2_SO_4_. The solvent was removed *in vacuo*, and the residue was further purified using flash column chromatography eluting with petroleum ether and ethyl acetate to afford 6a or 6b.

##### (14α)-(2′-Carboxy ethyl ester-4′-nitro)phenoxy-andrographolide (6a1)

White solid; mp 82.6 °C to 92.7 °C; 97% yield; ^1^H NMR (400 MHz, (CD_3_)_2_SO) *δ* 8.51 (d, *J* = 2.9 Hz, 1H), 8.44 (dd, *J* = 9.2, 3.0 Hz, 1H), 7.40 (d, *J* = 9.3 Hz, 1H), 6.99–6.93 (m, 1H), 6.05 (d, *J* = 5.4 Hz, 1H), 5.00 (d, *J* = 4.9 Hz, 1H), 4.81–4.74 (m, 2H), 4.47 (s, 1H), 4.39 (dd, *J* = 11.0, 1.5 Hz, 1H), 4.09 (dd, *J* = 7.5, 2.9 Hz, 1H), 3.79 (s, 3H), 3.79–3.74 (m, 1H), 3.24–3.11 (m, 2H), 2.48–2.44 (m, 1H), 2.38–2.27 (m, 2H), 1.96–1.86 (m, 2H), 1.70 (d, *J* = 13.2 Hz, 1H), 1.57–1.43 (m, 3H), 1.31 (m, 1H), 1.17–1.10 (m, 1H), 1.08–1.01 (m, 1H), 1.04 (s, 3H), 0.52 (s, 3H); ^13^C NMR (101 MHz, (CD_3_)_2_SO) *δ* 169.2, 164.5, 160.7, 151.7, 148.2, 141.3, 129.2, 127.2, 124.6, 122.1, 116.0, 108.5, 78.8, 73.4, 70.9, 63.0, 55.5, 54.8, 53.1, 42.7, 38.9, 37.8, 36.9, 28.2, 24.9, 24.3, 23.5, 14.9; ESI-HRMS: *m*/*z* 552.2203 [M + Na]^+^, calcd for C_28_H_35_NNaO_9_, 552.2210.

##### (14β)-(2′-Carboxy ethyl ester-4′-nitro)phenoxy-andrographolide (6b1)

White solid; mp 190 °C to 192 °C; 78% yield; ^1^H NMR (400 MHz, (CD_3_)_2_SO) *δ* 8.58 (d, *J* = 2.9 Hz, 1H), 8.49 (dd, *J* = 9.2, 3.0 Hz, 1H), 7.44 (d, *J* = 9.3 Hz, 1H), 7.02 (t, *J* = 6.9 Hz, 1H), 6.15 (d, *J* = 5.2 Hz, 1H), 4.99 (d, *J* = 4.8 Hz, 1H), 4.83 (s, 1H), 4.77 (dd, *J* = 11.1, 5.5 Hz, 1H), 4.55 (s, 1H), 4.41 (d, *J* = 11.0 Hz, 1H), 4.07 (dd, *J* = 7.5, 2.8 Hz, 1H), 3.82 (s, 3H), 3.75 (dd, *J* = 11.0, 2.8 Hz, 1H), 3.20 (m, 1H), 3.02–2.95 (m, 1H), 2.44 (m, 1H), 2.46–2.30 (m, 2H), 1.90 (m, 2H), 1.71 (m, 1H), 1.55–1.38 (m, 2H), 1.38–1.24 (m, 2H), 1.10–1.00 (m, 1H), 1.04 (s, 3H), 0.86 (m, 1H), 0.54 (s, 3H); ^13^C NMR (101 MHz, (CD_3_)_2_SO) *δ* 168.69, 163.67, 160.48, 151.11, 147.59, 140.66, 129.03, 126.96, 124.68, 120.98, 115.50, 107.80, 78.25, 72.78, 70.46, 62.47, 55.62, 54.18, 52.51, 42.12, 38.67, 37.35, 36.04, 27.71, 25.03, 23.86, 22.95, 14.54; ESI-HRMS: *m*/*z* 552.2204 [M + Na]^+^, calcd for C_28_H_35_NNaO_9_, 552.2210.

##### (14α)-(2′-Fluoro-4′-nitro)phenoxy-andrographolide (6a2)

White solid; mp 172 °C to 175 °C; 87% yield; ^1^H NMR (400 MHz, (CD_3_)_2_SO) *δ* 8.25 (dd, *J* = 10.9, 2.7 Hz, 1H), 8.18–8.13 (m, 1H), 7.48 (t, *J* = 8.7 Hz, 1H), 6.96 (t, *J* = 6.3 Hz, 1H), 6.05 (d, *J* = 5.3 Hz, 1H), 5.03 (d, *J* = 4.9 Hz, 1H), 4.79 (s, 1H), 4.74 (m, 1H), 4.54 (s, 1H), 4.44 (d, *J* = 12.2 Hz, 1H), 4.11 (m, 1H), 3.78 (dd, *J* = 11.0, 2.9 Hz, 1H), 3.19 (m, 2H), 2.45 (s, 1H), 2.42–2.26 (m, 2H), 1.98–1.87 (m, 2H), 1.71 (m, 1H), 1.54 (m, 3H), 1.38–1.25 (m, 1H), 1.18–1.10 (m, 2H), 1.06 (s, 3H), 0.54 (s, 3H); ^13^C NMR (101 MHz, (CD_3_)_2_SO) *δ* 168.64, 152.22, 151.45, 150.42, 150.32, 149.74, 147.67, 141.12, 141.04, 124.11, 121.27, 121.23, 115.72, 112.68, 112.45, 108.05, 78.36, 73.02, 70.34, 62.53, 55.12, 54.30, 42.18, 38.43, 37.36, 36.35, 27.77, 24.80, 23.84, 23.01, 14.52; ESI-HRMS: *m*/*z* 512.2054 [M + Na]^+^, calcd for C_26_H_32_FNNaO_7_, 512.2061.

##### (14β)-(2′-Fluoro-4′-nitro)phenoxy-andrographolide (6b2)

White solid; mp 162 °C to 165 °C; 84% yield; ^1^H NMR (400 MHz, CD_3_OD) *δ* 8.19–8.11 (m, 2H), 7.34 (t, *J* = 8.4 Hz, 1H), 7.13–7.04 (m, 1H), 6.03–5.97 (m, 1H), 4.72 (dd, *J* = 11.2, 5.5 Hz, 1H), 4.56 (s, 1H), 4.45 (dd, *J* = 11.2, 1.4 Hz, 1H), 4.02 (d, *J* = 11.1 Hz, 1H), 3.32 (m, 1H), 3.21 (dd, *J* = 11.8, 4.1 Hz, 1H), 2.52 (m, 1H), 2.45–2.34 (m, 2H), 2.07–1.92 (m, 2H), 1.87–1.78 (m, 1H), 1.73–1.46 (m, 3H), 1.41–1.28 (m, 1H), 1.22 (m, 1H), 1.17 (s, 3H), 1.10–0.98 (m, 1H), 0.64 (s, 3H); ^13^C NMR (101 MHz, CD_3_OD) *δ* 171.05, 154.37, 152.98, 151.89, 151.83, 151.73, 148.94, 143.42, 143.34, 126.12, 122.24, 122.20, 116.71, 113.79, 113.56, 108.54, 80.83, 74.64, 72.22, 64.88, 57.72, 56.33, 43.63, 40.24, 38.93, 37.90, 28.89, 26.76, 25.19, 23.39, 15.42; ESI-HRMS: *m*/*z* 512.2099 [M + Na]^+^, calcd for C_26_H_32_FNNaO_7_, 512.2061.

##### (14α)-(2′-Methoxy-4′-nitro)phenoxy-andrographolide (6a3)^25,26^

Yellow solid; mp 155 °C to 159 °C; 87% yield; ^1^H NMR (400 MHz, CD_3_OD) *δ* 7.95 (dd, *J* = 8.8, 2.6 Hz, 1H), 7.92 (d, *J* = 2.6 Hz, 1H), 7.16 (d, *J* = 8.8 Hz, 1H), 7.12–7.03 (m, 1H), 5.87 (d, *J* = 5.7 Hz, 1H), 4.91 (s, 1H), 4.73 (dd, *J* = 10.9, 5.8 Hz, 1H), 4.67 (s, 1H), 4.46 (dd, *J* = 10.9, 1.8 Hz, 1H), 4.10 (d, *J* = 11.1 Hz, 1H), 3.98 (s, 3H), 3.38 (t, *J* = 5.4 Hz, 1H), 2.58–2.40 (m, 3H), 2.09–1.95 (m, 2H), 1.90–1.83 (m, 1H), 1.80–1.60 (m, 3H), 1.44–1.32 (m, 1H), 1.32–1.17 (m, 2H), 1.22 (s, 3H), 0.66 (s, 3H); ^13^C NMR (101 MHz, CD_3_OD) *δ* 171.44, 153.11, 152.54, 151.94, 148.72, 144.43, 126.17, 118.27, 116.57, 109.47, 108.44, 80.88, 74.75, 72.58, 64.91, 57.17, 56.86, 56.29, 43.65, 39.84, 38.87, 38.15, 28.96, 26.36, 25.17, 23.35, 15.43; ESI-HRMS: *m*/*z* 524.2253 [M + Na]^+^, calcd for C_27_H_35_NNaO_8_, 524.2260.

##### (14β)-(2′-Methoxy-4′-nitro)phenoxy-andrographolide (6b3)^25,26^

Yellow solid; mp 151 °C to 153 °C; 90% yield; ^1^H NMR (400 MHz, CDCl_3_) *δ* 7.89 (m, 1H), 7.84–7.79 (m, 1H), 7.13–7.05 (m, 1H), 6.88 (dd, *J* = 9.2, 4.2 Hz, 1H), 5.71–5.65 (m, 1H), 4.83 (d, *J* = 1.9 Hz, 1H), 4.58 (dd, *J* = 11.0, 5.8 Hz, 1H), 4.42 (dd, *J* = 11.0, 1.8 Hz, 1H), 4.34 (s, 1H), 4.16–4.08 (m, 1H), 3.96 (s, 3H), 3.38 (dd, *J* = 11.6, 3.5 Hz, 1H), 3.28 (d, *J* = 11.1 Hz, 1H), 2.58 (d, *J* = 52.7 Hz, 2H), 2.44–2.36 (m, 2H), 2.32–2.21 (m, 1H), 1.97 (m, 1H), 1.89 (dd, *J* = 9.3, 2.7 Hz, 1H), 1.85–1.78 (m, 1H), 1.78–1.60 (m, 2H), 1.52 (m, 1H), 1.26–1.16 (m, 2H), 1.22 (s, 3H), 1.14–1.03 (m, 1H), 0.61 (s, 3H); ^13^C NMR (101 MHz, CDCl_3_) *δ* 168.99, 151.84, 151.03, 150.56, 147.09, 143.34, 124.42, 117.39, 116.00, 108.22, 107.50, 80.35, 77.37, 77.26, 77.05, 76.73, 73.32, 70.60, 64.04, 56.36, 55.75, 55.06, 42.82, 38.93, 37.65, 36.64, 28.16, 25.71, 23.70, 22.77, 15.12; ESI-HRMS: *m*/*z* 524.2253 [M + Na]^+^, calcd for C_27_H_35_NNaO_8_, 524.2260.

##### (14α)-(3′-Fluoro-4′-nitro)phenoxy-andrographolide (6a4)

White solid; mp 191 °C to 194 °C; 86% yield; ^1^H NMR (400 MHz, (CD_3_)_2_SO) *δ* 8.10 (t, *J* = 9.1 Hz, 1H), 7.65 (s, 1H), 7.13 (dd, *J* = 13.4, 2.6 Hz, 1H), 6.90 (dd, *J* = 9.3, 2.3 Hz, 1H), 5.11 (s, 1H), 5.04 (d, *J* = 4.9 Hz, 1H), 4.93–4.86 (m, 1H), 4.89 (s, 3H), 4.11 (m, 1H), 3.82 (m, 1H), 3.24 (m, 2H), 2.31 (m, 1H), 2.03–1.91 (m, 2H), 1.89–1.59 (m, 6H), 1.40–1.27 (m, 1H), 1.26–1.11 (m, 2H), 1.05 (m, 3H), 0.62 (s, 3H); ^13^C NMR (101 MHz, (CD_3_)_2_SO) *δ* 172.00, 163.60, 163.49, 157.78, 155.17, 149.66, 146.99, 131.27, 130.46, 130.40, 128.16, 111.88, 111.86, 107.72, 105.08, 104.84, 78.29, 72.99, 71.10, 62.58, 54.48, 51.23, 42.27, 38.45, 37.77, 36.50, 29.11, 27.80, 23.99, 22.95, 14.77; ESI-HRMS: *m*/*z* 512.2055 [M + Na]^+^, calcd for C_26_H_32_FNNaO_7_, 512.2061.

##### (14β)-(3′-Fluoro-4′-nitro)phenoxy-andrographolide (6b4)

Yellow solid; mp 163 °C to 166 °C; 90% yield; ^1^H NMR (400 MHz, CD_3_OD) *δ* 8.20 (t, *J* = 9.0 Hz, 1H), 7.18–7.06 (m, 2H), 6.99 (m, 1H), 5.97 (d, *J* = 5.3 Hz, 1H), 4.70 (dd, *J* = 11.1, 5.5 Hz, 1H), 4.57 (s, 1H), 4.40 (dd, *J* = 11.1, 1.3 Hz, 1H), 4.04 (d, *J* = 11.1 Hz, 1H), 3.61 (q, *J* = 7.0 Hz, 3H), 3.33 (s, 1H), 3.24 (m, 1H), 2.56 (m, 1H), 2.47–2.36 (m, 2H), 2.02 (m, 1H), 1.98–1.91 (m, 1H), 1.89–1.79 (m, 1H), 1.63 (m, 3H), 1.42–1.20 (m, 2H), 1.18 (s, 3H), 1.13–1.01 (m, 1H), 0.66 (s, 3H); ^13^C NMR (101 MHz, CD_3_OD) *δ* 171.07, 163.94, 163.83, 159.93, 157.31, 152.70, 148.90, 132.84, 132.77, 129.41, 126.15, 113.08, 113.05, 108.63, 106.14, 105.90, 80.82, 73.80, 72.22, 64.90, 57.52, 56.34, 43.65, 40.25, 38.95, 38.09, 28.91, 26.76, 25.20, 23.39, 15.44; ESI-HRMS: *m*/*z* 512.2093 [M + Na]^+^, calcd for C_26_H_32_FNNaO_7_, 512.2061.

##### (14α)-(3′-methoxy-4′-nitro)phenoxy-andrographolide (6a5)

White solid; mp 131 °C to 133 °C; 81% yield; ^1^H NMR (400 MHz, (CD_3_)_2_SO) *δ* 7.99 (d, *J* = 9.1 Hz, 1H), 6.96 (t, *J* = 6.8 Hz, 1H), 6.85 (d, *J* = 2.4 Hz, 1H), 6.73 (dd, *J* = 9.1, 2.4 Hz, 1H), 6.01 (d, *J* = 5.0 Hz, 1H), 5.04 (d, *J* = 4.9 Hz, 1H), 4.83 (s, 1H), 4.71 (dd, *J* = 11.0, 5.4 Hz, 1H), 4.56 (s, 1H), 4.39–4.33 (m, 1H), 4.12 (dd, *J* = 7.4, 2.9 Hz, 1H), 3.78 (dd, *J* = 11.0, 2.8 Hz, 1H), 3.26–3.14 (m, 2H), 2.44 (dd, *J* = 5.8, 3.0 Hz, 1H), 2.36–2.28 (m, 2H), 1.97–1.87 (m, 2H), 1.76–1.68 (m, 1H), 1.60–1.48 (m, 3H), 1.36–1.22 (m, 2H), 1.18–1.13 (m, 3H), 1.06 (s, 3H), 0.90–0.76 (m, 1H), 0.55 (s, 3H); ^13^C NMR (101 MHz, CDCl_3_) *δ* 168.7, 161.5, 155.8, 151.9, 146.6, 134.2, 128.7, 123.8, 109.1, 104.6, 101.7, 80.4, 71.8, 70.4, 64.1, 56.7, 55.8, 55.2, 42.8, 38.8, 37.7, 37.0, 28.1, 25.4, 23.6, 22.7, 15.2; ESI-HRMS: *m*/*z* 524.2252 [M + Na]^+^, calcd for C_27_H_35_NNaO_8_, 524.2260.

##### (14β)-(3′-Methoxy-4′-nitro)phenoxy-andrographolide (6b5)

Yellow solid; mp 126 °C to 129 °C; 85% yield; ^1^H NMR (400 MHz, CD_3_OD) *δ* 8.02 (d, *J* = 9.1 Hz, 1H), 7.15 (t, *J* = 6.6 Hz, 1H), 6.85 (d, *J* = 2.5 Hz, 1H), 6.71 (dd, *J* = 9.1, 2.5 Hz, 1H), 5.98 (d, *J* = 5.4 Hz, 1H), 4.72 (dd, *J* = 11.0, 5.5 Hz, 1H), 4.60 (s, 1H), 4.42 (dd, *J* = 11.0, 1.3 Hz, 1H), 4.06 (d, *J* = 11.1 Hz, 1H), 3.99 (s, 3H), 3.35 (d, *J* = 4.2 Hz, 1H), 3.25 (dd, *J* = 11.8, 4.1 Hz, 1H), 2.60–2.52 (m, 1H), 2.42 (m, 2H), 2.10–2.00 (m, 1H), 1.97 (m, 1H), 1.90–1.81 (m, 1H), 1.76–1.64 (m, 1H), 1.64–1.54 (m, 2H), 1.42–1.30 (m, 1H), 1.24 (m, 1H), 1.20 (s, 3H), 1.15–1.05 (m, 1H), 0.68 (s, 3H); ^13^C NMR (101 MHz, CD_3_OD) *δ* 171.33, 163.25, 156.92, 152.16, 148.93, 135.26, 129.27, 126.63, 108.56, 107.53, 102.64, 80.83, 73.31, 72.52, 64.88, 57.55, 57.34, 56.33, 43.65, 40.24, 38.95, 38.03, 28.91, 26.71, 25.20, 23.38, 15.41; ESI-HRMS: *m*/*z* 524.2254 [M + Na]^+^, calcd for C_27_H_35_NNaO_8_, 524.2260.

##### (14β)-(2′-Nitro)phenoxy-andrographolide (6b6)

Yellow solid; mp 167 °C to 168 °C; 89% yield; ^1^H NMR (400 MHz, CDCl_3_) *δ* 7.89 (dd, *J* = 8.1, 1.6 Hz, 1H), 7.62–7.55 (m, 1H), 7.18 (t, *J* = 7.7 Hz, 1H), 7.13 (dd, *J* = 10.1, 3.9 Hz, 1H), 6.92 (d, *J* = 8.3 Hz, 1H), 5.64 (d, *J* = 5.6 Hz, 1H), 4.84 (d, *J* = 15.2 Hz, 1H), 4.66 (dd, *J* = 10.9, 6.0 Hz, 1H), 4.44 (dd, *J* = 10.9, 2.0 Hz, 1H), 4.31 (s, 1H), 4.16–4.09 (m, 1H), 3.43 (dd, *J* = 11.5, 4.6 Hz, 1H), 3.28 (d, *J* = 11.1 Hz, 1H), 2.56–2.45 (m, 1H), 2.38 (dd, *J* = 13.2, 3.5 Hz, 1H), 2.26 (m, 1H), 2.09 (s, 3H), 1.98 (t, *J* = 12.3 Hz, 1H), 1.89 (d, *J* = 10.4 Hz, 1H), 1.85–1.78 (m, 1H), 1.78–1.63 (m, 2H), 1.59 (m, 1H), 1.31–1.18 (m, 2H), 1.24 (s, 3H), 1.10 (m, 1H), 0.58 (s, 3H); ^13^C NMR (101 MHz, CDCl_3_) *δ* 168.74, 152.64, 149.38, 147.27, 141.14, 134.14, 126.28, 123.69, 122.33, 115.90, 108.07, 80.31, 77.37, 77.25, 77.05, 76.73, 73.14, 70.30, 64.10, 55.80, 54.86, 42.80, 38.89, 37.63, 36.54, 28.12, 25.81, 23.69, 22.63, 15.12; ESI-HRMS: *m*/*z* 494.2158 [M + Na]^+^, calcd for C_26_H_33_NNaO_7_, 494.2155.

##### (14β)-(3′-Nitro)phenoxy-andrographolide (6b7)

Yellow solid; mp 144 °C to 146 °C; 93% yield; ^1^H NMR (400 MHz, CDCl_3_) *δ* 7.94 (m, 1H), 7.69 (t, *J* = 2.3 Hz, 1H), 7.58–7.49 (m, 1H), 7.20 (m, 1H), 7.17–7.11 (m, 1H), 5.62 (m, 1H), 4.87 (dd, *J* = 2.5, 1.3 Hz, 1H), 4.72–4.63 (m, 1H), 4.45–4.35 (m, 2H), 4.16–4.08 (m, 1H), 3.41 (m, 1H), 3.33–3.25 (m, 1H), 2.51 (m, 1H), 2.46–2.38 (m, 1H), 2.37–2.23 (m, 1H), 2.29 (s, 3H), 2.01–1.93 (m, 1H), 1.93–1.87 (m, 1H), 1.83 (m, 1H), 1.74 (m, 1H), 1.62 (m, 2H), 1.34–1.18 (m, 2H), 1.24 (s, 3H), 1.18–1.06 (m, 1H), 0.60 (s, 3H); ^13^C NMR (101 MHz, CDCl_3_) *δ* 168.90, 156.99, 151.67, 149.36, 146.91, 130.85, 124.19, 122.41, 117.26, 109.70, 108.28, 80.29, 77.38, 77.06, 76.74, 71.95, 70.39, 64.05, 55.77, 55.15, 42.80, 39.00, 37.68, 36.91, 28.05, 25.74, 23.68, 22.72, 15.10; ESI-HRMS: *m*/*z* 494.2156 [M + Na]^+^, calcd for C_26_H_33_NNaO_7_, 494.2155.

##### (14α)-(2′-Carboxy ethyl ester)phenoxy-andrographolide (6a8)

White solid; mp 157 °C to 159 °C; 96% yield; ^1^H NMR (400 MHz, C_6_D_6_) *δ* 7.78 (dd, *J* = 7.7, 1.8 Hz, 1H), 7.21 (t, *J* = 6.0 Hz, 1H), 6.98–6.92 (m, 1H), 6.71 (t, *J* = 7.6 Hz, 1H), 6.38 (d, *J* = 8.2 Hz, 1H), 5.11 (s, 1H), 4.84 (s, 1H), 4.57 (s, 1H), 4.19–4.11 (m, 2H), 4.09 (dd, *J* = 8.8, 4.0 Hz, 1H), 3.78 (dd, *J* = 17.5, 8.6 Hz, 2H), 3.44 (dd, *J* = 7.9, 3.7 Hz, 1H), 3.07 (d, *J* = 11.5 Hz, 1H), 2.22–2.13 (m, 3H), 1.89–1.80 (m, 1H), 1.78–1.67 (m, 1H), 1.60–1.51 (m, 1H), 1.51–1.43 (m, 2H), 1.42 (s, 3H), 1.35 (s, 3H), 1.31 (d, *J* = 2.5 Hz, 1H), 1.09 (s, 3H), 1.06 (t, *J* = 7.1 Hz, 3H), 0.99–0.88 (m, 3H), 0.80 (s, 3H); ^13^C NMR (101 MHz, C_6_D_6_) *δ* 168.70, 165.60, 156.04, 149.51, 147.00, 132.85, 132.04, 125.51, 124.38, 122.50, 117.83, 109.13, 99.26, 75.48, 73.78, 70.37, 64.02, 60.96, 55.62, 51.54, 38.23, 38.07, 37.66, 34.21, 26.70, 26.05, 25.29, 25.24, 24.89, 23.15, 16.22, 14.10; ESI-HRMS: *m*/*z*: 521.2509 [M + Na]^+^, calcd for C_29_H_38_NaO_7_, 521.2515.

##### (14β)-(2′-Carboxy ethyl ester)phenoxy-andrographolide (6b8)

White solid; mp 181 °C to 182 °C; 88% yield; ^1^H NMR (400 MHz, CDCl_3_) *δ* 7.88 (dd, *J* = 7.8, 1.7 Hz, 1H), 7.54–7.48 (m, 1H), 7.15 (t, *J* = 7.6 Hz, 1H), 7.04 (t, *J* = 7.0 Hz, 1H), 6.91 (d, *J* = 8.3 Hz, 1H), 5.61 (d, *J* = 5.3 Hz, 1H), 4.83 (d, *J* = 1.9 Hz, 1H), 4.58 (dd, *J* = 10.7, 5.4 Hz, 1H), 4.52 (dd, *J* = 10.7, 1.7 Hz, 1H), 4.34 (dd, *J* = 14.7, 7.6 Hz, 3H), 4.14 (d, *J* = 11.1 Hz, 1H), 3.46–3.36 (m, 1H), 3.33–3.25 (m, 1H), 2.47–2.25 (m, 4H), 2.22–2.21 (m, 1H), 2.02–1.90 (m, 1H), 1.88–1.78 (m, 2H), 1.78–1.61 (m, 2H), 1.53–1.46 (m, 1H), 1.36 (t, 3H), 1.32–1.16 (m, 2H), 1.24 (s, 3H), 1.10–0.99 (m, 1H), 0.56 (s, 3H); ^13^C NMR (101 MHz, CDCl_3_) *δ* 169.45, 165.73, 155.78, 150.94, 147.11, 133.38, 132.26, 124.95, 123.22, 122.60, 117.01, 108.19, 80.33, 77.36, 77.25, 77.04, 76.73, 73.39, 70.98, 64.09, 61.16, 55.76, 54.90, 42.81, 38.81, 37.63, 36.55, 28.12, 25.64, 23.68, 22.64, 15.07, 14.29 ESI-HRMS: *m*/*z* 521.2517 [M + Na]^+^, calcd for C_29_H_39_NaO_7_, 521.2515.

##### (14α)-(4′-Carboxy ethyl ester)phenoxy-andrographolide (6a9)

White solid; mp 183 °C to 185 °C; 84% yield; ^1^H NMR (400 MHz, (CD_3_)_2_SO) *δ* 8.00–7.91 (m, 2H), 7.09 (d, *J* = 8.9 Hz, 2H), 6.93 (t, *J* = 6.2 Hz, 1H), 5.88 (d, *J* = 5.5 Hz, 1H), 5.01 (d, *J* = 4.7 Hz, 1H), 4.82 (s, 1H), 4.73 (dd, *J* = 10.9, 5.6 Hz, 1H), 4.58 (s, 1H), 4.33–4.26 (m, 3H), 4.10 (d, *J* = 5.1 Hz, 1H), 3.77 (d, *J* = 10.8 Hz, 1H), 3.24–3.13 (m, 2H), 2.43 (s, 1H), 2.37–2.27 (m, 2H), 1.96–1.87 (m, 2H), 1.71 (d, *J* = 13.1 Hz, 1H), 1.58–1.45 (m, 3H), 1.38–1.24 (m, 1H), 1.31 (t, *J* = 7.1 Hz, 3H), 1.12 (dd, *J* = 15.9, 12.0 Hz, 2H), 1.05 (s, 3H), 0.52 (s, 3H); ^13^C NMR (101 MHz, (CD_3_)_2_SO) *δ* 169.4, 165.7, 161.0, 150.9, 148.2, 131.9, 125.3, 123.7, 115.8, 108.5, 78.8, 71.8, 71.2, 63.0, 60.9, 55.6, 54.8, 42.7, 38.9, 37.9, 36.9, 28.3, 25.2, 24.3, 23.5, 21.2, 15.0, 14.7, 14.5; ESI-HRMS: *m*/*z* 521.2507 [M + Na]^+^, calcd for C_29_H_38_NaO_7_, 521.2515.

##### (14β)-(4′-Carboxy ethyl ester)phenoxy-andrographolide (6b9)

White solid; mp 136 °C to 139 °C; 95% yield; ^1^H NMR (400 MHz, CD_3_OD) *δ* 8.07–8.01 (m, 2H), 7.12 (t, *J* = 7.3 Hz, 1H), 7.09–7.04 (m, 2H), 5.88 (d, *J* = 5.4 Hz, 1H), 4.70 (dd, *J* = 10.9, 5.5 Hz, 1H), 4.56 (s, 1H), 4.40–4.31 (m, 3H), 4.03 (d, *J* = 11.1 Hz, 1H), 3.29 (s, 1H), 3.20 (dd, *J* = 11.9, 4.1 Hz, 1H), 2.55–2.47 (m, 1H), 2.45–2.30 (m, 2H), 2.08–1.91 (m, 2H), 1.86–1.79 (m, 1H), 1.71–1.58 (m, 1H), 1.58–1.48 (m, 2H), 1.42–1.27 (m, 4H), 1.24–1.14 (m, 1H), 1.17 (s, 3H), 1.09–0.96 (m, 1H), 0.63 (s, 3H); ^13^C NMR (101 MHz, CD_3_OD) *δ* 171.50, 167.59, 162.09, 151.93, 148.95, 132.93, 126.89, 125.30, 116.59, 108.53, 80.79, 72.88, 72.66, 64.90, 62.07, 57.61, 56.29, 43.62, 40.21, 38.95, 37.99, 28.87, 26.65, 25.18, 23.36, 15.43, 14.68; ESI-HRMS: *m*/*z* 521.2509 [M + Na]^+^, calcd for C_29_H_38_NaO_7_, 521.2515.

##### (14β)-(4′-Cyano)phenoxy-andrographolide (6b10)

White solid; mp 182 °C to 184 °C; 76% yield; ^1^H NMR (400 MHz, CD_3_OD) *δ* 7.73–7.68 (m, 2H), 7.13–7.06 (m, 3H), 5.86 (d, *J* = 5.5 Hz, 1H), 4.85 (s, 1H), 4.65 (dd, *J* = 11.0, 5.5 Hz, 1H), 4.52 (s, 1H), 4.31 (dd, *J* = 10.9, 1.4 Hz, 1H), 3.99 (d, *J* = 11.1 Hz, 1H), 3.30–3.35 (m, 1H), 3.18 (dd, *J* = 11.8, 4.2 Hz, 1H), 2.53–2.45 (m, 1H), 2.41–2.28 (m, 2H), 2.02–1.89 (m, 2H), 1.82–1.76 (m, 1H), 1.63 (m, 1H), 1.51 (m, 2H), 1.36–1.24 (m, 1H), 1.20–1.15 (m, 1H), 1.14 (s, 3H), 1.06–0.97 (m, 1H), 0.60 (s, 3H); ^13^C NMR (101 MHz, CD_3_OD) *δ* 171.30, 161.67, 152.20, 148.92, 135.62, 126.56, 119.68, 117.60, 108.51, 106.25, 80.79, 72.91, 72.40, 64.87, 57.60, 56.33, 43.62, 40.21, 38.93, 38.04, 28.86, 26.67, 25.17, 23.35, 15.40; ESI-HRMS: *m*/*z* 474.2252 [M + Na]^+^ calcd for C_27_H_33_NNaO_5_, 474.2256.

##### (14β)-(4′-Methoxy)phenoxy-andrographolide (6b11)

White solid; mp 178 °C to 180 °C; 75% yield; ^1^H NMR (400 MHz, (CD_3_)_2_SO) *δ* 6.97–6.88 (m, 4H), 6.87–6.82 (m, 1H), 5.66 (d, *J* = 5.4 Hz, 1H), 4.83–4.77 (m, 1H), 4.60 (dd, *J* = 10.7, 5.4 Hz, 1H), 4.46–4.40 (m, 1H), 4.33 (dd, *J* = 10.7, 1.4 Hz, 1H), 3.78 (d, *J* = 10.9 Hz, 1H), 3.72 (s, 3H), 3.21 (d, *J* = 10.8 Hz, 1H), 3.09 (dd, *J* = 11.2, 4.5 Hz, 1H), 2.30 (m, 1H), 2.21–2.12 (m, 2H), 1.97–1.83 (m, 2H), 1.71 (m, 1H), 1.61–1.22 (m, 4H), 1.14–0.91 (m, 2H), 1.05 (s, 3H), 0.55 (s, 3H); ^13^C NMR (101 MHz, (CD_3_)_2_SO) *δ* 169.24, 154.49, 150.27, 149.39, 147.73, 125.79, 117.95, 117.95, 114.82, 114.82, 107.75, 78.30, 72.48, 71.16, 62.55, 55.38, 55.36, 54.22, 42.16, 38.59, 37.38, 36.24, 27.74, 24.95, 23.87, 22.96, 14.58; ESI-HRMS: *m*/*z* 479.2417 [M + Na]^+^, calcd for C_27_H_36_NaO_6_, 479.2410.

##### (14α)-(2′-Methoxy)phenoxy-andrographolide (6a12)

White solid; mp 155.2 °C to 161.6 °C; 92% yield; ^1^H NMR (400 MHz, (CD_3_)_2_SO) *δ* 7.07–7.03 (m, 2H), 6.97 (d, *J* = 7.5 Hz, 1H), 6.93–6.87 (m, 1H), 6.82 (dd, *J* = 6.8, 5.7 Hz, 1H), 5.58 (d, *J* = 5.3 Hz, 1H), 5.04 (d, *J* = 4.8 Hz, 1H), 4.79 (s, 1H), 4.57 (dd, *J* = 10.7, 5.4 Hz, 1H), 4.52 (s, 1H), 4.36 (dd, *J* = 10.7, 1.2 Hz, 1H), 4.12 (dd, *J* = 7.4, 2.7 Hz, 1H), 3.81–3.74 (m, 1H), 3.76 (s, 3H), 3.22 (dd, *J* = 10.9, 7.7 Hz, 1H), 3.16 (dd, *J* = 10.4, 5.0 Hz, 1H), 2.36–2.26 (m, 2H), 2.21–2.11 (m, 1H), 1.94–1.81 (m, 2H), 1.70 (d, *J* = 12.9 Hz, 1H), 1.62–1.51 (m, 2H), 1.50–1.43 (m, 1H), 1.30 (m, 1H), 1.15–1.07 (m, 2H), 1.05 (s, 3H), 0.53 (s, 3H); ^13^C NMR (101 MHz, (CD_3_)_2_SO) *δ* 169.8, 151.2, 149.9, 148.0, 145.9, 126.0, 124.0, 121.2, 118.9, 113.1, 108.6, 78.8, 73.3, 71.7, 63.0, 56.0, 55.5, 54.8, 42.7, 38.9, 37.9, 36.9, 28.3, 24.9, 24.4, 23.5, 15.0; ESI-HRMS: *m*/*z* 479.2406 [M + Na]^+^, calcd for C_27_H_36_NaO_6_, 479.2410.

##### (14β)-(2′-Methoxy)phenoxy-andrographolide (6b12)

White solid; mp 175 °C to 178 °C; 88% yield; ^1^H NMR (400 MHz, CDCl_3_) *δ* 7.13–7.02 (m, 1H), 7.01–6.83 (m, 4H), 5.56 (d, *J* = 5.4 Hz, 1H), 4.81 (d, *J* = 1.8 Hz, 1H), 4.55–4.40 (m, 2H), 4.32 (s, 1H), 4.13 (t, *J* = 8.9 Hz, 1H), 3.92–3.81 (m, 3H), 3.47–3.35 (m, 1H), 3.30 (d, *J* = 11.1 Hz, 1H), 2.42–2.33 (m, 1H), 2.32–2.21 (m, 2H), 2.02–1.90 (m, 1H), 1.88–1.73 (m, 3H), 1.73–1.65 (m, 1H), 1.60–1.52 (m, 1H), 1.32–1.24 (m, 1H), 1.23 (s, 3H), 1.22–1.16 (m, 1H), 1.10 (td, *J* = 13.2, 4.2 Hz, 1H), 0.59 (s, 1H); ^13^C NMR (101 MHz, CDCl_3_) *δ* 169.77, 151.35, 150.68, 147.23, 145.29, 125.46, 124.33, 121.09, 119.97, 112.35, 108.12, 80.45, 73.42, 71.20, 64.14, 55.80, 55.69, 55.03, 42.87, 38.88, 37.69, 36.64, 28.22, 25.55, 23.73, 22.70, 15.10; ESI-HRMS: *m*/*z* 479.2400 [M + Na]^+^, calcd for C_27_H_36_NaO_6_, 479.2410.

##### (14β)-(3′-Methoxy)phenoxy-andrographolide (6b13)

White solid; mp 156 °C to 158 °C; 92% yield; ^1^H NMR (400 MHz, (CD_3_)_2_SO) *δ* 7.29–7.16 (m, 1H), 6.94 (dd, *J* = 7.2, 6.0 Hz, 1H), 6.64–6.49 (m, 3H), 5.79 (d, *J* = 5.3 Hz, 1H), 4.99 (d, *J* = 4.9 Hz, 1H), 4.81 (s, 1H), 4.66 (dd, *J* = 10.7, 5.4 Hz, 1H), 4.52 (s, 1H), 4.28 (dd, *J* = 10.7, 1.1 Hz, 1H), 4.10 (dd, *J* = 7.5, 2.8 Hz, 1H), 3.79–3.37 (m, 1H), 3.74 (s, 3H), 3.19 (dd, *J* = 10.8, 7.7 Hz, 1H), 3.05–2.98 (m, 1H), 2.43–2.35 (m, 1H), 2.33–2.22 (m, 2H), 1.92 (t, *J* = 10.2 Hz, 2H), 1.70 (d, *J* = 13.2 Hz, 1H), 1.47 (m, 3H), 1.36–1.21 (m, 1H), 1.11–1.05 (m, 1H), 1.02 (s, 3H), 0.94 (m, 1H), 0.55 (s, 3H); ^13^C NMR (101 MHz, (CD_3_)_2_SO) *δ* 169.09, 160.65, 157.69, 149.66, 147.70, 130.25, 125.73, 107.71, 107.55, 107.35, 102.01, 78.24, 71.14, 71.04, 62.52, 55.66, 55.16, 54.21, 42.13, 38.68, 37.41, 36.17, 27.75, 24.91, 23.88, 22.93, 14.56; ESI-HRMS: *m*/*z* 479.2400 [M + Na]^+^, calcd for C_27_H_36_NaO_6_, 479.2410.

##### (14β)-(Naphthyl-1′-oxy)andrographolide (6b14)

White solid; mp 172 °C to 175 °C; 88% yield; ^1^H NMR (400 MHz, (CD_3_)_2_SO) *δ* 8.07 (d, *J* = 8.1 Hz, 1H), 7.92 (d, *J* = 7.7 Hz, 1H), 7.62–7.41 (m, 4H), 7.14 (t, *J* = 7.2 Hz, 1H), 7.01 (d, *J* = 7.5 Hz, 1H), 5.99 (d, *J* = 5.2 Hz, 1H), 4.84 (s, 1H), 4.83–4.79 (m, 1H), 4.76 (d, *J* = 4.9 Hz, 1H), 4.62 (s, 1H), 4.44 (dd, *J* = 10.8, 1.0 Hz, 1H), 4.05–4.00 (m, 1H), 3.63 (dd, *J* = 11.0, 2.8 Hz, 1H), 3.07 (dd, *J* = 10.8, 7.6 Hz, 1H), 2.45–2.33 (m, 2H), 2.33–2.22 (m, 2H), 1.96–1.86 (m, 2H), 1.63–1.54 (m, 1H), 1.24 (m, 3H), 1.06 (m, 1H), 0.80 (s, 3H), 0.69 (dd, *J* = 12.6, 2.2 Hz, 1H), 0.58–0.47 (m, 1H), 0.43 (s, 3H); ^13^C NMR (101 MHz, (CD_3_)_2_SO) *δ* 169.13, 168.88, 151.95, 150.00, 147.65, 134.31, 127.60, 126.69, 126.08, 125.97, 125.58, 124.93, 121.86, 121.66, 121.00, 107.65, 106.45, 99.49, 77.83, 71.21, 62.40, 56.28, 53.62, 48.13, 41.83, 38.72, 38.61, 37.39, 35.72, 27.56, 24.92, 23.78, 22.59, 20.75, 20.75, 14.45; ESI-HRMS: *m*/*z* 499.2462 [M + Na]^+^, calcd for C_30_H_36_NaO_5_, 499.2460.

##### (14α)-(2′-Nitro-pyridinyl-3′-oxy)andrographolide (6a15)

White solid; mp 140 °C to 142 °C; 83% yield; ^1^H NMR (400 MHz, (CD_3_)_2_SO) *δ* 8.21 (dd, *J* = 4.5, 1.1 Hz, 1H), 8.02 (dd, *J* = 8.5, 1.0 Hz, 1H), 7.83 (dd, *J* = 8.5, 4.6 Hz, 1H), 6.93 (t, *J* = 6.0 Hz, 1H), 6.06 (d, *J* = 5.4 Hz, 1H), 5.02 (d, *J* = 4.9 Hz, 1H), 4.77 (s, 1H), 4.72 (dd, *J* = 11.2, 5.6 Hz, 1H), 4.46 (dd, *J* = 11.1, 1.3 Hz, 1H), 4.40 (s, 1H), 4.12 (dd, *J* = 7.4, 2.9 Hz, 1H), 3.79 (dd, *J* = 11.0, 2.8 Hz, 1H), 3.26–3.13 (m, 2H), 2.47–2.41 (m, 1H), 2.36–2.25 (m, 2H), 1.91 (m, 2H), 1.71 (m, 1H), 1.63–1.44 (m, 3H), 1.32 (m, 1H), 1.19–1.11 (m, 1H), 1.10–1.02 (m, 1H), 1.06 (s, 3H), 0.55 (s, 3H); ^13^C NMR (101 MHz, (CD_3_)_2_SO) *δ* 168.58, 151.60, 149.00, 147.58, 144.24, 140.35, 129.44, 126.35, 123.96, 108.04, 78.36, 73.08, 70.19, 62.54, 55.07, 54.28, 42.19, 38.44, 37.30, 36.38, 27.77, 24.62, 23.82, 23.02, 14.51; ESI-HRMS: *m*/*z* 495.2102 [M + Na]^+^, calcd for C_25_H_32_N_2_NaO_7_, 495.2107.

##### (14β)-(2′-Nitro-pyridinyl-3′-oxy)andrographolide (6b15)

White solid; mp 146 °C to 149 °C; 90% yield; ^1^H NMR (400 MHz, CD_3_OD) *δ* 8.18 (dd, *J* = 4.6, 1.2 Hz, 1H), 7.90 (dd, *J* = 8.5, 1.1 Hz, 1H), 7.75 (dd, *J* = 8.5, 4.6 Hz, 1H), 7.16 (td, *J* = 7.3, 1.5 Hz, 1H), 6.05 (d, *J* = 5.5 Hz, 1H), 4.90 (s, 1H), 4.73 (dd, *J* = 11.2, 5.6 Hz, 1H), 4.54 (s, 1H), 4.47 (dd, *J* = 11.2, 1.4 Hz, 1H), 4.07 (d, *J* = 11.1 Hz, 1H), 3.34 (d, *J* = 2.9 Hz, 1H), 3.28 (dd, *J* = 11.9, 4.1 Hz, 1H), 2.54 (m, 1H), 2.47–2.33 (m, 2H), 2.04 (m, 1H), 1.95 (m, 1H), 1.90–1.82 (m, 1H), 1.77–1.53 (m, 3H), 1.41–1.26 (m, 2H), 1.21 (s, 3H), 1.09–0.99 (m, 1H), 0.66 (s, 3H); ^13^C NMR (101 MHz, CD_3_OD) *δ* 170.88, 153.35, 151.17, 149.05, 145.93, 141.67, 130.25, 126.69, 125.80, 108.52, 80.74, 74.60, 72.00, 64.95, 57.59, 56.03, 43.64, 40.20, 38.89, 37.88, 28.93, 26.91, 25.20, 23.29, 15.47; ESI-HRMS: *m*/*z* 495.2101 [M + Na]^+^, calcd for C_25_H_32_N_2_NaO_7_, 495.2107.

##### (14α)-(Quinolyl-4′-oxy)andrographolide (6a16)

White solid; mp 102.6 °C to 104.8 °C; 83% yield; ^1^H NMR (400 MHz, (CD_3_)_2_SO) *δ* 8.75 (d, *J* = 5.2 Hz, 1H), 8.03 (d, *J* = 7.5 Hz, 1H), 7.95 (d, *J* = 8.3 Hz, 1H), 7.77–7.71 (m, 1H), 7.53 (t, *J* = 7.6 Hz, 1H), 7.05 (d, *J* = 5.3 Hz, 1H), 7.01 (t, *J* = 6.8 Hz, 1H), 6.08 (d, *J* = 5.3 Hz, 1H), 4.96 (d, *J* = 4.9 Hz, 1H), 4.84–4.76 (m, 2H), 4.55 (s, 1H), 4.41 (d, *J* = 11.0 Hz, 1H), 4.05 (dd, *J* = 7.4, 2.8 Hz, 1H), 3.68 (dd, *J* = 11.0, 2.8 Hz, 1H), 3.13 (dd, *J* = 11.1, 7.6 Hz, 1H), 3.10–3.02 (m, 1H), 2.43–2.34 (m, 1H), 2.25 (d, *J* = 11.9 Hz, 1H), 1.94 (d, *J* = 11.4 Hz, 1H), 1.89–1.83 (m, 1H), 1.63 (d, *J* = 13.6 Hz, 1H), 1.49–1.41 (m, 1H), 1.40–1.35 (m, 2H), 0.98 (s, 3H), 0.85–0.67 (m, 3H), 0.64–0.56 (m, 1H), 0.40 (s, 3H); ^13^C NMR (101 MHz, (CD_3_)_2_SO) *δ* 169.4, 159.3, 151.8, 151.6, 149.4, 148.3, 130.5, 129.2, 126.5, 124.9, 121.8, 121.1, 108.5, 103.0, 78.8, 72.1, 71.1, 63.0, 55.5, 54.7, 42.6, 38.9, 37.7, 36.8, 28.2, 25.3, 24.2, 23.5, 14.9; ESI-HRMS: *m*/*z* 478.2587 [M + H]^+^, calcd for C_29_H_36_NO_5_, 478.2593.

##### (14β)-(Quinolyl-4′-oxy)andrographolide (6b16)

White solid; mp 175 °C to 177 °C; 88% yield; ^1^H NMR (400 MHz, (CD_3_)_2_SO) *δ* 8.78 (d, *J* = 5.2 Hz, 1H), 8.07 (d, *J* = 7.6 Hz, 1H), 7.99 (d, *J* = 8.1 Hz, 1H), 7.81–7.74 (m, 1H), 7.21 (t, *J* = 7.2 Hz, 1H), 7.07 (d, *J* = 5.3 Hz, 1H), 6.13 (d, *J* = 5.2 Hz, 1H), 4.84 (s, 1H), 4.78 (d, *J* = 4.8 Hz, 1H), 4.66 (s, 1H), 4.48 (d, *J* = 11.1 Hz, 1H), 4.04–3.97 (m, 1H), 3.61 (dd, *J* = 11.0, 2.9 Hz, 1H), 3.06 (dd, *J* = 10.9, 7.6 Hz, 1H), 2.44 (d, *J* = 8.6 Hz, 1H), 2.31–2.24 (m, 3H), 1.89 (d, *J* = 9.3 Hz, 2H), 1.65–1.52 (m, 2H), 1.31–1.24 (m, 2H), 1.24–1.13 (m, 2H), 1.05–0.94 (m, 1H), 0.78 (s, 3H), 0.71–0.59 (m, 2H), 0.44 (s, 3H); ^13^C NMR (101 MHz, (CD_3_)_2_SO) *δ* 169.0, 166.5, 153.0, 147.9, 147.7, 145.9, 135.4, 129.6, 124.5, 123.8, 121.2, 120.7, 108.4, 104.2, 78.6, 74.8, 70.6, 62.8, 56.7, 54.6, 42.5, 39.3, 37.9, 36.6, 28.0, 25.7, 24.3, 23.3, 15.0; ESI-HRMS: *m*/*z* 478.2588 [M + H]^+^, calcd for C_29_H_36_NO_5_, 478.2593.

##### (14α)-(Quinolyl-8′-oxy)andrographolide (6a17)

White solid; mp 151 °C to 153 °C; 79% yield; ^1^H NMR (400 MHz, (CD_3_)_2_SO) *δ* 8.89 (dd, *J* = 4.1, 1.7 Hz, 1H), 8.39 (dd, *J* = 8.4, 1.7 Hz, 1H), 7.69 (dd, *J* = 8.2, 0.9 Hz, 1H), 7.62–7.51 (m, 2H), 7.28 (dd, *J* = 7.6, 1.0 Hz, 1H), 6.90 (t, *J* = 6.3 Hz, 1H), 6.06 (d, *J* = 5.3 Hz, 1H), 5.00 (d, *J* = 4.9 Hz, 1H), 4.76–4.68 (m, 2H), 4.56–4.49 (m, 2H), 4.09 (dd, *J* = 7.5, 2.8 Hz, 1H), 3.71 (dd, *J* = 11.0, 2.7 Hz, 1H), 3.19–3.00 (m, 2H), 2.27–2.18 (m, 2H), 2.17–2.07 (m, 1H), 1.81 (m, 2H), 1.64 (m, 1H), 1.40 (m, 2H), 1.26–1.20 (m, 2H), 1.15 (m, 1H), 1.03 (m, 1H), 1.00 (s, 3H), 0.36 (s, 3H); ^13^C NMR (101 MHz, (CD_3_)_2_SO) *δ* 169.9, 152.7, 150.1, 149.9, 147.8, 141.3, 136.7, 130.0, 127.2, 126.1, 122.9, 122.5, 116.3, 108.6, 78.7, 74.0, 71.8, 63.0, 55.4, 54.6, 42.6, 38.8, 37.8, 36.6, 28.2, 25.1, 24.3, 23.4, 14.8; ESI-HRMS: *m*/*z* 478.2588 [M + H]^+^, calcd for C_29_H_36_NO_5_, 478.2593.

##### (14β)-(Quinolyl-8′-oxy)andrographolide (6b17)

White solid; mp 134 °C to 136 °C; 76% yield; ^1^H NMR (400 MHz, CD_3_OD) *δ* 8.88 (dd, *J* = 4.3, 1.7 Hz, 1H), 8.40 (dd, *J* = 8.3, 1.7 Hz, 1H), 7.67–7.58 (m, 3H), 7.31–7.22 (m, 2H), 6.09 (d, *J* = 5.5 Hz, 1H), 4.89 (d, *J* = 1.0 Hz, 1H), 4.83–4.79 (m, 1H), 4.62–4.55 (m, 2H), 3.91 (d, *J* = 11.1 Hz, 1H), 3.63 (q, *J* = 7.0 Hz, 1H), 3.21 (d, *J* = 10.3 Hz, 1H), 2.52 (dd, *J* = 11.9, 4.2 Hz, 1H), 2.48–2.34 (m, 2H), 2.32–2.21 (m, 1H), 2.06 (m, 2H), 1.77–1.68 (m, 1H), 1.48–1.34 (m, 1H), 1.30–1.24 (m, 1H), 1.20 (m, 2H), 1.09 (m, 1H), 0.99 (s, 3H), 0.84 (m, 1H), 0.58 (m, 1H), 0.49 (s, 3H); ^13^C NMR (101 MHz, CD_3_OD) *δ* 171.84, 153.59, 151.90, 150.48, 149.14, 141.29, 138.32, 131.55, 128.29, 127.54, 123.50, 122.60, 112.92, 108.19, 80.50, 73.65, 73.10, 64.79, 57.59, 55.73, 43.35, 40.01, 38.80, 37.39, 28.65, 26.58, 25.10, 23.18, 15.28; ESI-HRMS: *m*/*z* 500.2407 [M + Na]^+^, calcd for C_29_H_35_NNaO_5_, 500.2413.

#### 4.2.3. Preparation of compound 7b1

To a solution of 0.48 g of 6b17 (1.0 mmol) in 15 mL of dichloromethane, 0.15 g of trimethylamine (1.5 mmol) in 2 mL of dichloromethane was added; the mixture was then treated with 0.094 g of acetyl chloride (1.2 mmol) in 2 mL of dichloromethane at 0 °C for 30 min. After removing the volatile solvents by distillation, the residue was dissolved in ethyl acetate and washed with saturated NaHCO_3_ solution and brine. The organic phase was dried over anhydrous Na_2_SO_4_, filtered, and evaporated to dryness; the residue was purified by silica gel column chromatography (petroleum ether/ethyl acetate 1/1) to afford 0.47 g of (14β)-(quinolyl-8′-oxy)-19-acetoxy andrographolide (7b1). 7b1: white solid; mp 74 °C to 77 °C; 90% yield; ^1^H NMR (400 MHz, CD_3_Cl) *δ* 9.11 (s, 1H), 8.42 (d, *J* = 7.9 Hz, 1H), 7.64 (dd, *J* = 23.1, 7.8 Hz, 3H), 7.16 (q, *J* = 7.0 Hz, 2H), 6.08 (s, 1H), 4.83 (s, 1H), 4.76–4.60 (m, 2H), 4.38 (s, 1H), 4.22 (d, *J* = 11.6 Hz, 1H), 4.02 (d, *J* = 11.7 Hz, 1H), 3.00 (t, *J* = 8.0 Hz, 1H), 2.48–2.33 (m, 2H), 2.28–2.17 (m, 1H), 2.03 (s, 3H), 1.99–1.89 (m, 2H), 1.82 (s, 1H), 1.47–1.20 (m, 5H), 1.15 (dd, *J* = 12.6, 2.7 Hz, 1H), 1.08 (s, 3H), 0.94 (q, *J* = 11.7, 11.2 Hz, 1H), 0.47 (s, 3H); ^13^C NMR (101 MHz, (CD_3_)_2_SO) *δ* 170.8, 169.8, 152.7, 150.5, 149.9, 148.4, 140.7, 136.6, 129.9, 127.2, 126.7, 122.6, 122.0, 113.3, 108.0, 76.6, 72.8, 71.7, 65.4, 56.2, 53.68, 4.76, 39.2, 37.9, 36.3, 27.7, 25.35, 25.1, 23.0, 21.4, 14.2; ESI-HRMS: *m*/*z* 520.2691 [M + H]^+^, calcd for C_31_H_38_NO_6_, 520.2699.

#### 4.2.4. Preparation of compound 7b2

At room temperature, to a solution of 0.4 g (0.84 mmol) 6b17 in 18.0 mL dry dichloromethane (DCM), 0.66 mL (5.0 mmol) triethylamine (TEA) and then 0.64 g (4.2 mmol) TBSCl in 2.0 mL dry DCM were added carefully. After the reaction was complete in 40 min, the reaction mixture was treated with sol. sat. NH_4_Cl. The organic phase was extracted with ethyl acetate and washed with sol. sat. NaHCO_3_ and brine, then dried over anhydrous Na_2_SO_4_. The residue was filtered and chromatographed on silica gel (petroleum ether/ethyl acetate) to provide 0.44 g of (14β)-(quinolyl-8′-oxy)-19-(*t*-butyldimethylsilyloxy)andrographolide (7b2). 7b2: white solid; mp 149 °C to 151 °C; 89% yield; ^1^H NMR (400 MHz, (CD_3_)_2_SO) *δ* 8.88 (dd, *J* = 4.1, 1.7 Hz, 1H), 8.37 (dd, *J* = 8.3, 1.6 Hz, 1H), 7.64 (d, *J* = 7.5 Hz, 1H), 7.61–7.52 (m, 2H), 7.23 (d, *J* = 6.9 Hz, 1H), 7.06 (t, *J* = 6.9 Hz, 1H), 6.06 (d, *J* = 5.1 Hz, 1H), 4.77 (m, 2H), 4.54–4.45 (m, 2H), 4.22 (d, *J* = 5.2 Hz, 1H), 3.58 (d, *J* = 10.5 Hz, 1H), 3.47 (d, *J* = 10.5 Hz, 1H), 2.36–2.14 (m, 4H), 2.00 (m, 1H), 1.87 (m, 1H), 1.62 (m, 1H), 1.48 (m, 1H), 1.22 (m, 2H), 0.97 (m, 1H), 0.80 (s, 9H), 0.77 (s, 3H), 0.69–0.63 (m, 1H), 0.56 (m, 1H), 0.47 (s, 3H), −0.07 (d, *J* = 2.3 Hz, 6H); ^13^C NMR (101 MHz, (CD_3_)_2_SO) *δ* 169.27, 152.18, 150.02, 149.36, 148.25, 140.20, 136.03, 129.42, 126.68, 126.20, 122.06, 121.40, 112.65, 107.18, 76.67, 72.24, 71.24, 64.12, 55.88, 53.68, 42.24, 38.75, 37.78, 36.08, 27.47, 25.60 (3C), 25.30, 24.88, 22.69, 17.73, 13.81, −5.78, −5.81; ESI-HRMS: *m*/*z* 592.3453 [M + H]^+^, calcd for C_35_H_50_NO_5_Si, 592.3458.

#### 4.2.5. Preparation of compound 8b1 or 8b2

At room temperature and under protection from light, 0.6 mmol of 7b1 or 7b2 was dissolved in 8.0 mL dry DCM and treated with 0.50 g (1.17 mmol) Dess–Martin periodinane. The oxidation reaction was complete in 3 h, and the mixture was diluted with 100 mL ethyl acetate and then treated with sol. sat. Na_2_S_2_O_3_. The organic phase was washed with sol. sat. NaHCO_3_, brine and distilled water. After drying over anhydrous Na_2_SO_4_, the solvent was evaporated and the residue was purified by silica gel chromatography (petroleum ether/ethyl acetate) to afford (14β)-(quinolyl-8′-oxy)-3-keto-19-acetoxy andrographolide (8b1) or (14β)-(quinolyl-8′-oxy)-3-keto-19-(*t*-butyldimethyl silyloxy)andrographolide (8b2).

##### 8b1

White solid; mp 56 °C to 59 °C; 90% yield; ^1^H NMR (400 MHz, (CD_3_)_2_SO) *δ* 8.87 (d, *J* = 4.2 Hz, 1H), 8.37 (d, *J* = 8.3 Hz, 1H), 7.71–7.47 (m, 3H), 7.25 (d, *J* = 7.6 Hz, 1H), 7.02 (t, *J* = 7.2 Hz, 1H), 6.11 (d, *J* = 5.3 Hz, 1H), 4.88 (s, 1H), 4.78 (dd, *J* = 10.9, 5.5 Hz, 1H), 4.60 (s, 1H), 4.50 (d, *J* = 10.9 Hz, 1H), 4.35 (d, *J* = 11.3 Hz, 1H), 3.78 (d, *J* = 11.3 Hz, 1H), 2.31 (q, *J* = 9.3, 7.7 Hz, 3H), 2.17–2.08 (m, 1H), 2.04–1.91 (m, 2H), 1.87 (s, 3H), 1.75–1.53 (m, 3H), 1.35 (s, 1H), 1.27–1.11 (m, 3H), 1.08–0.94 (m, 2H), 0.86 (s, 3H); ^13^C NMR (101 MHz, (CD_3_)_2_SO) *δ* 211.7, 170.5, 169.7, 152.6, 150.0, 149.9, 147.4, 140.8, 136.6, 129.9, 127.2, 126.7, 122.5, 122.2, 113.9, 109.1, 73.1, 71.7, 65.8, 55.5, 54.9, 51.6, 38.9, 37.2, 37.0, 35.0, 25.7, 24.7, 20.9, 20.7, 14.3; ESI-HRMS: *m*/*z* 518.2536 [M + H]^+^, calcd for C_31_H_36_NO_6_, 518.25426.

##### 8b2

White solid; mp 125 °C to 127 °C; 86% yield; ^1^H NMR (400 MHz, (CD_3_)_2_SO) *δ* 8.85 (dd, *J* = 4.1, 1.7 Hz, 1H), 8.36 (dd, *J* = 8.4, 1.7 Hz, 1H), 7.64 (d, *J* = 7.4 Hz, 1H), 7.59–7.50 (m, 2H), 7.24 (d, *J* = 6.8 Hz, 1H), 7.02 (t, *J* = 6.8 Hz, 1H), 6.10 (d, *J* = 5.3 Hz, 1H), 4.86 (s, 1H), 4.77 (dd, *J* = 10.8, 5.5 Hz, 1H), 4.57 (s, 1H), 4.52–4.45 (m, 1H), 3.67 (d, *J* = 10.1 Hz, 1H), 3.36 (d, *J* = 10.3 Hz, 1H), 2.28 (m, 4H), 2.09 (m, 1H), 1.97 (m, 1H), 1.76–1.68 (m, 1H), 1.60 (m, 1H), 1.57–1.49 (m, 1H), 1.40–1.33 (m, 1H), 1.30–1.27 (m, 1H), 0.97–0.90 (m, 1H), 0.79 (s, 3H), 0.75 (s, 9H), 0.63 (s, 3H), −0.09 (d, *J* = 1.7 Hz, 6H); ^13^C NMR (101 MHz, (CD_3_)_2_SO) *δ* 212.4, 169.7, 152.6, 150.1, 149.8, 147.9, 140.8, 136.6, 129.9, 127.2, 126.6, 122.5, 122.2, 113.9, 108.9, 73.1, 71.7, 65.9, 55.4, 55.0, 53.5, 38.9, 37.4, 36.9, 35.6, 26.8, 26.1, 26.0, 25.8, 24.9, 21.3, 18.2, 14.4, −5.3, −5.4; ESI-HRMS: *m*/*z* 590.3297 [M + H]^+^, calcd for C_35_H_48_NO_5_Si, 590.3302.

#### 4.2.6. Preparation of compound 9b from 8b1 or 8b2

0.26 g (0.50 mmol) of 8b1 was dissolved in 5 mL methanol and then treated with 0.17 g (1.0 mmol) of *p*-TSA at 40 °C for 8 h. The mixture was diluted with ethyl acetate and washed with sat. NaHCO_3_ solution and brine; the organic phase was dried over anhydrous Na_2_SO_4_, filtered, and then concentrated under reduced pressure. The residue was purified by silica gel column chromatography to afford (14β)-(quinolyl-8′-oxy)-3-keto andrographolide (9b) in 80% yield.

A solution of 0.20 g (0.34 mmol) 8b2 in 4.0 mL dry DCM was cooled to −20 °C and treated with 4.0 mL trifluoroacetic acid (TFA) for 20 min. The reaction mixture was diluted with ethyl acetate and carefully treated with sol. sat. NaHCO_3_. The organic phase was washed with sol. sat. NaHCO_3_ and brine, then dried over anhydrous Na_2_SO_4_. The residue was filtered, dried and purified by silica gel column chromatography (petroleum ether/ethyl acetate) to afford (14β)-(quinolyl-8′-oxy)-3-keto andrographolide (9b) in 81% yield. 9b: white solid; mp 141 °C to 143 °C; ^1^H NMR (400 MHz, (CD_3_)_2_SO) *δ* 8.85 (dd, *J* = 4.1, 1.7 Hz, 1H), 8.36 (dd, *J* = 8.3, 1.7 Hz, 1H), 7.64 (d, *J* = 7.5 Hz, 1H), 7.60–7.50 (m, 2H), 7.24 (d, *J* = 6.8 Hz, 1H), 7.01 (dd, *J* = 7.3, 6.1 Hz, 1H), 6.10 (d, *J* = 5.3 Hz, 1H), 4.85 (s, 1H), 4.77 (dd, *J* = 10.8, 5.5 Hz, 1H), 4.56 (s, 1H), 4.49 (dd, *J* = 10.8, 1.1 Hz, 1H), 4.43 (t, *J* = 5.4 Hz, 1H), 3.66 (dd, *J* = 10.9, 5.7 Hz, 1H), 3.16 (dd, *J* = 10.9, 5.2 Hz, 1H), 2.42 (m, 1H), 2.28 (m, 3H), 2.06 (m, 1H), 2.00–1.90 (m, 1H), 1.67 (m, 1H), 1.62–1.47 (m, 2H), 1.34 (m, 1H), 1.25–1.18 (m, 1H), 0.99–0.87 (m, 1H), 0.80 (s, 3H), 0.65 (s, 3H); ^13^C NMR (101 MHz, (CD_3_)_2_SO) *δ* 212.64, 169.30, 152.13, 149.76, 149.45, 147.42, 140.31, 136.18, 129.50, 126.72, 126.19, 122.12, 121.79, 113.51, 108.43, 72.64, 71.29, 63.84, 55.38, 54.75, 53.68, 38.55, 37.11, 36.98, 35.21, 25.32, 24.24, 20.10, 14.15; ESI-HRMS: *m*/*z* 476.2431 [M + H]^+^, calcd for C_29_H_34_NO_5_, 476.2437.

#### 4.2.7. Antibacterial evaluation

The tested compound or ciprofloxacin (positive drug) was dissolved in DMSO and diluted with LB liquid medium into a stock solution containing 20% DMSO before use; the stock solution of the carrier was 20% DMSO in LB liquid medium. All the experiments, including blank controls, were performed in triplicate.

Bacteriostatic screening was performed by applying a modified protocol of the National Committee on Clinical Laboratory Standards (NCCLS).^29^ Briefly, *E. coli*, *S. aureus* and *E. faecalis* cells (American Type Culture Collection, ATCC) were grown in LB plate medium for one passage, then seeded in LB liquid medium and cultured at 37 °C for 12 h. After being diluted 1000 times, 190 μL diluted cells were aliquoted to 96-well plates; then, 10 μL stock solution of carrier or a gradient amount of the tested compound or the positive compound, ciprofloxacin, were added; the final concentration of DMSO was 1% in all wells. The blank controls contained 1% DMSO and LB medium but no bacterial cells. The cultures were incubated at 37 °C for 24 h, and the growth data were expressed as OD_630_; the IC_50_ values were calculated based on growth data *versus* test concentration.

#### 4.2.8. Activity (EC_50_) against signaling pathways and cytotoxicity (CC_50_) of testing compounds in AD-293 cells

Cells from the AD-293 cell line, a derivative of the HEK293 cell line with improved adherence and plaque formation properties, were purchased from Stratagene (La Jolla, CA, USA). The protocols were adopted and modified from our previous report.^25^ All compounds were dissolved in DMSO at 10 mM as a stock solution. The final concentration of DMSO was 0.1% in the culture medium. AD-293 cells bearing luciferase reporters with promoter regions of IL-6/STAT3 and TNF-α/NF-κB in pGL4.20 vector (Promega) were maintained in DMEM high glucose medium supplemented with 10% FBS and 1% penicillin streptomycin in the presence of 1 μg mL^−1^ puromycin. AD-293 cells overexpressing TLR4 and stably transfected with NF-κB reporter were maintained in DMEM high glucose medium in the presence of 1 μg mL^−1^ puromycin and 10 μg mL^−1^ blasticidin. All cells were maintained in a humidified incubator at 37 °C in 95% air and 5% CO_2_.

##### Cytotoxicity of testing compounds

AD-293 cells were plated in a 96-well plate at a concentration of 1.0 × 10^5^ cells per well overnight to allow cell attachment. Working solutions of the tested compounds, the positive drug, DCB-3503 ^36^ of a cryptopleurine analog, or the DMSO vehicle as a control were dispensed appropriately into the partitioned 96-well plates, which were then incubated for another 24 h. Then, the medium was discarded and the cells were incubated for 4 h at 37 °C in MTT solution (final concentration 0.5 mg mL^−1^). The solution was then replaced with 100 μL DMSO to dissolve the violet formazan crystals in the intact cells. Cell growth was assessed by MTT according to the manufacturer's protocol. The absorbance was measured at 570 nm as the reference wavelength. The cytotoxicity as the CC_50_ value (concentration of 50% cell growth/viability inhibition) was calculated based on the percentage of cell viability data compared to the control group. Each concentration was repeated 3 times independently.

##### Signaling pathway reporter assay

Reporter cells were treated with 50 ng mL^−1^ TNF-α to stimulate the NF-κB signaling pathway, 1 μg mL^−1^ LPS to stimulate the TLR4/NF-κB signaling pathway, and 2.5 μg mL^−1^ IL-6 to stimulate the IL-6/STAT3 signaling pathway. The medium was removed at the end of the treatment, cell extracts were prepared, and the luciferase activity was measured using a Luciferase assay kit (Promega) according to the manufacturer's instructions. EC_50_ was defined as the concentration of drug that inhibited stimulator-triggered luciferase reporter activation by 50% after continuous drug exposure for 4 (TNF-α/NF-κB) or 16 (IL-6/STAT3 and TLR4/NF-κB) hours. Each concentration was repeated 3 times independently.

## Author contributions

GCZ, DW and YW conceived and supervised the project, analyzed the data and wrote the paper. GCZ, FL, DS, XN, ZL and DW designed the andrographolide derivatives; FL, DS, XN, ZL and DW conducted the syntheses of andrographolide derivatives. YW, XML, SRC, QZ, and YTW designed the biological experiments. XML, SRC and QZ conducted the biology experiments. All authors have read and approved the final manuscript.

## Conflicts of interest

There are no conflicts to declare.

## Supplementary Material

RA-008-C8RA01063C-s001
