# Supplementary material for: Discovery and preliminary SAR of 14-aryloxy-andrographolide derivatives as antibacterial agents with immunosuppressant activity
Source: RSC Adv. 2018 Mar 6;8(17):9440–56. doi: 10.1039/c8ra01063c (PMC9078697; doi:10.1039/c8ra01063c)

Electronic Supplementary Material (ESI) for RSC Advances.
This journal is © The Royal Society of Chemistry 2018

Discovery and preliminary SAR of 14-aryloxy-andrographolide derivatives as antibacterial agents with immunosuppressant activity

Feng Li,‡[a] Xiao-Min Li,‡[b] Dekuan Sheng,[a] Shao-Ru Chen,[b] Xin Nie,[a] Zhuyun Liu,§[a] Decai Wang,*[a] Qi Zhao,[c] Yitao Wang,[b] Ying Wang,*[b] Guo-Chun Zhou*[a]

[a] *School of Pharmaceutical Sciences, Nanjing Tech University, Nanjing 211816, PR China*

[b] *State Key Laboratory of Quality Research in Chinese Medicine and Institute of Chinese Medical Sciences, University of Macau, Avenida da Universidade, Taipa, Macao SAR, PR China*

[c] *Faculty of Health Sciences, University of Macau, Avenida da Universidade, Taipa, Macao SAR, PR China*

§ *Current address: Department of Pharmaceutical and Chemical Engineering, Taizhou Polytechnic College, Taizhou 225300, Jiangsu, PR China*

**Corresponding authors. Tel: 86-25-58139415, E-mail: gczhou@njtech.edu.cn (gcz), or emilyywang@umac.mo (yw), or dcwang@njtech.edu.cn (dw).*

‡ These authors contributed equally to this work.

Contents

^{1}H NMR and ^{13}C NMR spectra of **5a** and **5b**------------------------------pp2-29
^{1}H NMR and ^{13}C NMR spectra of **6a** and **6b**------------------------------pp30-57
^{1}H NMR and ^{13}C NMR spectra of 7b, 8b and 9b------------------------------pp58-62

^{1}H NMR of **5a1**

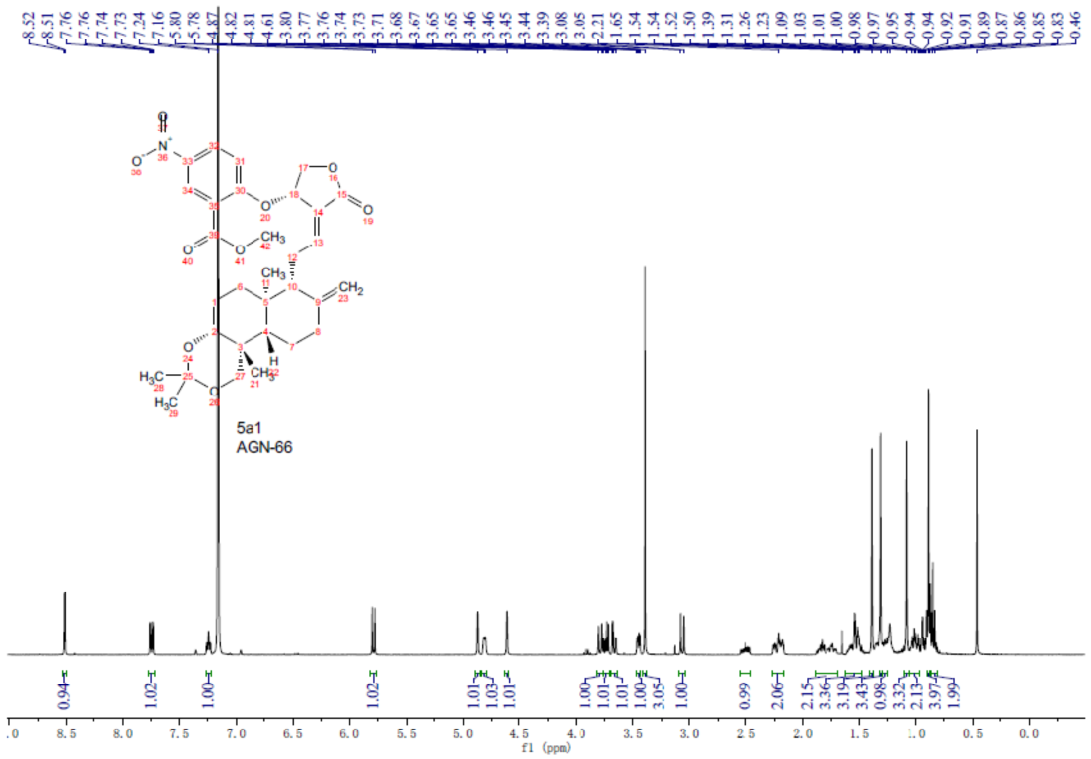

^{13}C NMR of **5a1**

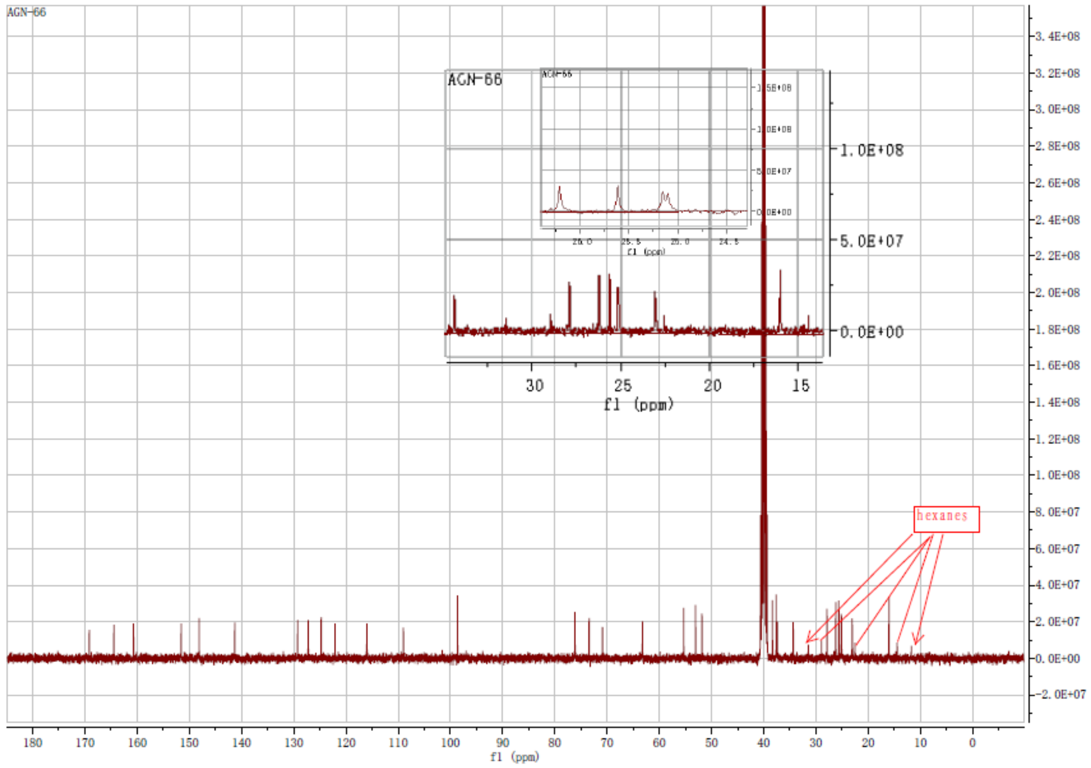

^{1}H NMR of **5b1**

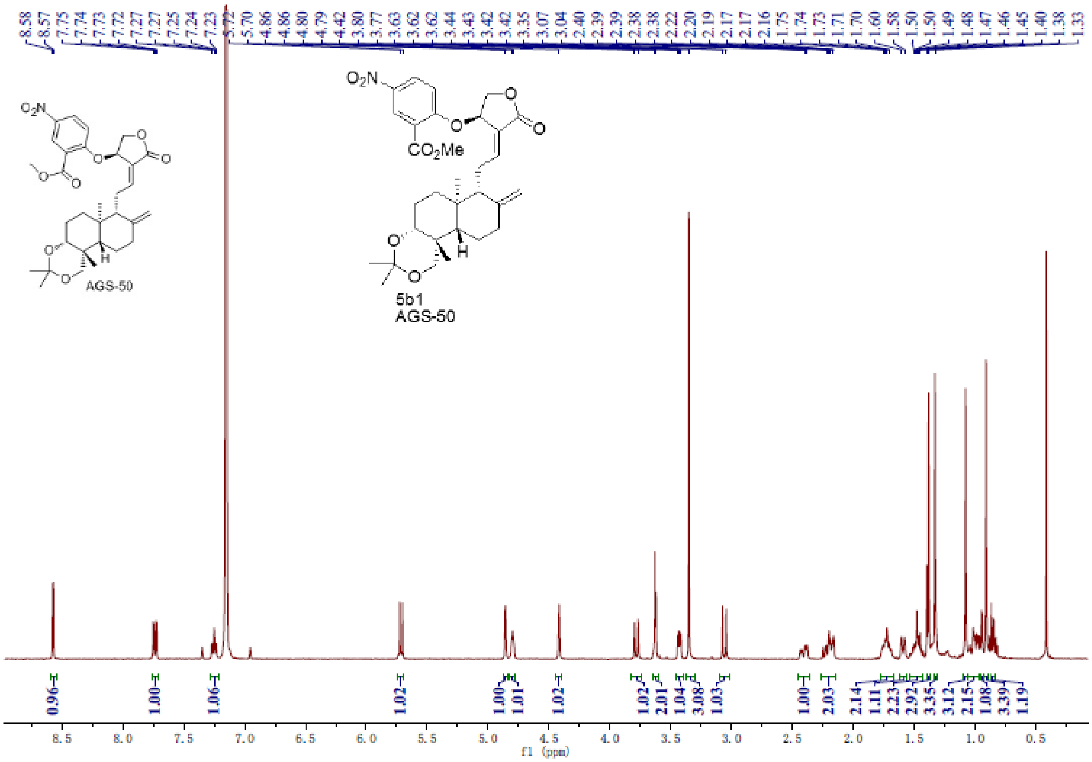

^{13}C NMR of **5b1**

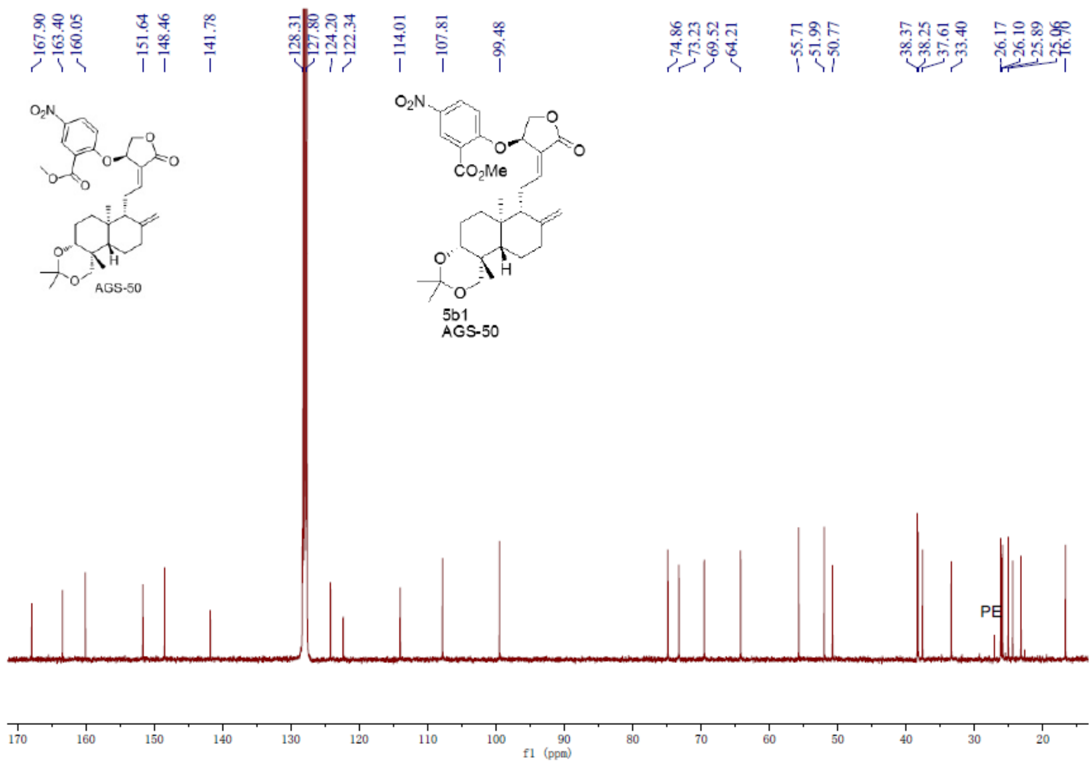

^{1}H NMR of **5a2**

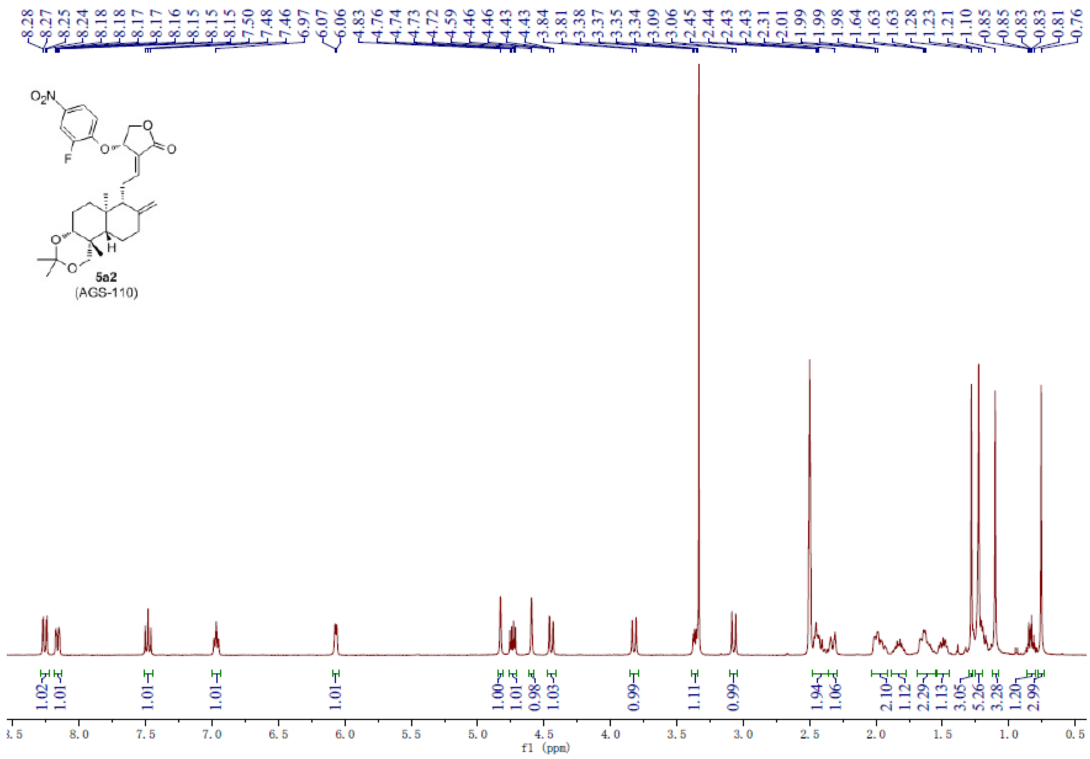

^{13}C NMR of **5a2**

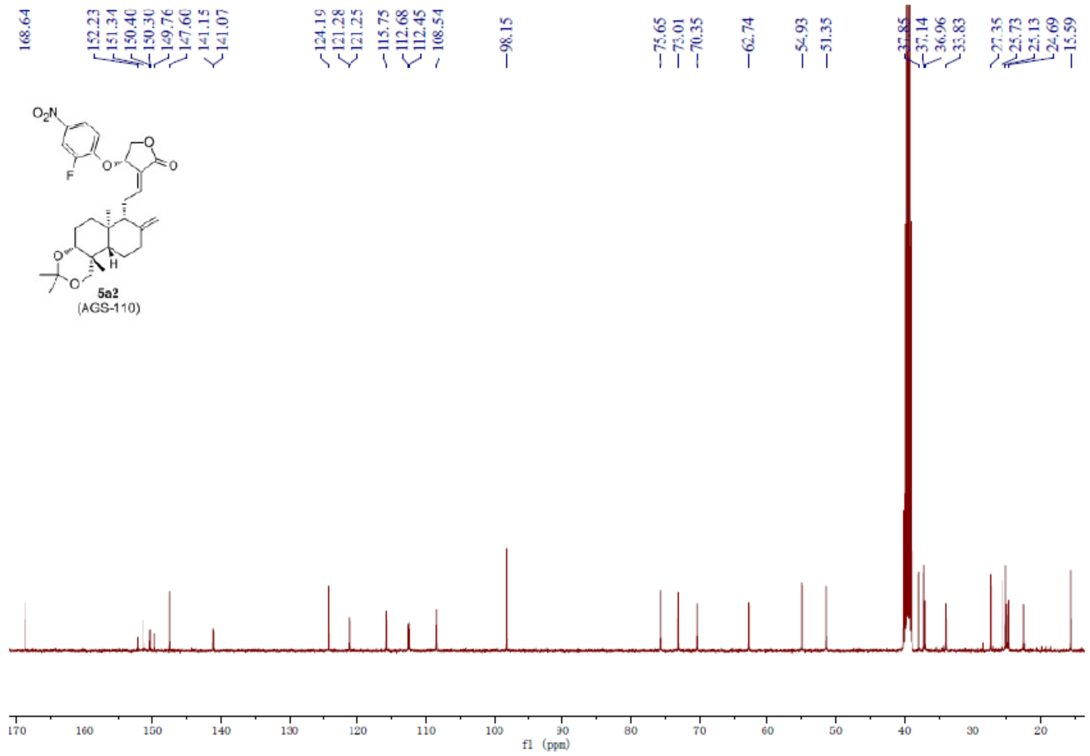

^{1}H NMR of **5b2**

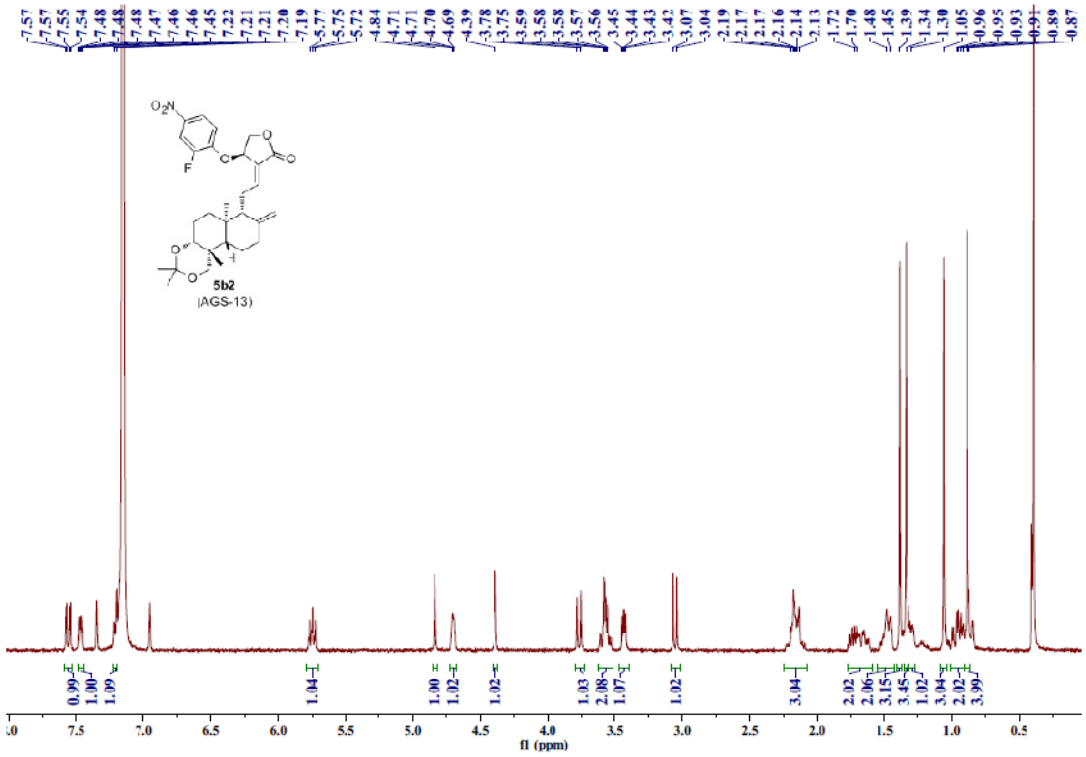

^{13}C NMR of **5b2**

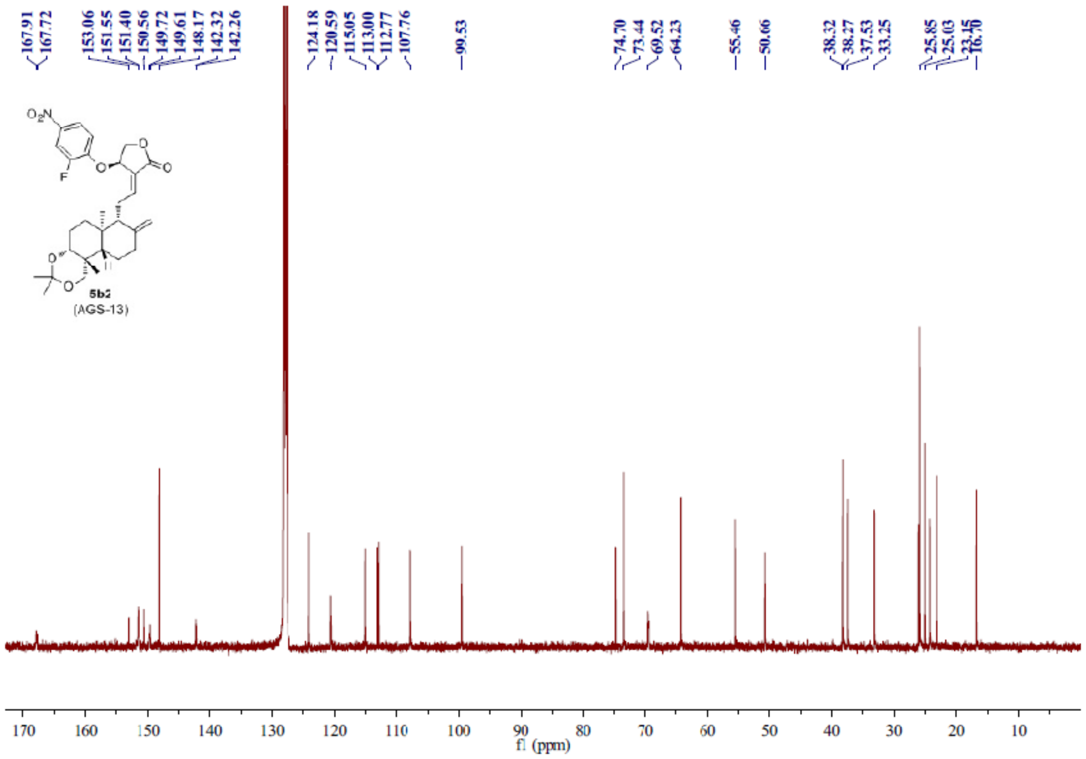

^{1}H NMR of **5a3**

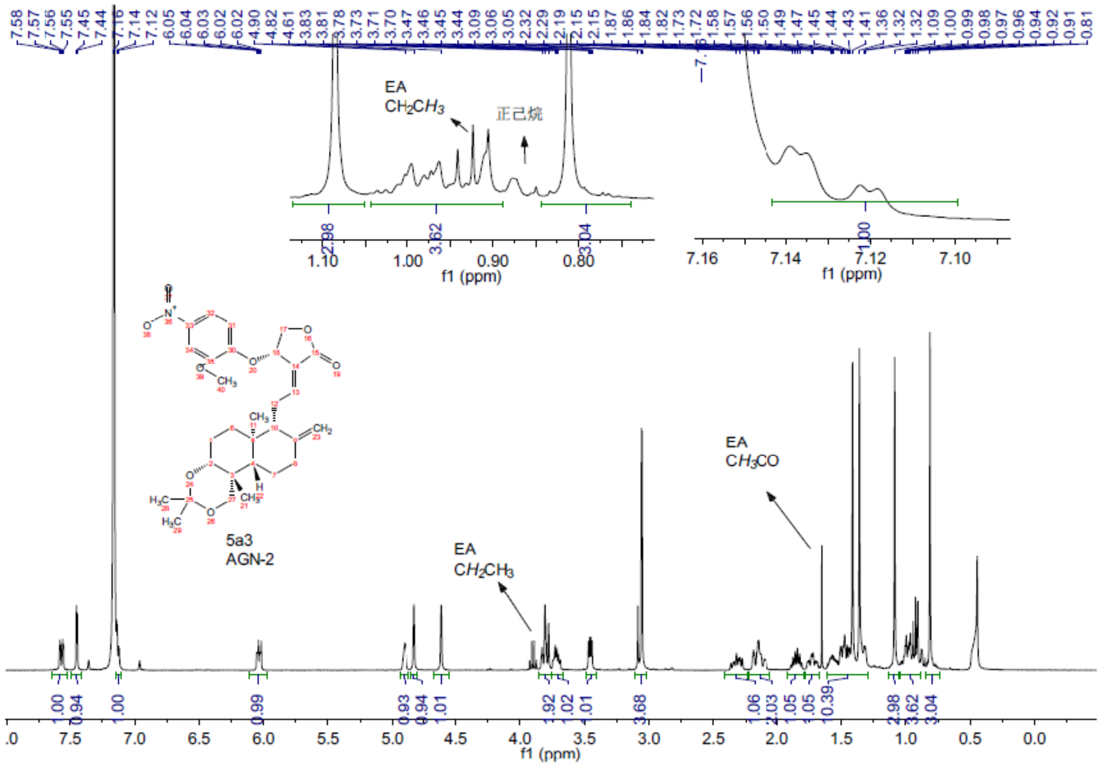

^{13}C NMR of **5a3**

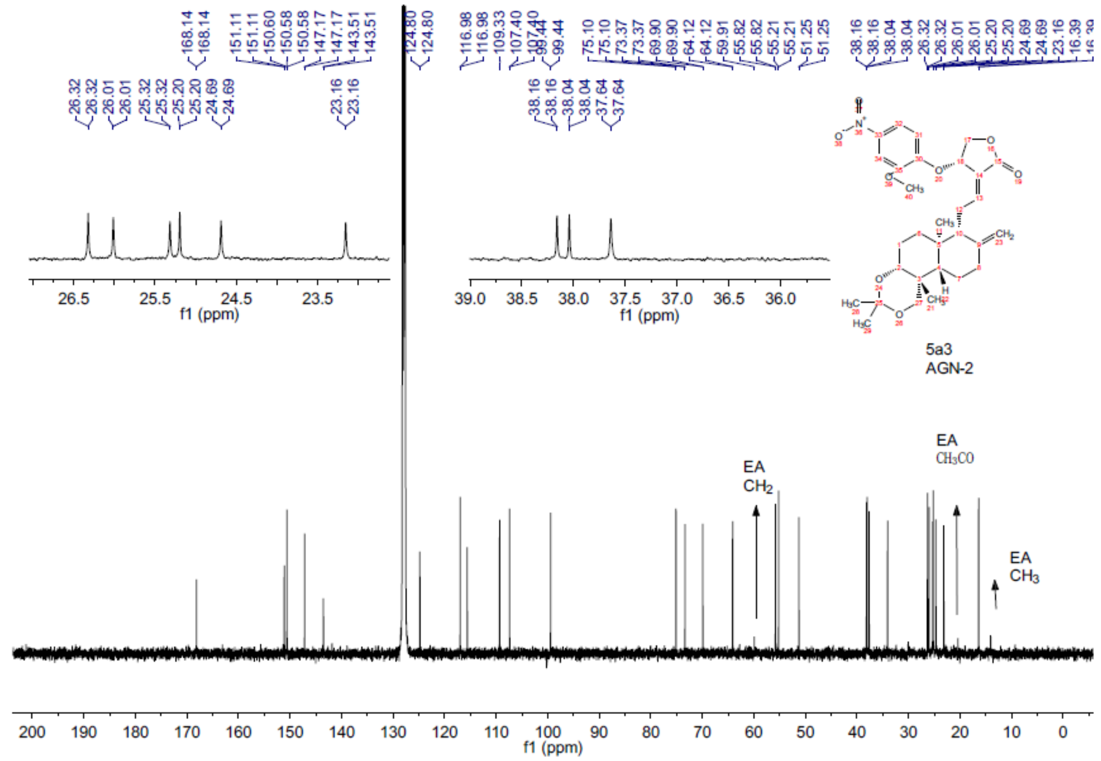

^{1}H NMR of **5b3**

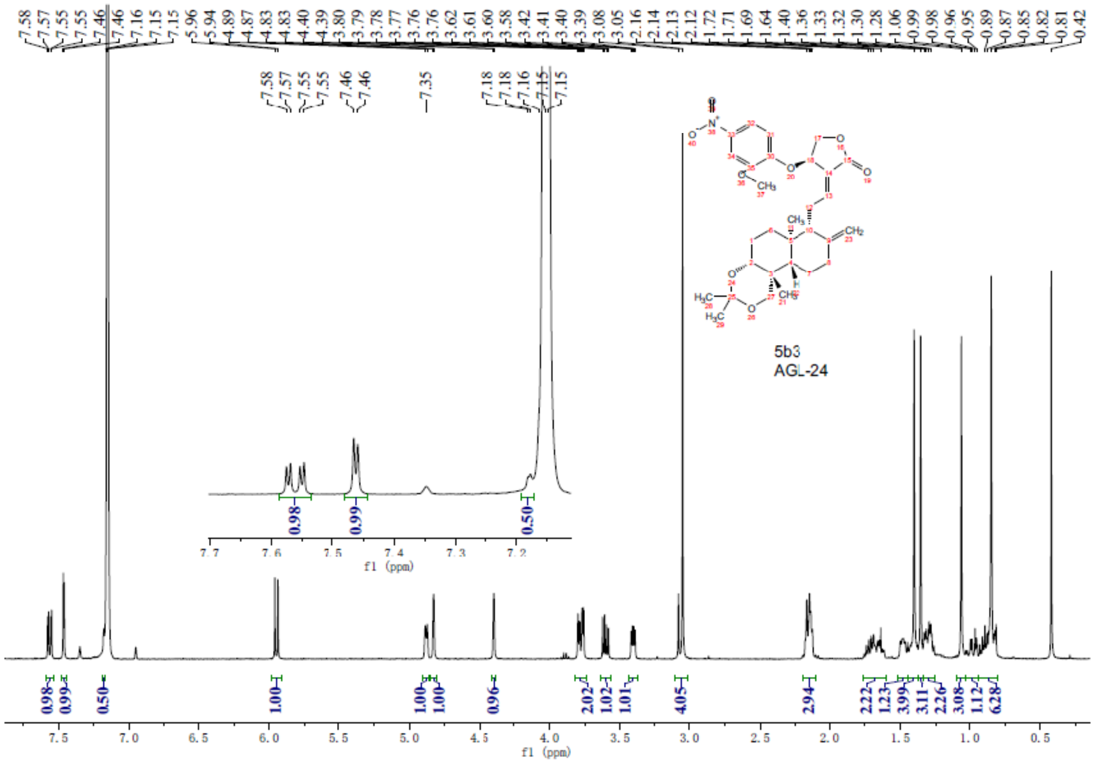

^{13}C NMR of **5b3**

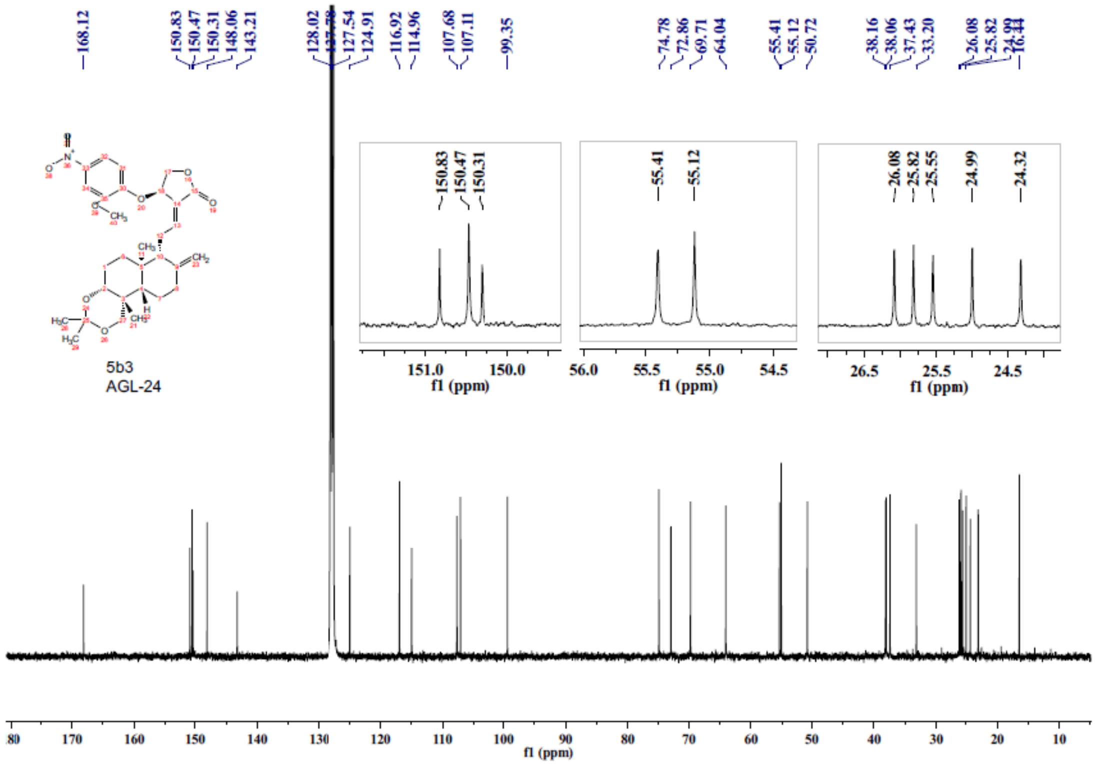

^{1}H NMR of **5a4**

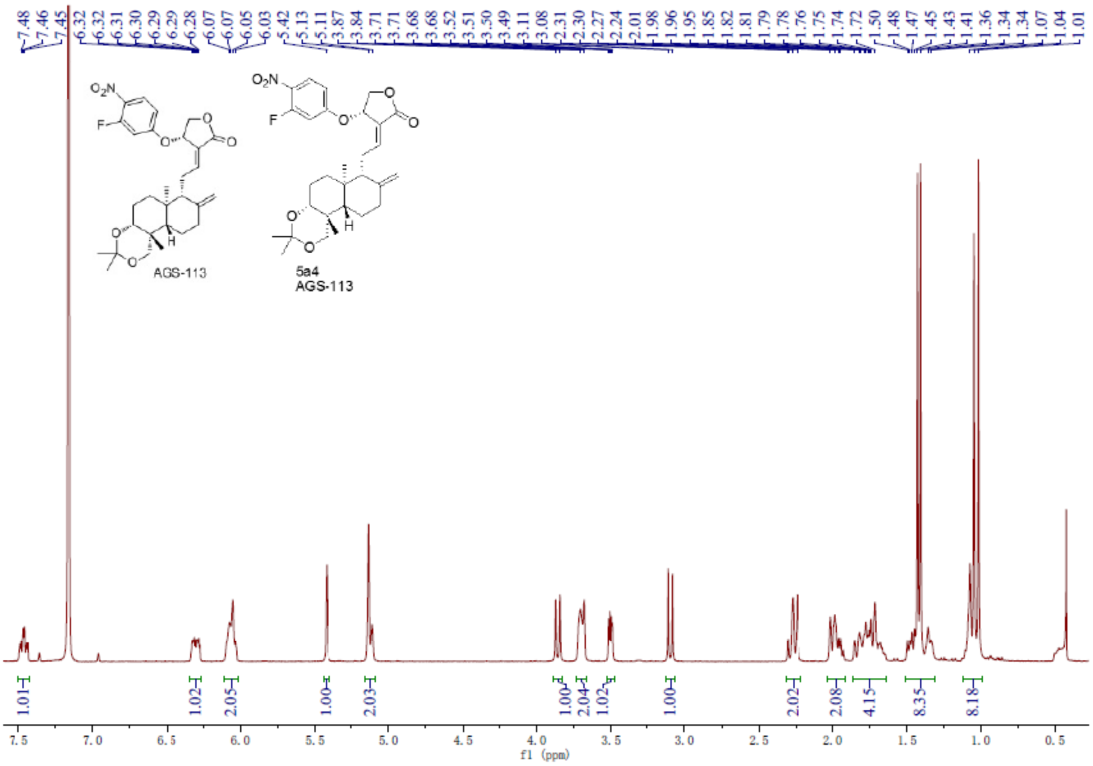

^{13}C NMR of **5a4**

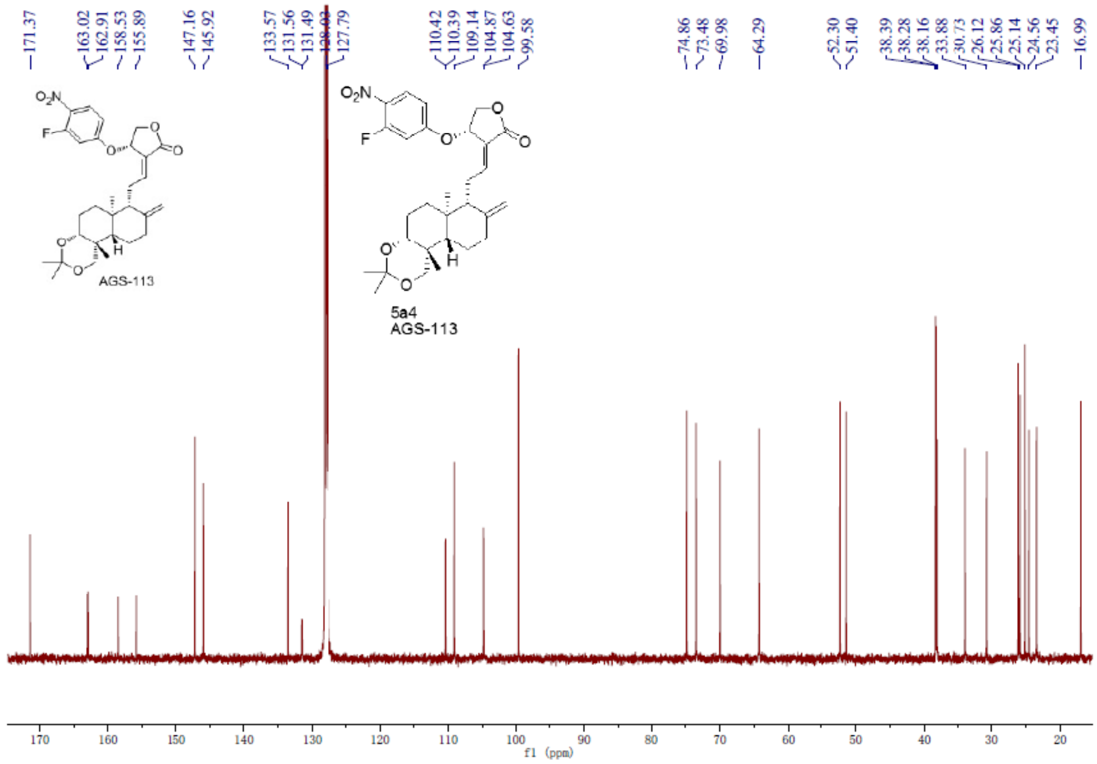

^{1}H NMR of **5b4**

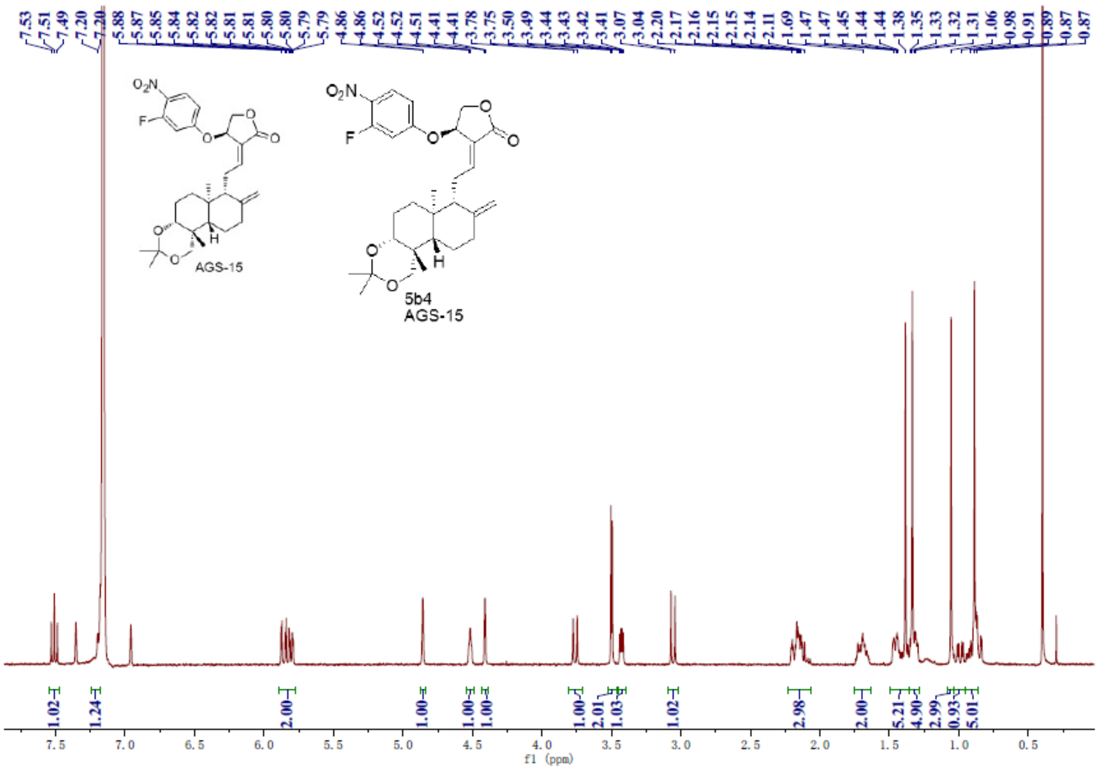

^{13}C NMR of **5b4**

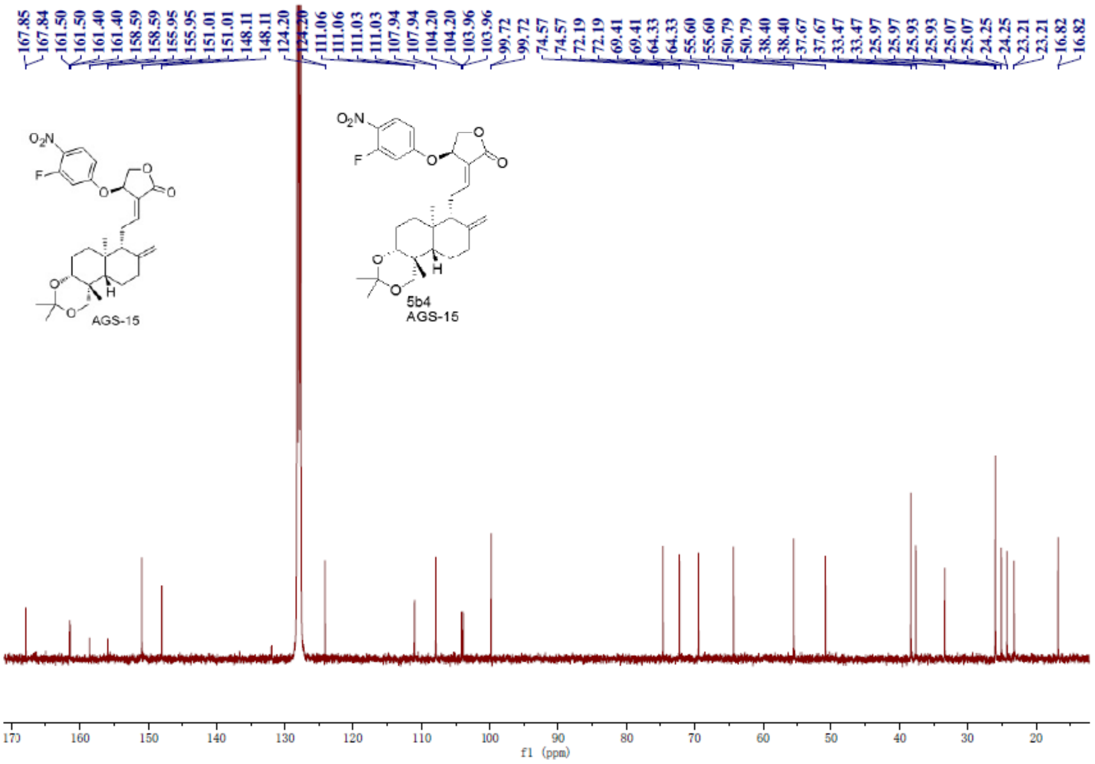

^{1}H NMR of **5a5**

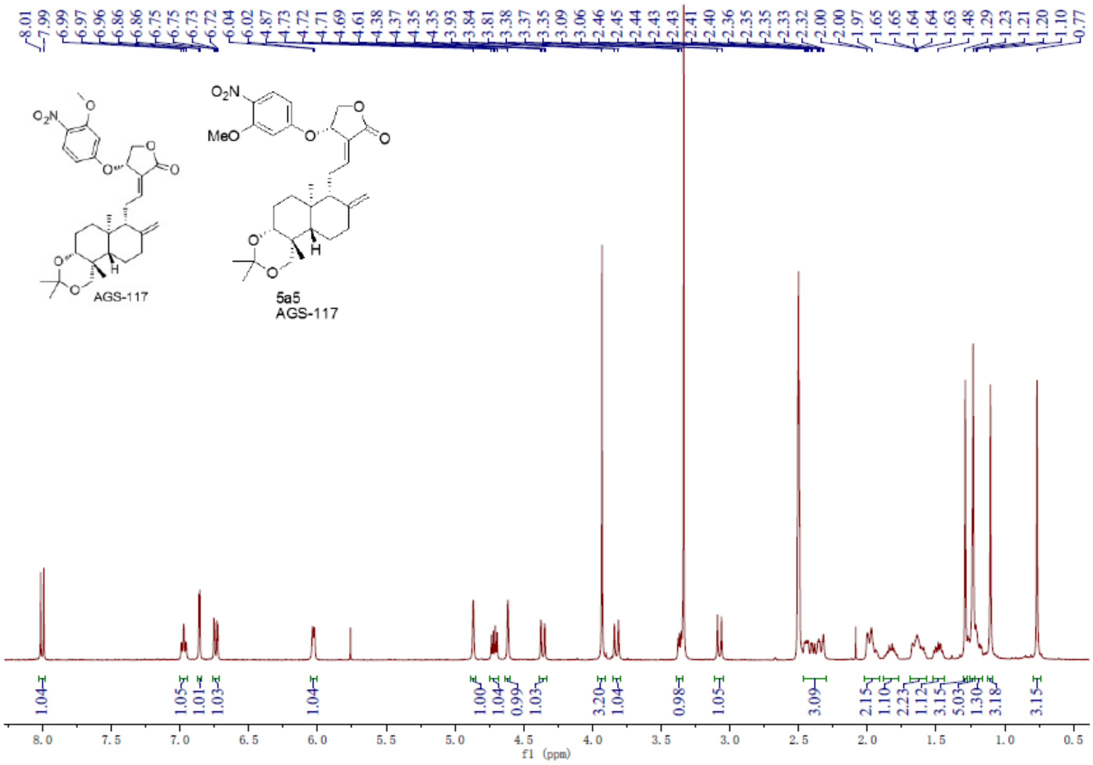

^{13}C NMR of **5a5**

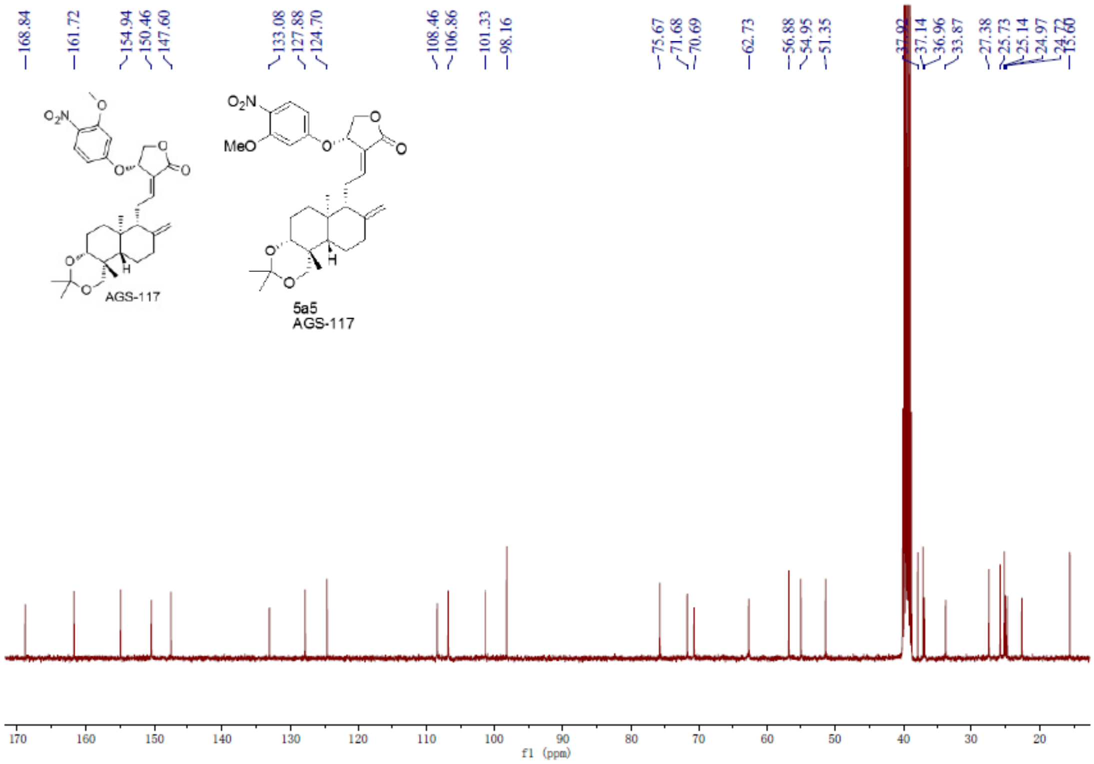

^{1}H NMR of **5b5**

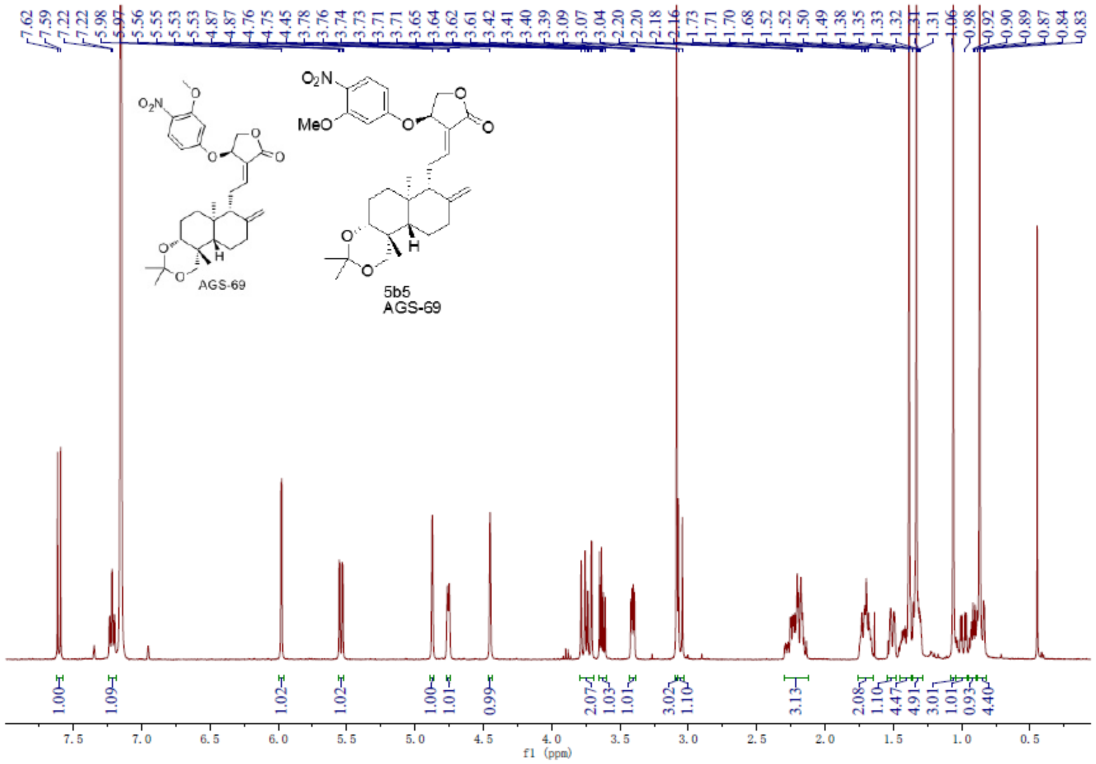

^{13}C NMR of **5b5**

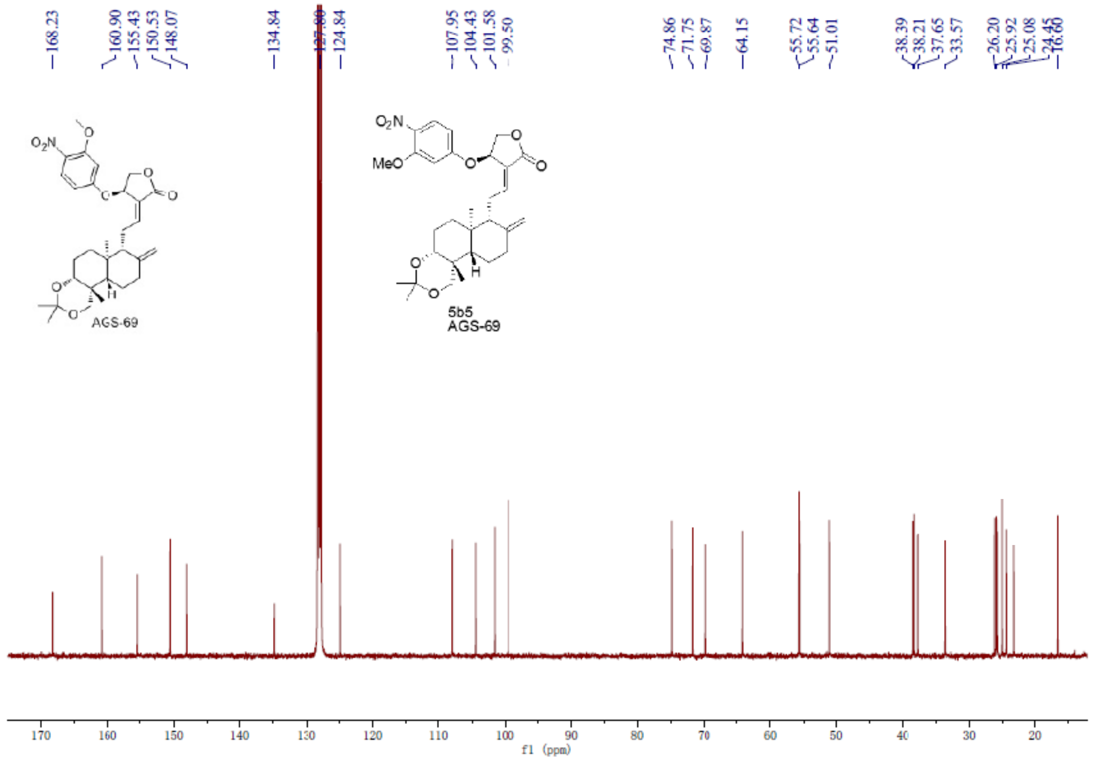

^{1}H NMR of **5b6**

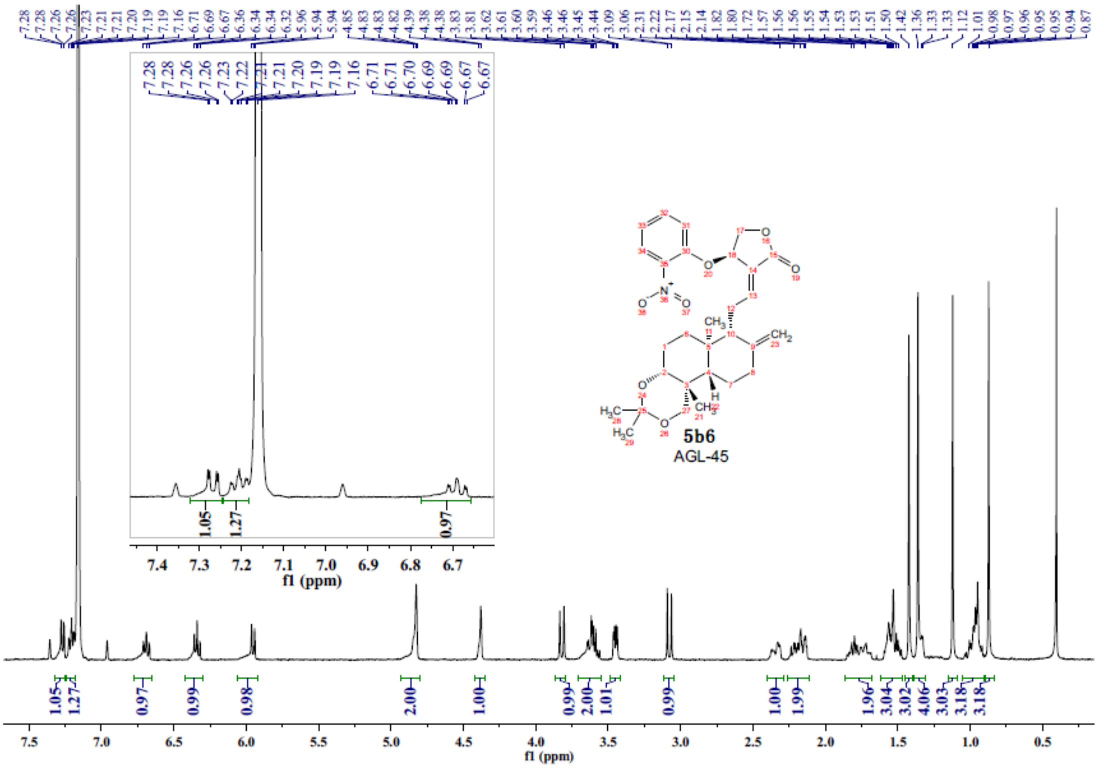

^{13}C NMR of **5b6**

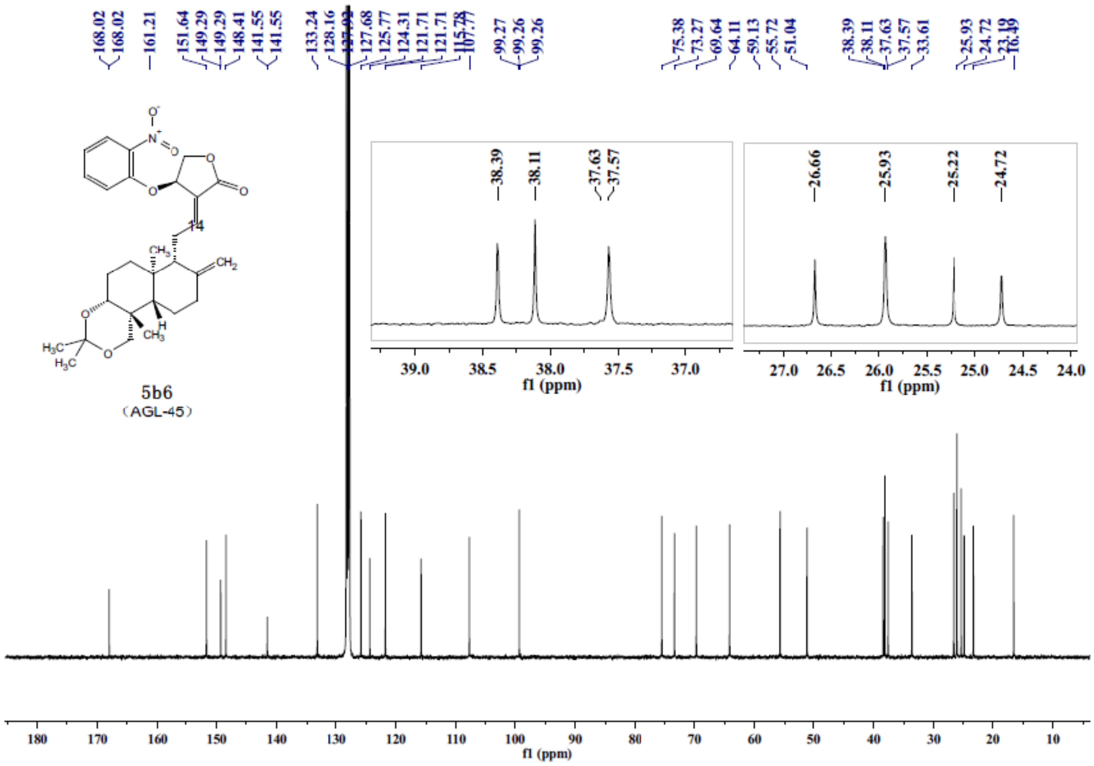

^{1}H NMR of **5b7**

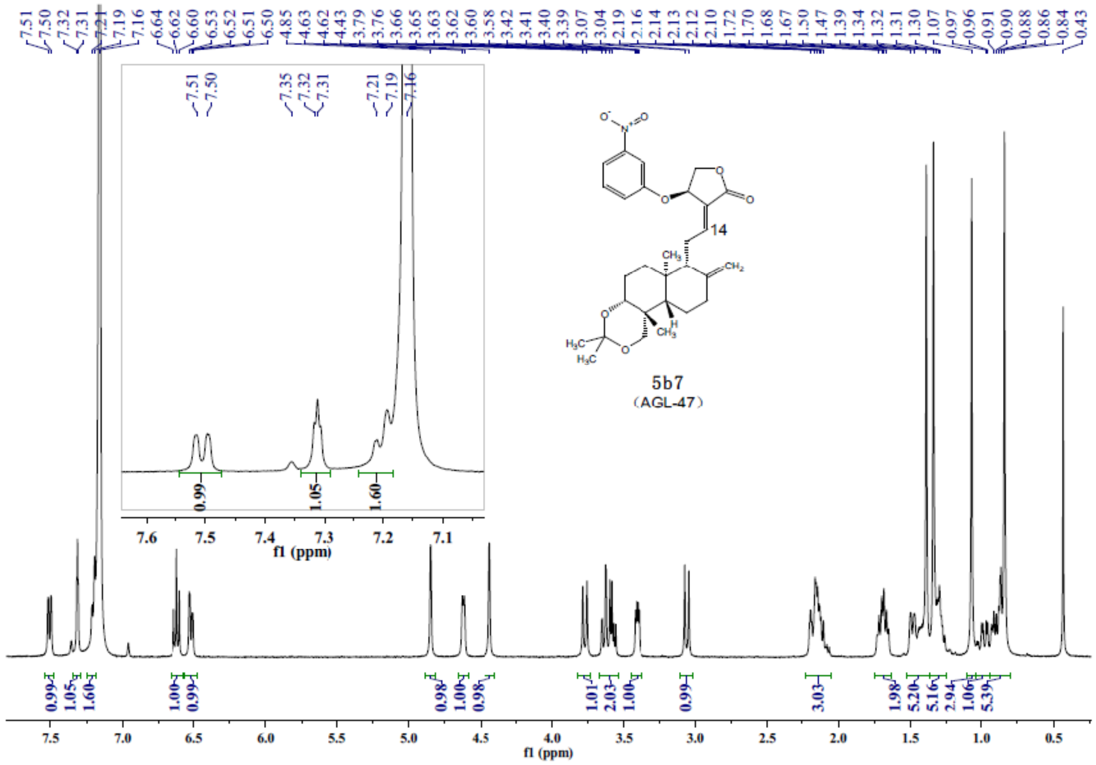

^{13}C NMR of **5b7**

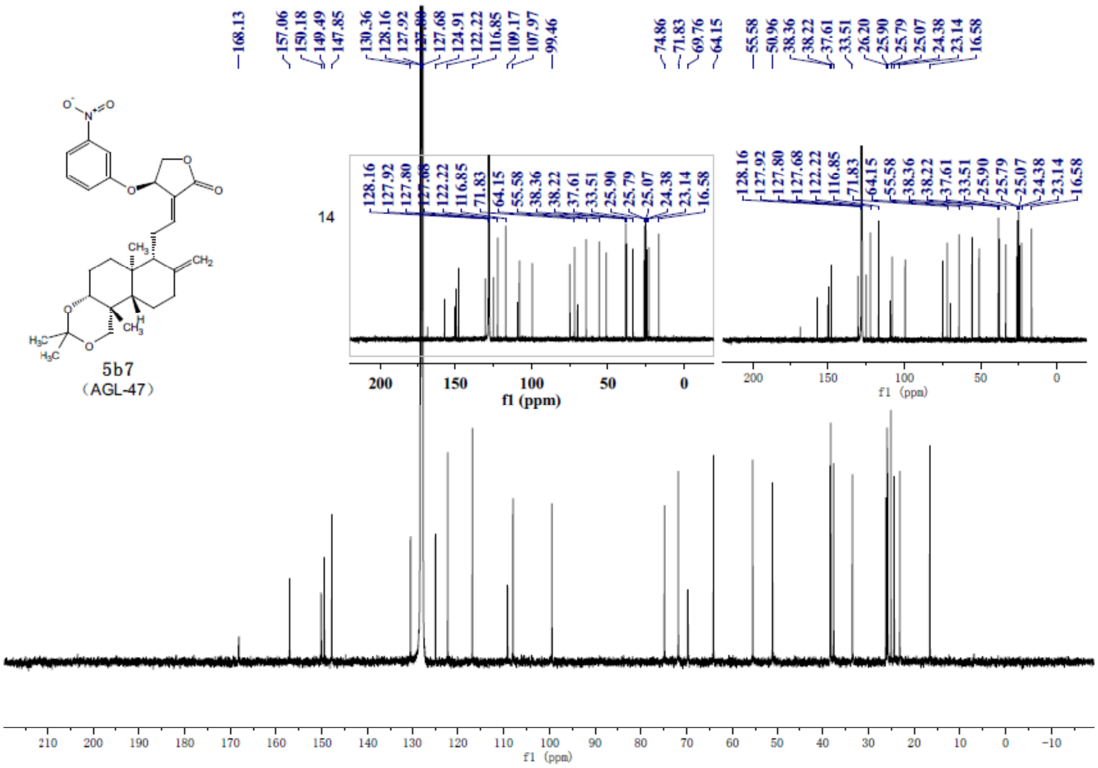

^{1}H NMR of **5a8**

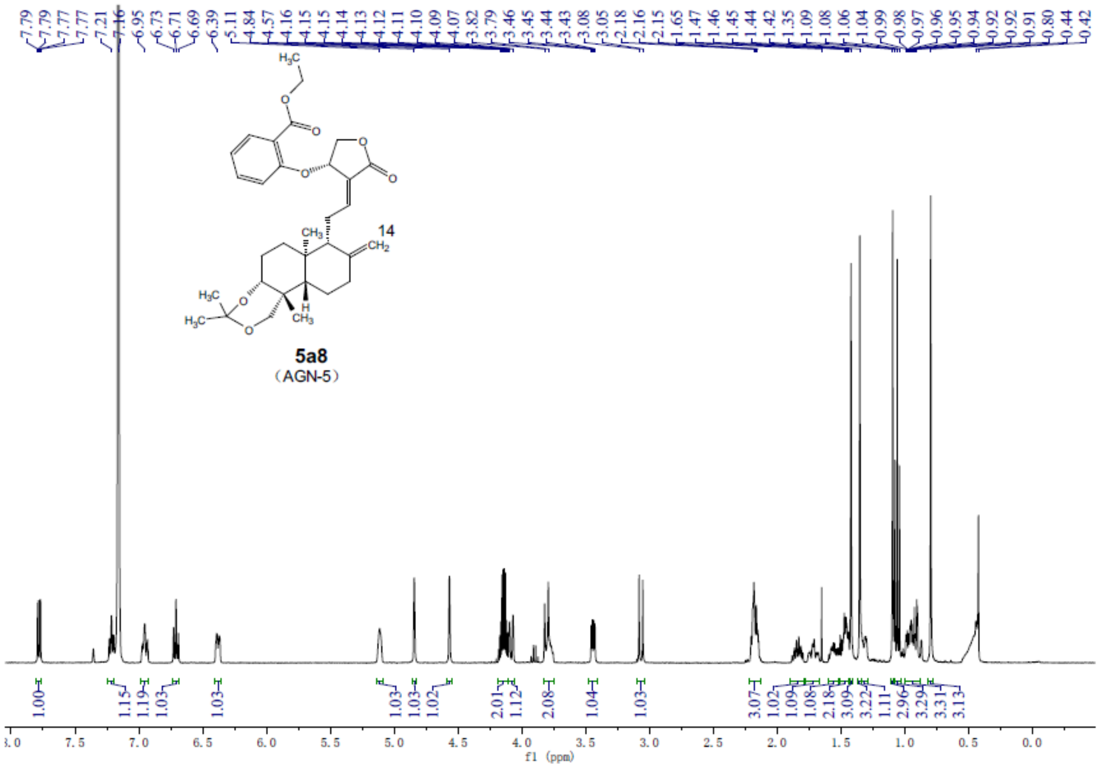

^{13}C NMR of **5a8**

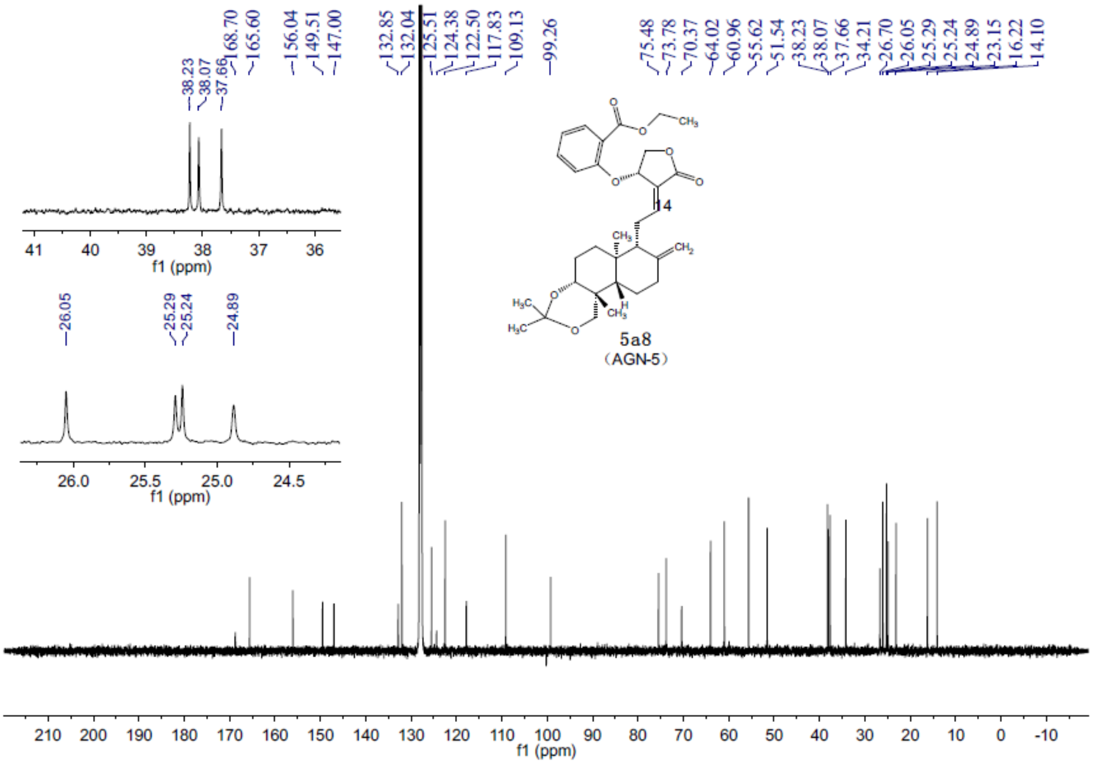

^{1}H NMR of **5b8**

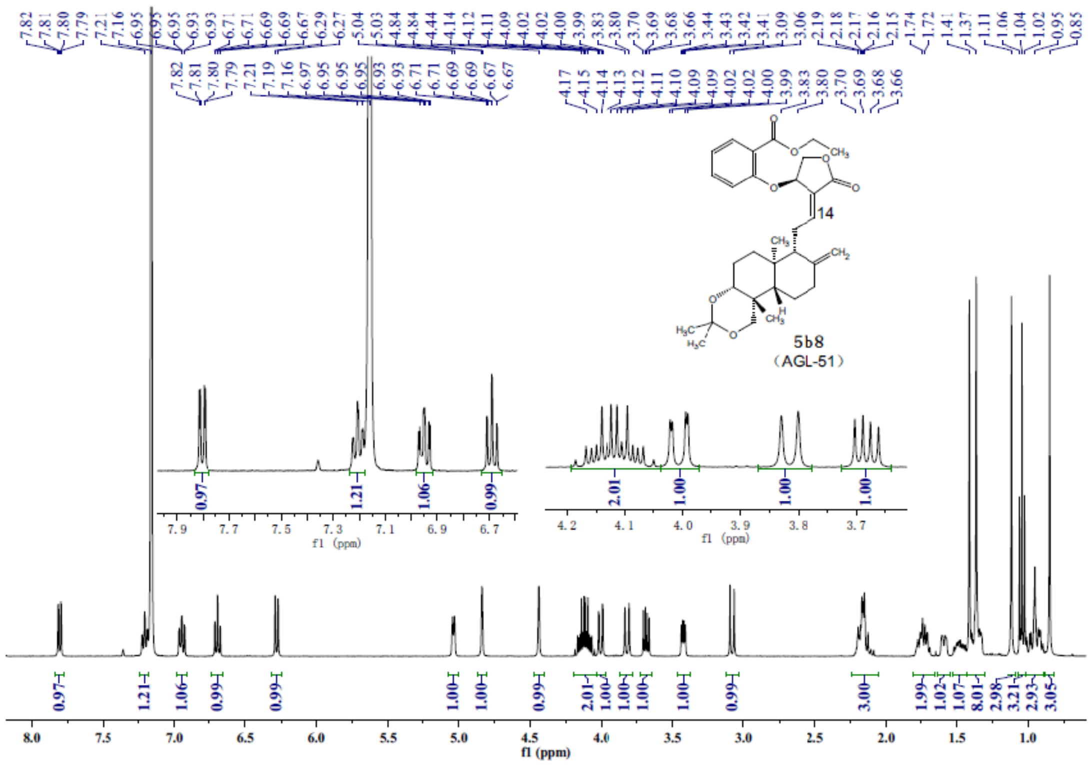

^{13}C NMR of **5b8**

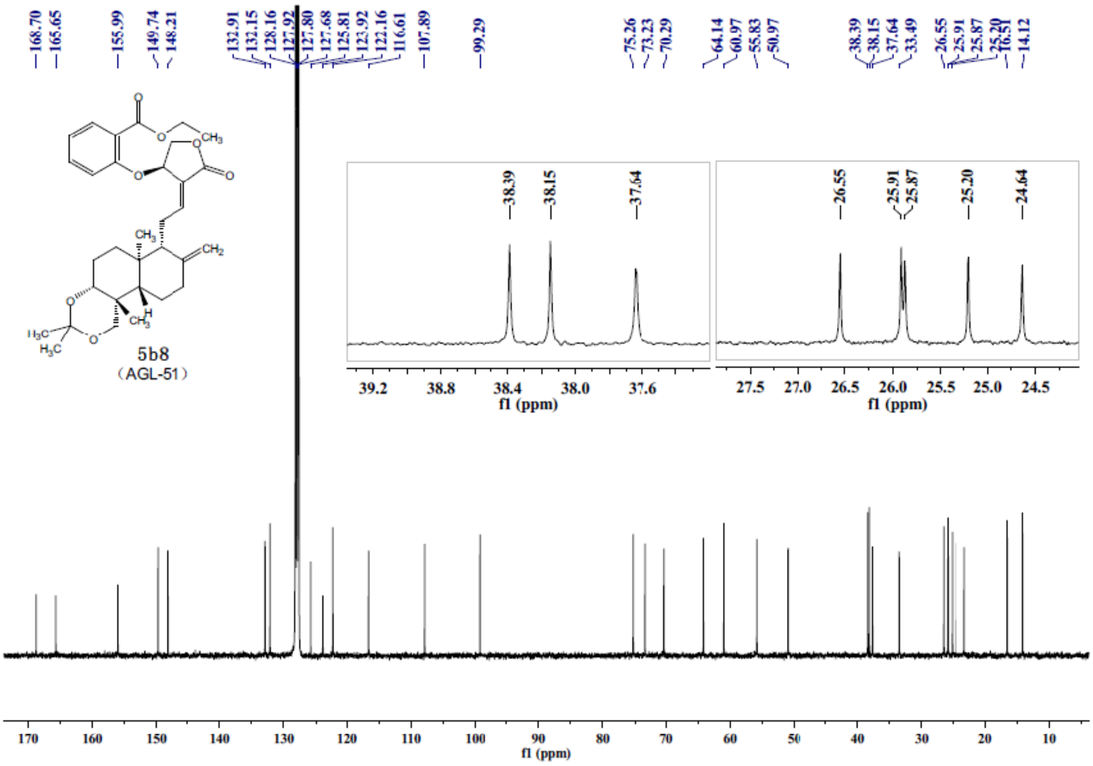

^{1}H NMR of **5a9**

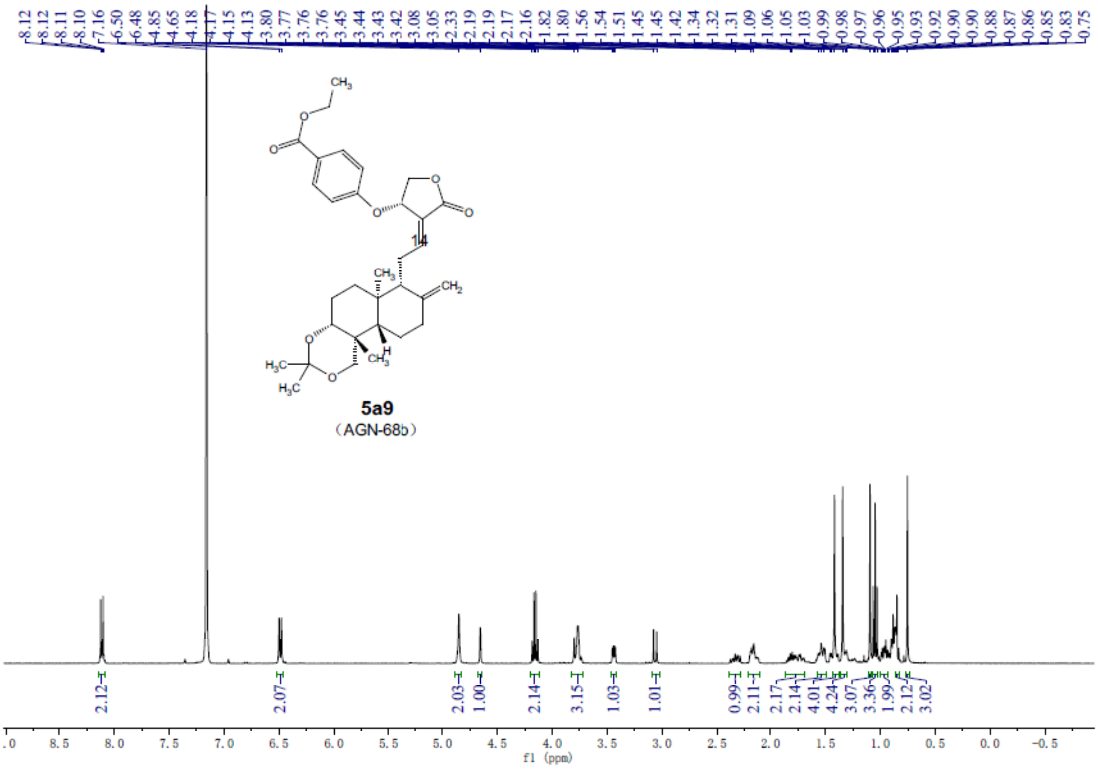

^{13}C NMR of **5a9**

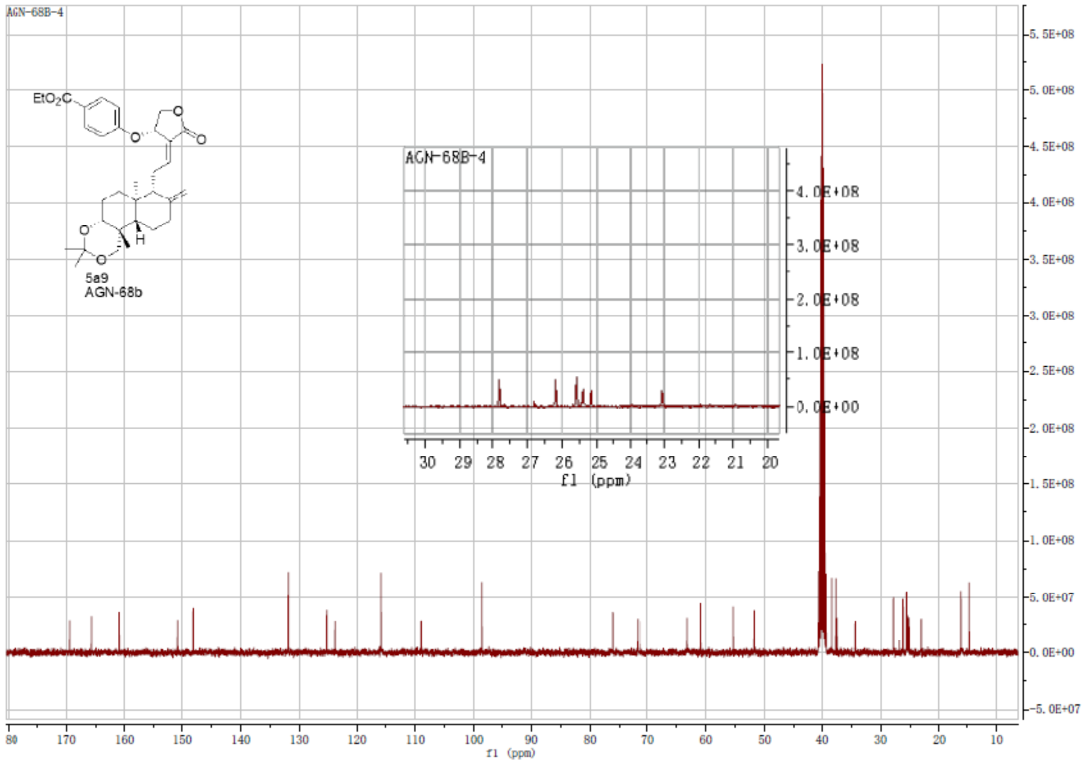

^{1}H NMR of **5b9**

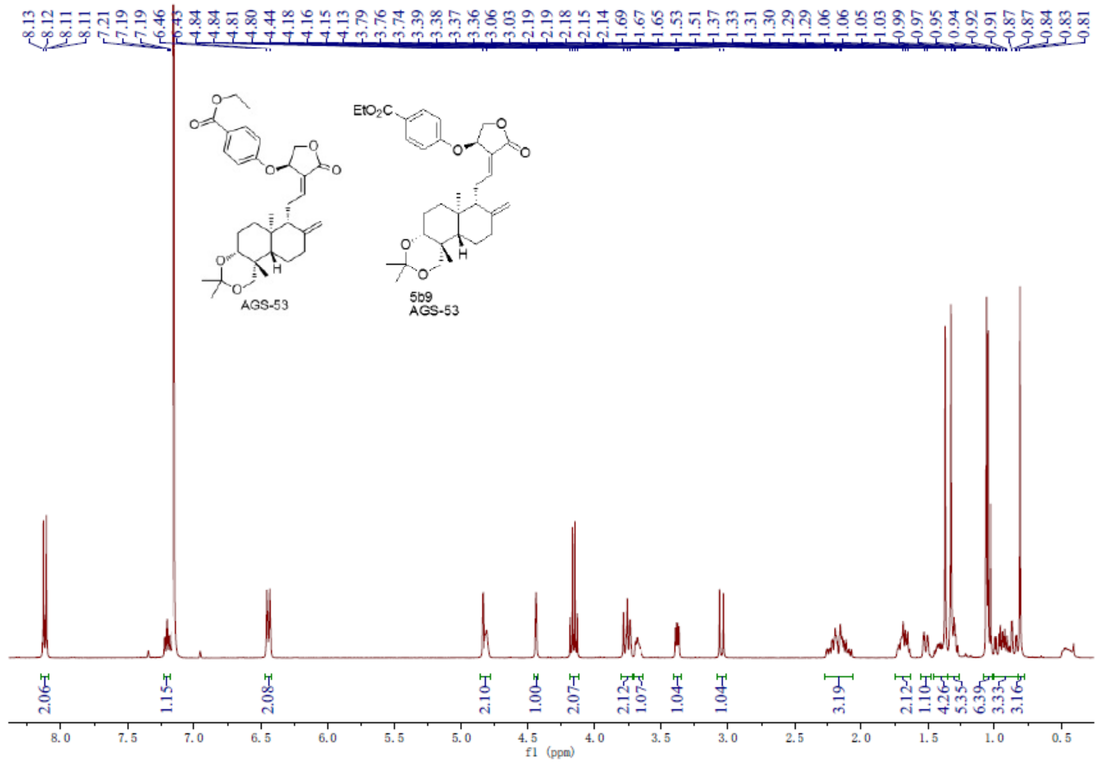

^{13}C NMR of **5b9**

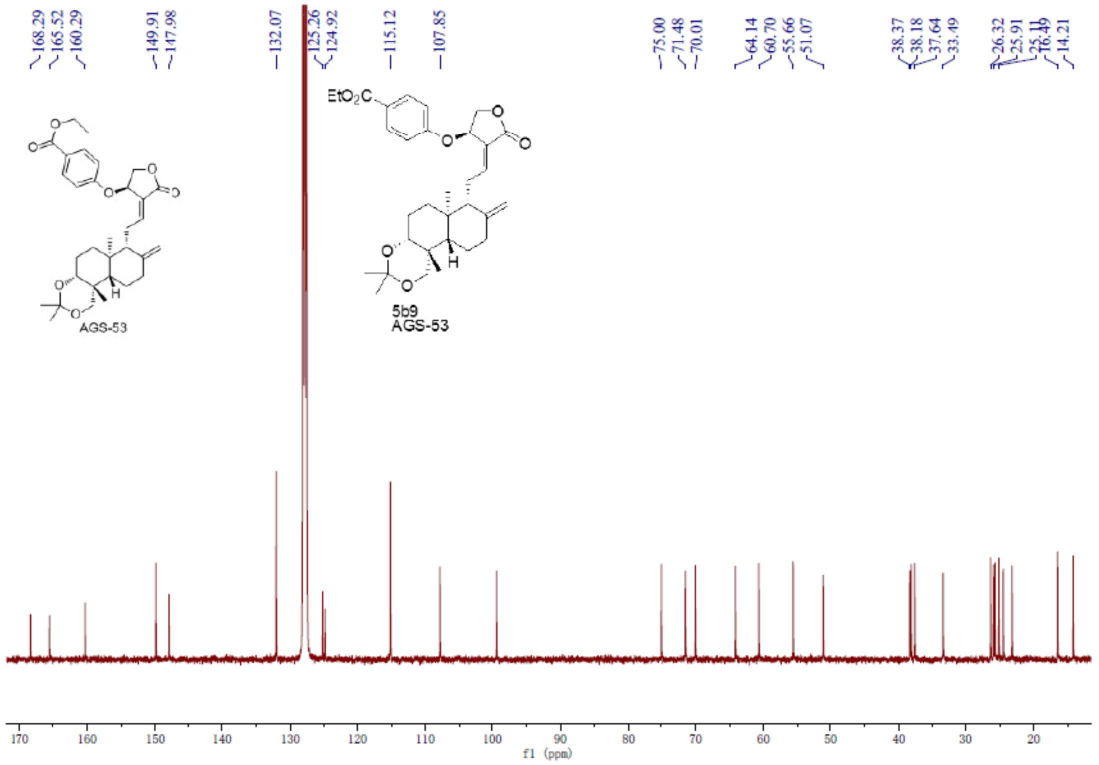

^{1}H NMR of **5b10**

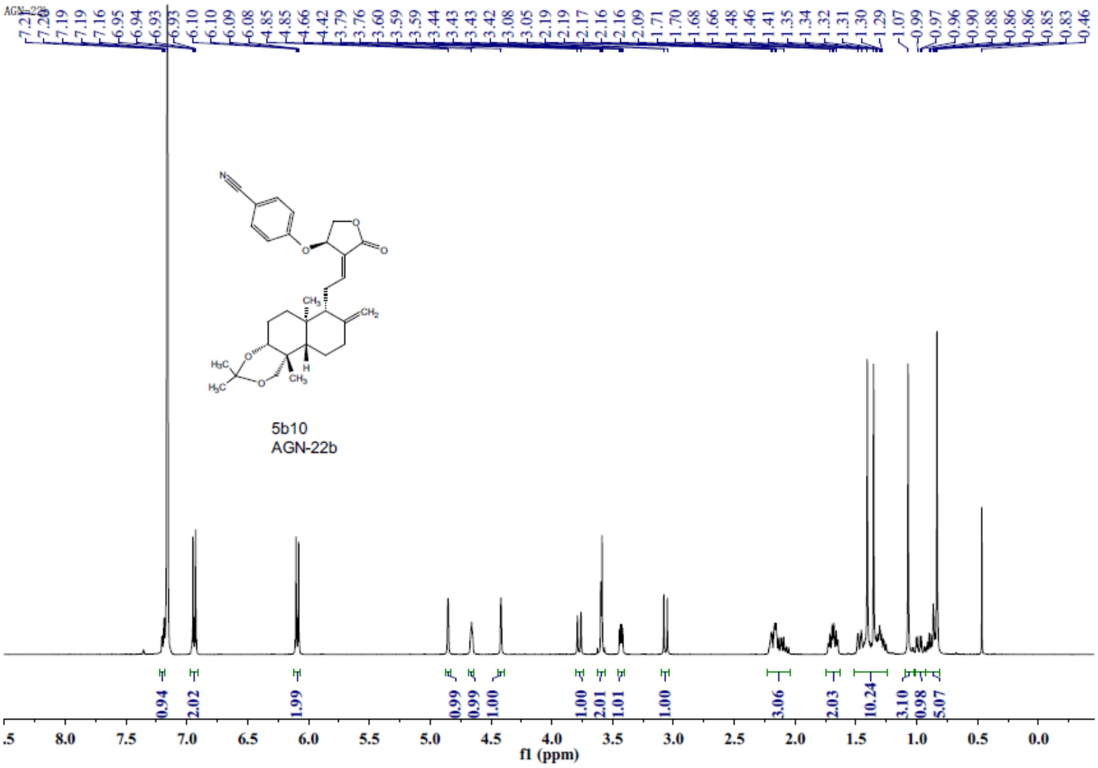

^{13}C NMR of **5b10**

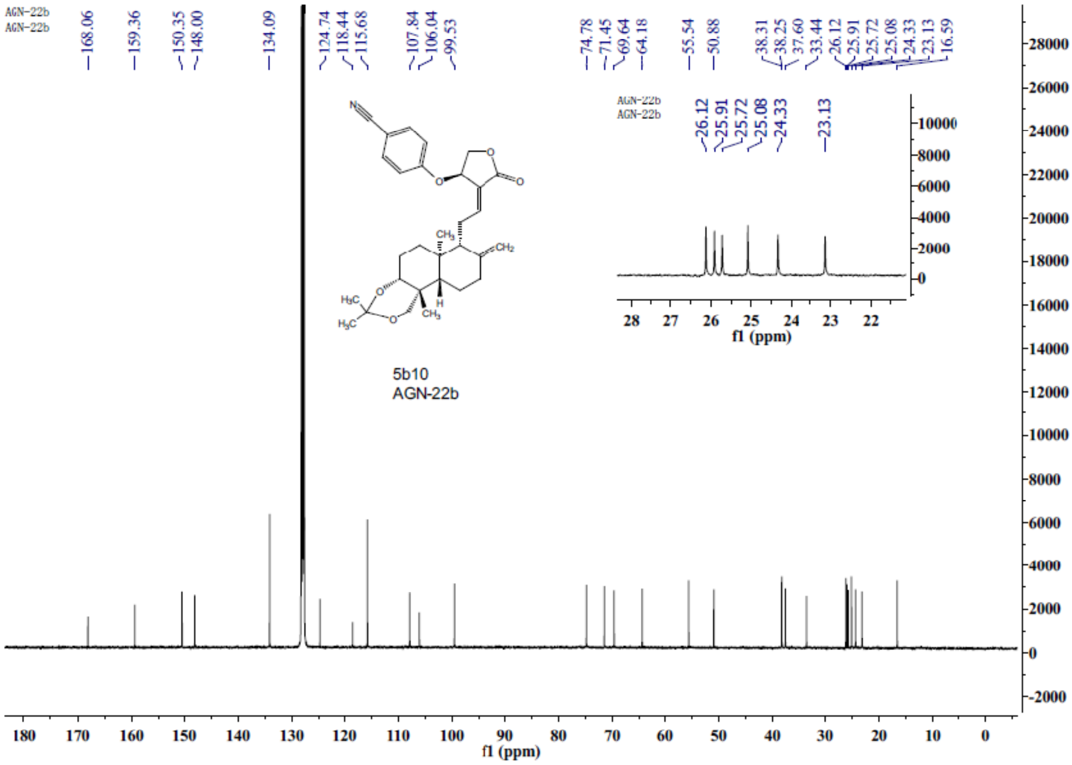

^{1}H NMR of **5b11**

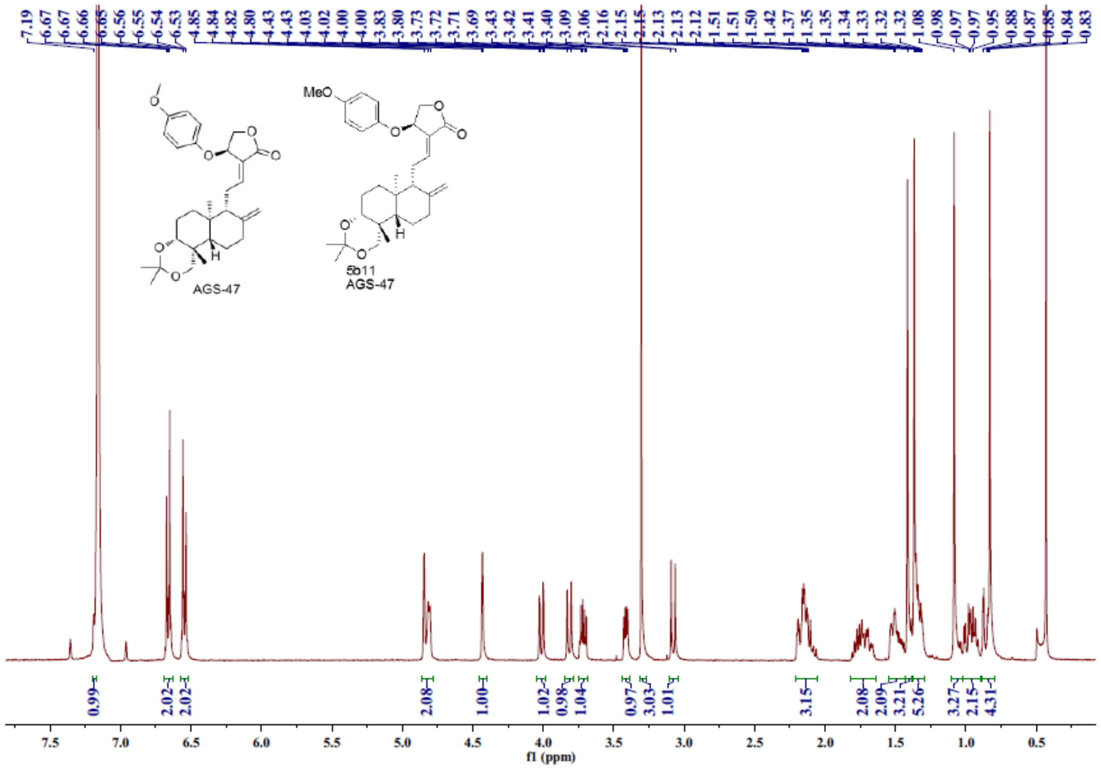

^{13}C NMR of **5b11**

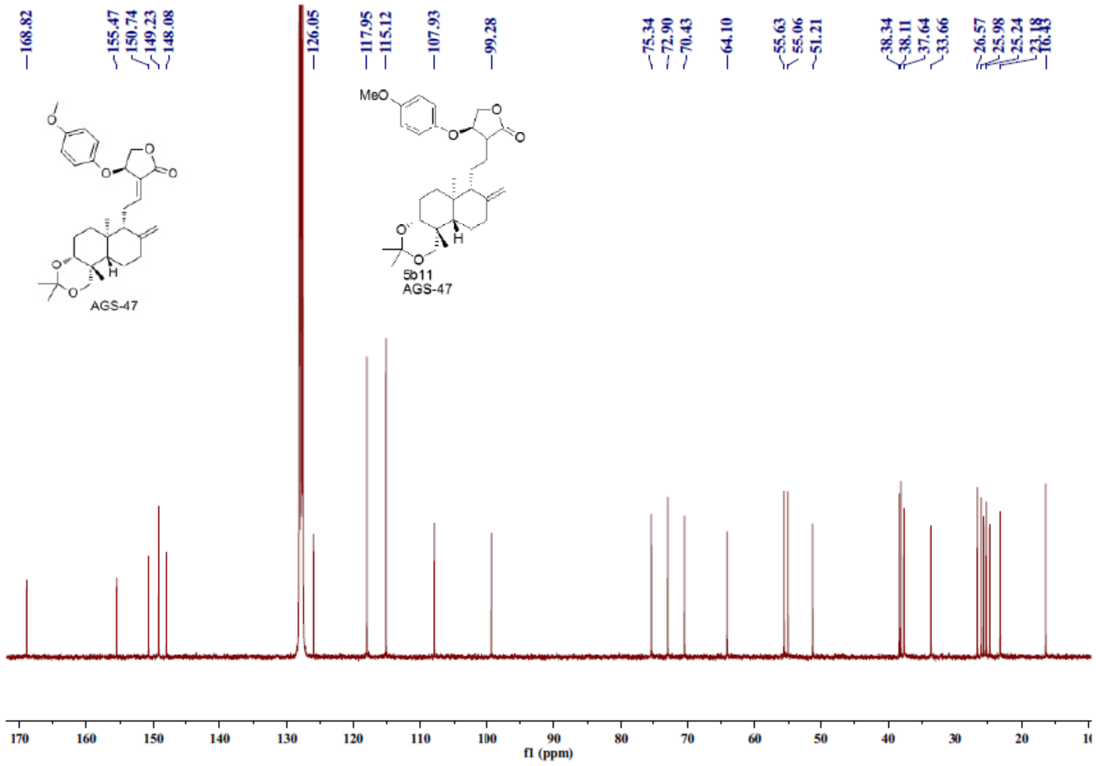

^{1}H NMR of **5a12**

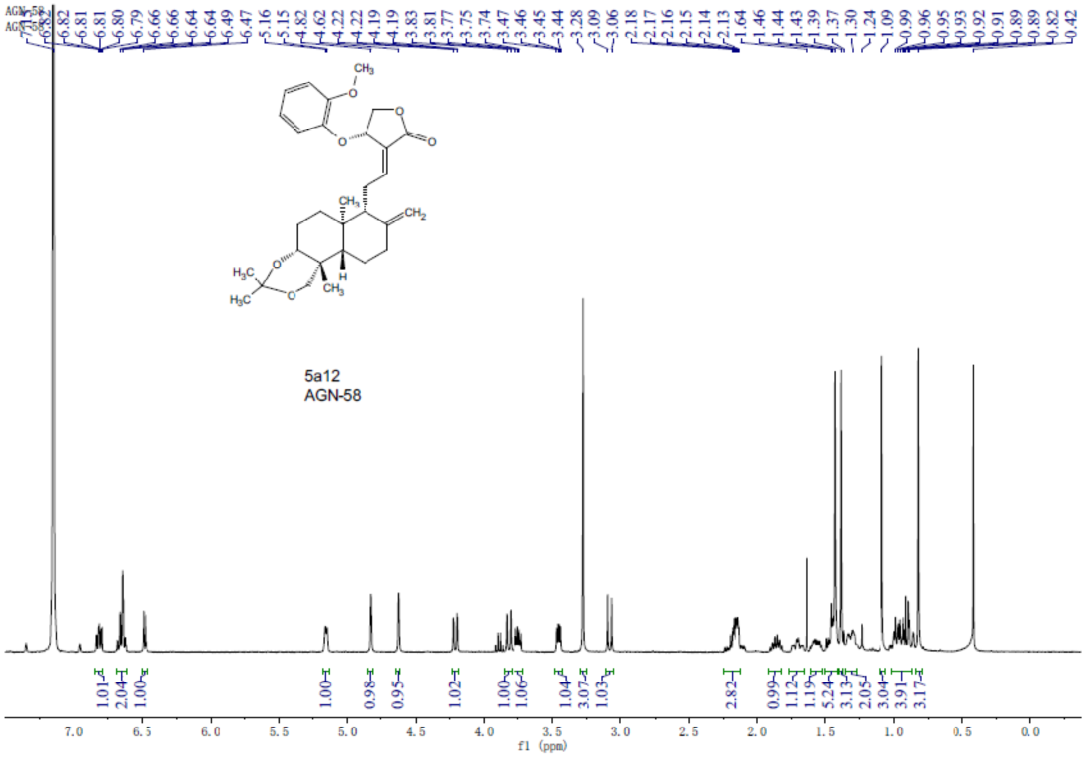

^{13}C NMR of **5a12**

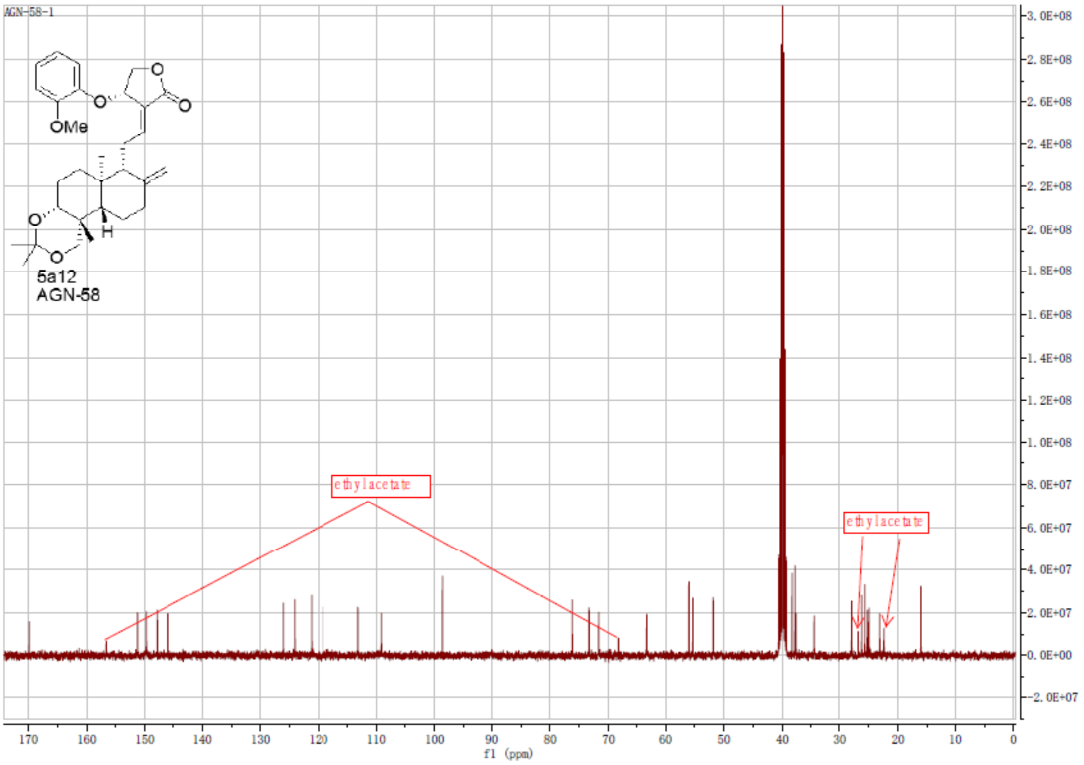

^{1}H NMR of **5b12**

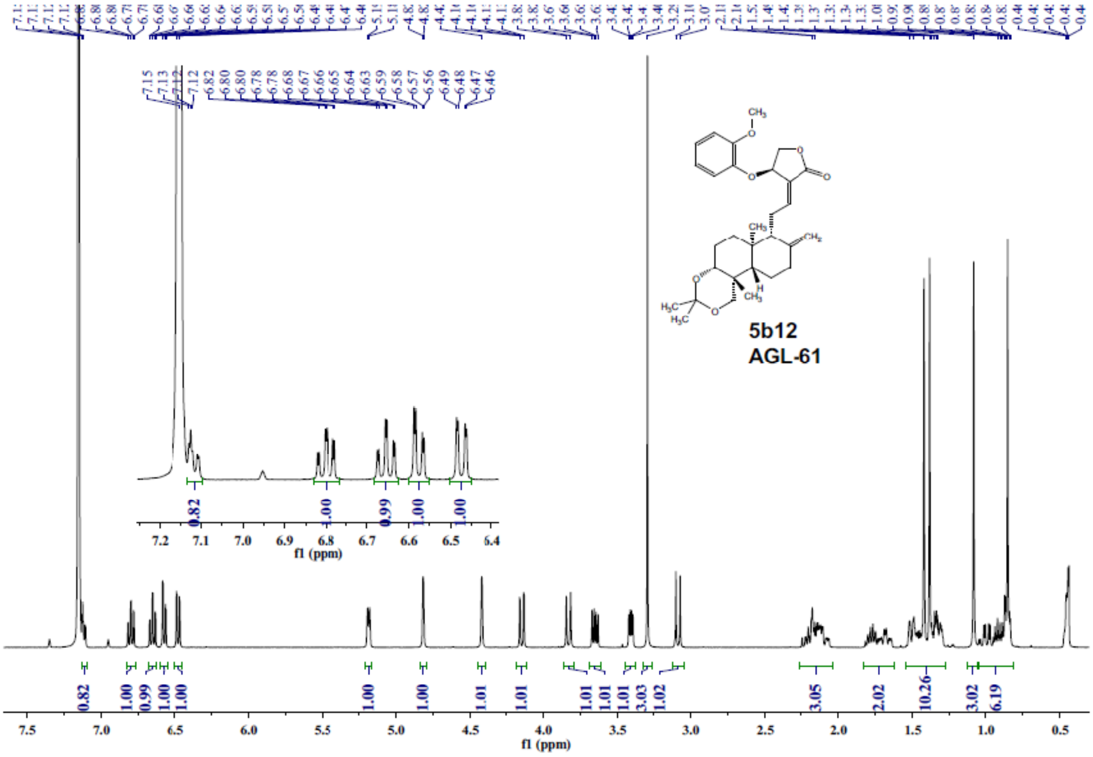

^{13}C NMR of **5b12**

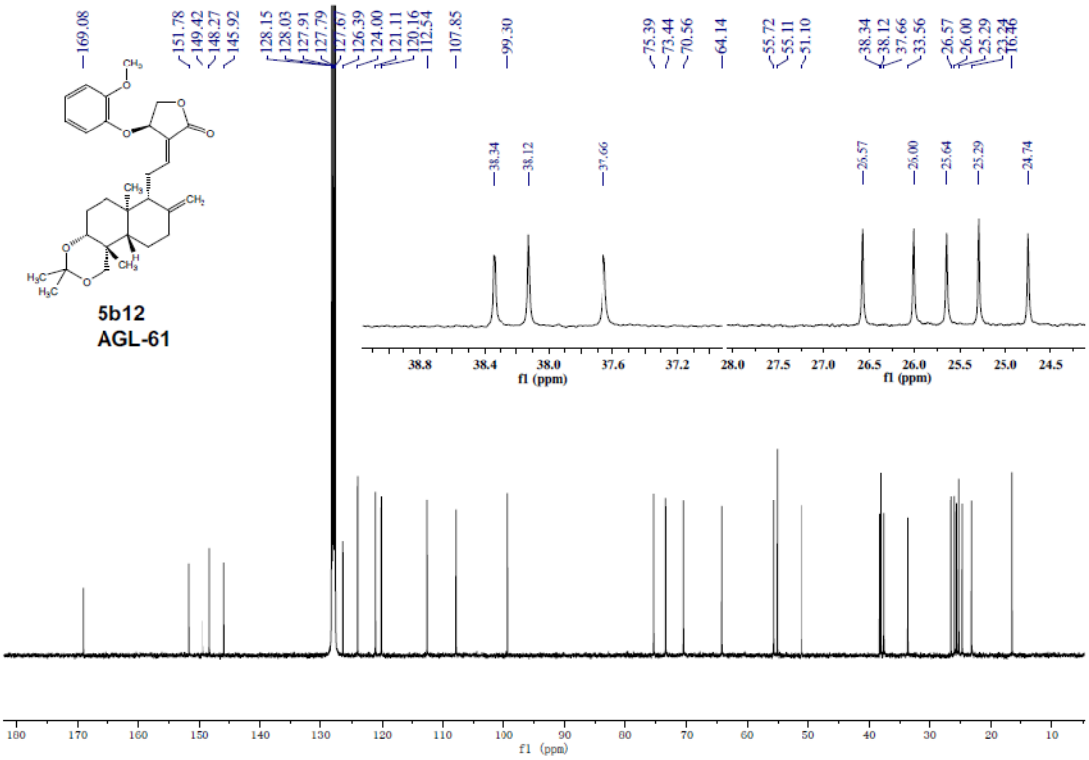

^{1}H NMR of **5b13**

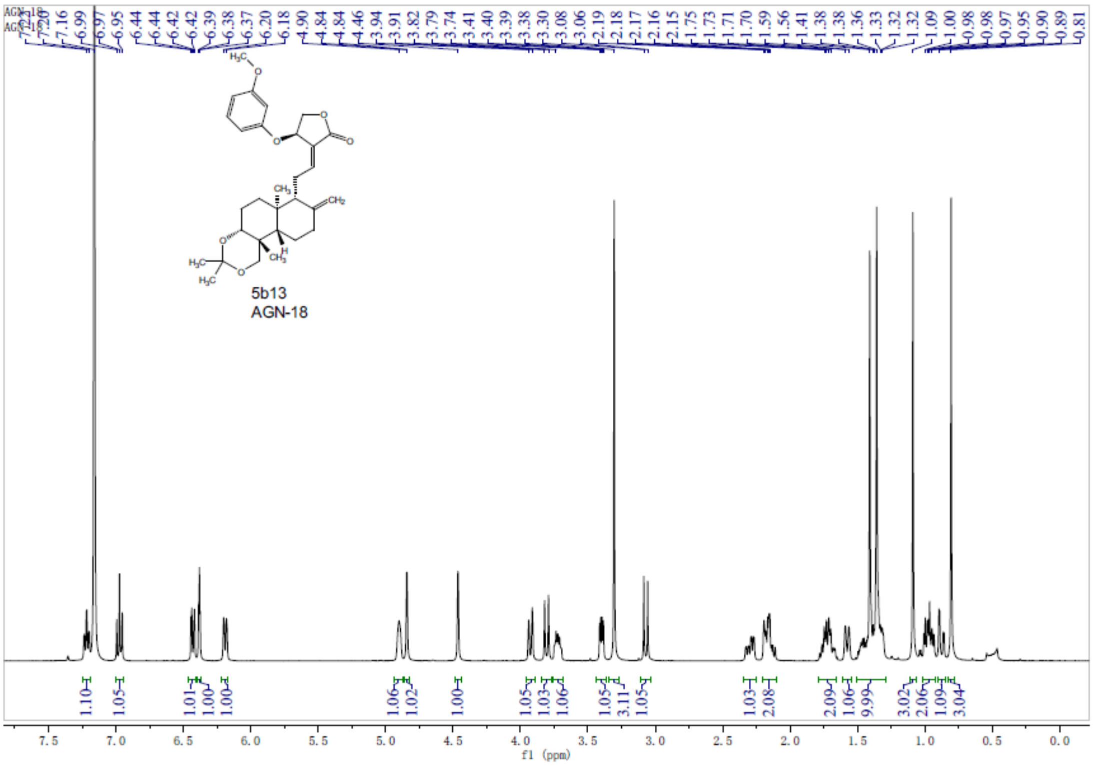

^{13}C NMR of **5b13**

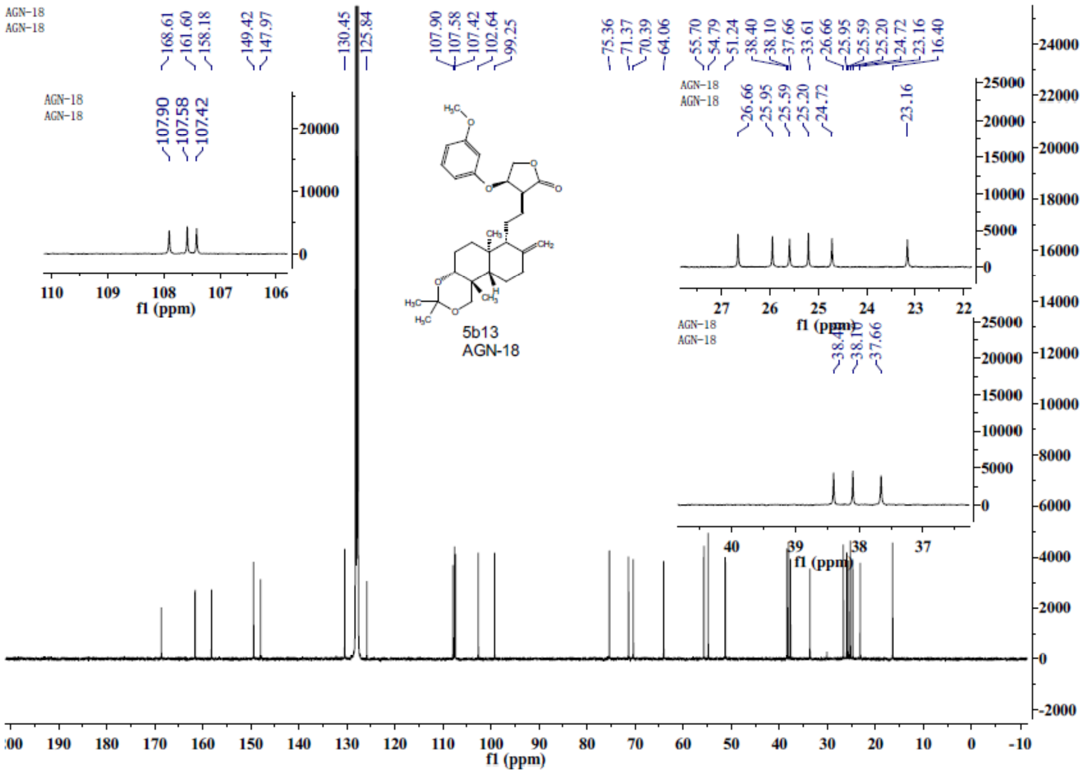

^{1}H NMR of **5b14**

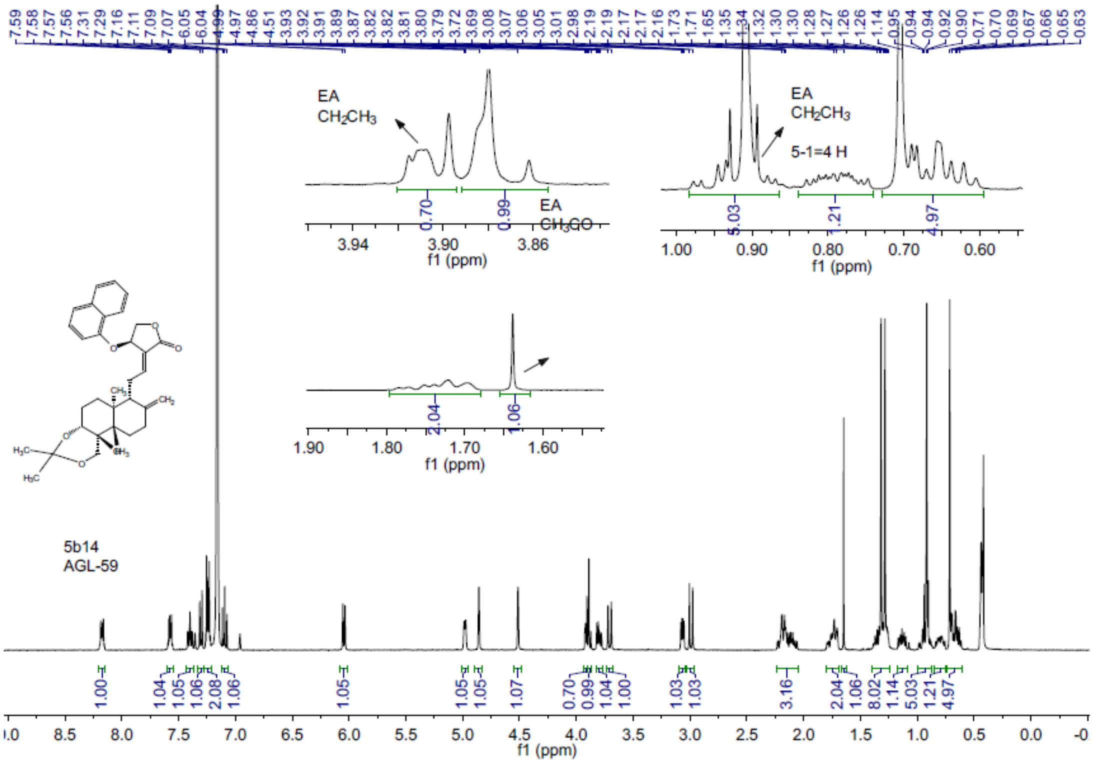

^{13}C NMR of **5b14**

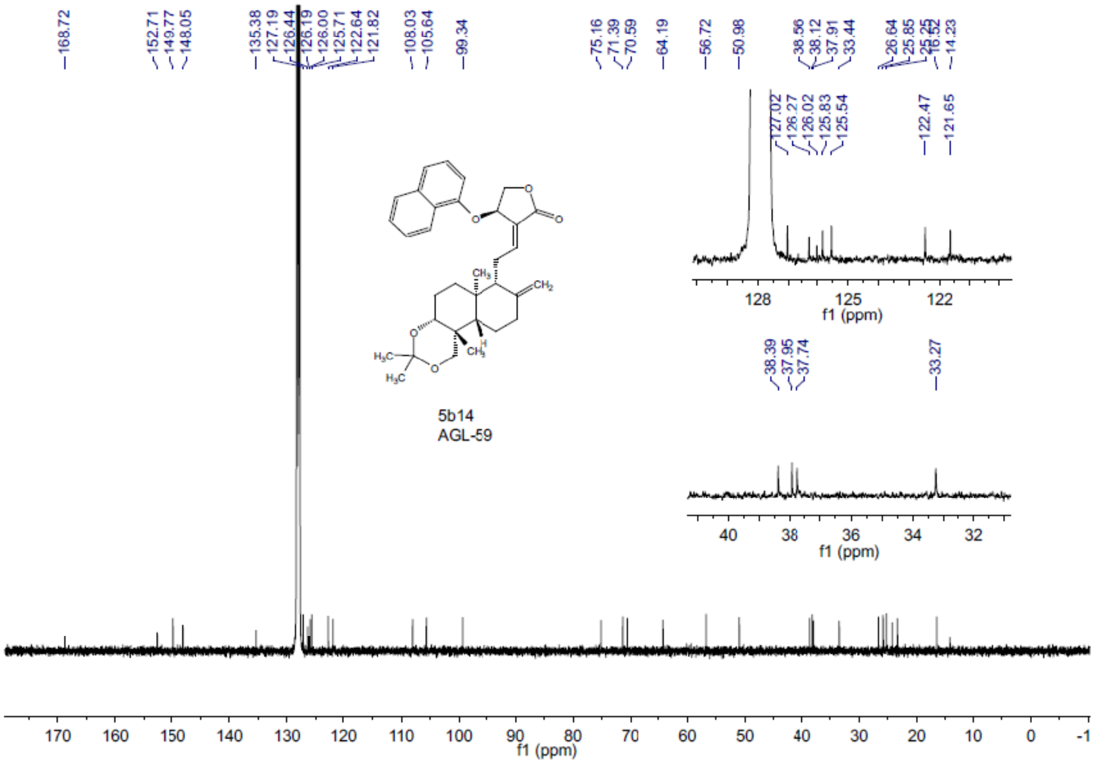

^{1}H NMR of **5a15**

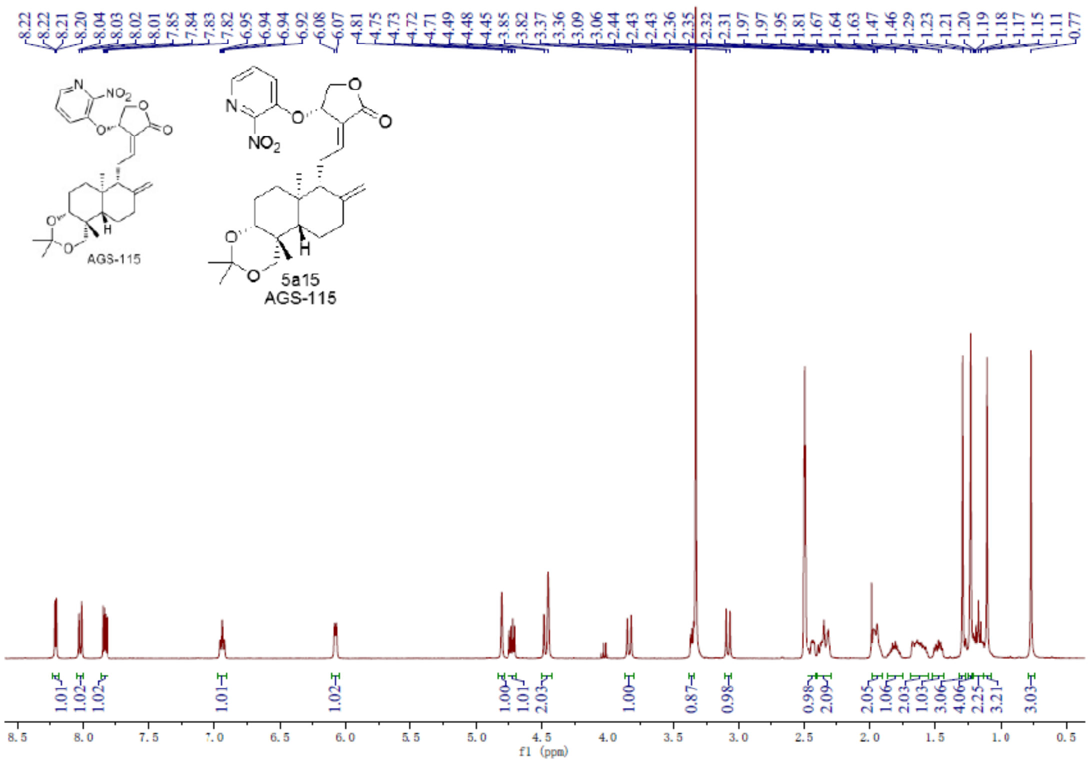

^{13}C NMR of **5a15**

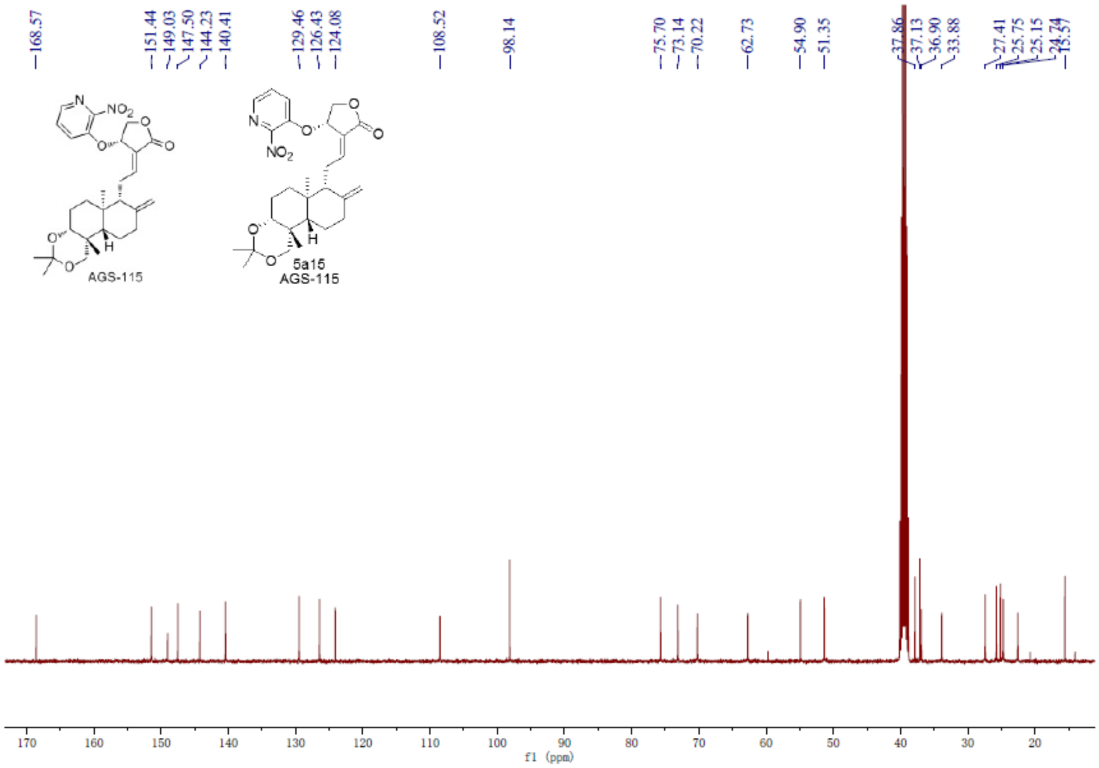

^{1}H NMR of **5b15**

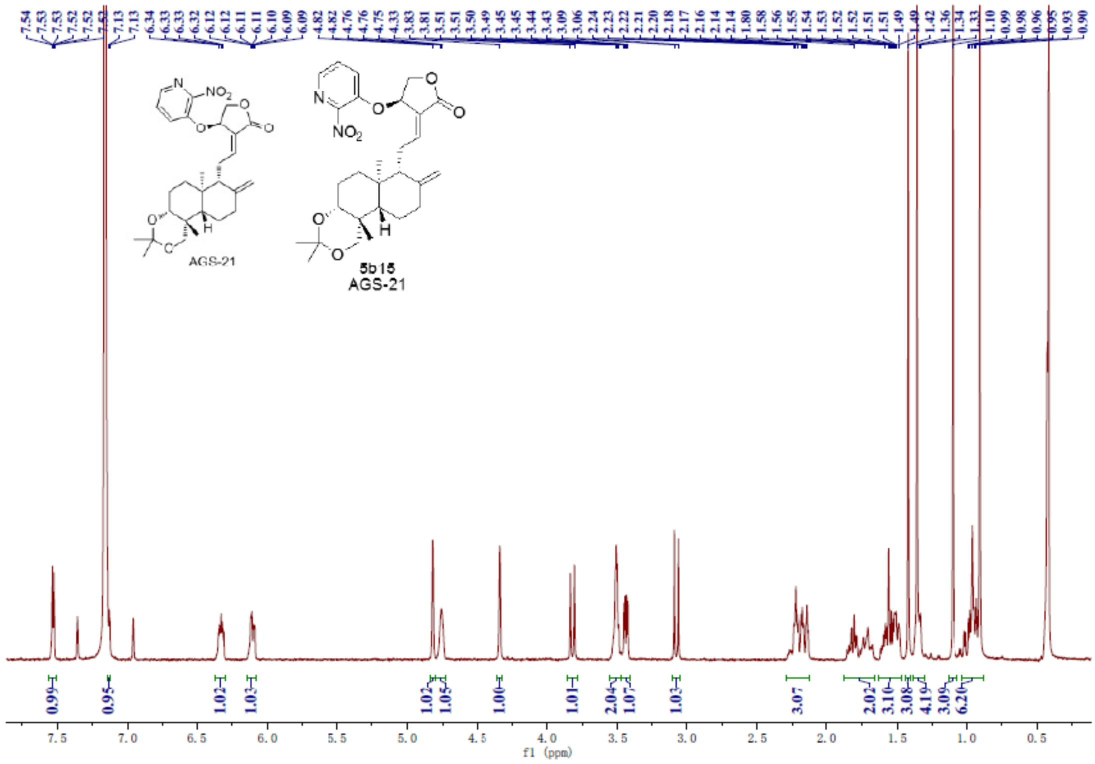

^{13}C NMR of **5b15**

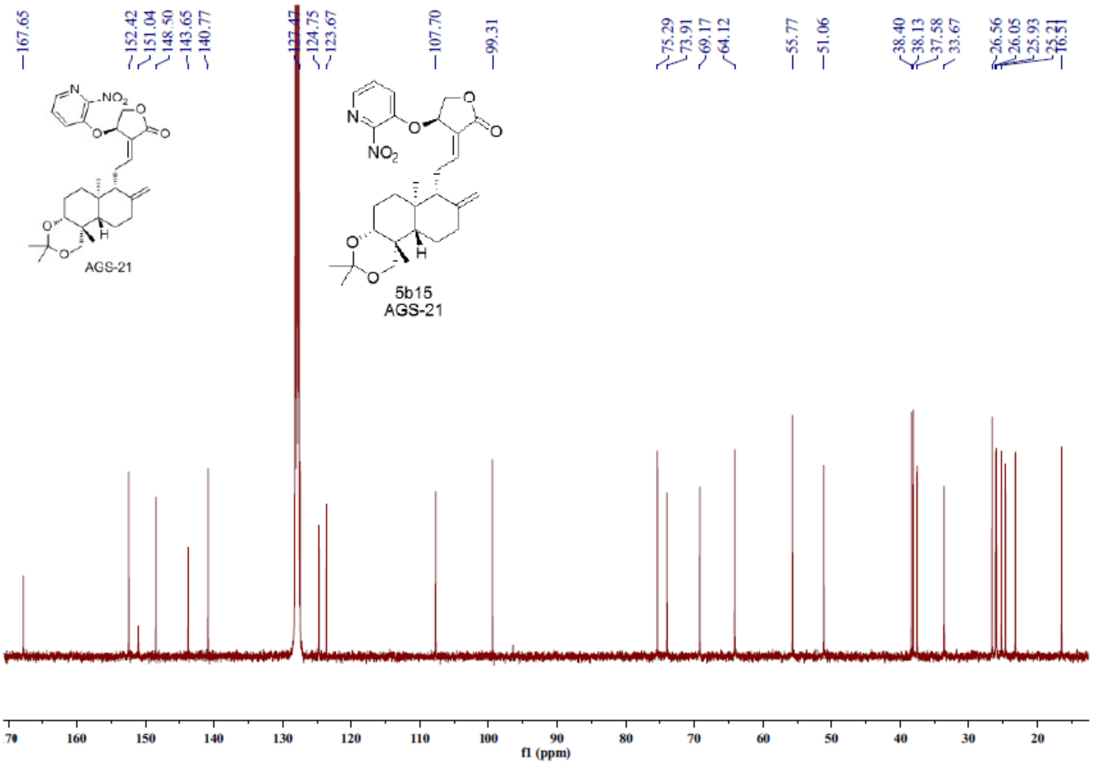

^{1}H NMR of **5a16**

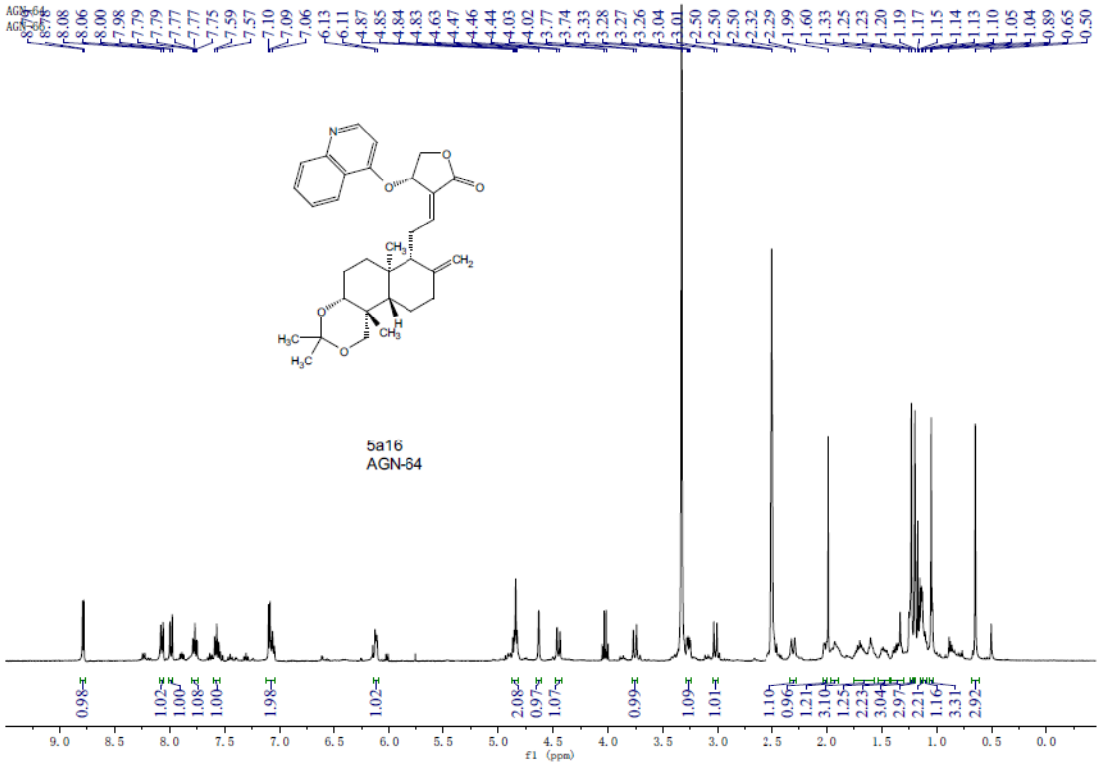

^{13}C NMR of **5a16**

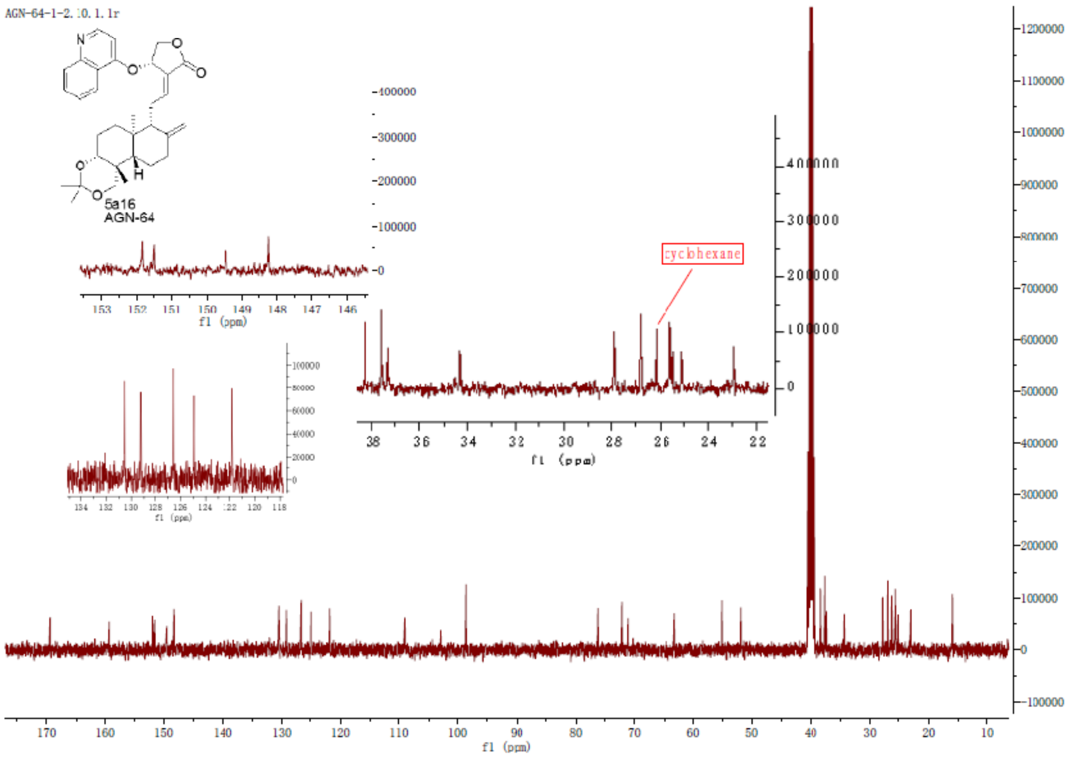

^{1}H NMR of **5b16**

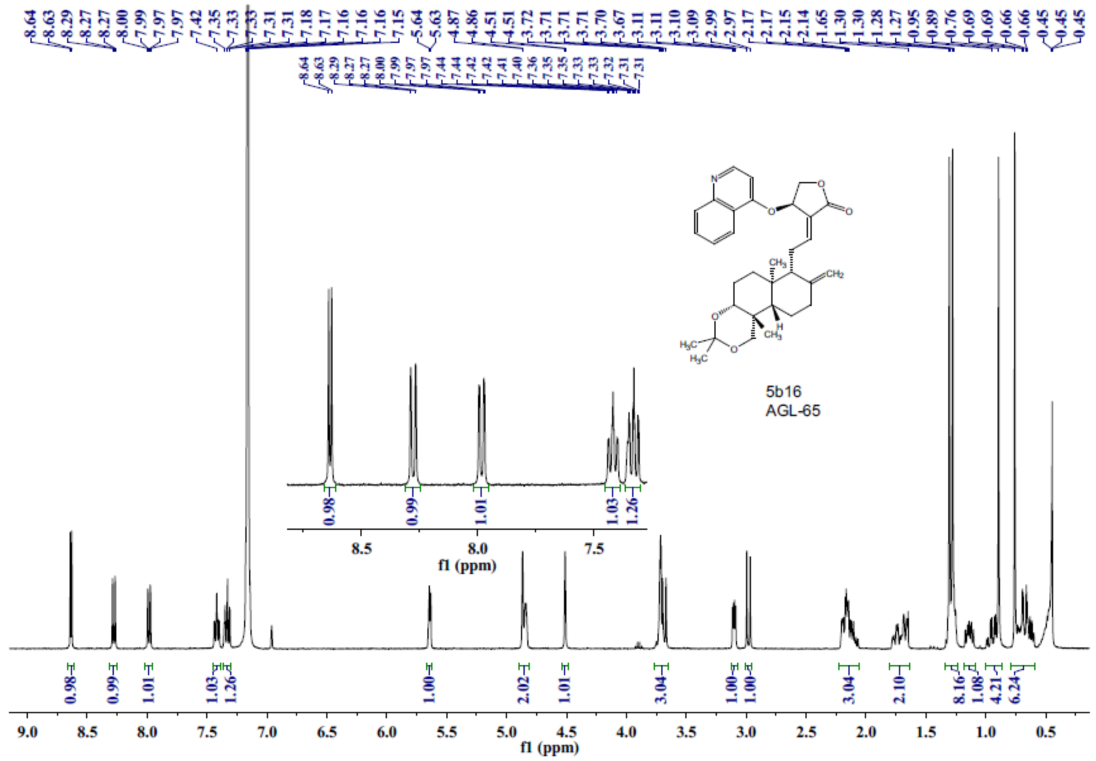

^{13}C NMR of **5b16**

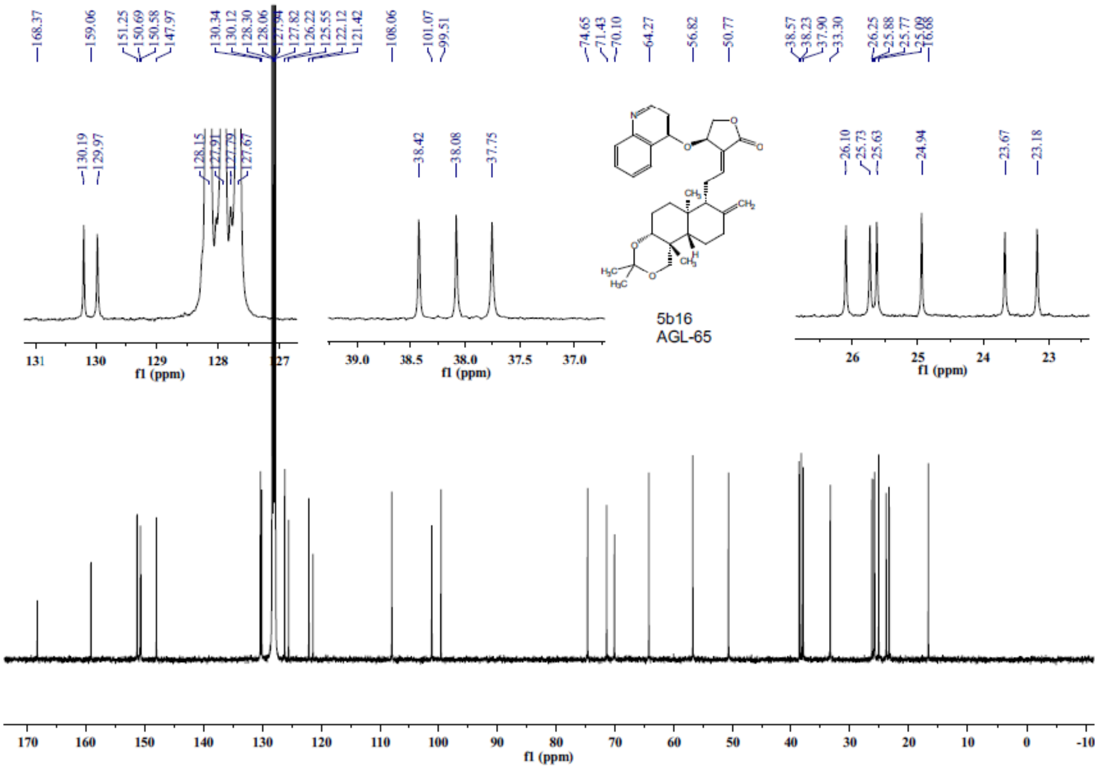

^{1}H NMR of **5a17**

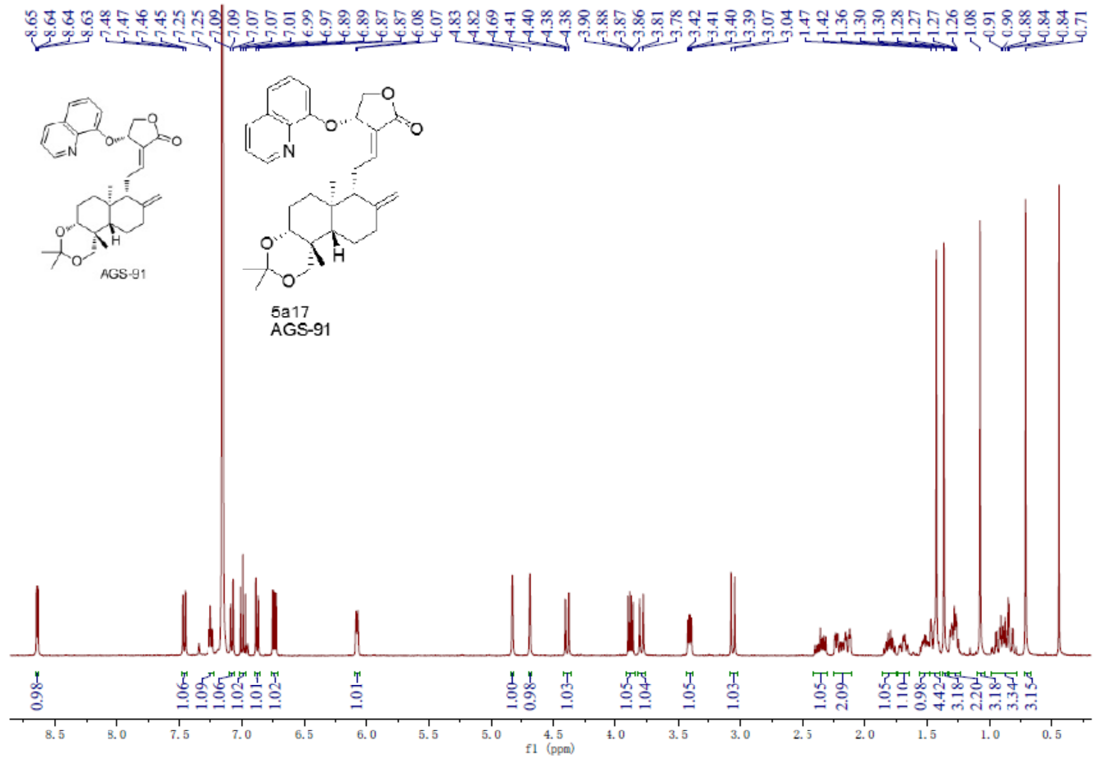

^{13}C NMR of **5a17**

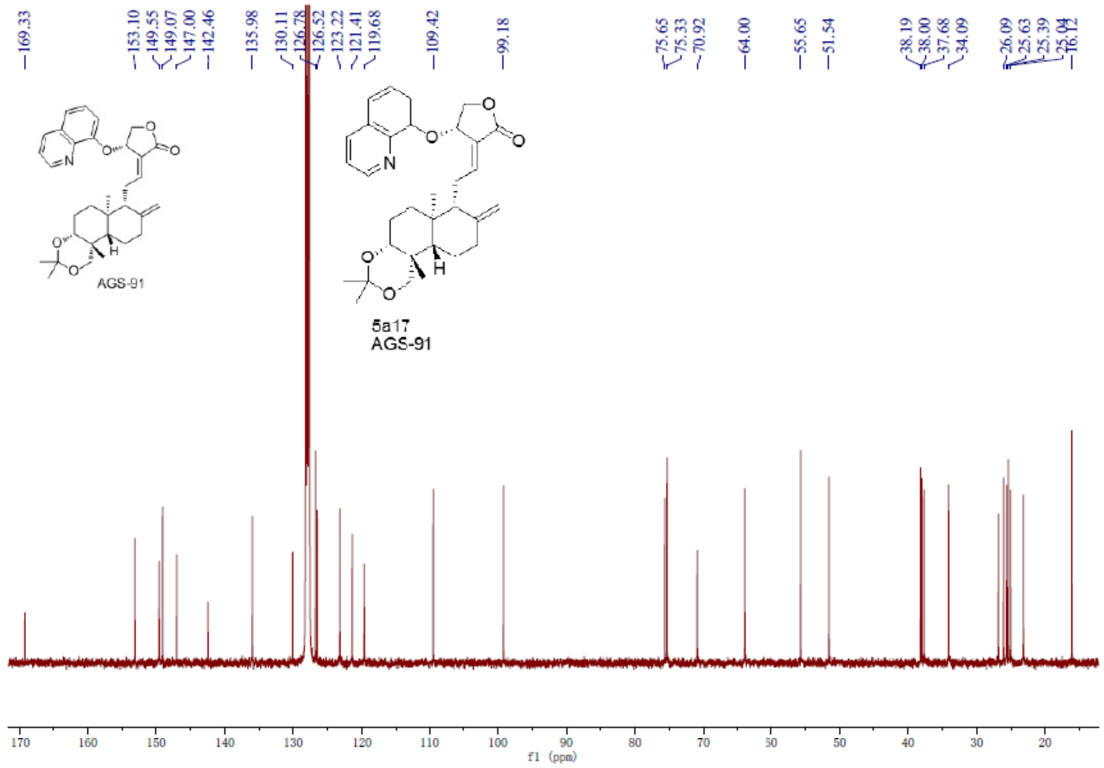

^{1}H NMR of **5b17**

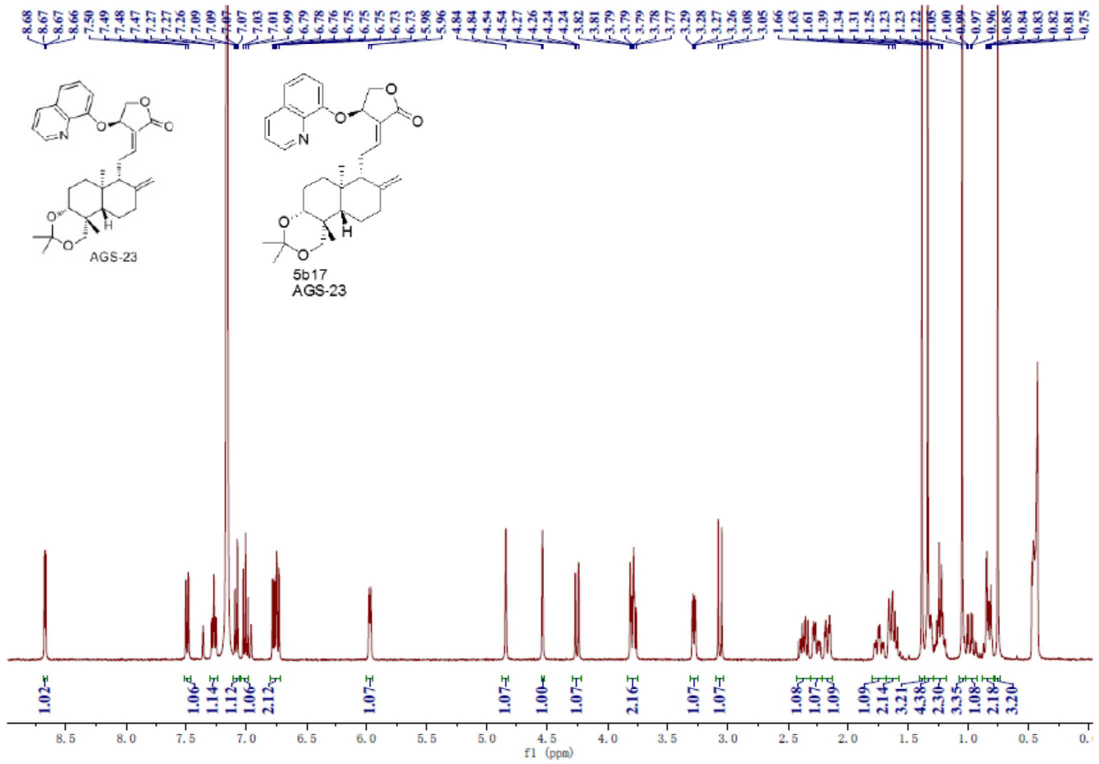

^{13}C NMR of **5b17**

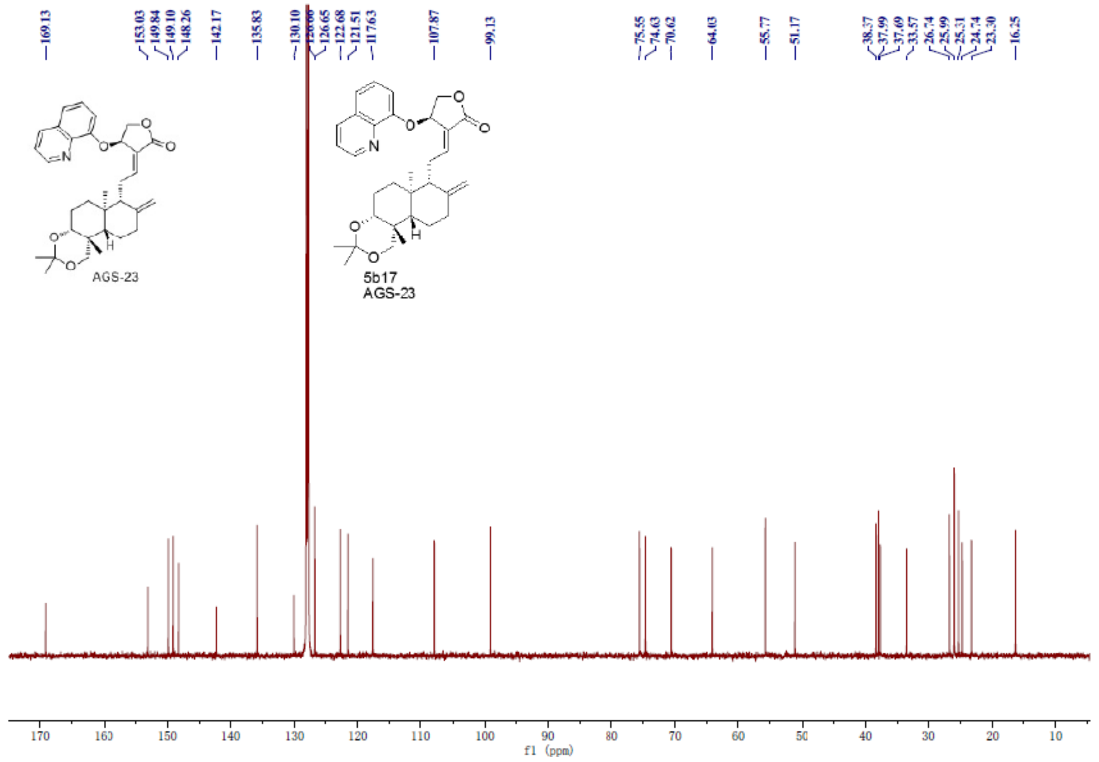

^{1}H NMR of **6a1**

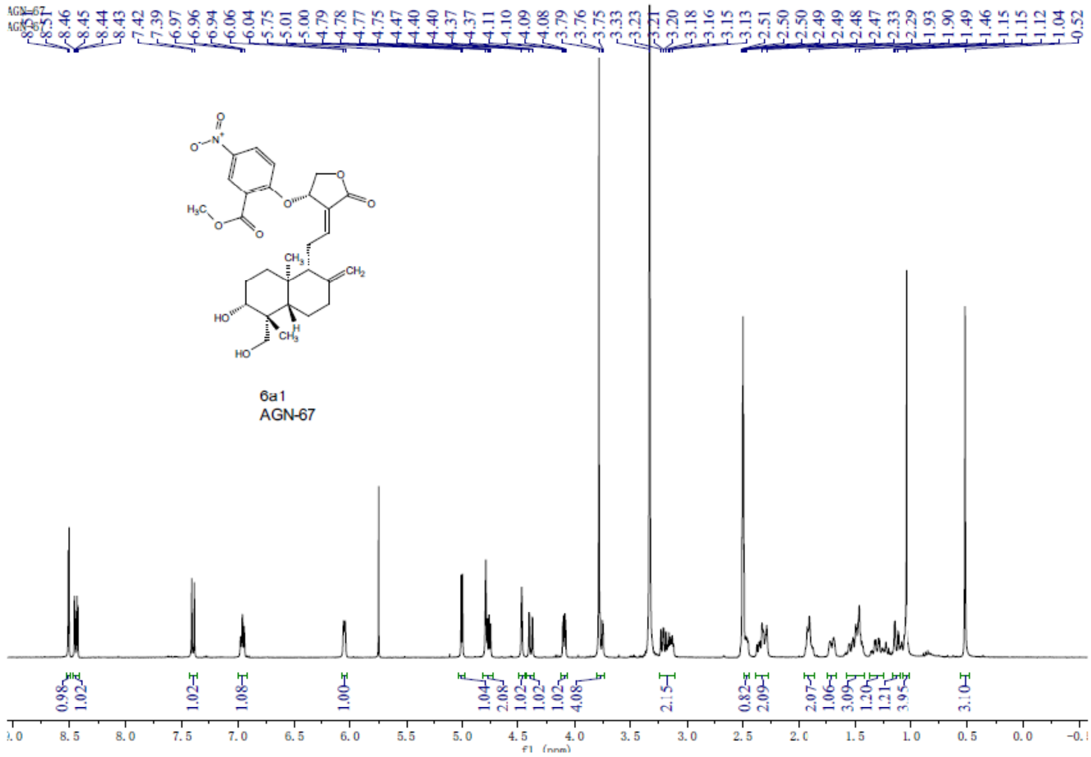

^{13}C NMR of **6a1**

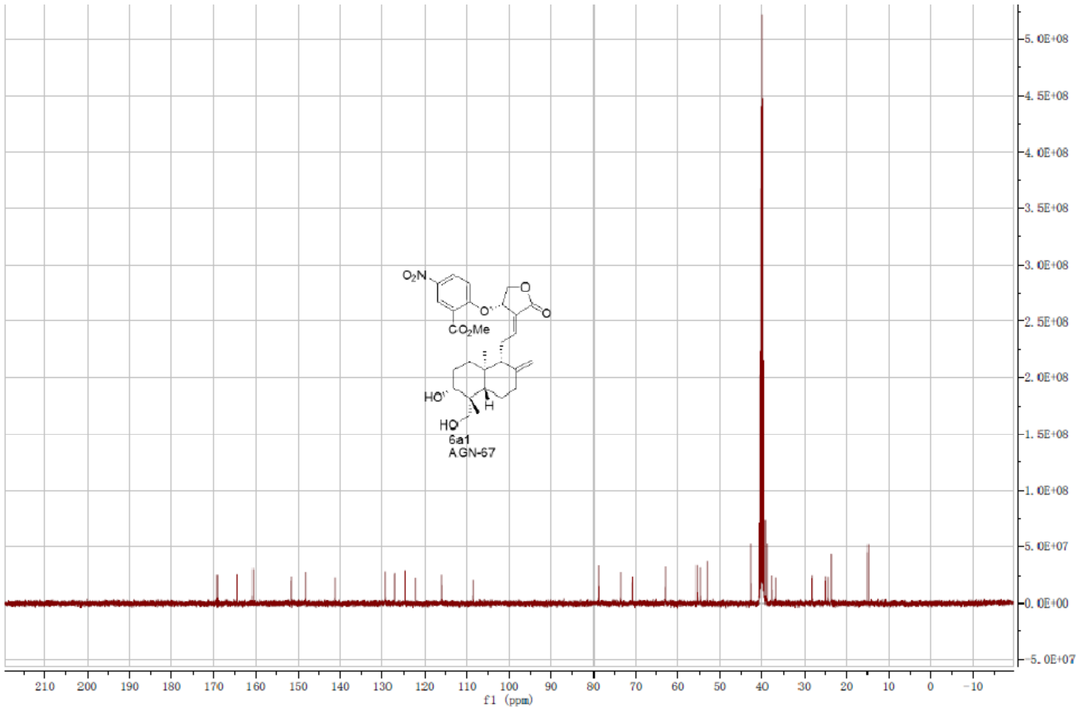

^{1}H NMR of **6b1**

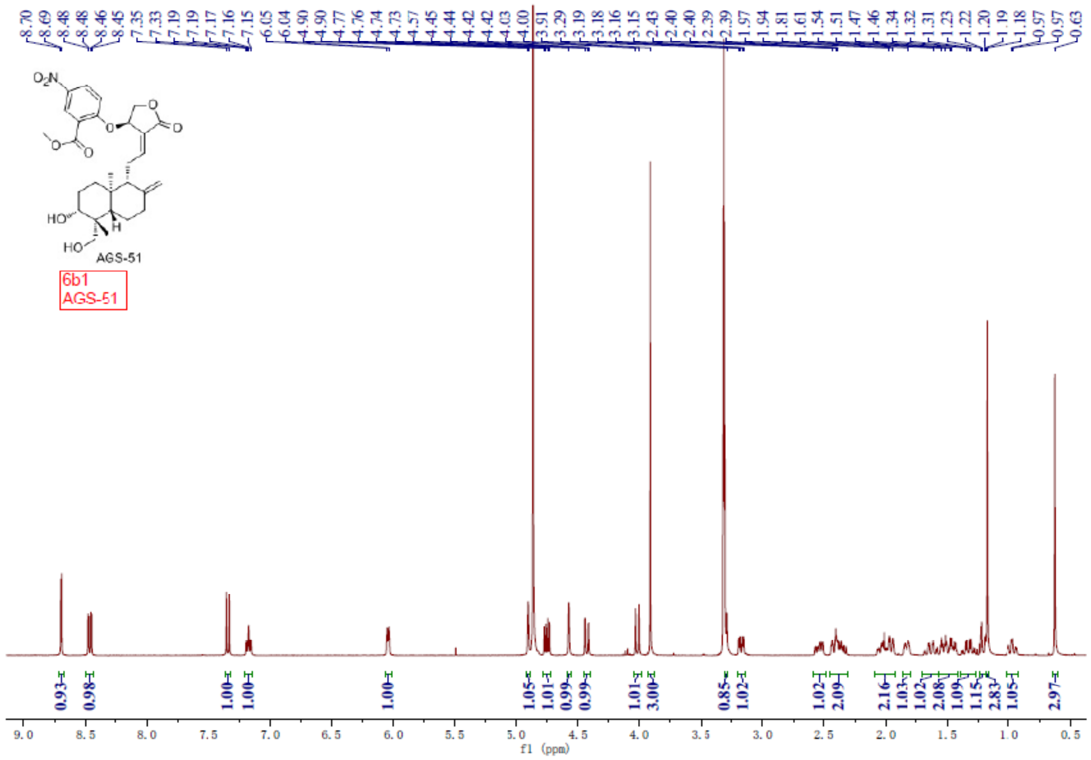

^{13}C NMR of **6b1**

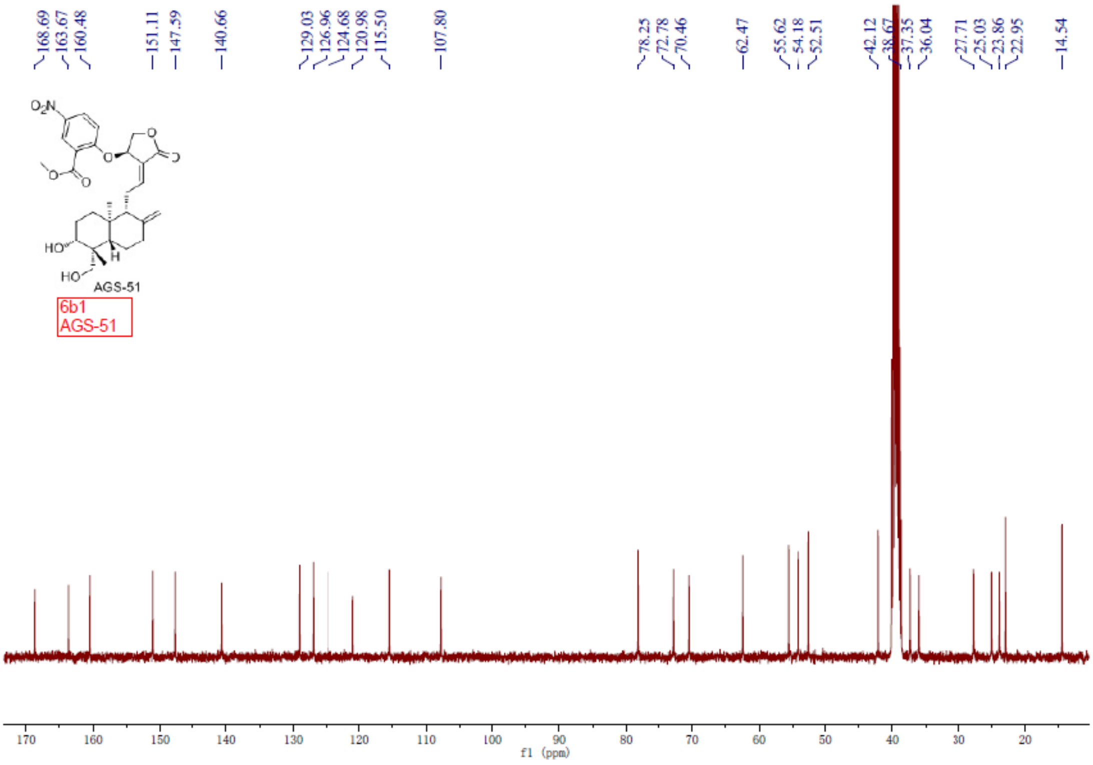

^{1}H NMR of **6a2**

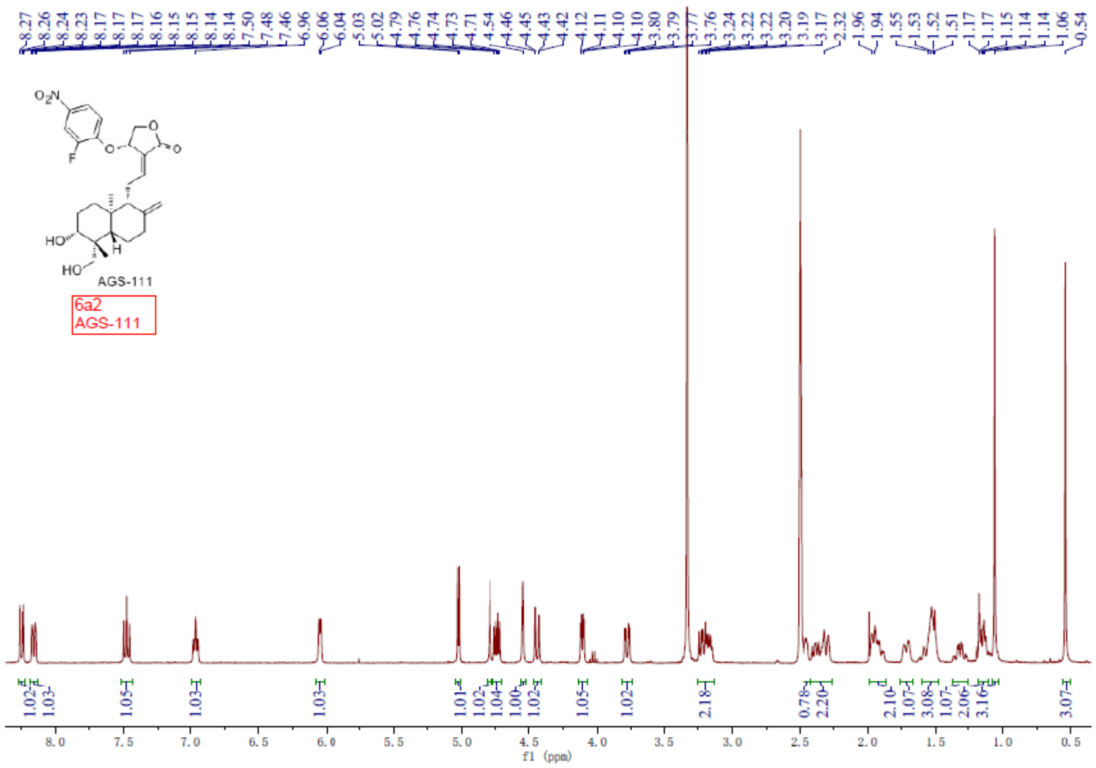

^{13}C NMR of **6a2**

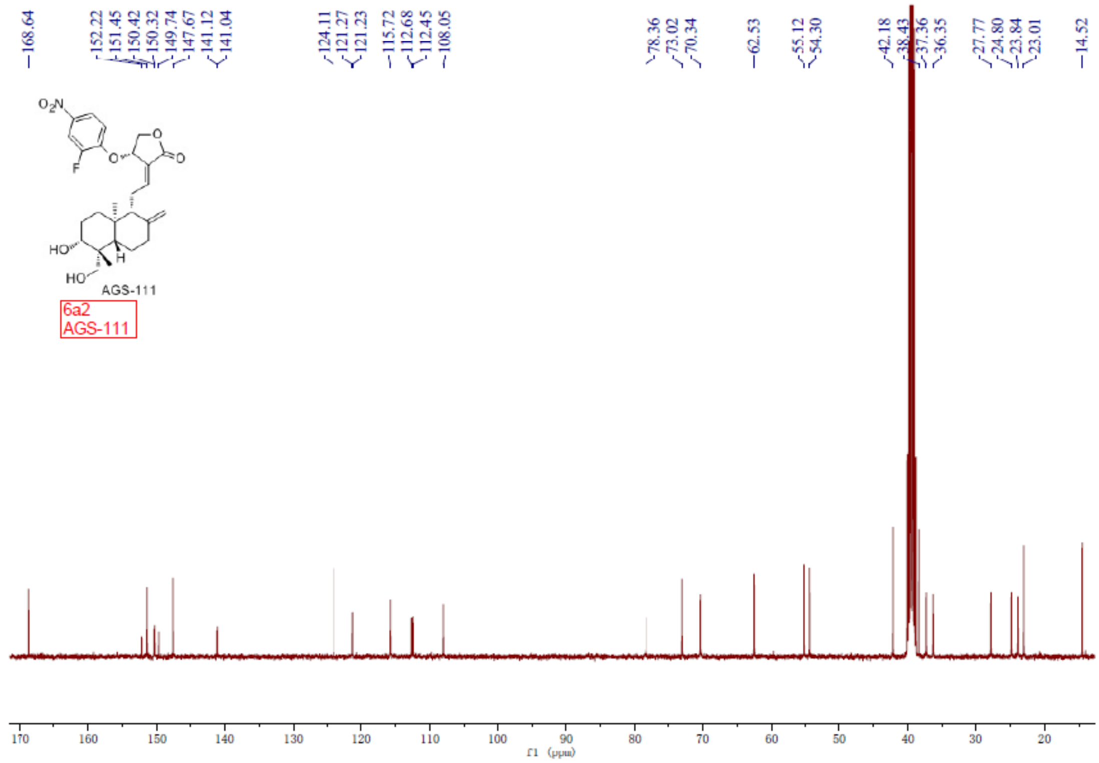

^{1}H NMR of **6b2**

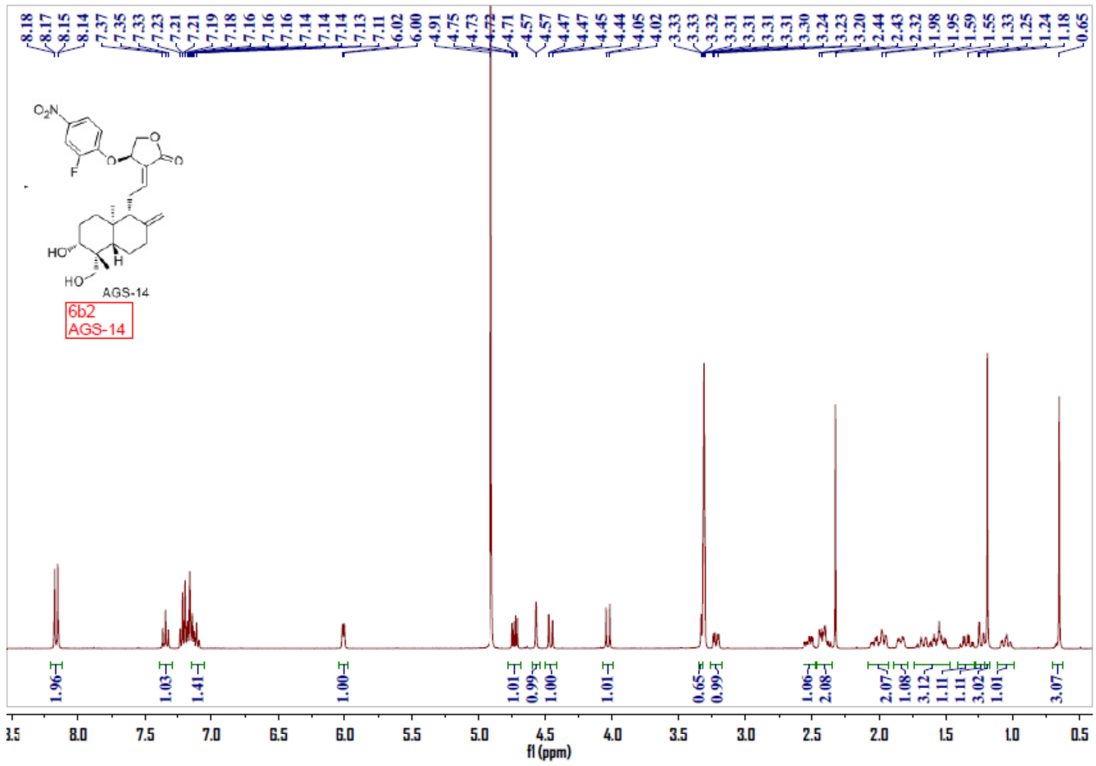

^{13}C NMR of **6b2**

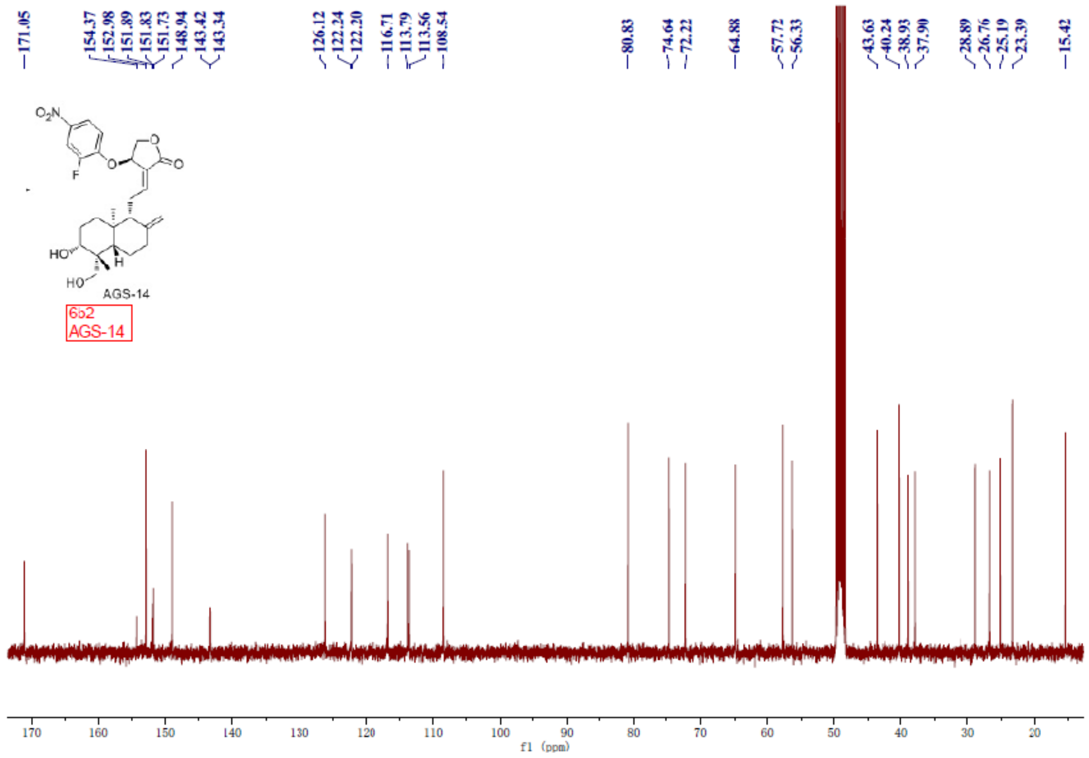

^{1}H NMR of **6a3**

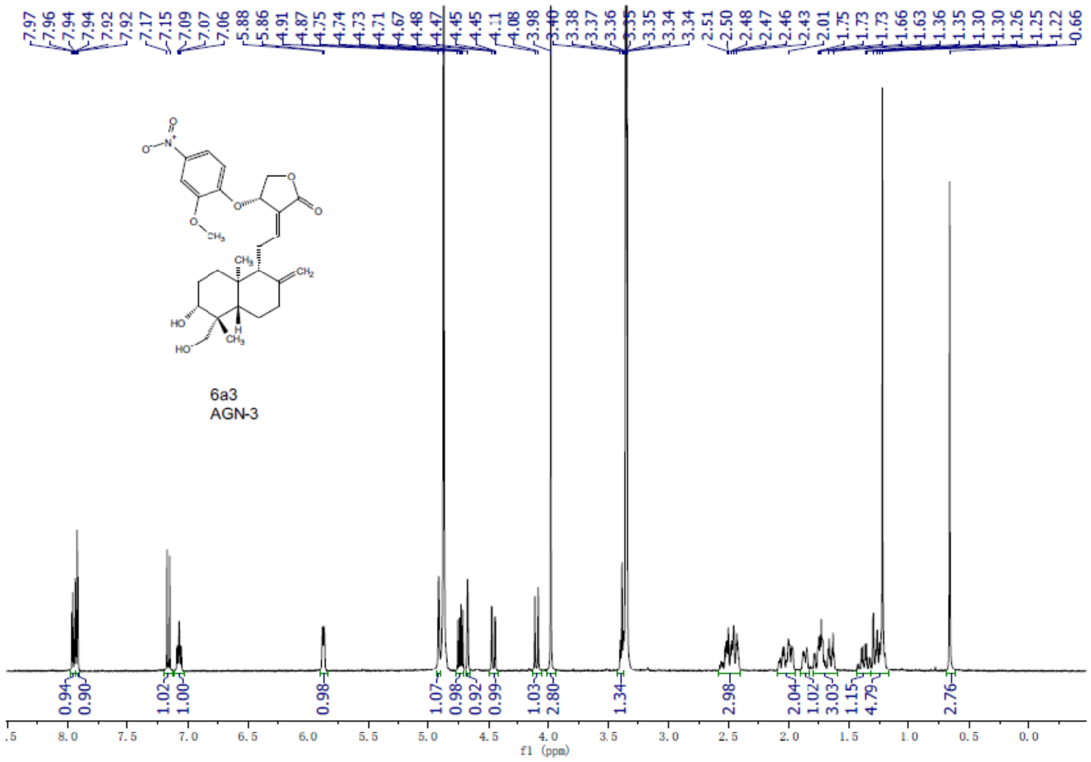

^{13}C NMR of **6a3**

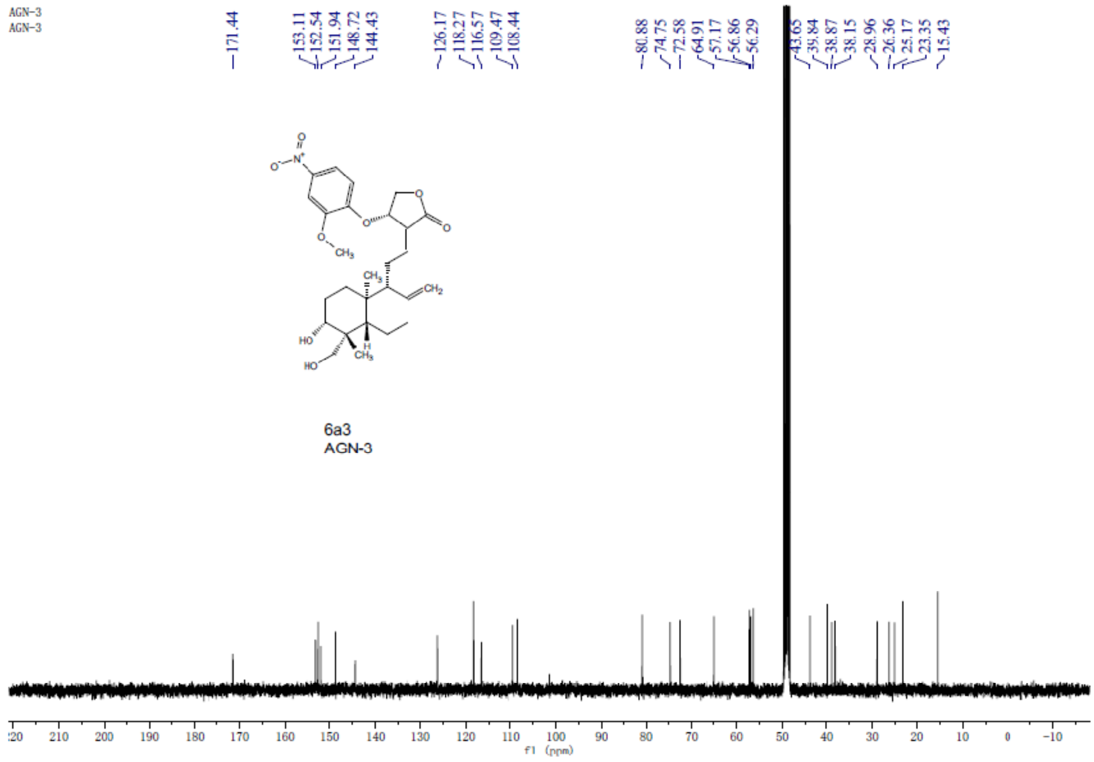

^{1}H NMR of **6b3**

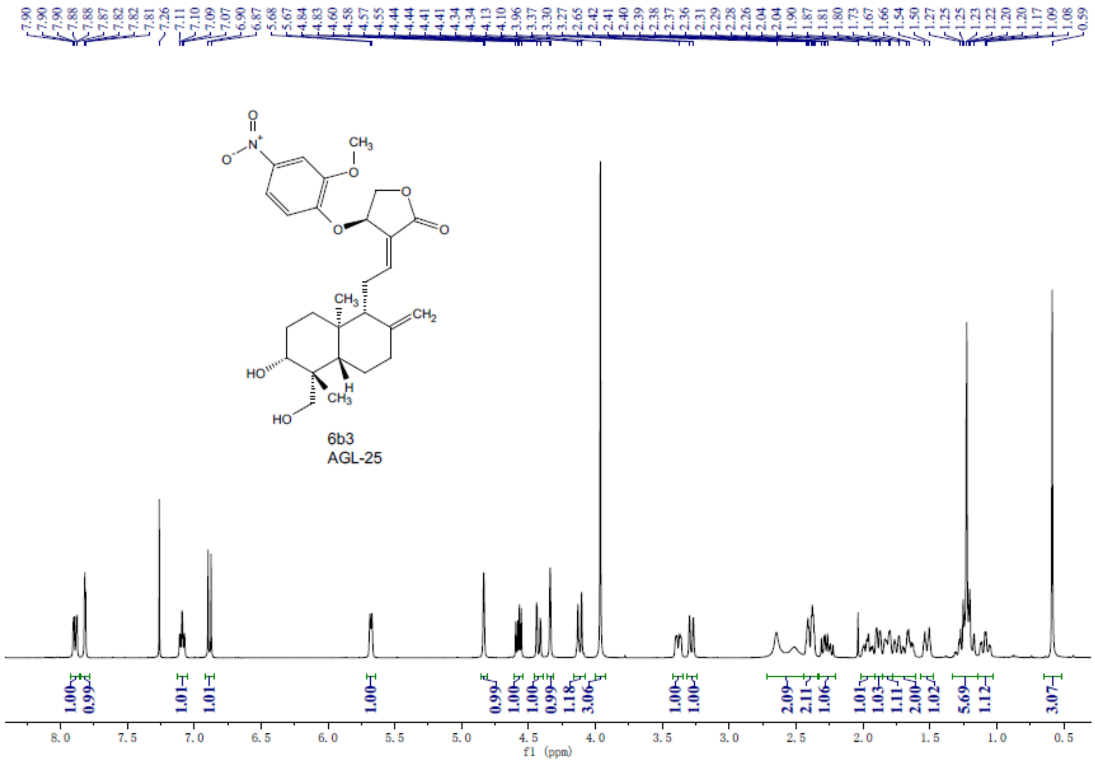

^{13}C NMR of **6b3**

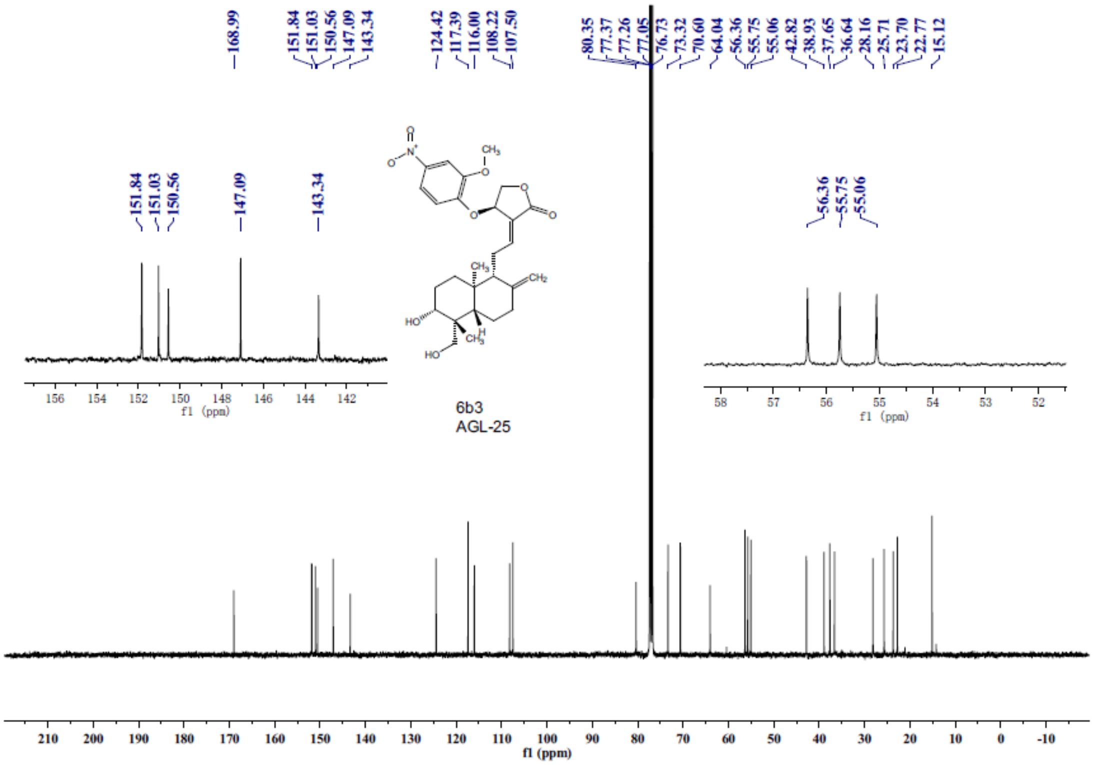

^{1}H NMR of **6a4**

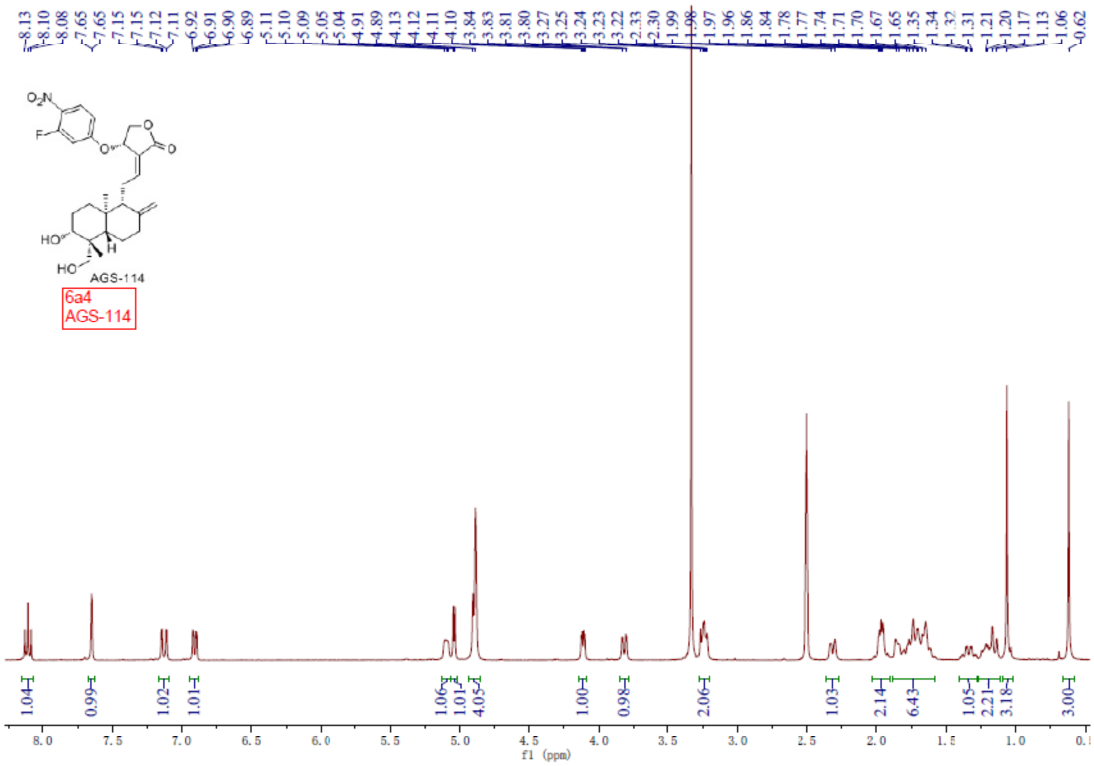

^{13}C NMR of **6a4**

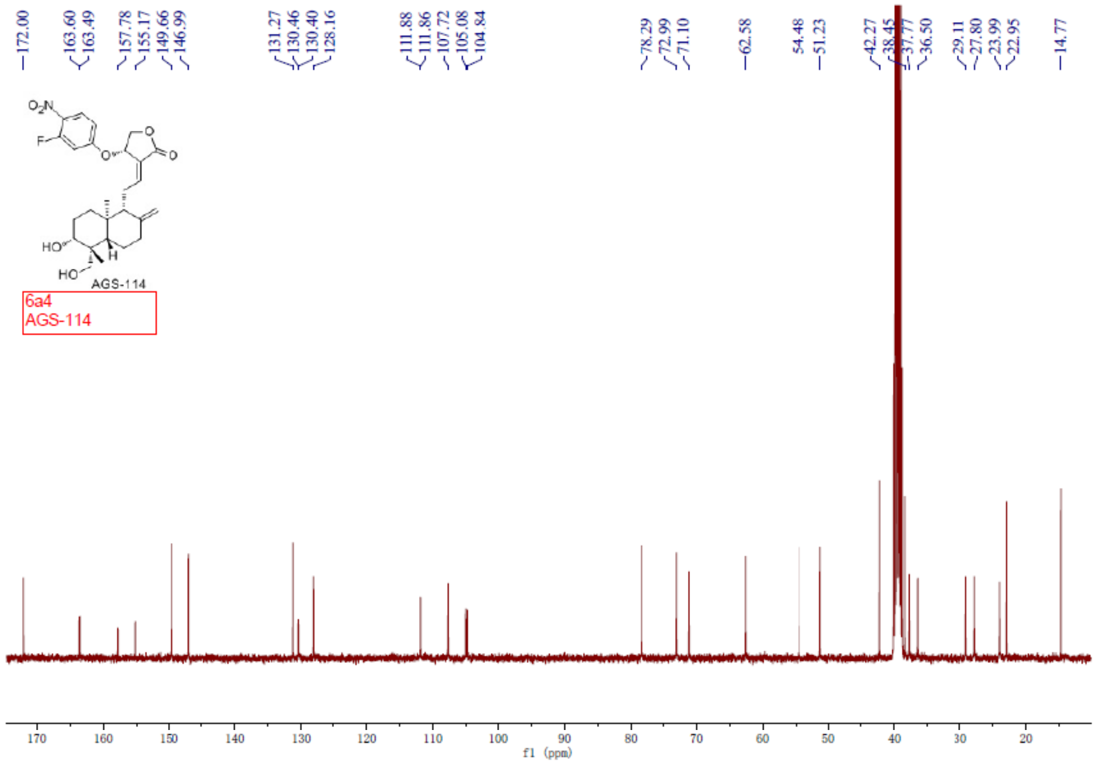

^{1}H NMR of **6b4**

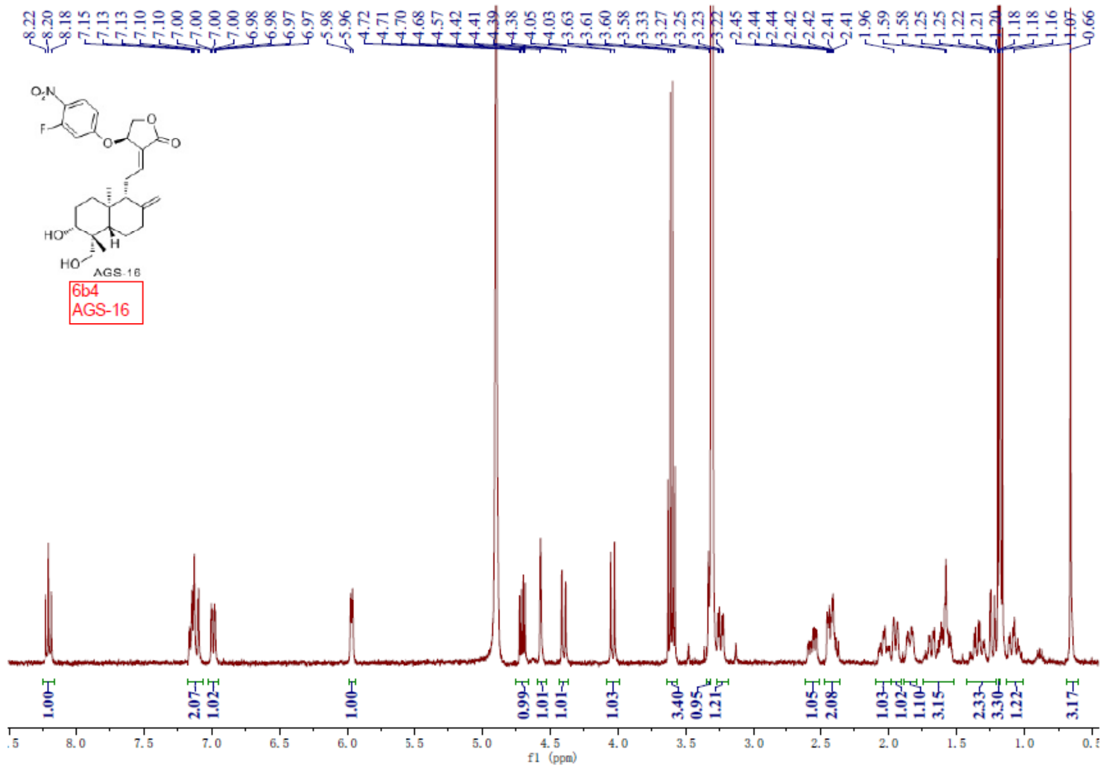

^{13}C NMR of **6b4**

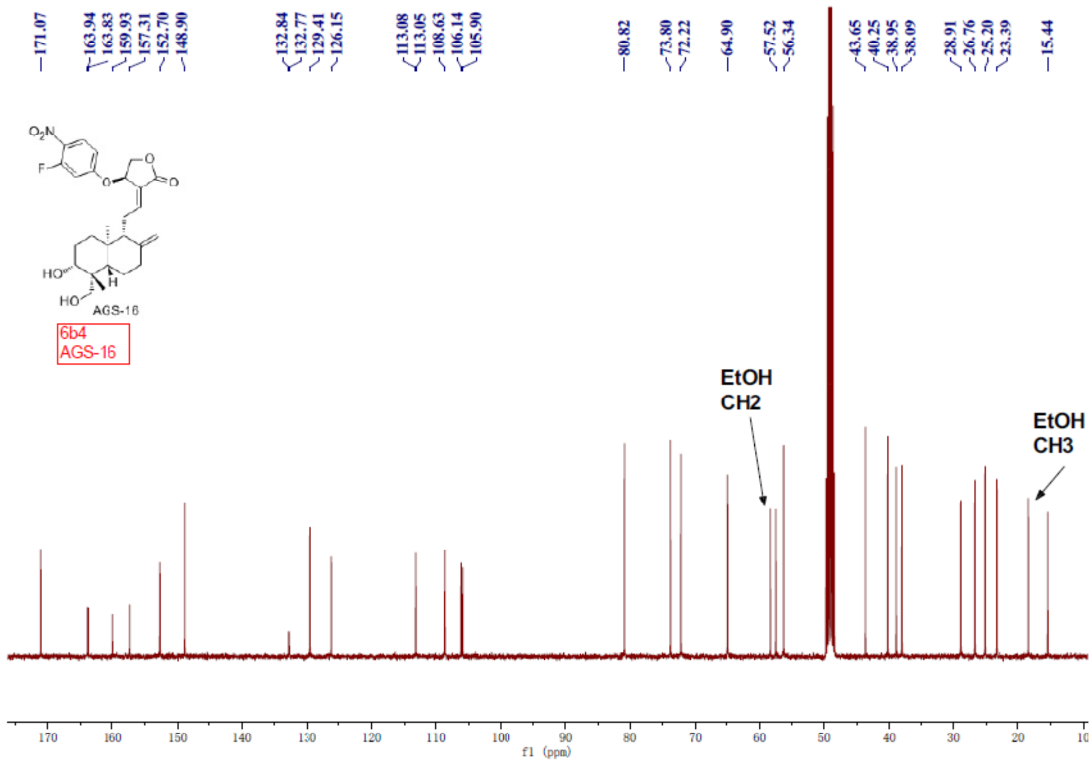

^{1}H NMR of **6a5**

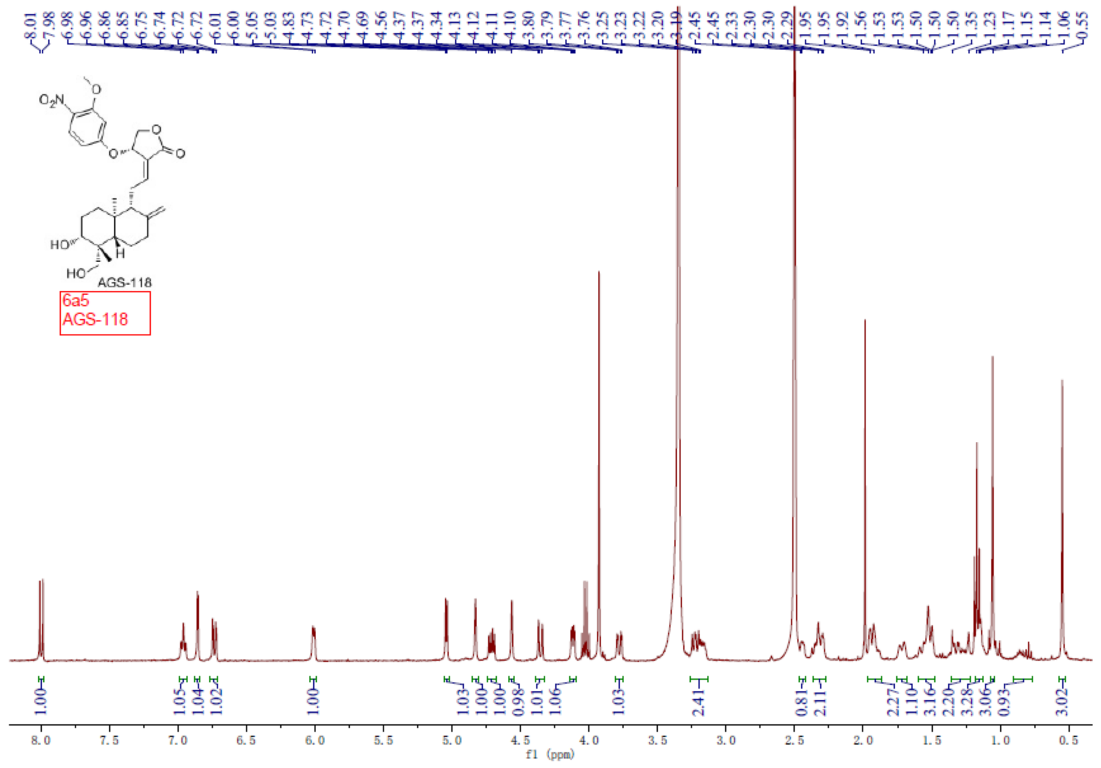

^{13}C NMR of **6a5**

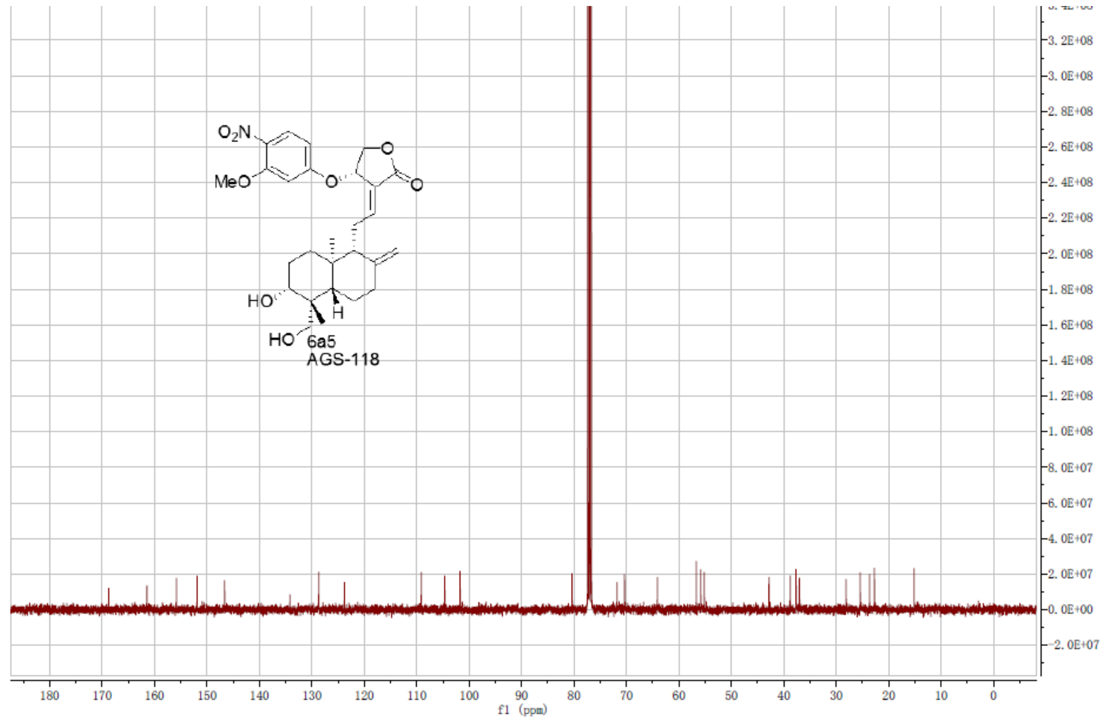

^{1}H NMR of **6b5**

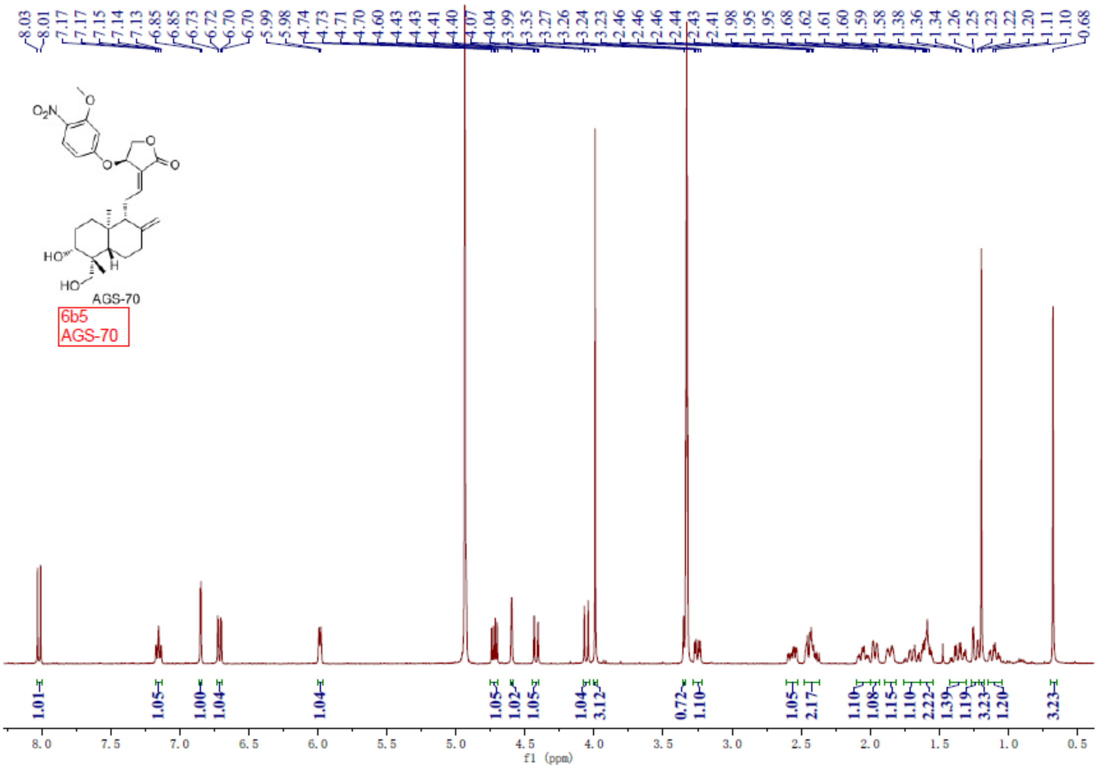

^{13}C NMR of **6b5**

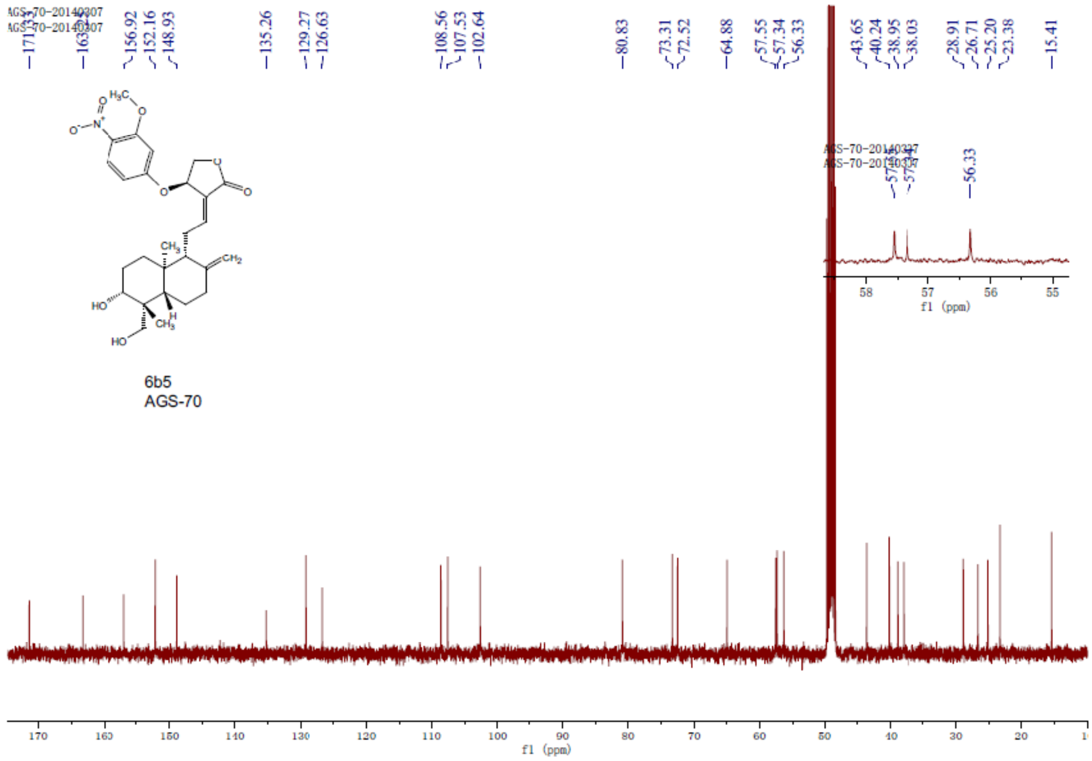

^{1}H NMR of **6b6**

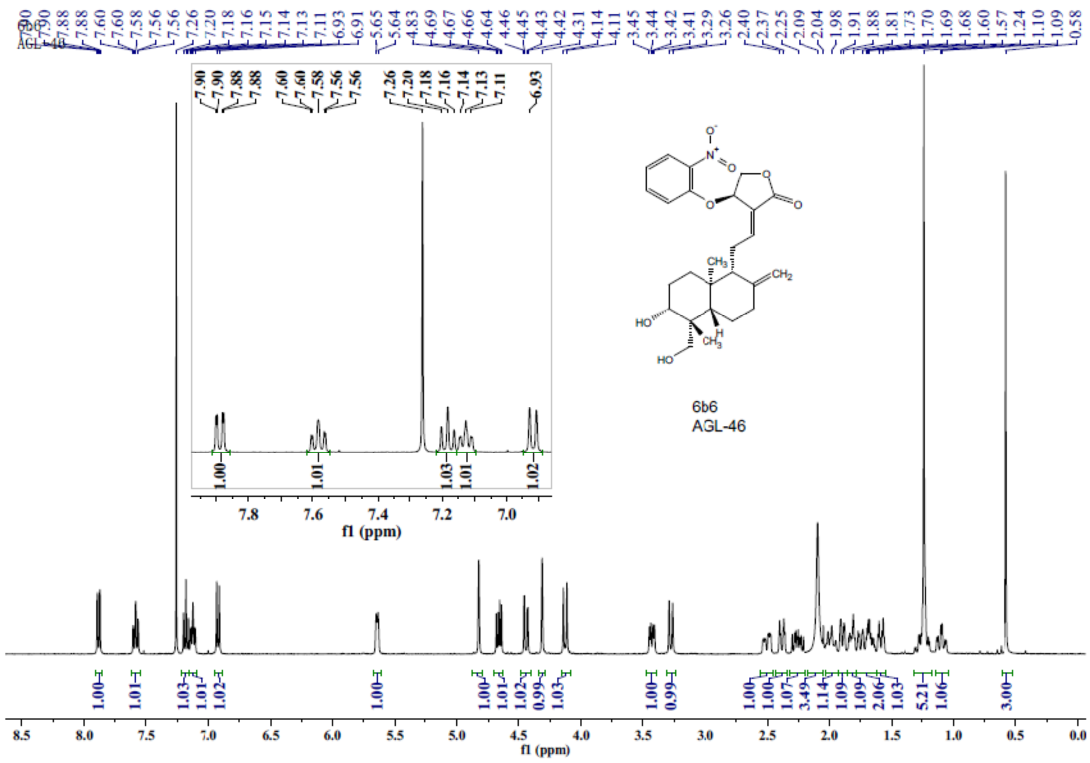

^{13}C NMR of **6b6**

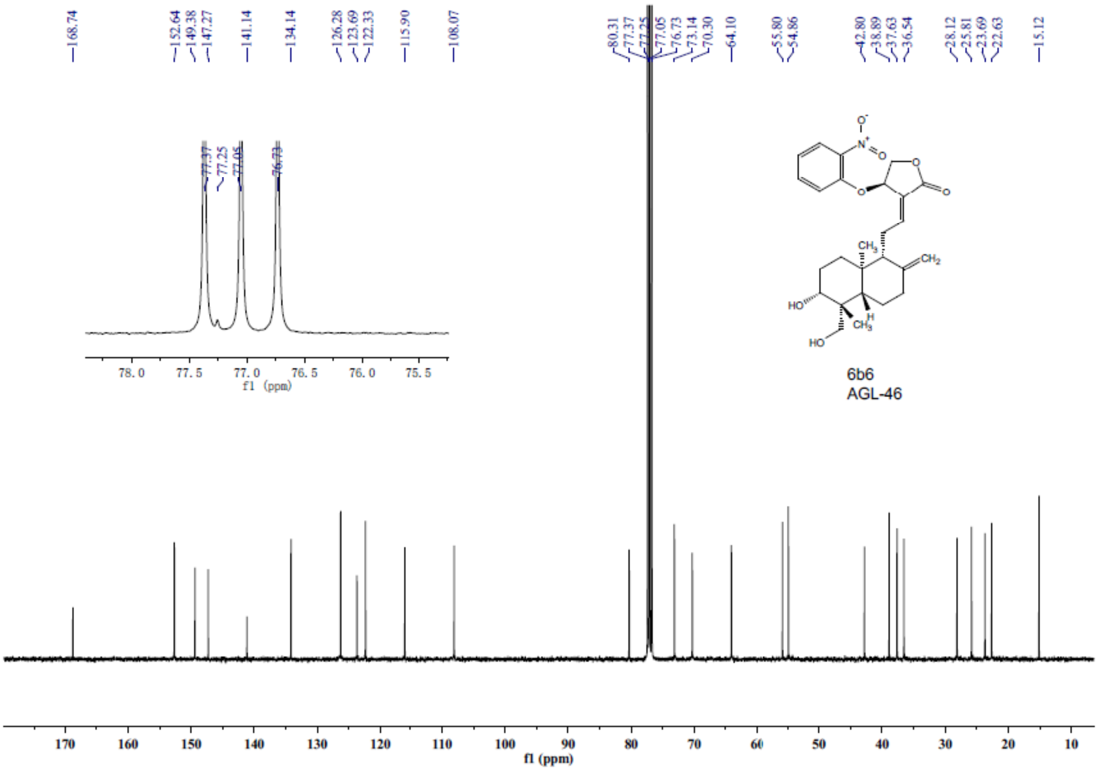

^{1}H NMR of **6b7**

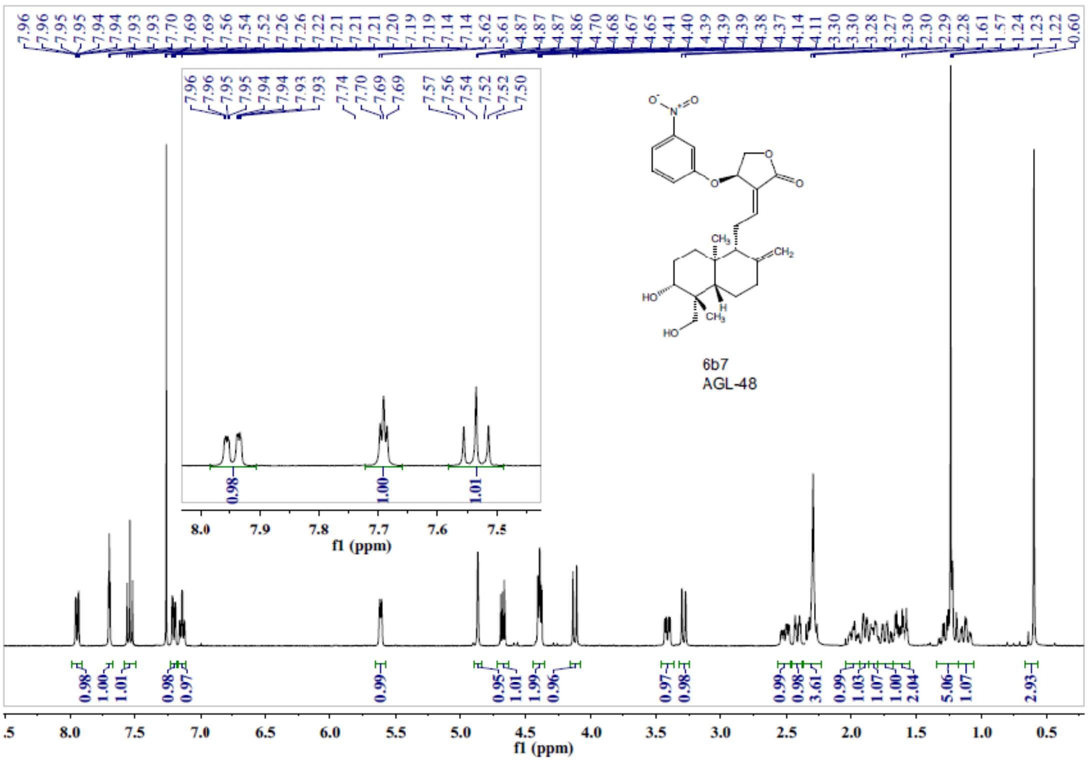

^{13}C NMR of **6b7**

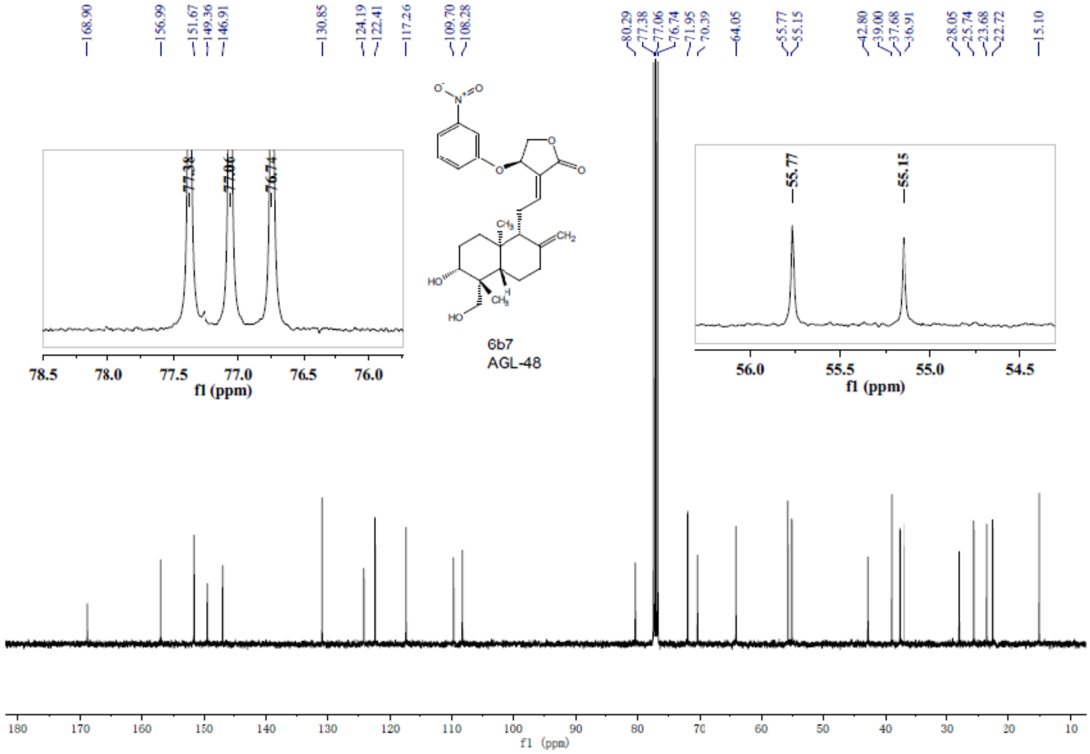

^{1}H NMR of **6a8**

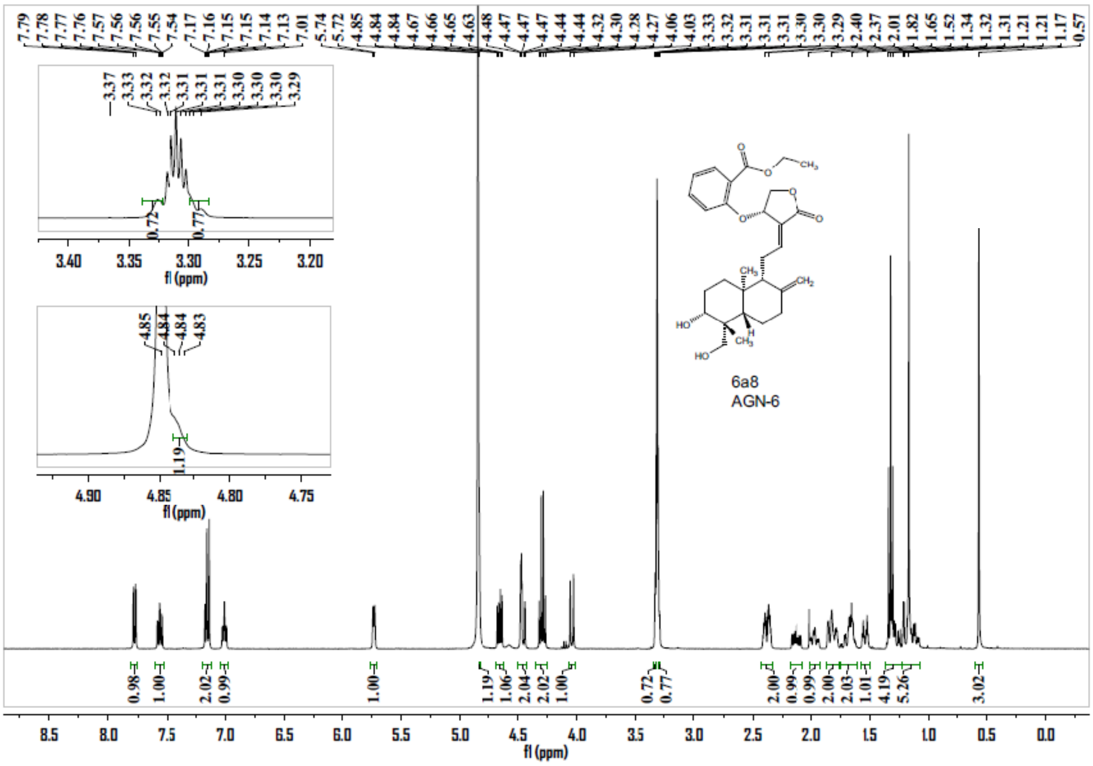

^{13}C NMR of **6a8**

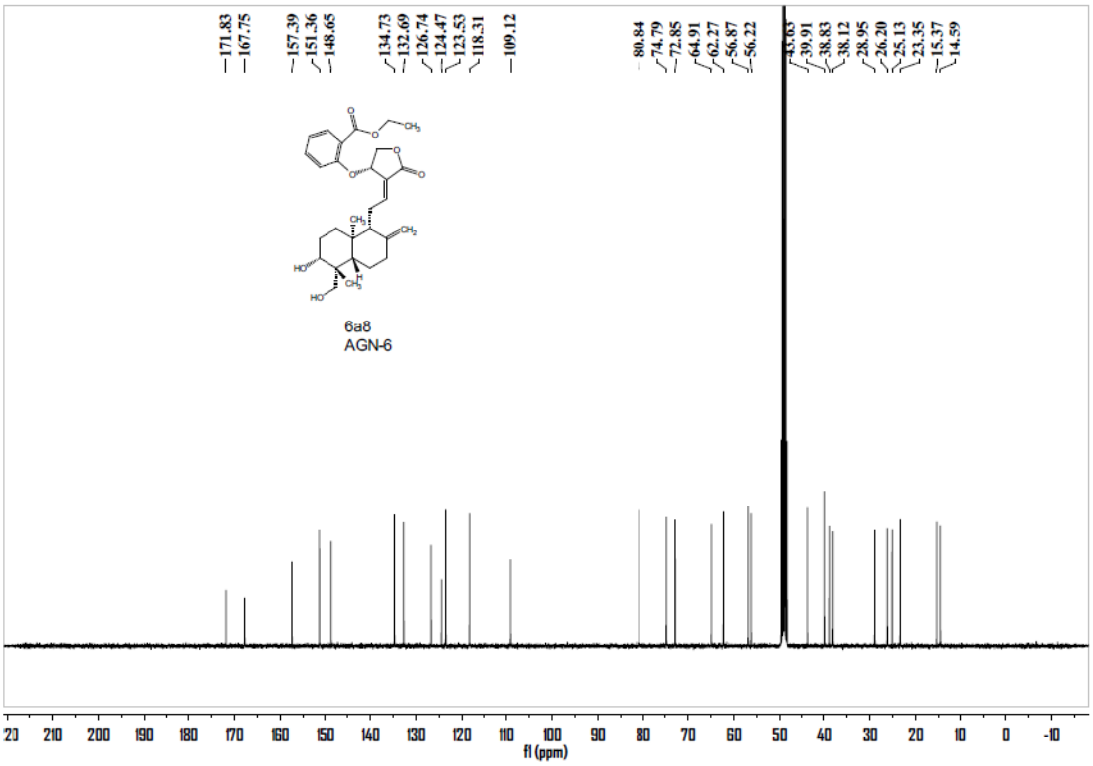

^{1}H NMR of **6b8**

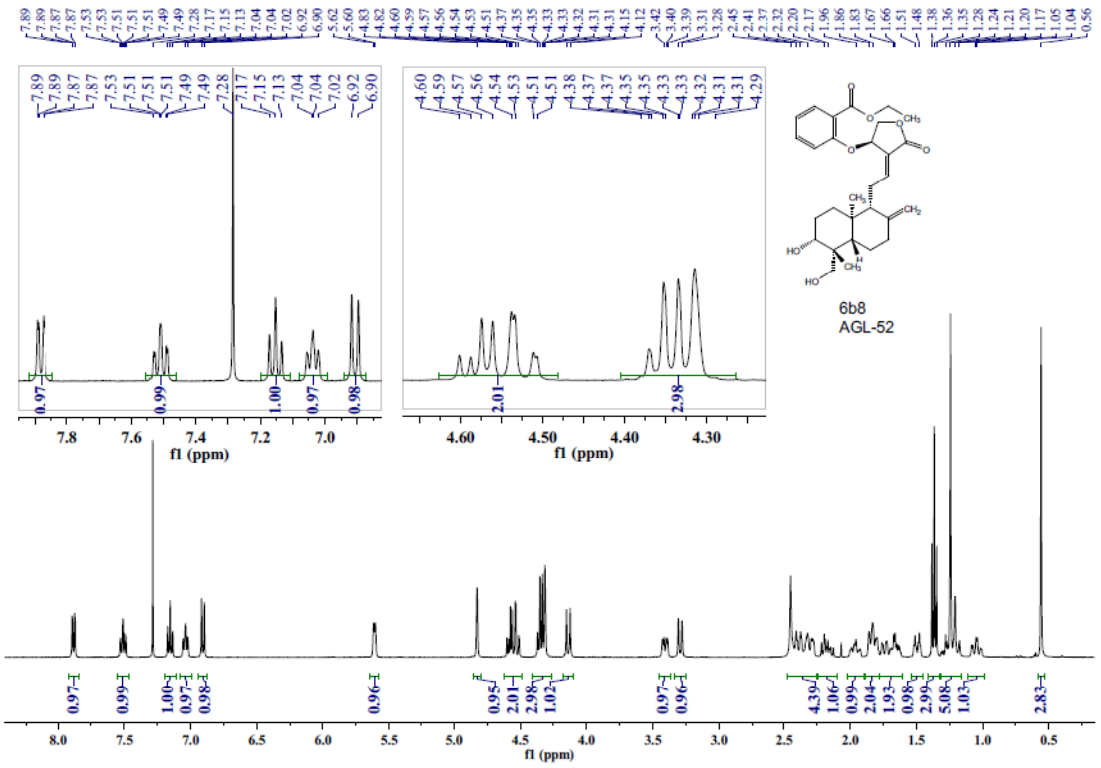

^{13}C NMR of **6b8**

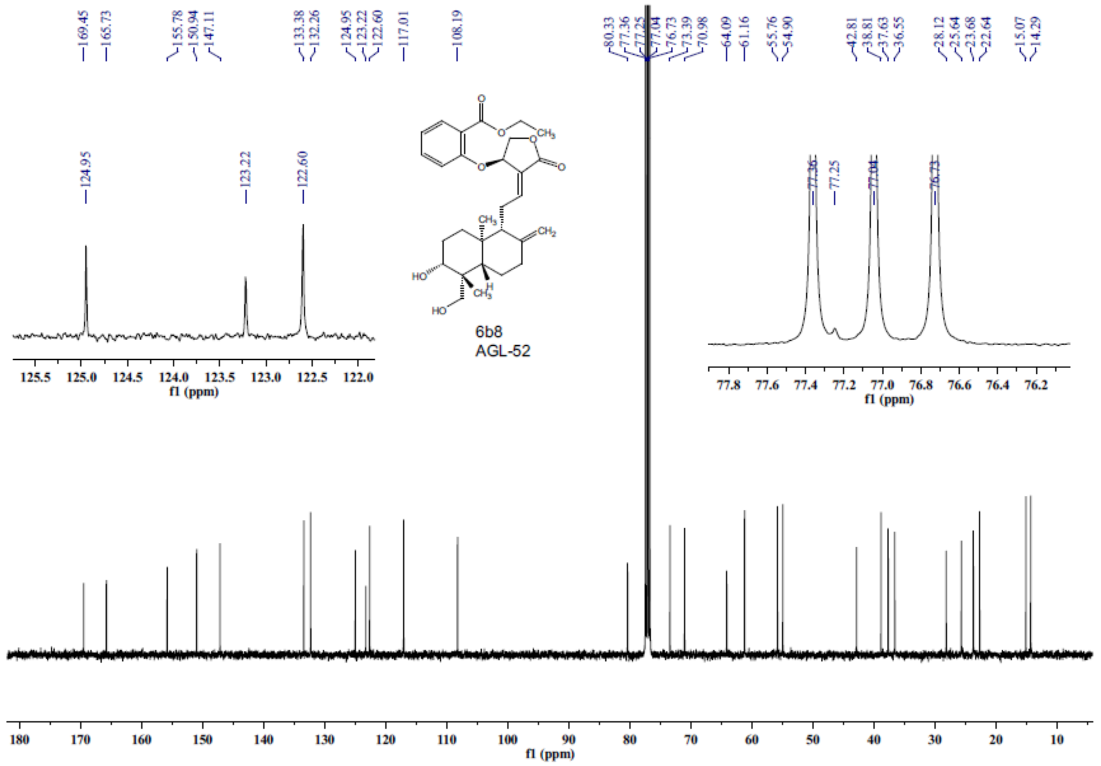

^{1}H NMR of **6a9**

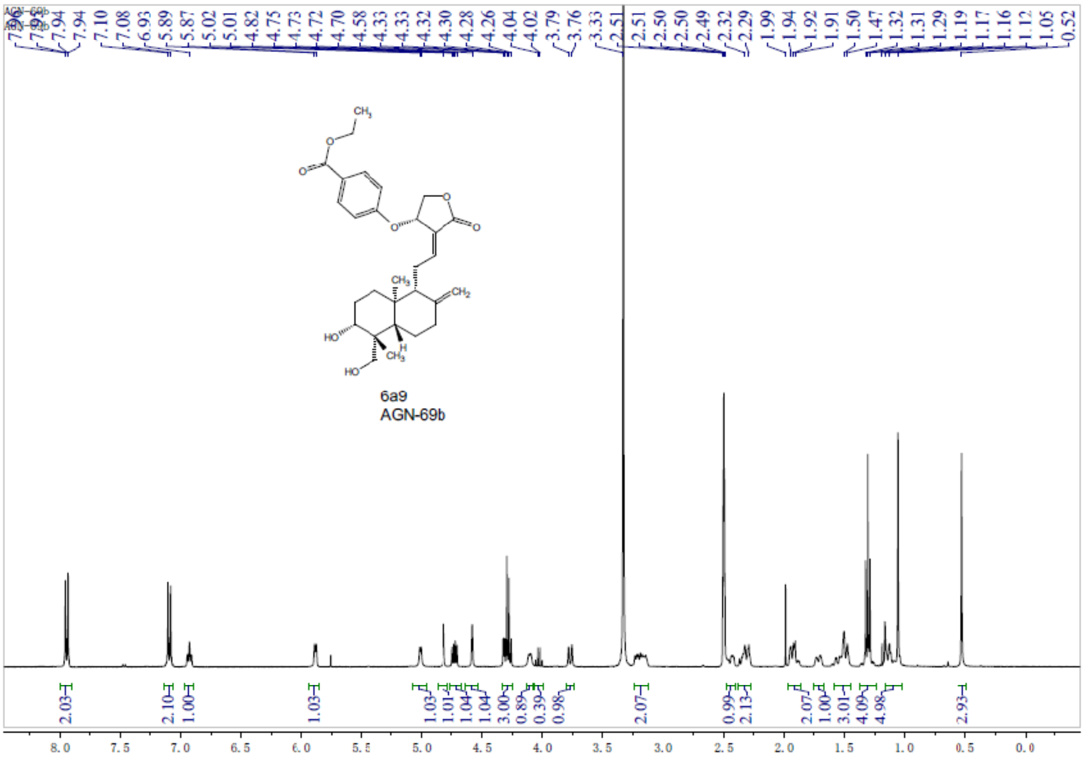

^{13}C NMR of **6a9**

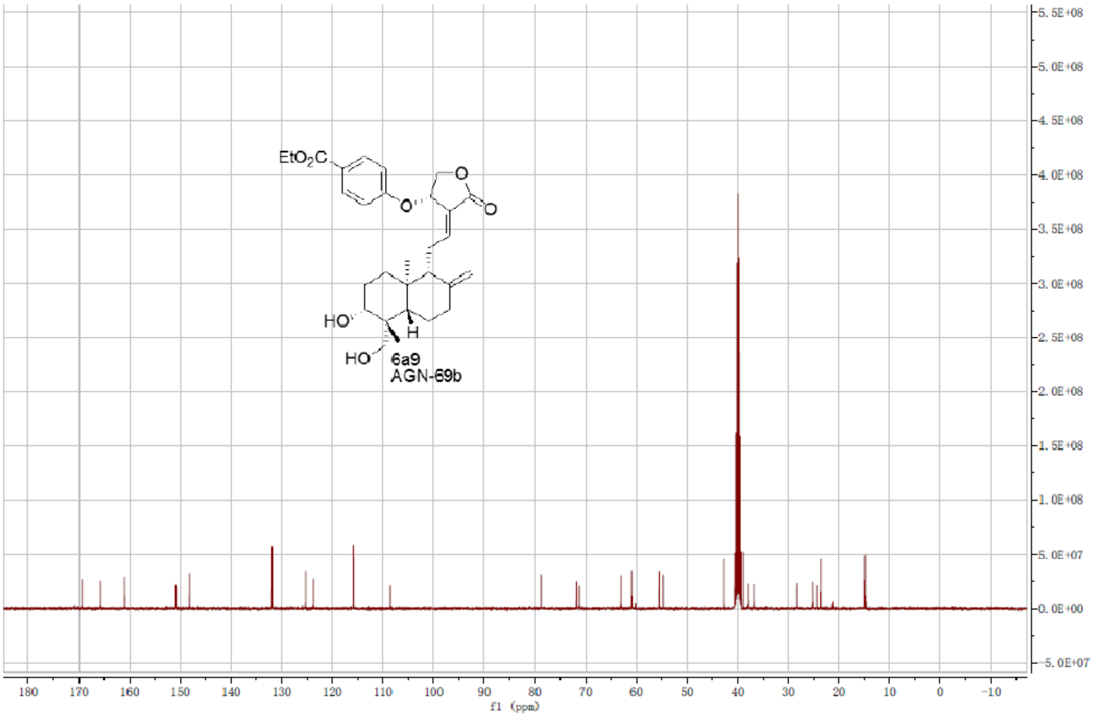

^{1}H NMR of **6b9**

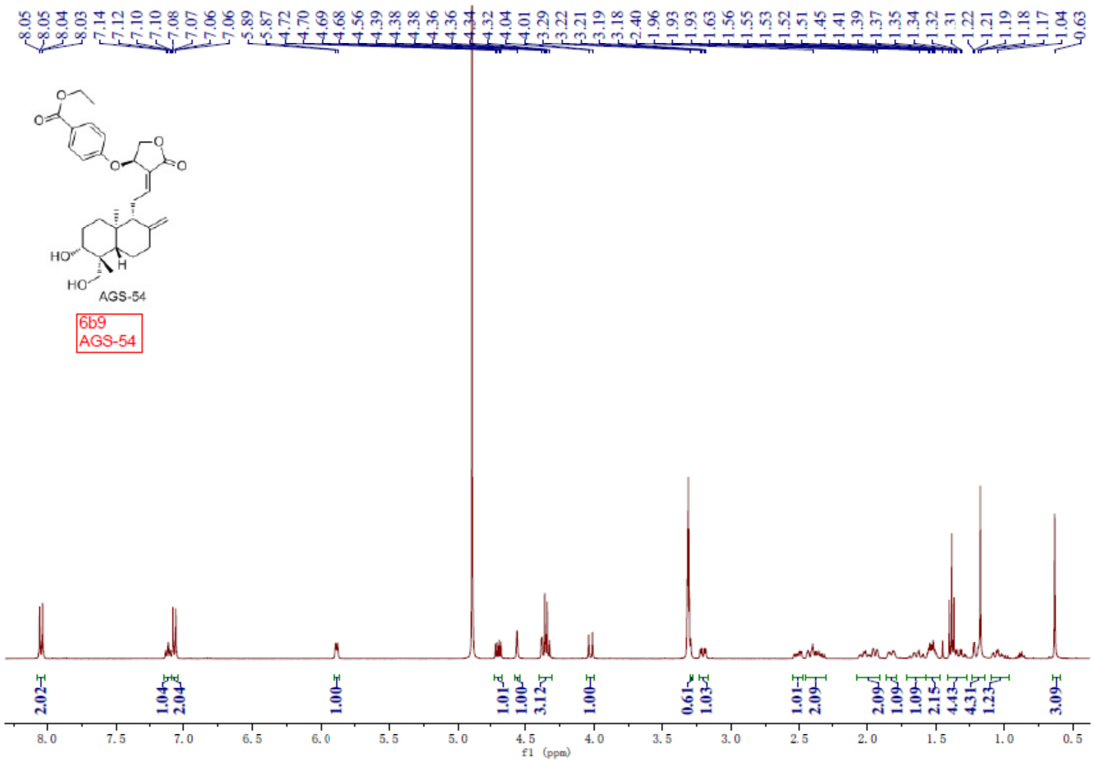

^{13}C NMR of **6b9**

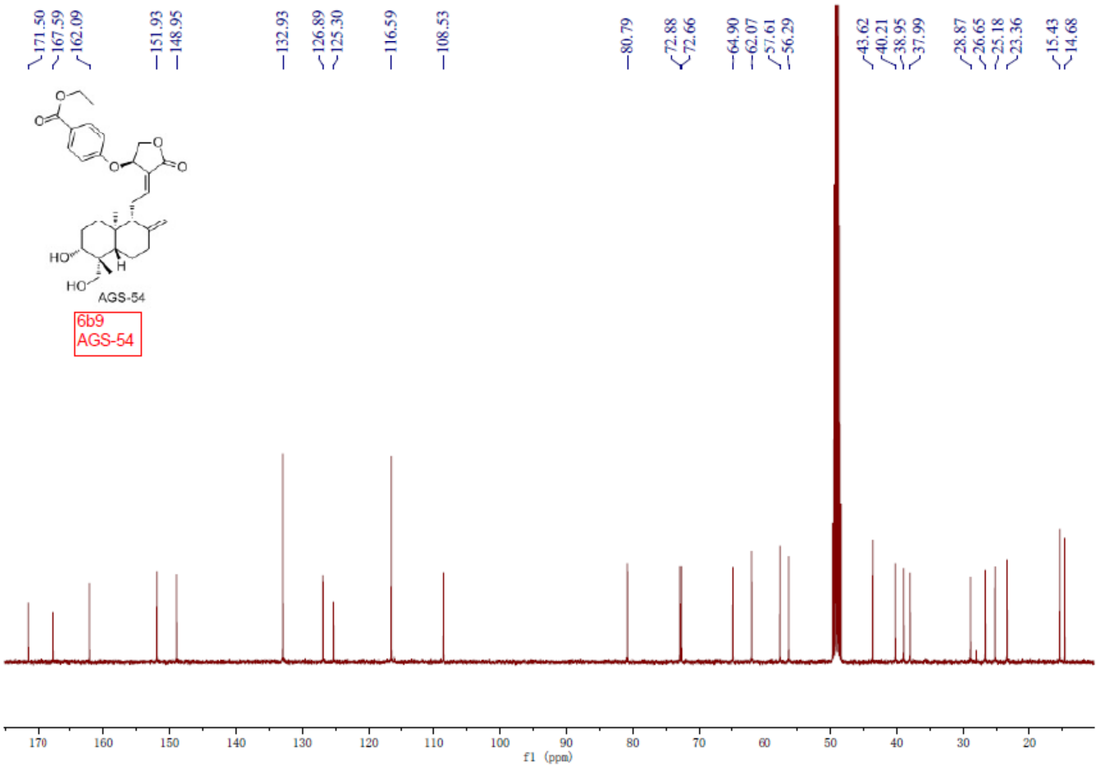

^{1}H NMR of **6b10**

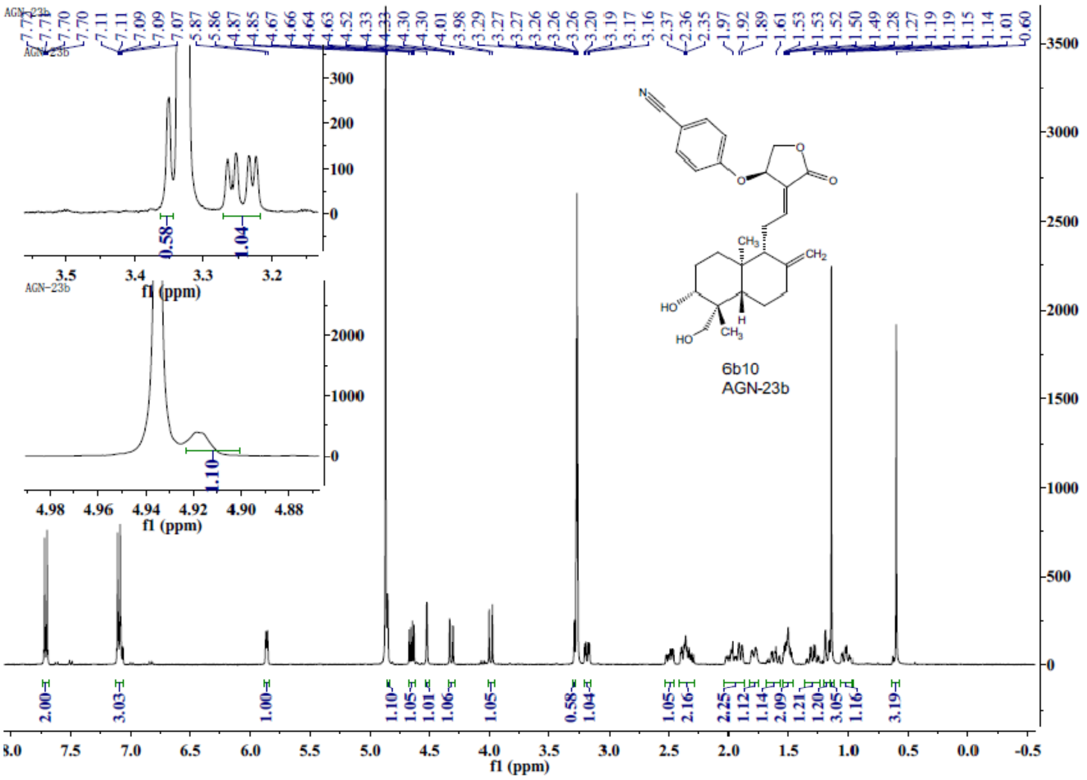

^{13}C NMR of **6b10**

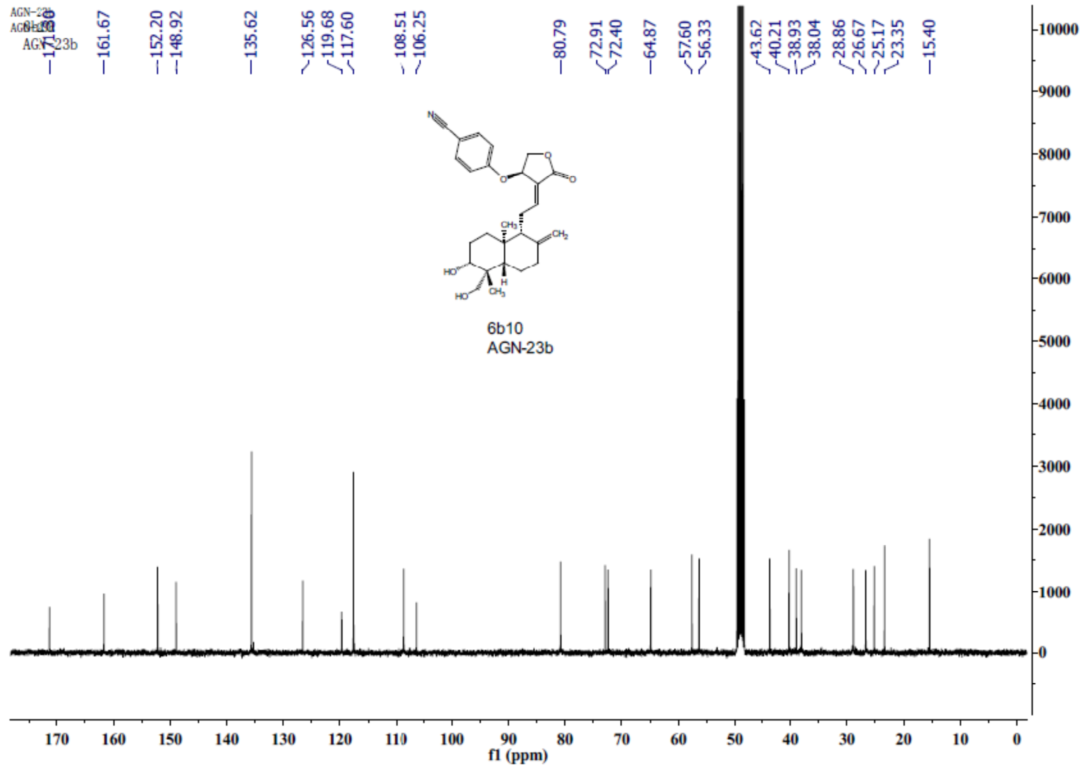

^{1}H NMR of **6b11**

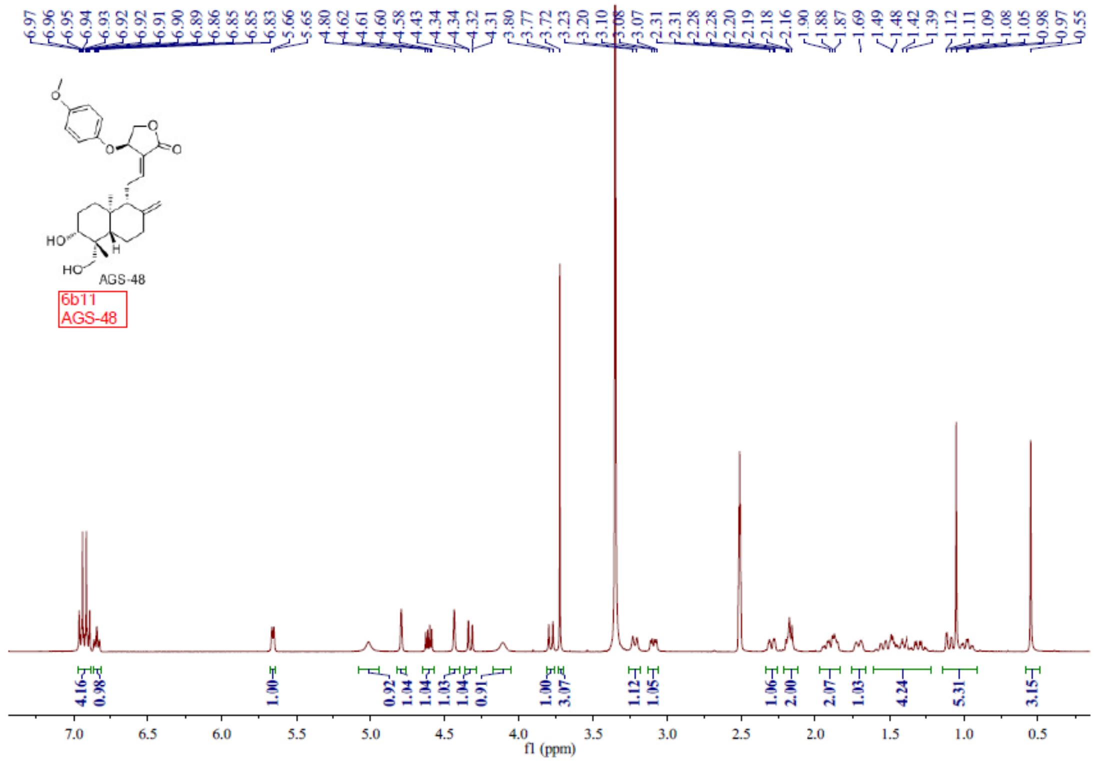

^{13}C NMR of **6b11**

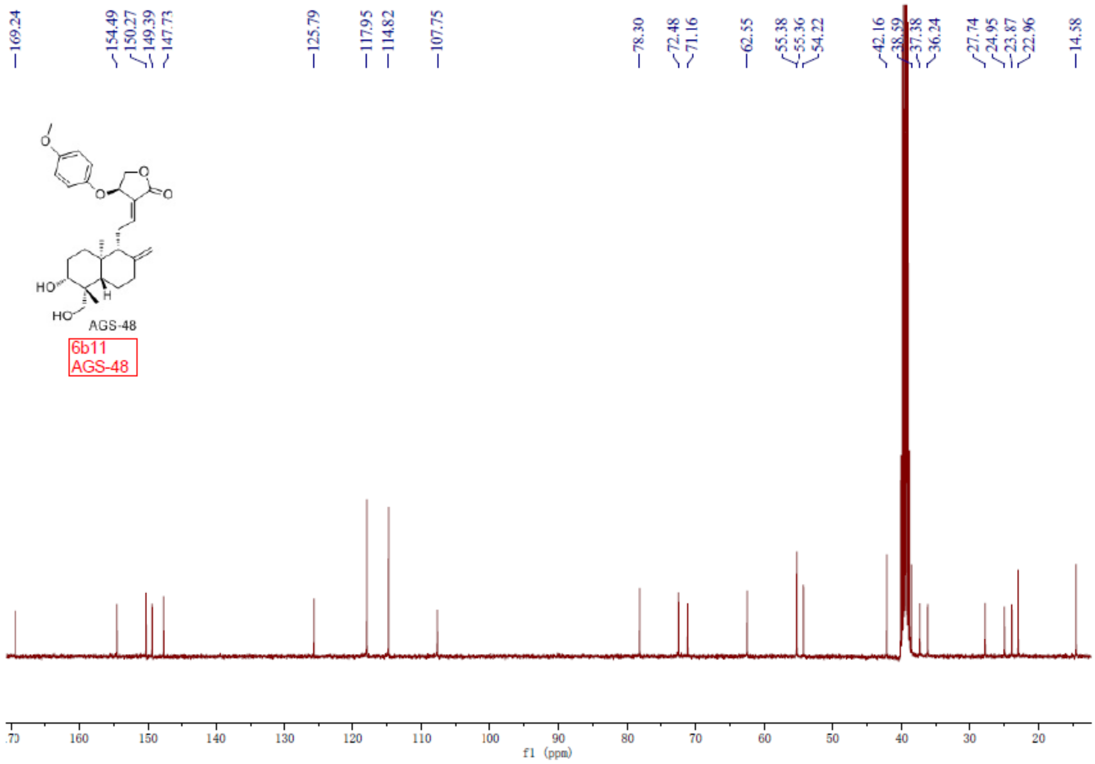

^{1}H NMR of **6a12**

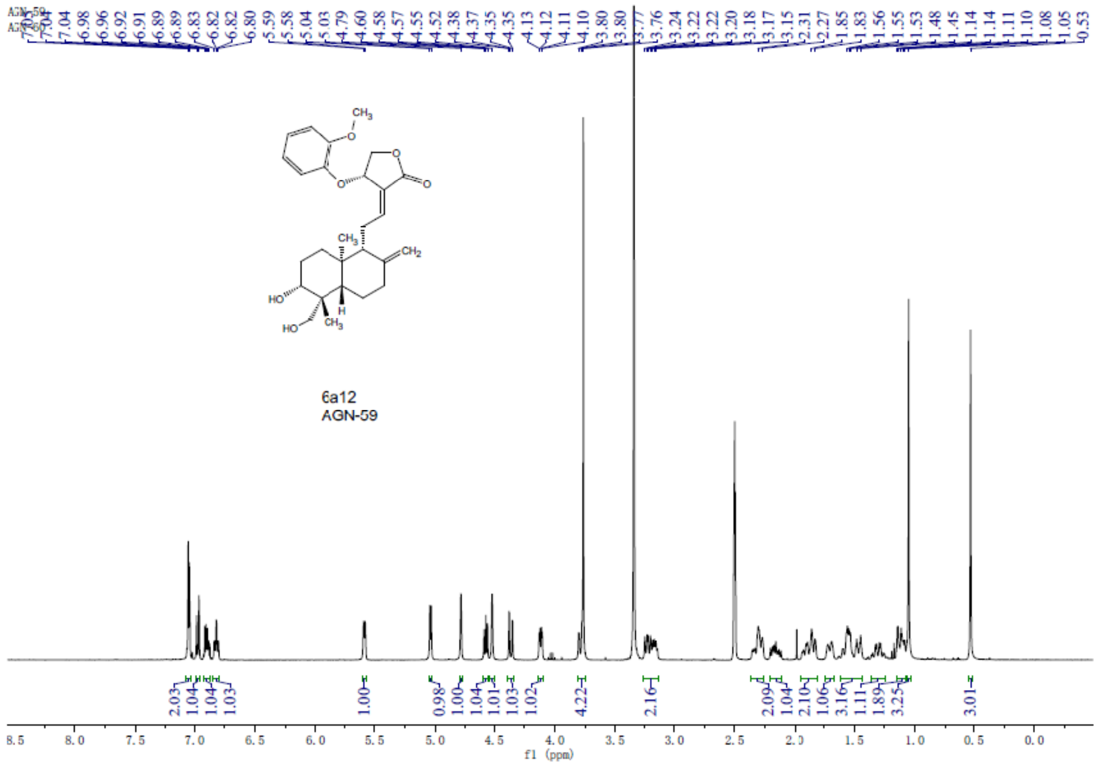

^{13}C NMR of **6a12**

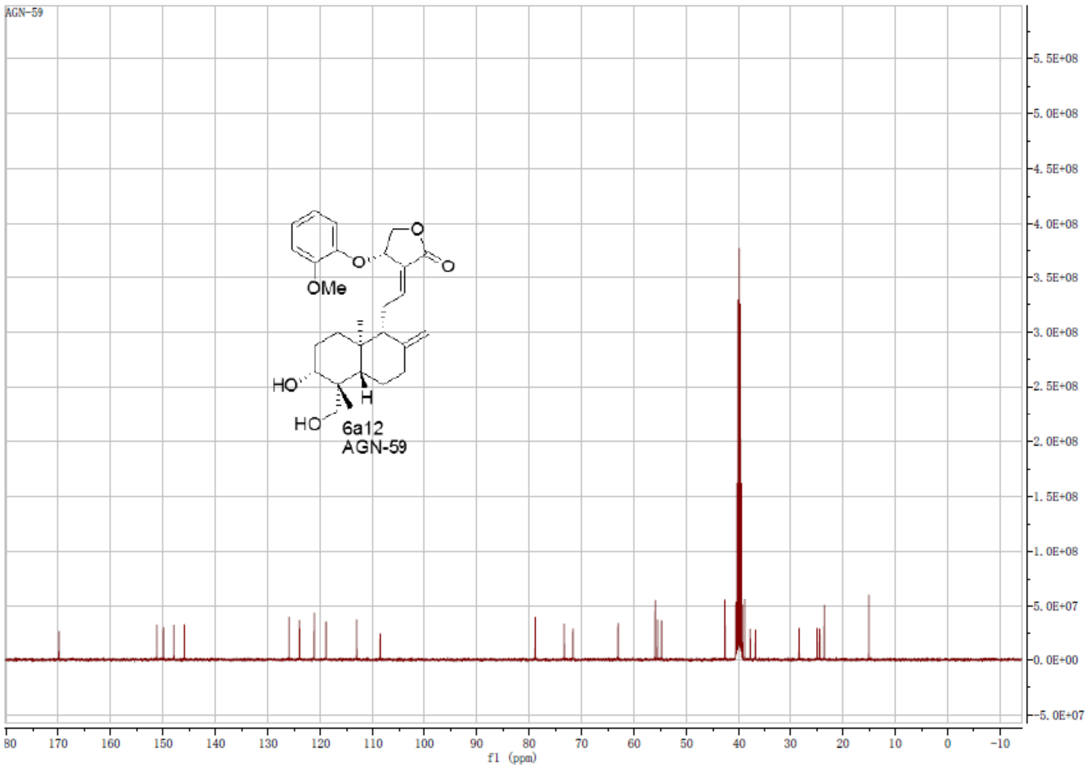

^{1}H NMR of **6b12**

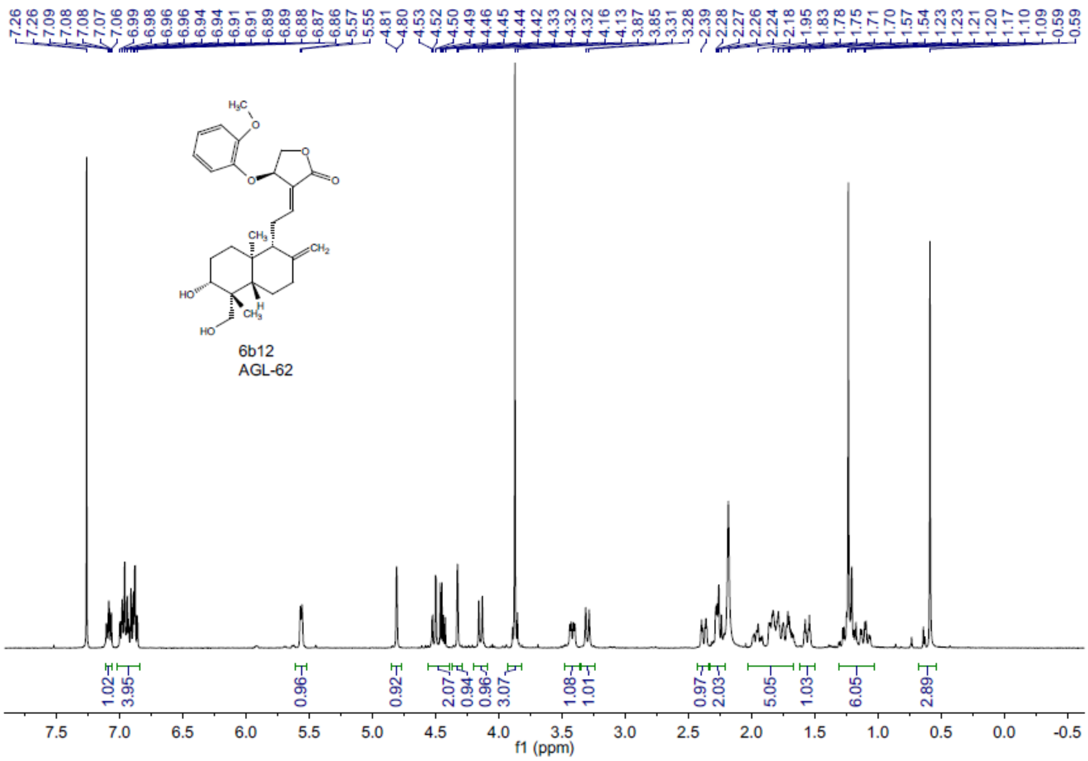

^{13}C NMR of **6b12**

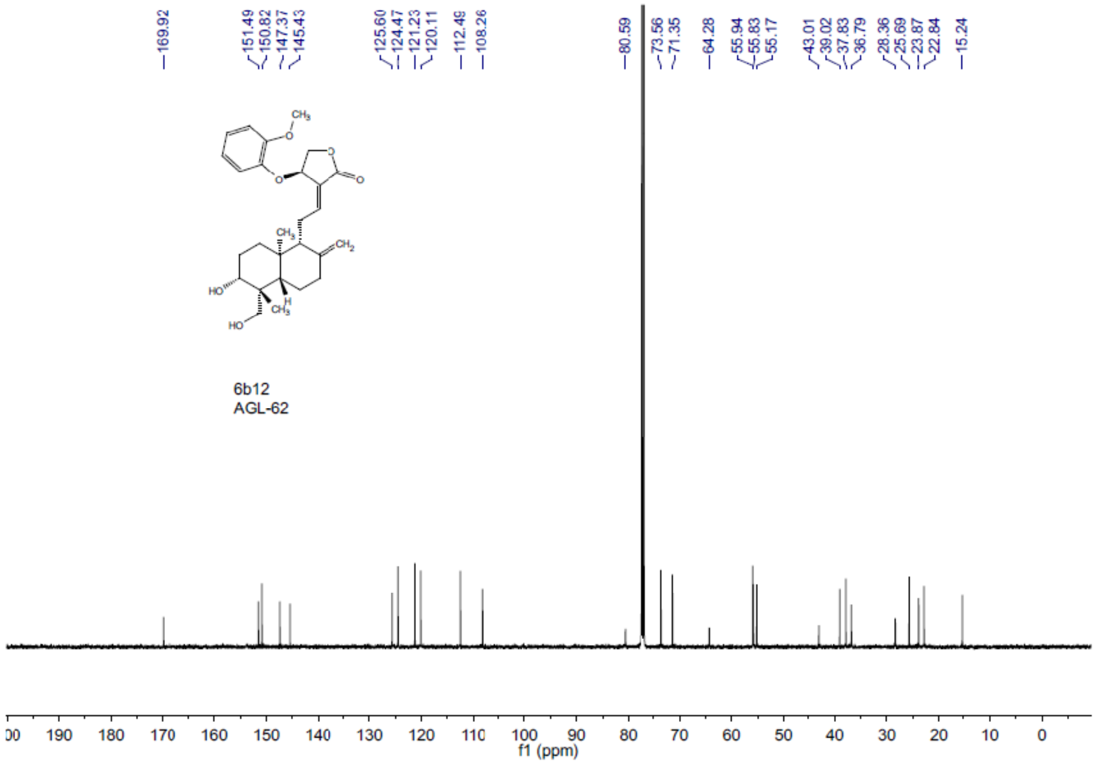

^{1}H NMR of **6b13**

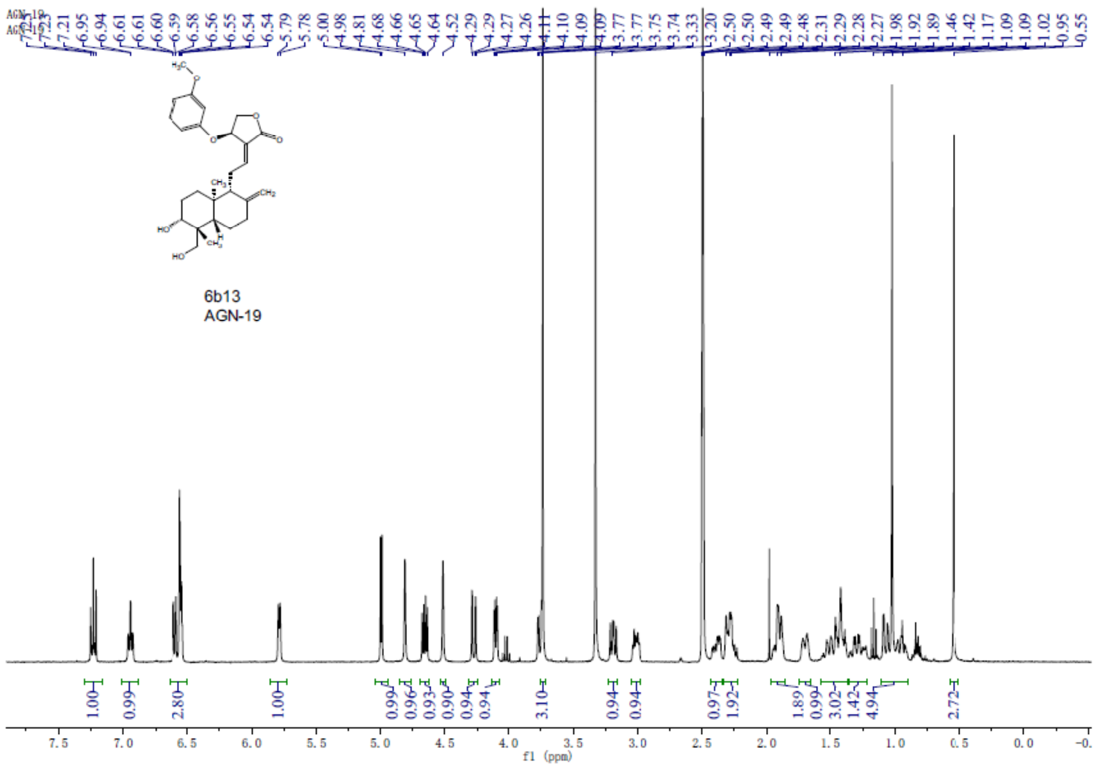

^{13}C NMR of **6b13**

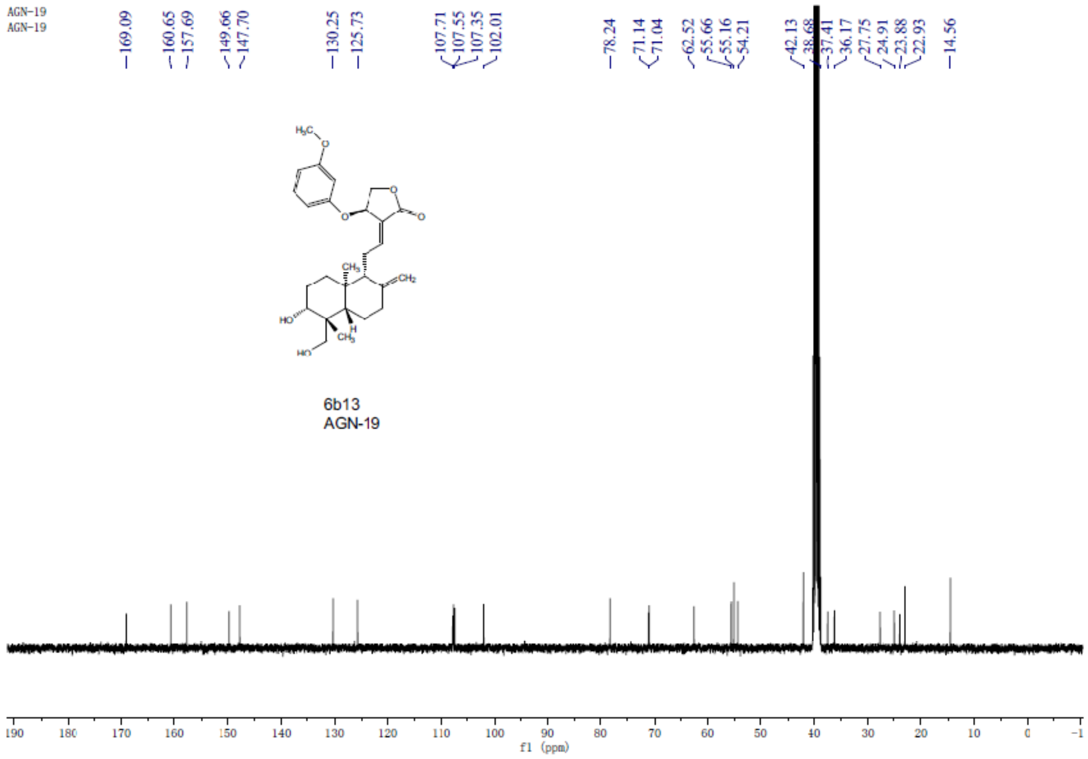

^{1}H NMR of **6b14**

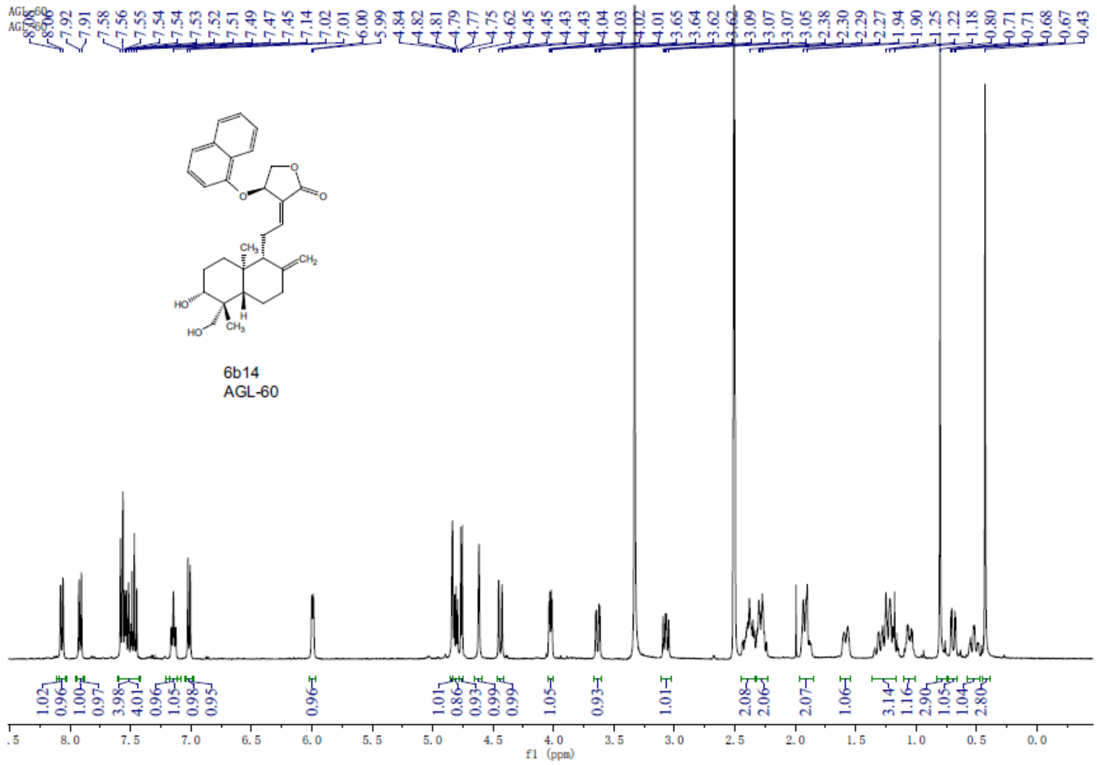

^{13}C NMR of **6b14**

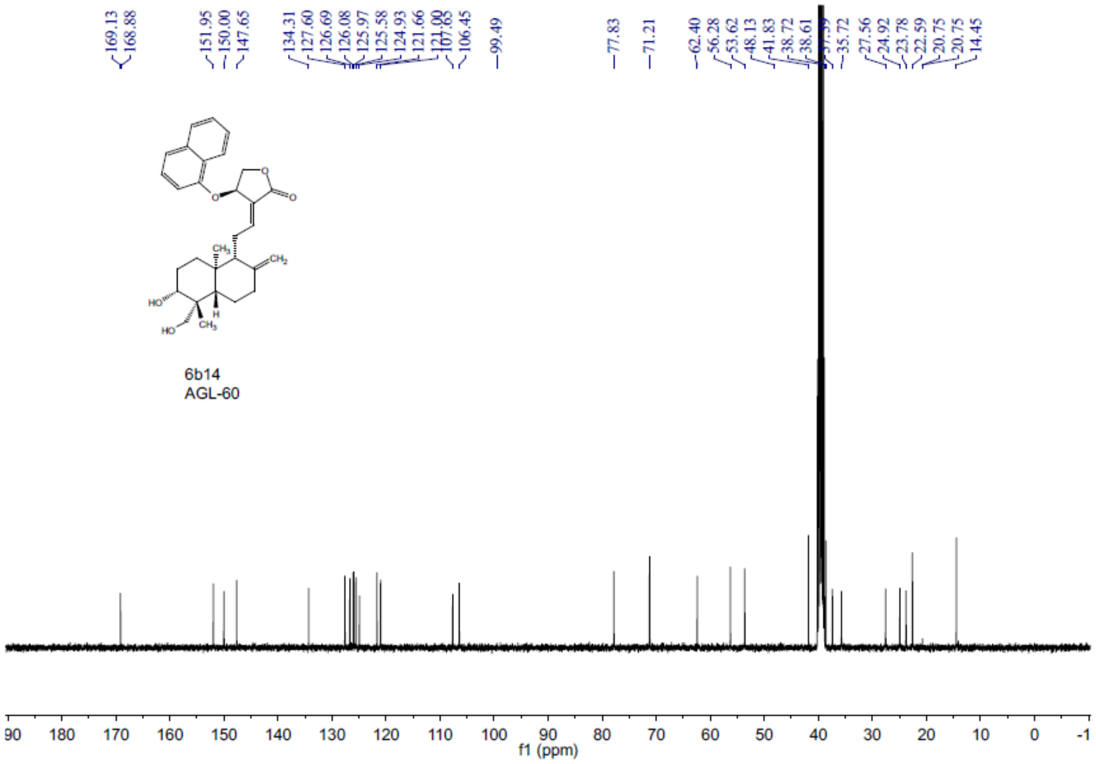

^{1}H NMR of **6a15**

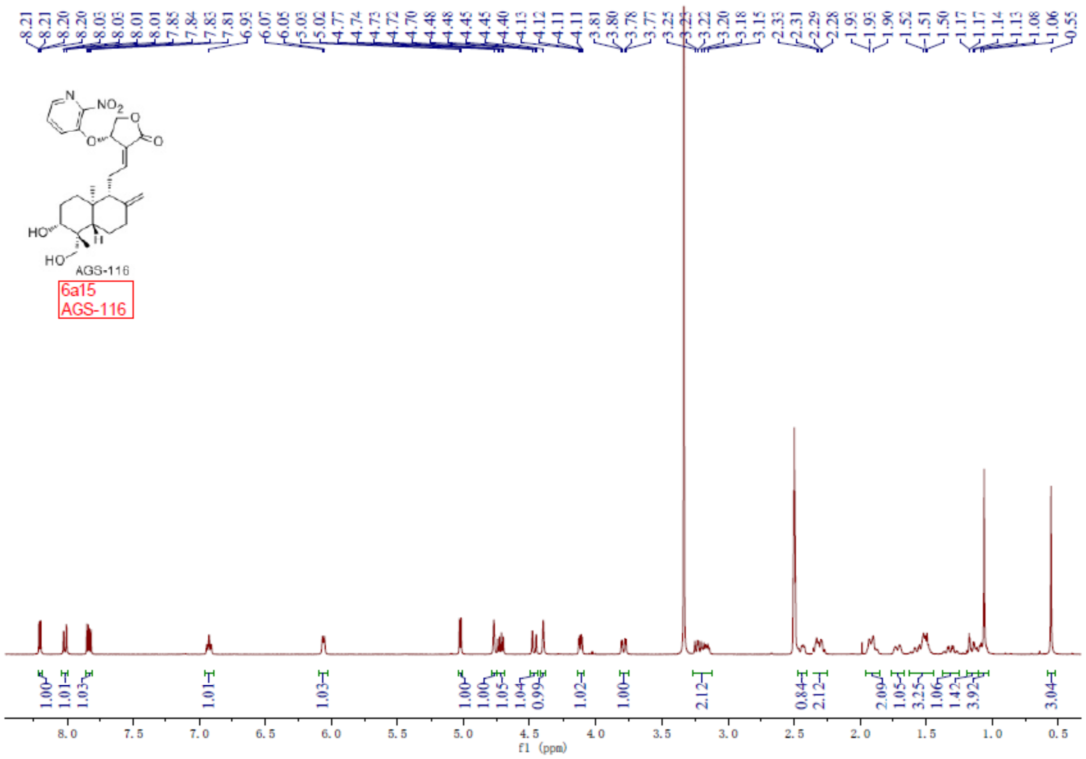

^{13}C NMR of **6a15**

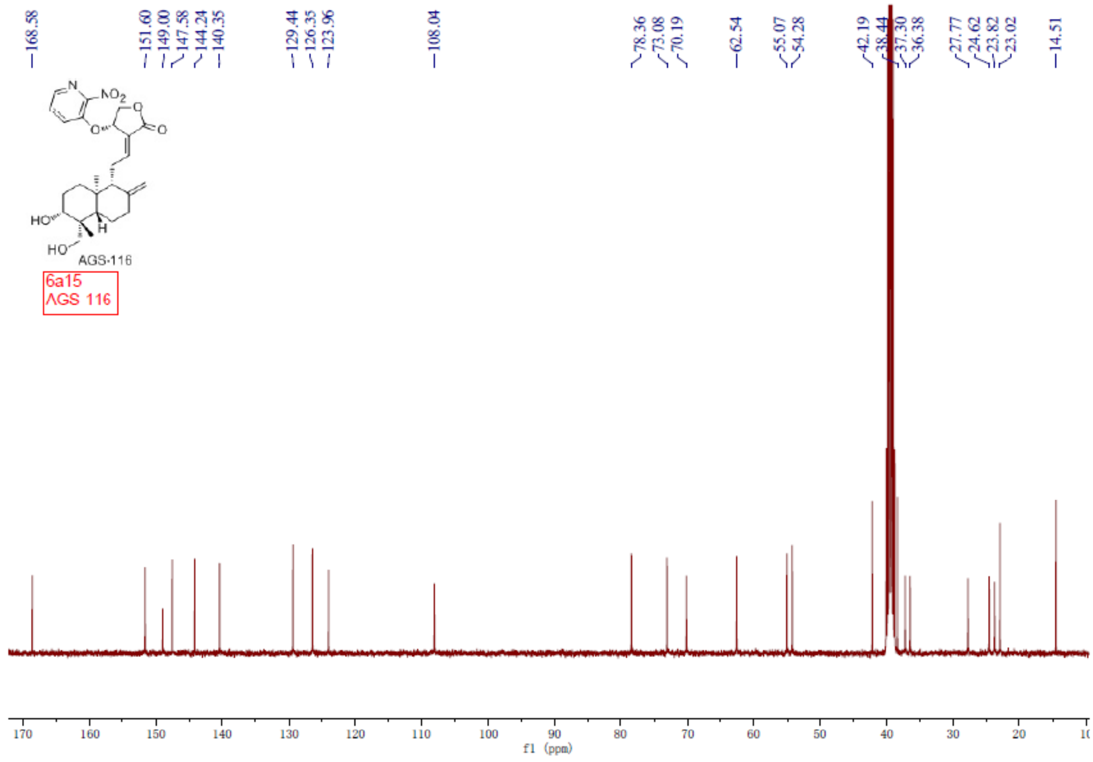

^{1}H NMR of **6b15**

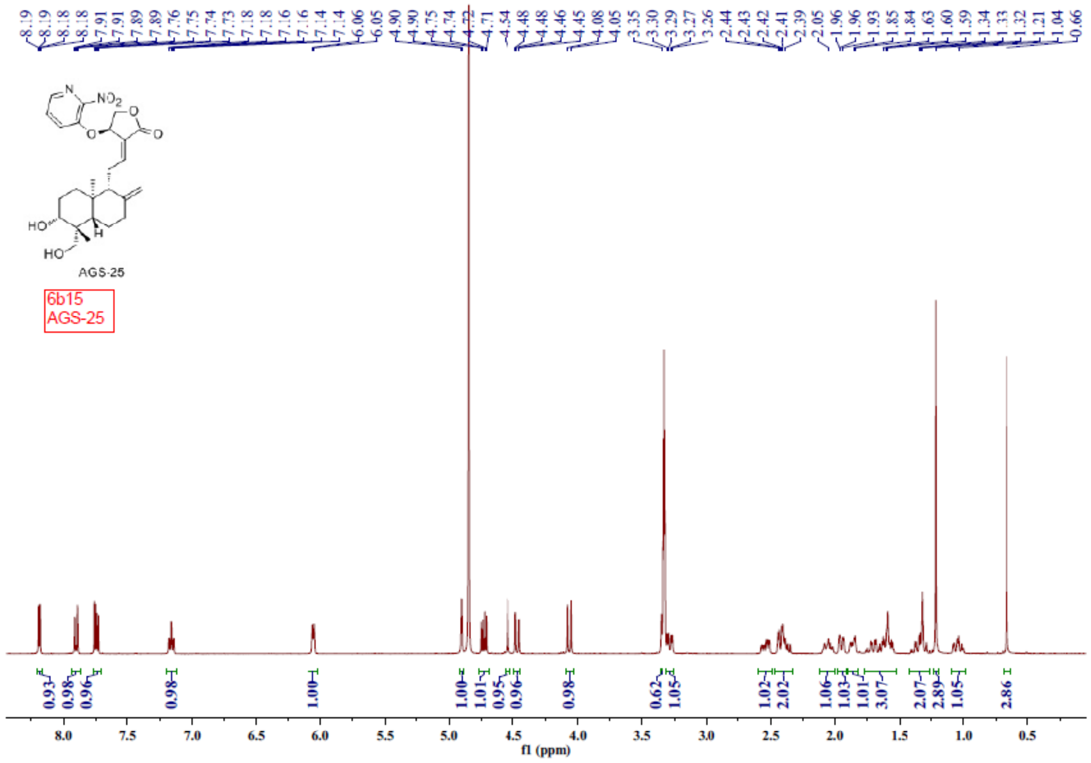

^{13}C NMR of **6b15**

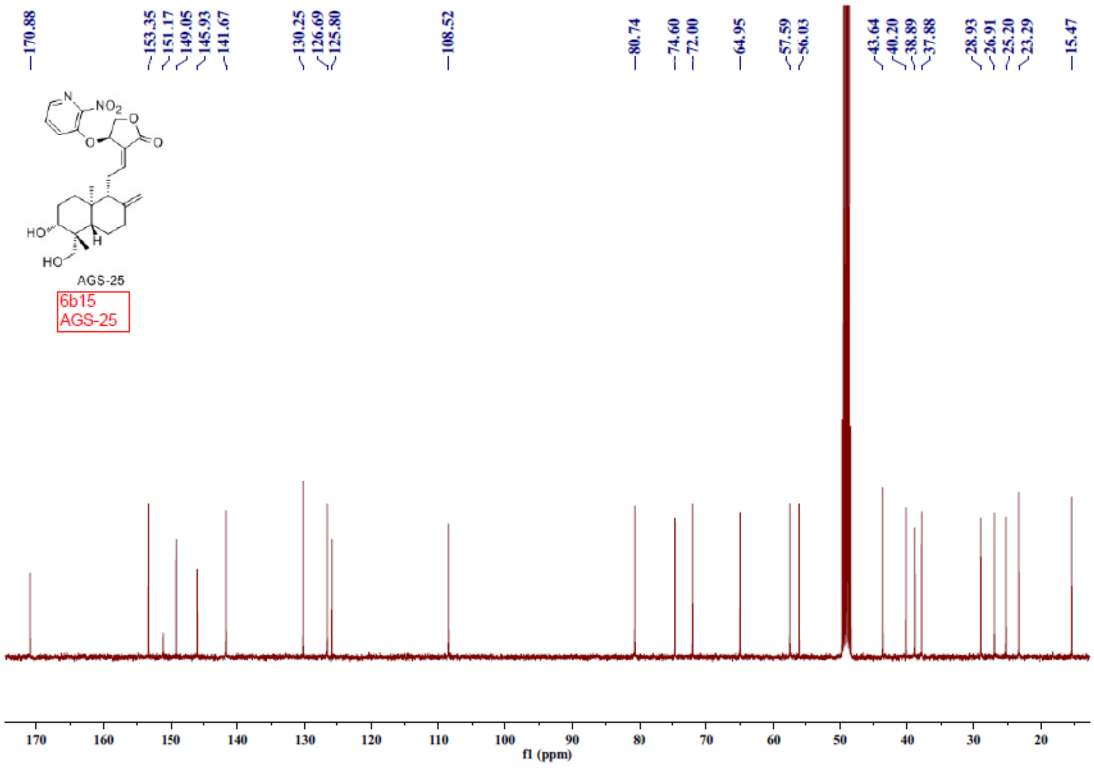

^{1}H NMR of **6a16**

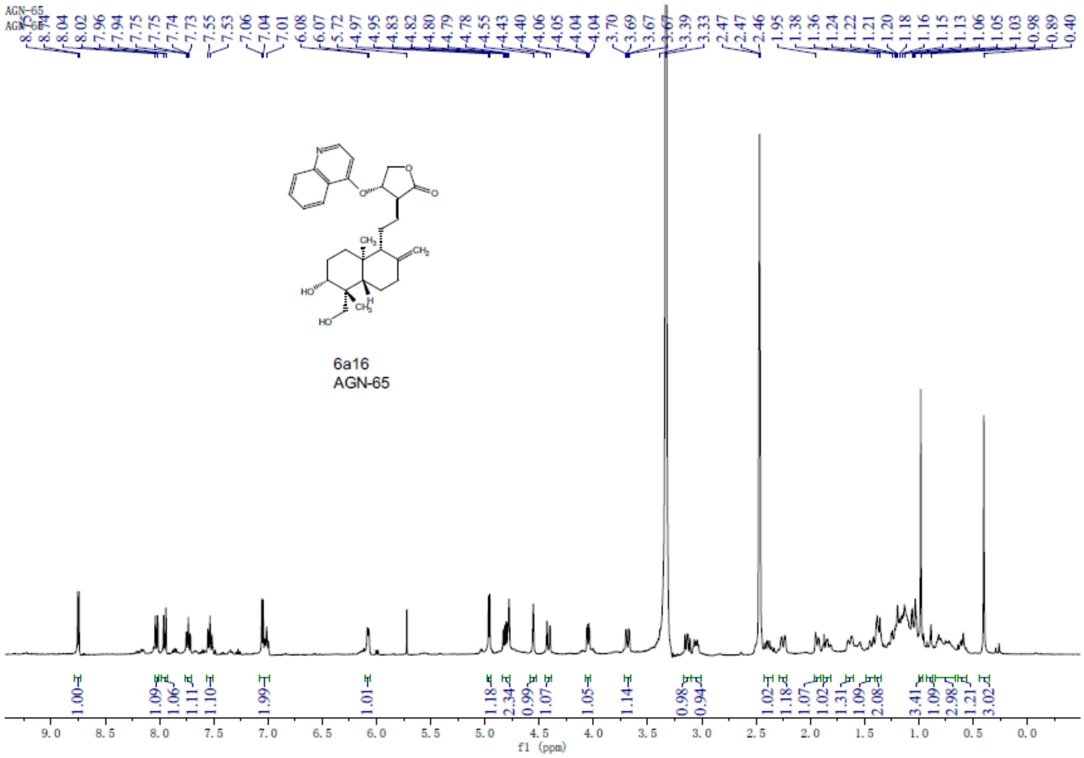

^{13}C NMR of **6a16**

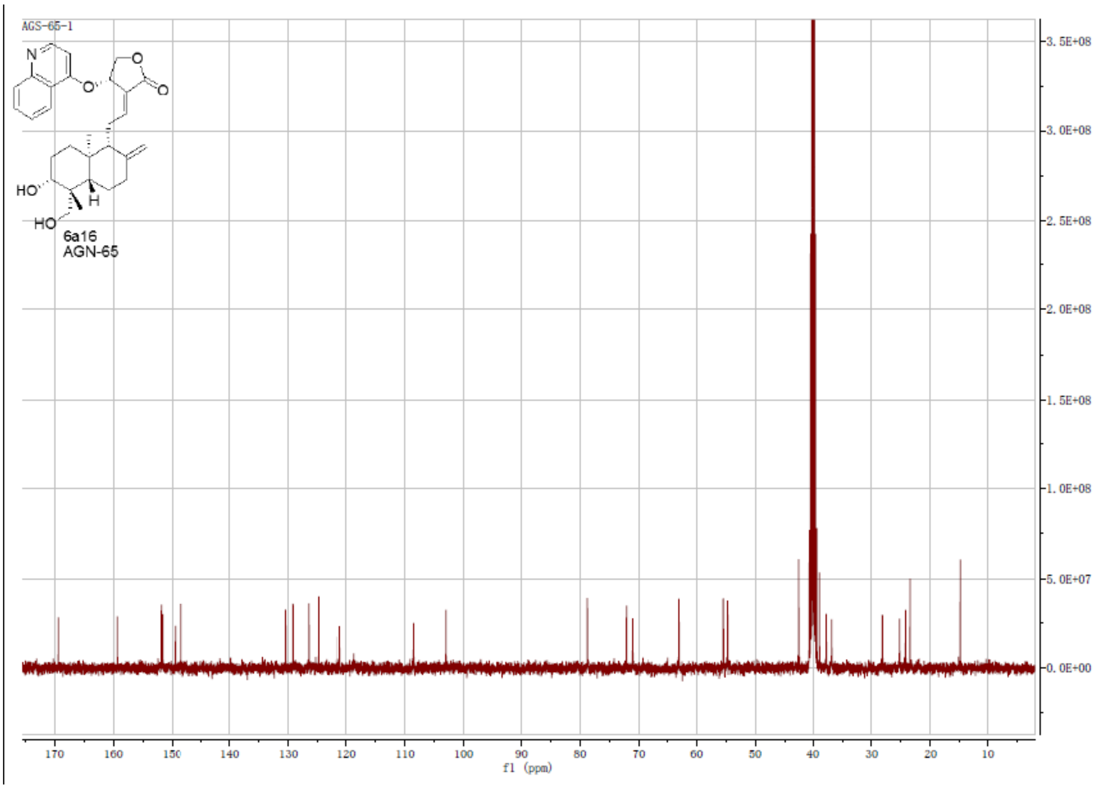

^{1}H NMR of **6b16**

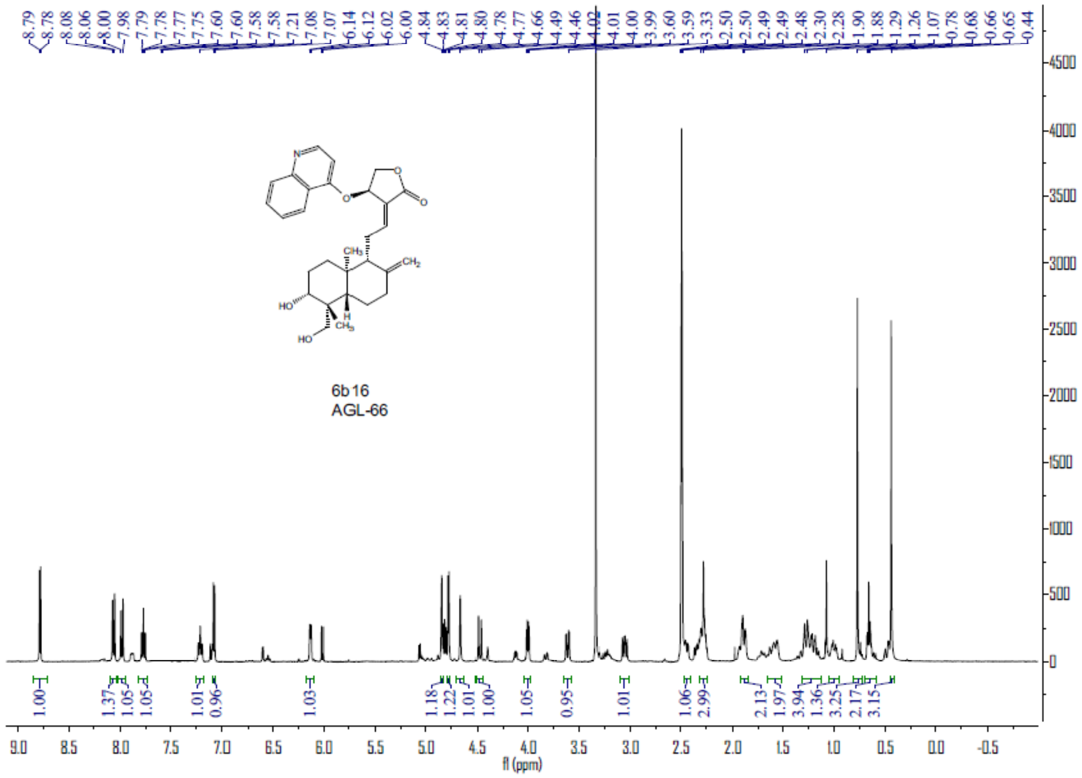

^{13}C NMR of **6b16**

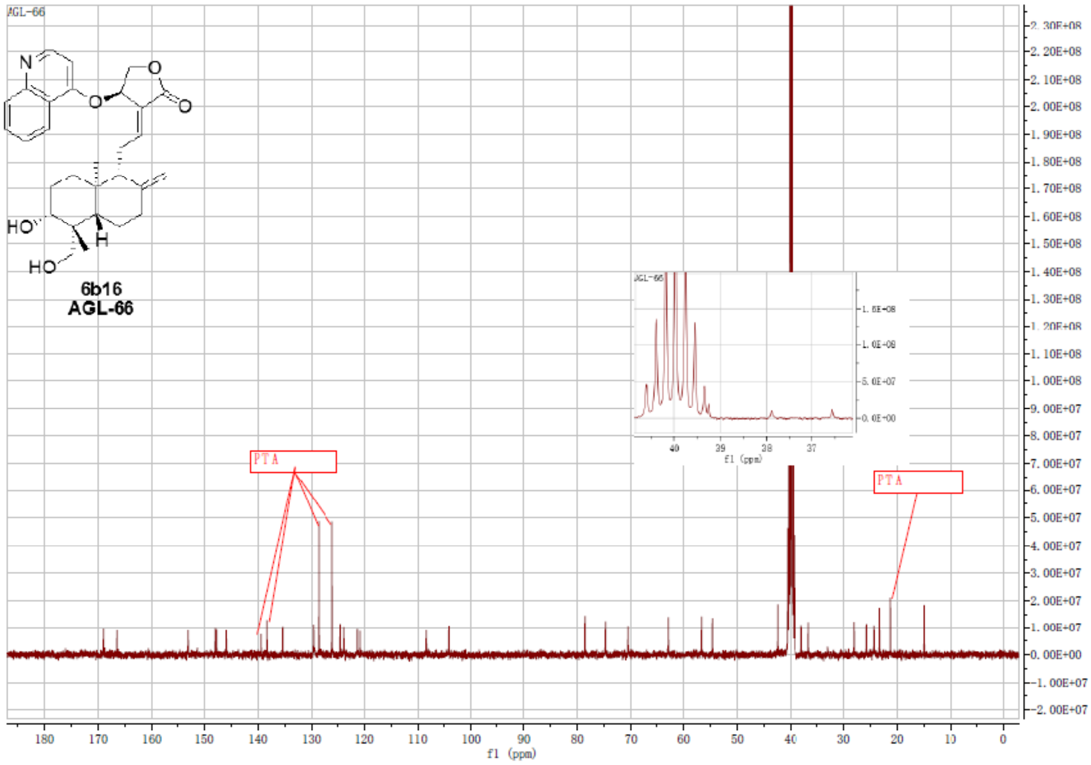

^{1}H NMR of **6a17**

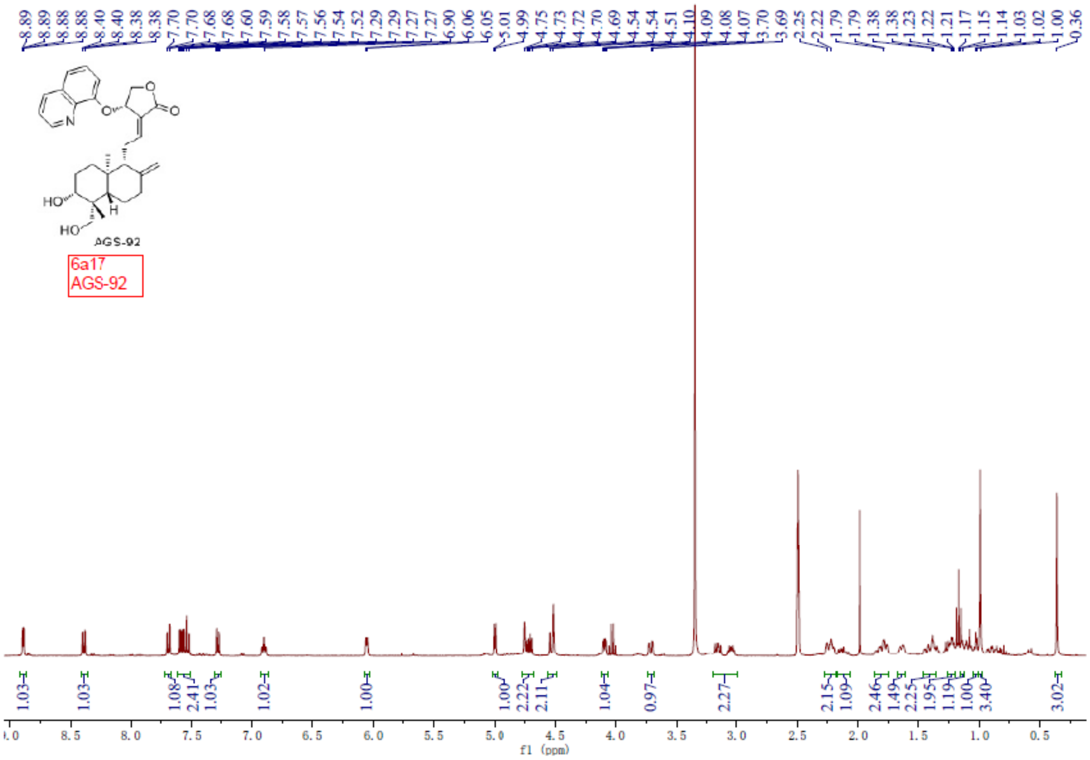

^{13}C NMR of **6a17**

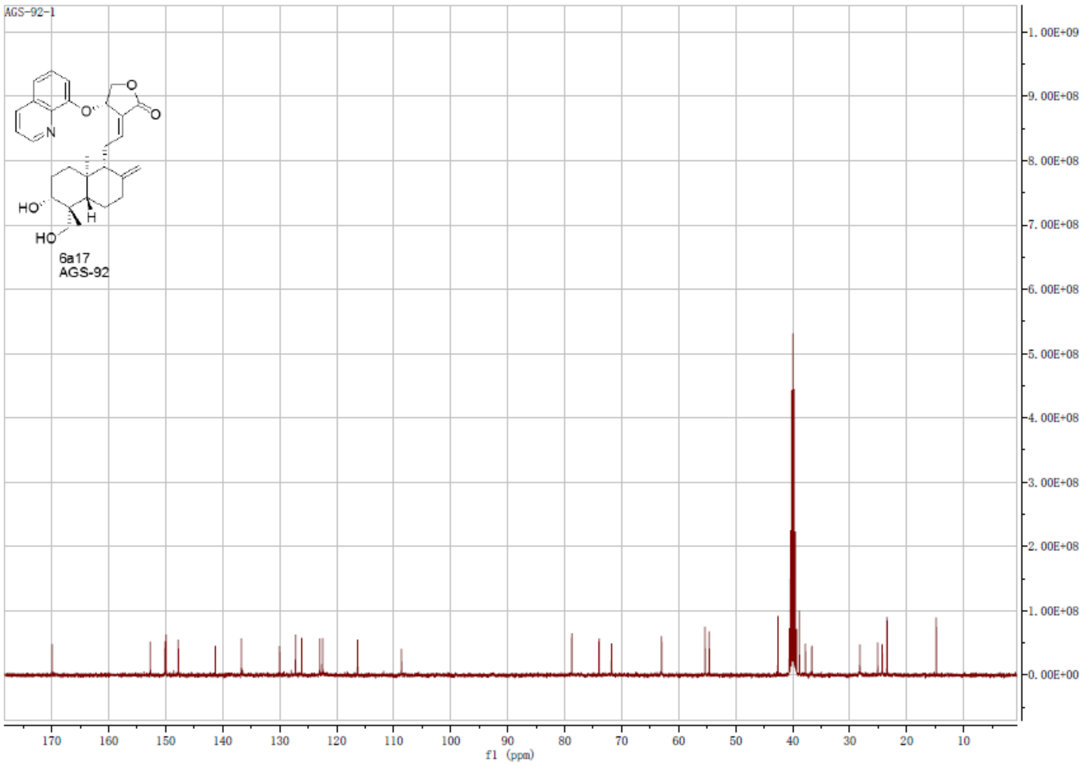

¹H NMR of **6b17**

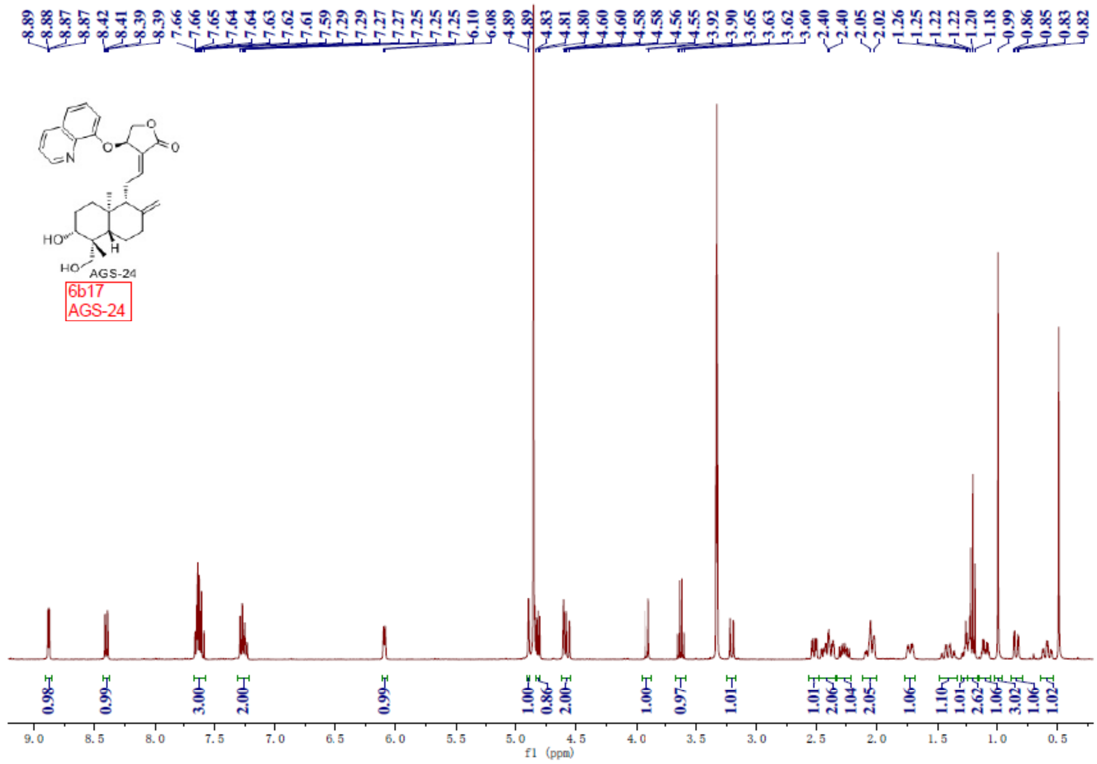

¹³C NMR of **6b17**

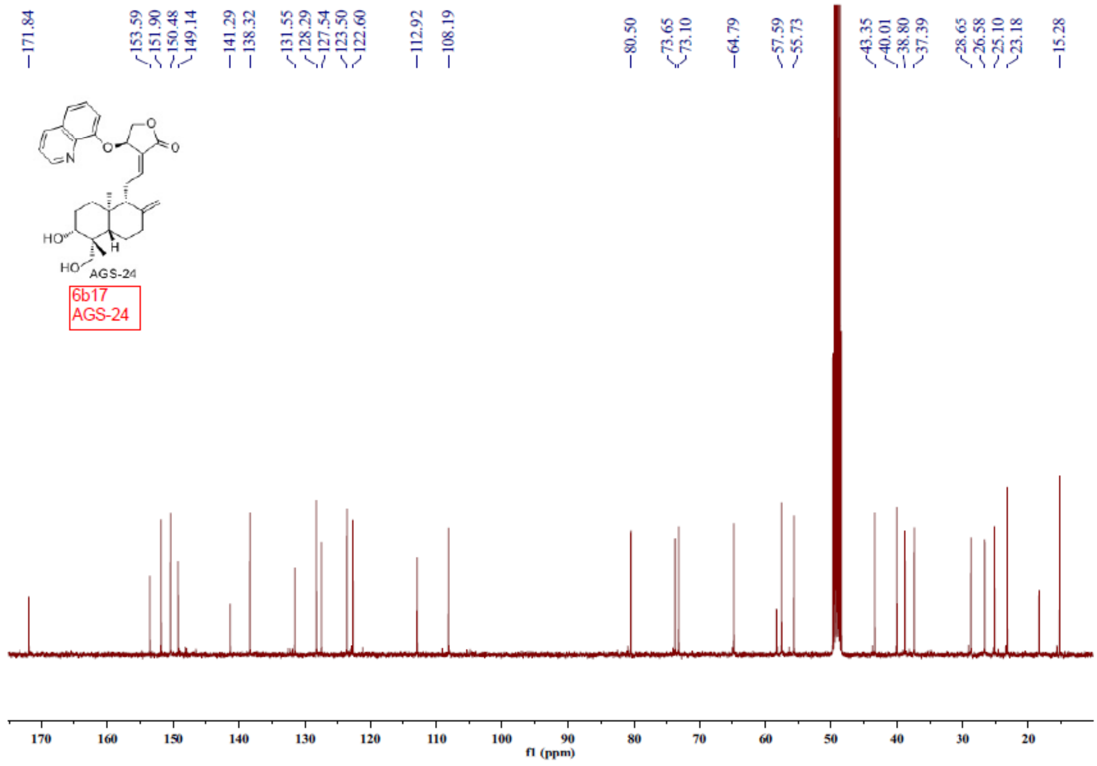

^{1}H NMR of **7b1**

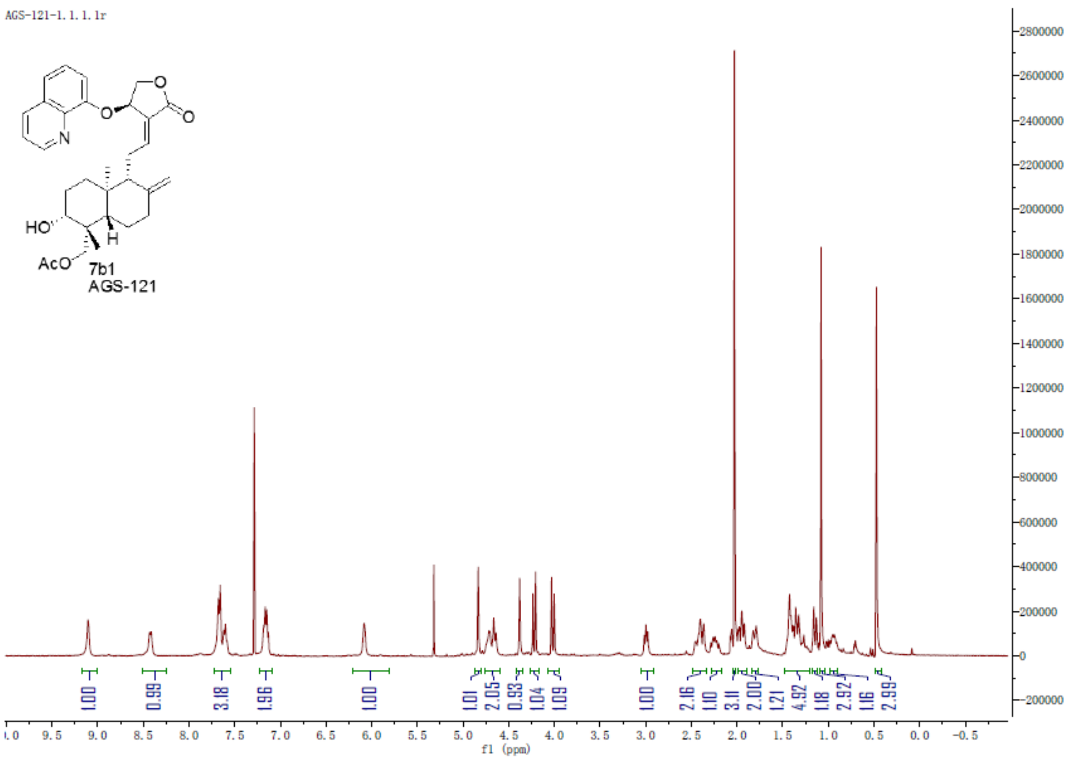

^{13}C NMR of **7b1**

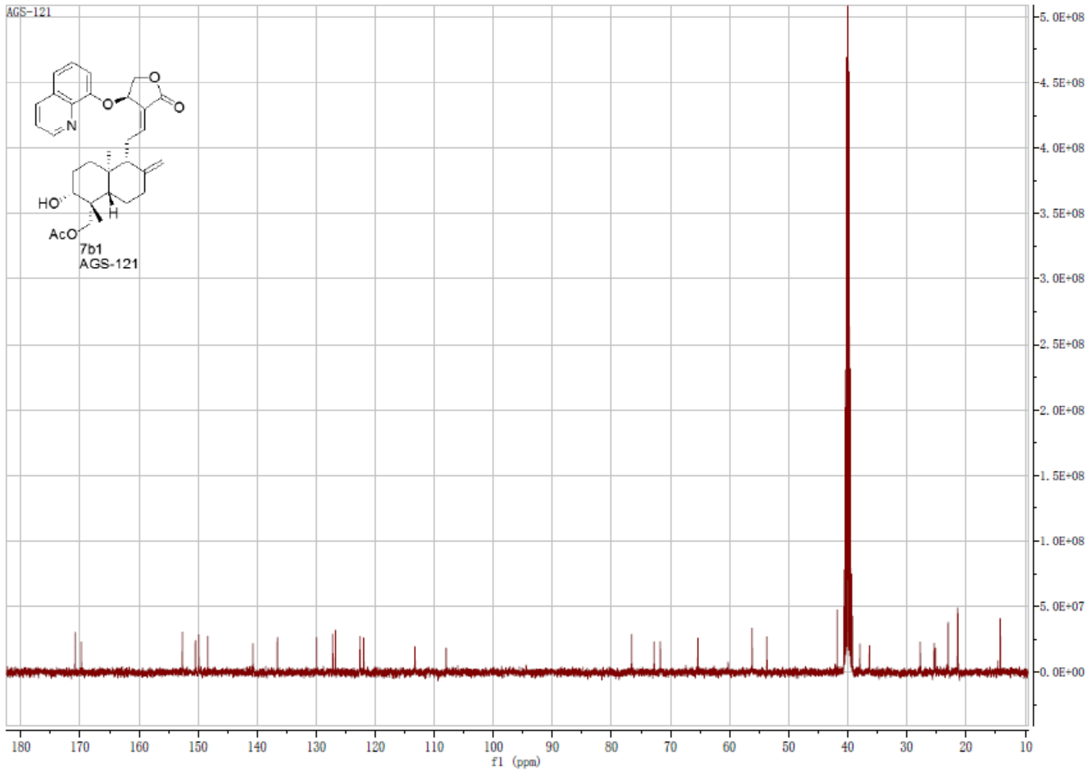

^{1}H NMR of **7b2**

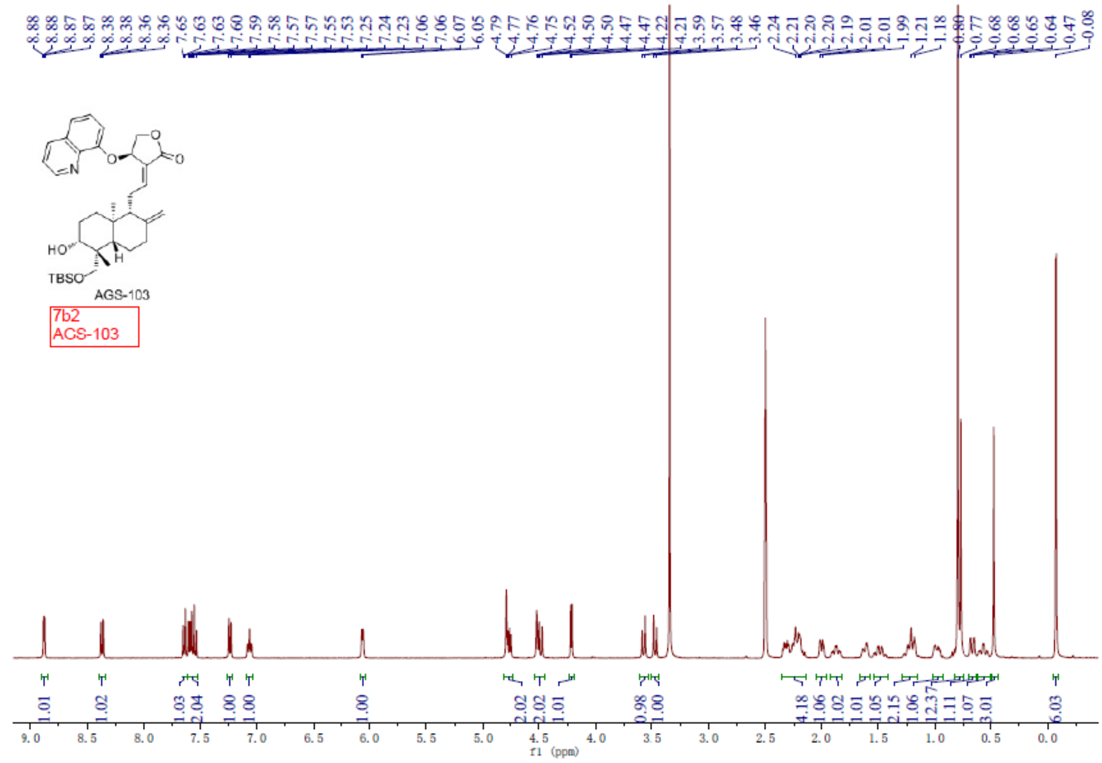

^{13}C NMR of **7b2**

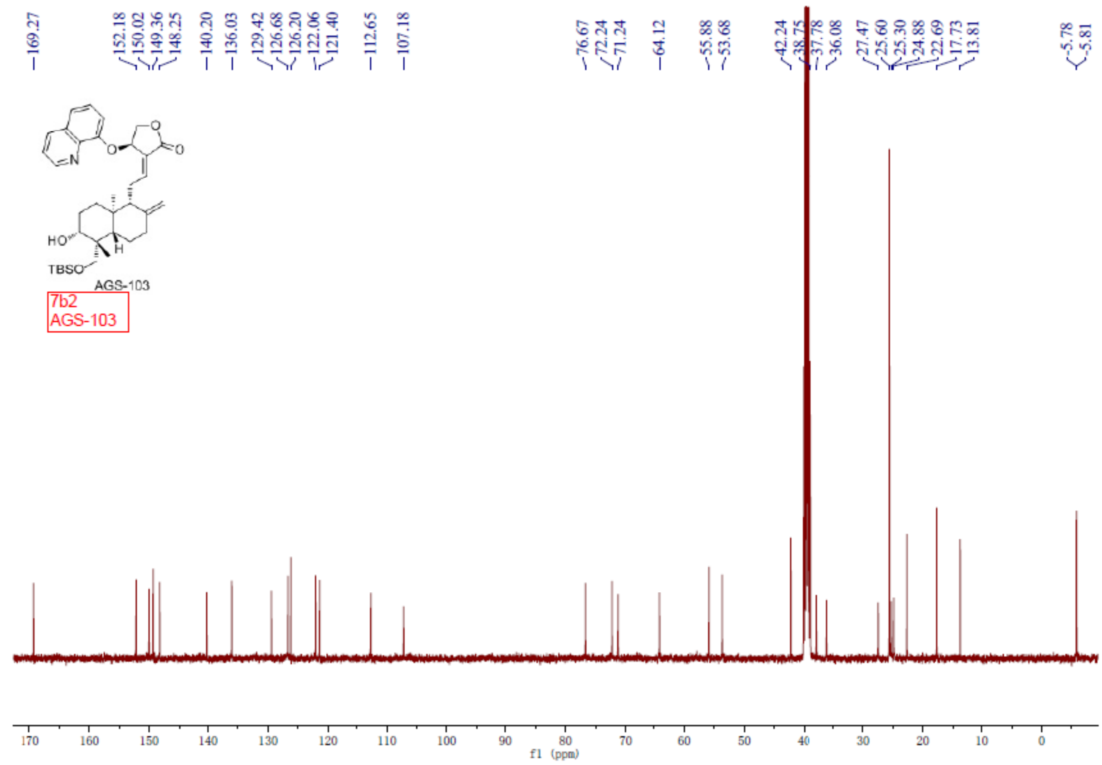

^{1}H NMR of **8b1**

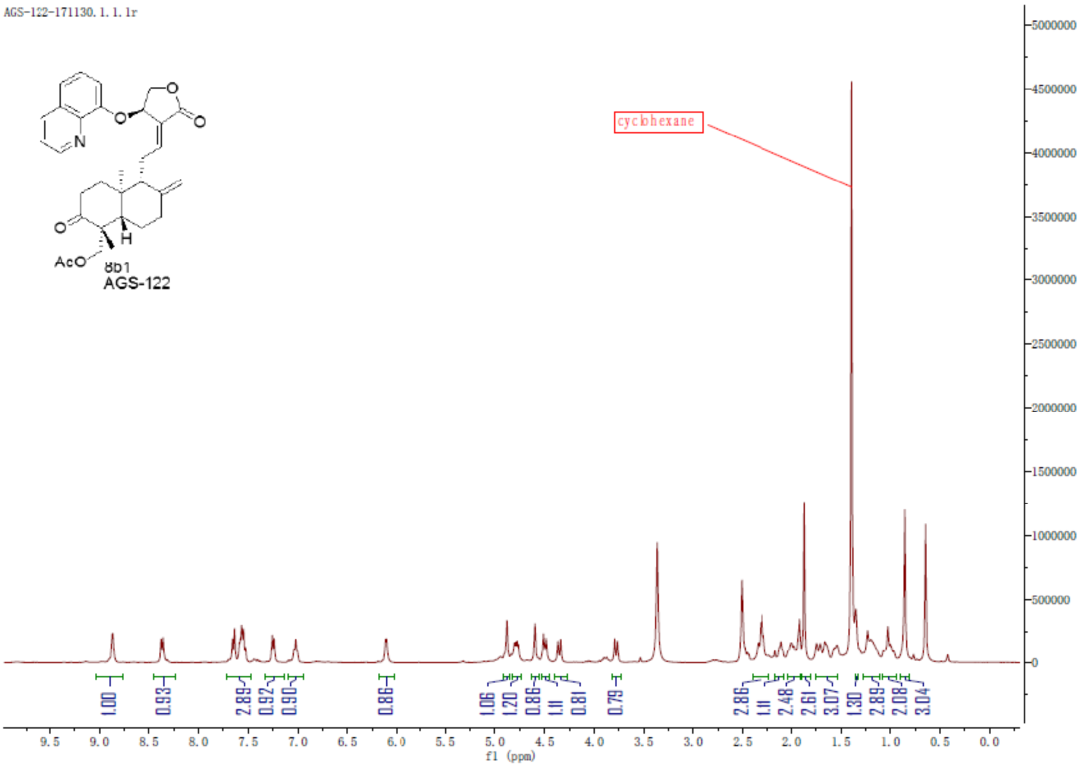

^{13}C NMR of **8b1**

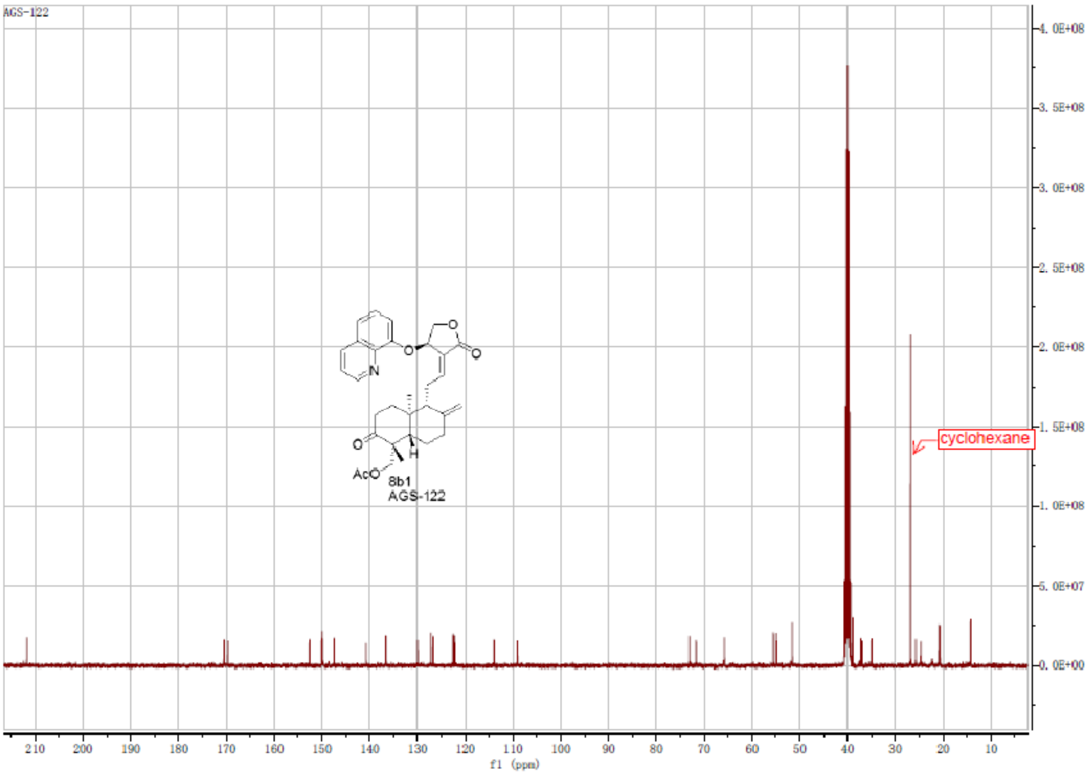

^{1}H NMR of **8b2**

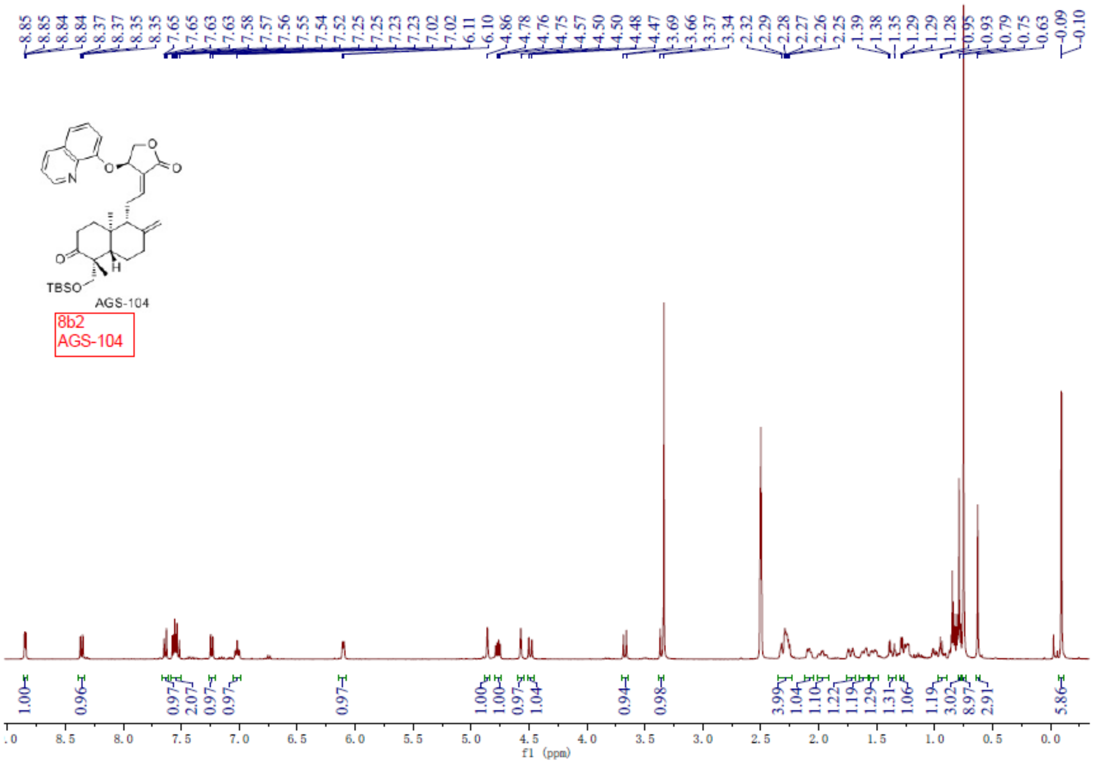

^{13}C NMR of **8b2**

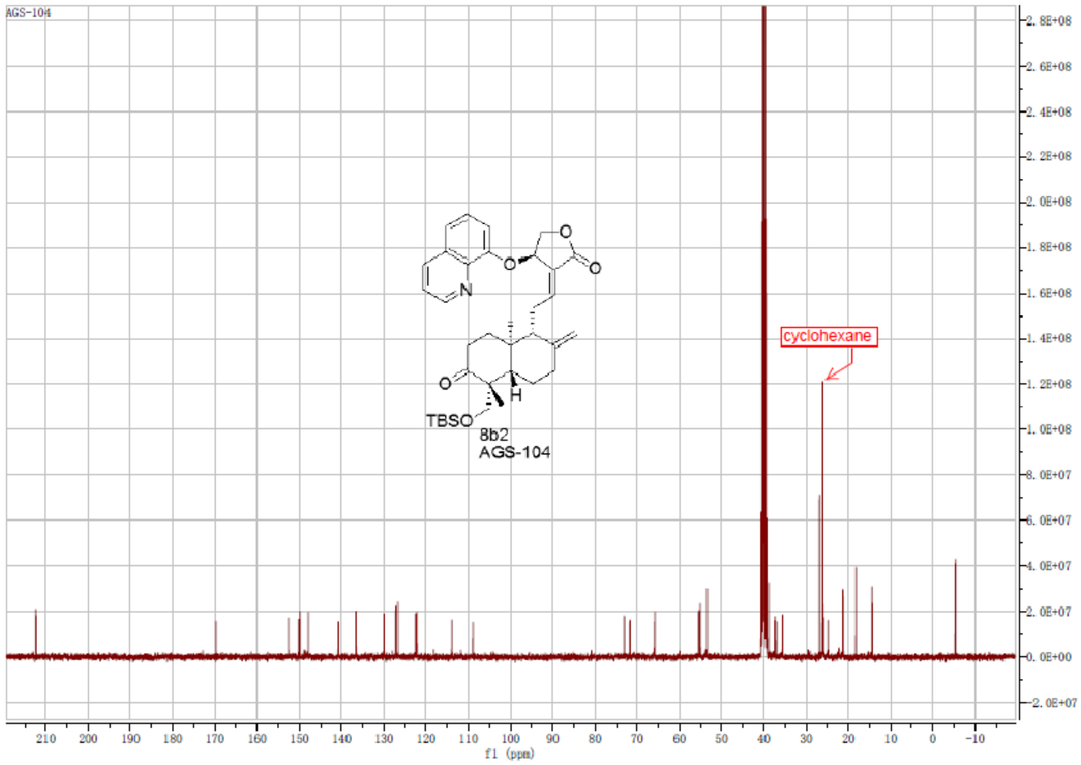

^{1}H NMR of **9b**

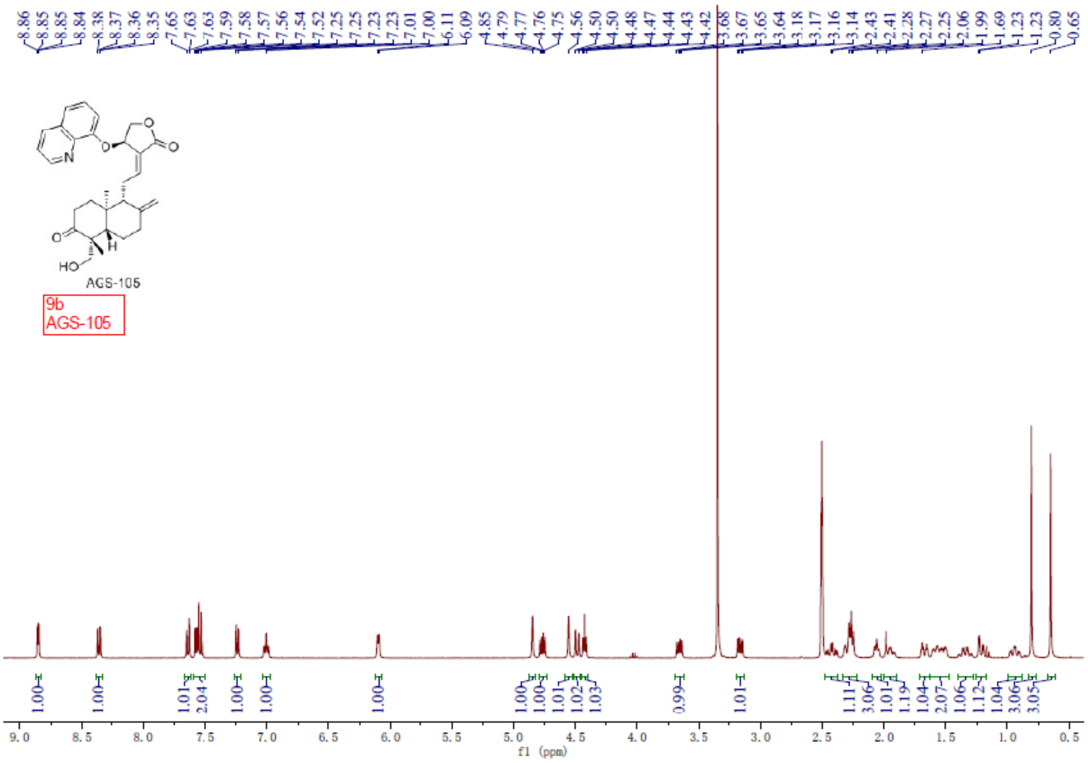

^{13}C NMR of **9b**

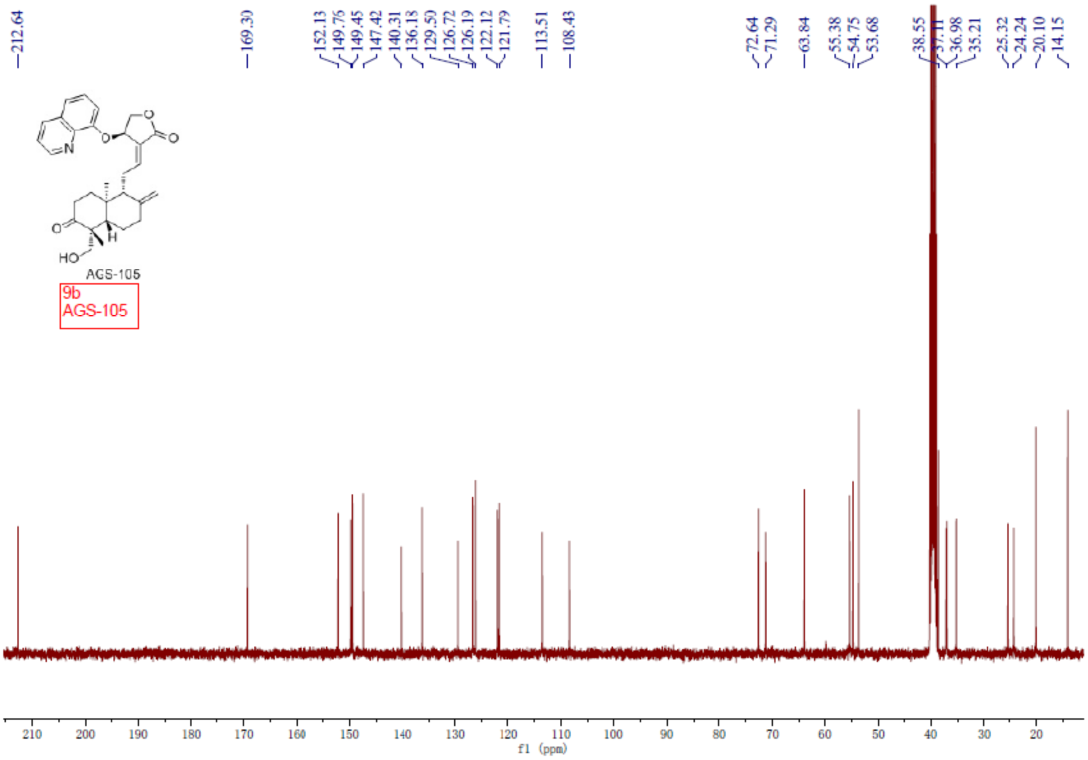

Supplement: RA-008-C8RA01063C-s001 [file RA-008-C8RA01063C-s001.pdf]
